# Arylpyrrolylidene-indanones as readily synthesised photoswitches offering dual- and single-colour fluorescence toggling with nearly quantitative *E*/*Z* isomerisation under visible light

**DOI:** 10.1039/d5sc04551g

**Published:** 2025-08-04

**Authors:** Satyajit Bera, Supriya Bhunia, Anirban Dolai, Sk Majid Box, Arpan Das, Subhas Samanta

**Affiliations:** a Department of Chemistry, University of Calcutta 92 A.P.C. Road Kolkata 700009 India sschem@caluniv.ac.in

## Abstract

Super-resolution fluorescence imaging and ultrahigh-density optical data storage, in particular, rely on the switches that demonstrate single- or (multi) dual-colour fluorescence toggling, with the latter option being more advantageous. However, a large number of switches are non-emissive owing to their fast photoisomerisation. Also, many fluorescent switches suffer from the necessity of high-energy UV light irradiation, low isomerisation yields, photodegradation, or the comparatively low fluorescence quantum yields. Herein, we present a new class of visible-light-responsive novel arylpyrrolylidene-indanone switches that permit high-intensity dual-colour fluorescence switching and quantitative photoisomerisation between exceptionally thermally stable *E*- and *Z*-isomers. The presence of an intramolecular hydrogen bond (–C=O⋯H–N) renders the *Z*-isomer significantly more emissive, achieving a quantum yield (*Q*_f_) of 0.5. These photoswitches are highly fatigue resistant and additionally display solvent viscosity-dependent fluorescence emission by the *E*-isomers as well. By speeding up the rotation of a C–C bond between pyrrole and C=C with the addition of two large substituents at sp^3^-C, guided by the DFT calculations, the emission of the *E*-isomer is almost completely lost, making these compounds single-colour (on/off) fluorescent photoswitches. Fluorescence photoswitching in 50% DMSO-PBS buffer works well, revealing the potential of these switches for biological applications, as well as for material uses.

## Abstract 2

Visible-light-responsive arylpyrrolylidene-indanones enable single- and dual-colour fluorescence photoswitching with (near) quantitative *E*/*Z* isomerization between two thermally stable photoisomers.

## Graphical abstract

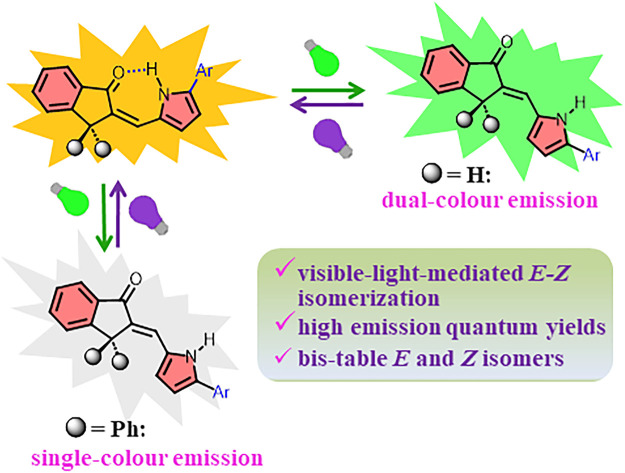

## Introduction

During the past 15 years, the advancement of photochromic molecules has been significant, particularly with respect to their development for a wide range of applications in different science disciplines.^1^ For example, applications in materials science include light-driven molecular machines,^2^ solar-thermal energy storage systems,^3^ and ultrahigh-density optical data recording and storage devices,^4^ among others, while in biomedical research, they have become a key tool to remotely and noninvasively regulate biological processes under *in vivo* settings^5^ and to visualize subcellular processes at the nanoscale with the aid of super-resolution fluorescence imaging.^6^ The super-resolution fluorescence imaging that caused a revolution in the understanding of biological systems and the ultrahigh-density optical data storage essentially require a photochromic substance that may fluoresce in one of its two isomeric states, serving as a single-colour fluorescent switch, or fluoresce in both isomeric states, forming a dual-colour fluorescent photoswitch.^7^ The latter type is more advantageous than the former, as it allows elimination of background autofluorescence in bioimaging and permits storage of double bits of electronic information on a single pixel of optical memory systems.^8^

Photoswitches, such as spiropyrans and diarylethanes (DAEs), which alter π-conjugations *via* the pericyclic mechanism of isomerisation, exhibit single-colour fluorescence emission typically by the highly conjugated isomers.^9^ To engineer (multi)-double-colour fluorescent photoswitches, these monolithic switches are often combined with a variety of established fluorophores including small-molecule dyes,^10^ proteins^11^ or luminescent nanoparticles,^12^ which have permitted modulation of the emissive property reversibly by Förster resonance energy transfer (FRET) or photoinduced electron transfer (PeT) from the excited fluorophore to the photoswitch.^8,13^ However, these composite fluorescent photoswitches often suffer from (i) their bigger sizes with complicated molecular design rules, (ii) the interference of the intrinsic fluorescence emission with the energy transfer from the excited state, and (iii) the gradual decomposition of the composite dye, raising the fluorescence background.^14^ Additionally, the constituent spiropyran and DAE switches suffer from the necessity for toxic UV light for the isomerisation, at least in one direction,^15^ and the poor photofatigue resistance of the DAE switches, in particular.^16^ Thus, the development of monolithic photochromic small molecules featuring dual-colour fluorescence photoswitching is of high value.^8^ Also, these compounds need to show (i) complete isomerisation, (ii) strong resistance to fading from light, (iii) isomerisation when exposed to visible light, ideally in the near IR or IR ranges, and (iv) fluorescence photoswitching in water for use in biological applications.^17^

**Fig. 1:**
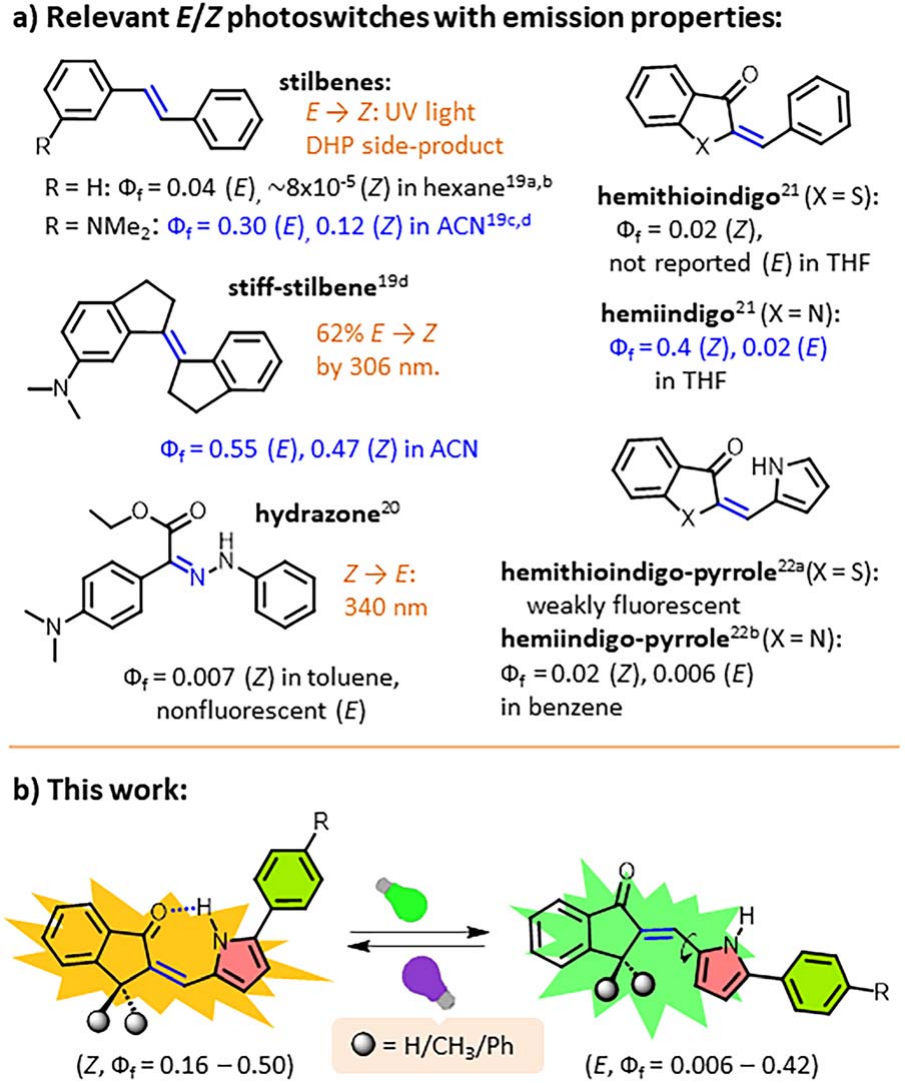
(a) Structures of relevant fluorescent *E*/*Z* photoswitches and (b) photoswitchable fluorescent arylpyrrolylidene-indanones of this study.

The *E*/*Z* photoswitches that induce large geometrical changes *via* isomerisation around N=N, C=C, or C=N bonds, including azobenzenes,^18^ stilbenes,^19^ hydrazones,^20^ hemithioindigos,^21^ hemithioindigo-pyrroles,^22^ aurones^23^ and imines^24^ are either nonfluorescent or weakly fluorescent in one of the two isomeric states (Fig. 1a). The fact that both *E*/*Z* isomerisation and emission occur from a common excited state, along with the much faster rate of the former process (femtoseconds or picoseconds) compared to the latter (nanoseconds), restricts the radiative deactivation of the excited state, which results in suppressed fluorescence emission.^7*a*,18^

The slowing down of photoisomerisation of *cis*-(*Φ*_f_ = ∼8 × 10^−5^ in hexane) and *trans*-stilbenes (*Φ*_f_ = 0.04)^19*a*,*b*^ by the incorporation of *N,N*-dimethylamino substitution^19*c*,*d*^ at the *meta*-position is known to enhance the fluorescence intensity of both *cis*-(*Φ*_f_ = 0.12 in ACN), and *trans*-isomers (*Φ*_f_ = 0.30) (Fig. 1a). However, these switches face a big problem because they essentially produce unwanted side products such as dihydrophenanthrene (DHP) upon photolysis of the *cis* stilbene.^19*e*^ Inhibition of the DHP formation involving stiff-stilbene (Fig. 1a), as introduced by Yang and coworkers,^19*d*^ improved the fluorescence intensity of the *cis*-stilbene substantially (*Q*_f_ = 0.21). Alarmingly, the isomerisation yields were very poor (62% *E* → *Z* at 306 nm and 72% *Z* → *E* at 400 nm in ACN). Aprahamian's group recently reported an interesting push–pull hydrazone derivative (Fig. 1a) that exhibited on (*Z*)/off (*E*) fluorescence photoswitching in solution as well as solid states; however, its emission quantum yields were restricted to only varying between 0.007 and 0.03 in different solvents and 0.27 in the solid state.^20^ Moreover, all of these modified stilbene and hydrazone switches require UV light (306–340 nm) for inducing isomerisation, at least in one direction, which limits their applications as fluorescent photoswitches. In 2025, in-depth fluorescence photoswitching studies on hemithioindigo and hemiindigo switches (Fig. 1a),^21^ conducted by Szumna and co-workers, revealed that both compounds exhibit single-colour fluorescence switching in THF solvent (*Φ*_f_ = 0.4 for *Z* and 0.02 for *E* isomers of hemiindigo; *Φ*_f_ = 0.02 for *Z* isomer of hemithioindigo THF). Replacement of the stilbene phenyl ring of these compounds with a pyrrole ring leading to thioindigo-pyrrole and hemiindigo-pyrrole switches reduced the emission drastically (Fig. 1).^22^

**Chart 1:**
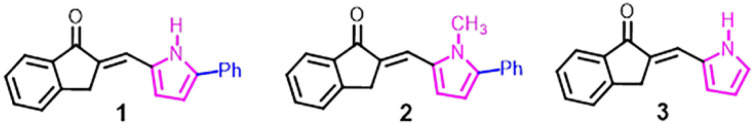
Structures of series I pyrrolylidene-indanone.

Pyrrolylidene-indanone has been an underutilised *E*/*Z* photoswitch due to the poor *E* → *Z* isomerisation yield obtained from a few scattered reports.^25^ Here, we present a new class of arylpyrrolidine-indanone (API) photoswitches (Chart 1), which permit dual-colour fluorescence photoswitching (with a maximum *Φ*_f_ of 0.5) *via* isomerisation of the C=C bond. High to (near) quantitative *E*/*Z* photoconversion in both directions is achieved under visible light illumination in both directions. The presence of an intermolecular H-bond between pyrrole NH and indanone C=O in the *Z*-isomers increases their fluorescence emission and thermal stability, rendering them P-type photoswitches at ambient temperature. The photophysical and photochemical properties have been tuned by introducing electroactive substituents in the phenyl ring attached to the pyrrole moiety. Remarkably, adding large groups like dialkyl or diphenyl at the sp^3^-C next to the C=C bond almost completely stops the *E*-isomers from glowing, making the photochromes work as single-colour fluorescent switches that can turn on and off. These switches constitute a rare example of photoswitches, which allow intense single- and dual-colour fluorescence photoswitching with (near) quantitative isomerisation yields between two thermally stable photoisomers under visible light.

## Results and discussion

**Scheme 1:**
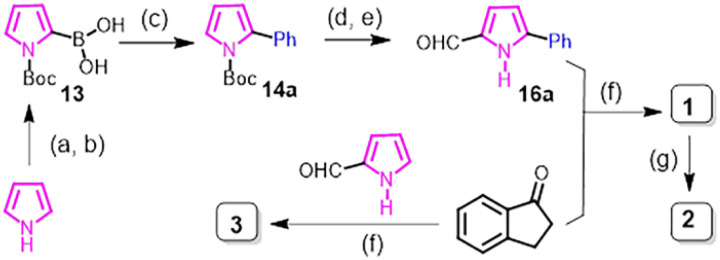
Sythesis of series I APIs. Reagents and conditions: (a) (Boc)_2_O, Et_3_N, DMAP, DCM, 6 h, rt.; (b) *n*-BuLi, DIPEA, trimethyl borate, dry THF, −78 °C; (c) Pd(PPh_3_)_4_, Na_2_CO_3_, DME/H_2_O (3 : 1), 90 °C, 18 h; (d) NaOEt, THF, rt., 2 h; (e) POCl_3_, DMF, rt., 3 h; (f) piperidine, toluene, 110 °C, 18 h; (g) MeI, NaH, DMF, rt., 2 h.

To investigate photophysical and photochemical properties of APIs, we initially designed pyrrolylidene-indanone 1 (Scheme 1), which was conveniently synthesised by following a six-step reaction sequence starting from commercially available pyrrole and 1-indanone (Scheme 1). After Boc protection of pyrrole NH, it was transformed into *N*-Boc-2-pyrroleboronic acid 13 by reacting with trimethyl borate in the presence of LDA, which was then subjected to the Suzuki cross-coupling reaction with bromobenzene to afford 2-phenyl-*N*-Boc-pyrroles 14a. Boc deprotection followed by formylation at the C2 position using the Vilsmeier–Haack reaction protocol led to the formation of 5-phenyl-1*H*-pyrrole-2-carbaldehyde, 16a. This building block was then subjected to an aldol condensation reaction with commercially available 1-indanone under refluxing conditions at 110 °C in toluene solvent to afford the desired API 1 as a mixture of 70% *Z*- and 16% *E*-isomers. The existence of both isomers at such a high temperature implies that both isomers have comparable thermodynamic stability and that the energy barrier for thermal isomerisation (in both directions) is too high to permit any isomerisation at room temperatures. The preference for the formation of the *Z*-isomer (usually less thermally stable) over the *E*-isomer is presumably due to stabilisation caused by an intramolecular H-bond between the pyrrole N–H and indanone C=O group (Fig. 1b).

**Fig. 2:**
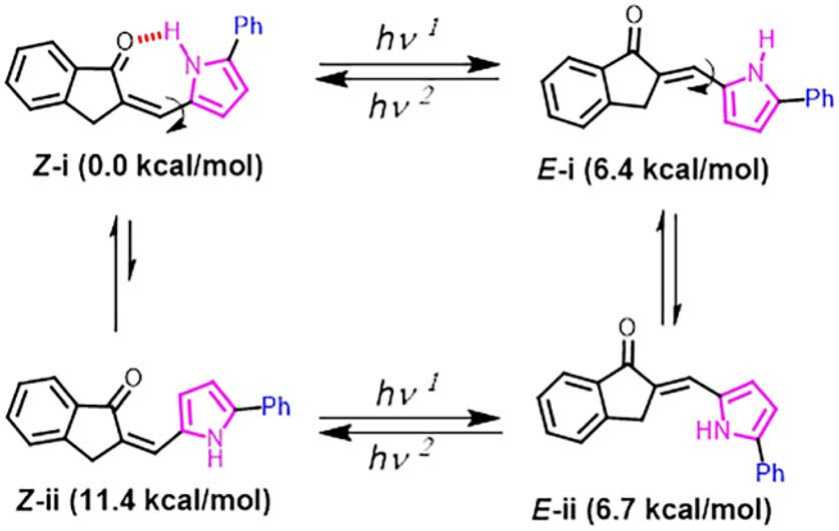
Structures of four possible photoisomers of the representative phenylpyrrolylidene-indanone switch 1.

Ideally, both *E*- and *Z*-isomers of API 1 can each have two conformers due to the rotation of the C–C bond connecting the pyrrole ring with the C=C bond, leading to the formation of four isomers: *E*-i, *E*-ii, *Z*-i, and *Z*-ii (Fig. 2). All these isomers were geometry optimised using DFT calculations at the B3LYP/6-31* level of theory, as done previously for analogous compounds.^26^ The two conformers of the *E*-isomer (*E*-i and *E*-ii) were found to be isoenergetic with only a 0.3 kcal mol^−1^ free energy difference, which is within the error limit of DFT data (Fig. S39 and Table S1). In the case of the *Z*-isomer, the *Z*-i conformer that can form an intramolecular hydrogen bond showed −11.4 kcal mol^−1^ higher stability than the *Z*-ii conformer, rendering the former one the sole populated conformer (>99.9%). From here on, the *Z*-isomer shall refer to the *Z*-i, while the *E*-isomer shall refer both *E*-i and *E*-ii.

Theoretically, the existence of the intramolecular H-bond was characterised by an O⋯H distance of 1.74 Å and a C=O⋯H angle of 113°.^27^ This theoretical prediction is in agreement with the experimental data that showed a decrease in C=O bond stretching of the *Z*-isomer by 31 nm and a highly deshielded N–{H} signal (*δ* = 14.04 ppm) in the ^1^H NMR spectrum in comparison to that of the *E*-isomer (Fig. S4). Despite the intramolecular H-bond-induced stabilisation of the *Z*-isomer, the energy difference between the lowest energy conformers of *Z*- and *E*-isomers was restricted to only 6.4 kcal mol^−1^, while azobenzene with a thermal half-life of <24 h of its cis isomer is known to have a difference of 15.2 kcal mol^−1^ energy between *E*- and *Z*-isomers.^28^ The reduced driving force of API 1 is indicative of a high activation energy barrier for the thermal *E-Z* and *Z-E* isomerisations.

**Fig. 3:**
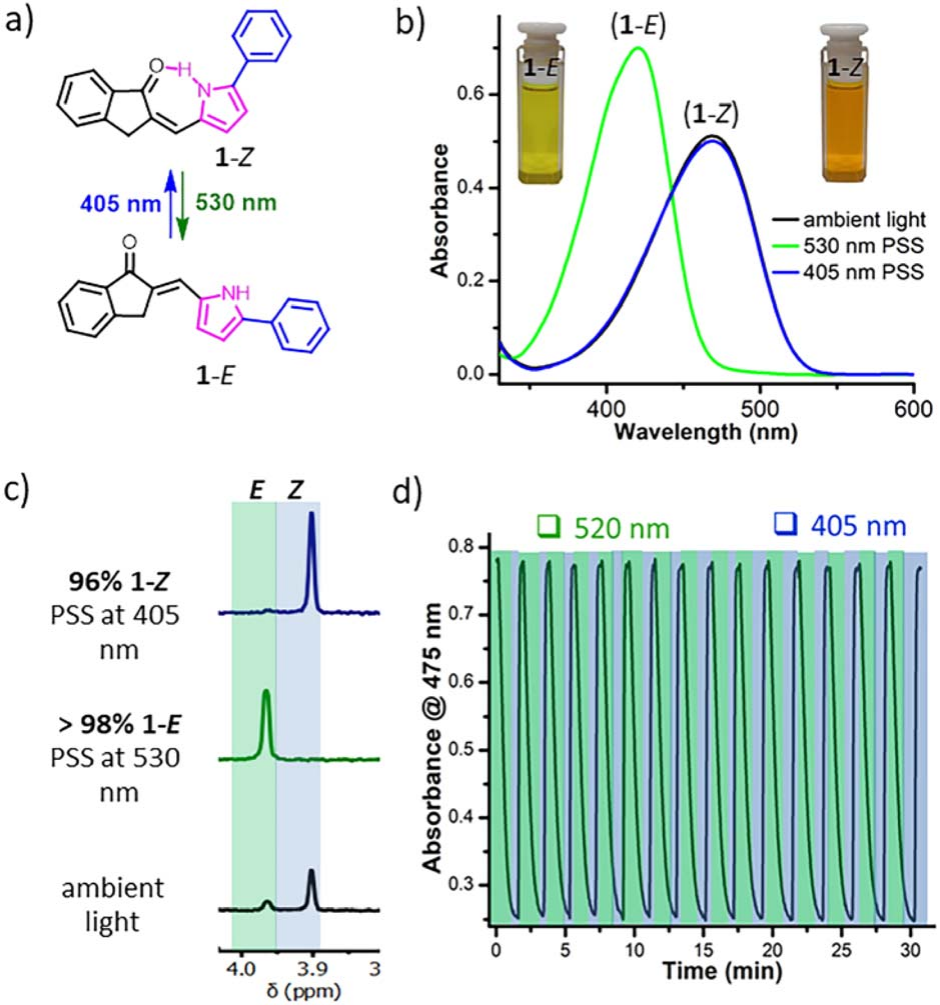
Photoisomerisation of API 1. (a) Structures of *E*-and *Z*-photoisomers of 1. (b) UV-vis absorption spectra of 1 (15 μM) in dichloromethane at 25 °C. (c) ^1^H NMR spectra of PSSs of 1 (0.3 mM) in CDCl_3_ solvent upon exposure to 405 nm light for 2 min (*E* → *Z*) and 530 nm light for 10 min (*Z* → *E*). (d) Photo-fatigue resistance of 1 (25 μM) in dichloromethane over 15 photoisomerisation cycles using alternating irradiations under 520 nm and 405 nm light at 25 °C.

The UV-vis absorption spectrum of the *Z*-isomer of 1 in dichloromethane showed an intense π–π* absorption band in the visible region (*λ*_max_ = 469 nm; *ε* = 24 478 mol^−1^ cm^−1^) with a tail expanded beyond 530 nm (Fig. 3b). Irradiation with visible light of *λ* > 500 nm led to a noticeable colour change from orange to yellow followed by a gradual disappearance of the absorption band of the *Z*-isomer with the concomitant appearance of a new band in the violet region of the spectrum (*λ*_max_ = 421 nm). The photostationary state (PSS) of *Z* → *E* isomerisation induced by 530 nm light ((15 nm full width at half maximum (fwhm)) contained >98% *E*-isomer, as shown by the ^1^H NMR spectrum in CDCl_3_ (Fig. 3c). When the *E*-isomer was exposed to visible light at 405 nm (fwhm = 9 nm), about 96% of the *Z*-isomer was formed again, and this increased to 98% when UV light at 365 nm (fwhm = 8 nm) was used (Table 1). The theoretical absorption spectra obtained by time-dependent DFT calculations resembled the experimentally acquired spectra (Fig. S43). The absorption bands are characterised by π–π* character, arising out of HOMO–LUMO electronic transitions. The longer wavelength absorption of the *Z*-isomer than that of the *E*-isomer is contrary to the absorption spectral profile of stilbene, in which *trans*-stilbene (*ca.* 300 nm) absorbs at longer wavelengths than *cis*-stilbene (*ca.* 280 nm).^29^ In comparison to the HOMO–LUMO energies of 1-*E*, the 1-*Z* showed increased HOMO and lowered LUMO energies, resulting in a considerable decrease in the HOMO–LUMO energy gap, which can be attributed to its rigid planar geometry accessible by the intramolecular H-bond. Despite undergoing multiple *E* → *Z* isomerisation cycles using visible light, noticeable degradation was not found, assuring the endurance of API switches (Fig. 3d). Noticeably, after keeping the metastable *E*-isomer in toluene solvent at 80 °C for 2 days in the dark, negligible thermal reversion into the stable *Z*-isomer was noticed (Fig. S19), characterising it as a p-type switch that permits reverse isomerisation only by light, whereas an analogous hemithioindigo switch showed a thermal half-life of 9.3 h at 80 ^o^C in toluene.^30^ The isomerisation quantum yields were estimated to be 0.05 for *Z* → *E* conversion at 520 nm and 0.04 for *E* → *Z* conversion at 385 nm (see Table 1). These values are substantially less than those of the azo-based photochromes^31^ and the analogous hemithioindigo switches,^32^ but are fairly comparable to those of spiropyrans (*Φ*_f_ = 0.03).^33^ The less efficient photoisomerisation of 1 would mean that this push–pull API may emit fluorescence like the spiropyrans. Notably, both *E*- and *Z*-isomers were found to be emissive, as proposed. The *Z*-isomer of 1 emitted green fluorescence (*λ*^max^_em_ = 537 nm) with electronic excitation at *λ*_ex_ = 470 nm, while its *E*-isomer fluoresced cyan colour (*λ*^max^_em_ = 489 nm) upon excitation at 420 nm in dichloromethane. The emission quantum yields were estimated to be 0.25 and 0.18 for *Z*- and *E*-isomers, respectively. Around ten rounds of *Z*/*E* isomerisation cycles did not incur any noticeable damage to the fluorescence efficiencies of both isomers (Fig. S34).

**Table 1:** Photophysical and photochemical properties of series I APIs in dichloromethane

API	*λ*_max_/nm (*Z*)	*λ*_max_/nm (*E*)	PSS composition (irradiation wavelength)	*ε*/L mol^−1^ cm^−1^	*Φ*_*Z*→*E*_	*Φ*_*E*→*Z*_	*λ* ^max^_em_/nm (*Z*)	*λ* ^max^_em_ /nm (*E*)	*Φ*_f_ (*Z*)	*Φ*_f_ (*E*)
1	469	421	*E*: >98% (530 nm)	32 314 (*E*)	0.05	0.04	537	489	0.25	0.18
			*Z*: >98% (365 nm), 96% (405 nm)	24 478 (*Z*)						
2	438	423	*E*: >98% (505 nm)	—a	—a	—a	—a	486	—b	0.07
			Z: 68% (385 nm)	—a						
3	419	379	*E*: >98% (475 nm)	22 294 (*E*)	—a	—a	—a	451	—b	0.03
			*Z*: 90% (365 nm)	20 247 (*Z*)						

a Not performed.

b Non-fluorescent.

To figure out the structural basis of fluorescence emission, we designed compounds 2–3 in series I (Chart 1), in which the photoisomerisation of 3 had been studied before.^25^ Compound 3 was readily synthesised by the aldol condensation reaction of 1-indanone with 2-formylpyrrole (Scheme 1). Methylation of the pyrrole NH of either the *E*- or *Z*-isomer of 1 with MeI/NaH at ambient temperature resulted in the formation of mostly the *E*-isomer of API 2. The disappearance of the 2-*Z* isomer in the final product can be accounted for by its inability to form the intramolecular H-bond, which indirectly supports the necessity of the intramolecular H-bond for stabilising the *Z*-isomer of API 1. Before measuring the fluorescence emission, we (re)investigated the isomerisation efficiencies of 2–3 under our experimental photoirradiation conditions. Noticeably, both compounds could produce quantitative *Z* → *E* isomerisation (>98%) by irradiation with visible light of *λ* = 505 nm (fwhm = 17 nm), and 475 nm (fwhm = 11 nm), respectively (Table 1, Fig. S5 and S6), whereas the reverse *E* → *Z* conversion led to only 68% 2-*Z* upon irradiation with 385 nm light (fwhm = 10 nm) and 90% 3-*Z* isomers upon irradiation with 365 nm light (fwhm = 8 nm). It is worthwhile to mention here that the API 3 was previously reported to induce 40% and 76% *E* → *Z* conversion in DMSO-*d*_6_ and CD_3_CN, respectively, after irradiation with 365 nm light for 45 min.^25^ The inappropriate selection of the irradiation wavelength can be accounted for by these poor photoconversion yields. Importantly, *E*-isomers of 2–3 exhibited weak fluorescence (*Φ*_f_ = 0.07 for 2 and 0.03 for 3) while their *Z*-isomers were non-fluorescent. Calculations revealed that the 2-*Z* has a non-planar geometry, while the 3-*Z* has a planar rigid geometry due to the presence of an intramolecular C=O⋯H bond, similar to that in the 1-*Z*. The flexible 2-*Z* geometry, which lacks an intramolecular H-bond, has experienced fluorescence quenching, indicating that the presence of the intramolecular H-bond is essential for conferring fluorescence emission in the *Z*-isomer. In contrast, despite having a planar rigid conformation of 3-*Z* aided by the intramolecular H-bond, it is non-emissive, unlike 1-*Z* that has a phenyl substitution in the pyrrole ring, indicating the utility of extended π-conjugation for achieving fluorescent emission. Thus, these structure–property-relationship analyses ascertained that a pyrrole ring with a free NH, which can form an intramolecular C=O⋯H bond, and an aryl ring attached to the pyrrole moiety are crucial to induce emissive properties in the *Z*-isomers of the API class of switches.

To tune the photoswitching and fluorescence emission properties of 1, we introduced electroactive substituents such as OMe, CN, or CO_2_Et on the phenyl ring at the *para*- (or *ortho*) position with respect to the pyrrole ring position, which led to the design of series II APIs 4–8, as shown in Table 2. These newly designed compounds 4–8 were readily prepared starting from commercially available starting materials—1-indanone, pyrrole, and appropriate aryl bromides—in the six-step reaction sequence as employed for the preparation of the API 1 (Scheme 1).

**Table 2:** Photophysical and photochemical properties of series II APIs in dichloromethane^a^

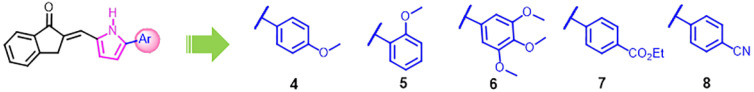										
API	*λ*_max_/nm (*Z*)	*λ*_max_/nm (*E*)	PSS composition (irradiation wavelength)	*ε*/L mol^−1^ cm^−1^	*Φ*_*Z*→*E*_	*Φ*_*E*→*Z*_	*λ* ^max^_em_/nm (*Z*)	*λ* ^max^_em_ /nm (*E*)	*Φ*_f_ (*Z*)	*Φ*_f_ (*E*)
4	485	431	*E*: >98% (550 nm)	28 032 (*E*)	0.03	0.03	557	515	0.48^b^	0.22^b^
			*Z*: 97% (405 nm), >98% (365 nm)	23 257 (*Z*)						
5	474	435	*E*: 98% (530 nm)	36 502 (*E*)	0.06	0.04	546	505	0.20	0.29
			*Z*: 92% (405 nm), 93% (365 nm)	26 336 (*Z*)						
6	478	426	*E*: 87% (520 nm)	29 886 (*E*)	0.02	0.02	560	520	0.38	0.21
			*Z*: 94% (405 nm), 95% (385 nm)	22 857 (*Z*)						
7	467	423	*E*: >98% (520 nm)	48 390 (*E*)	0.04	0.03	534	484	0.20	0.19
			*Z*: >96% (405 nm), >98% (385 nm)	34 298 (*Z*)						
8	463	420	*E*: 98% (520 nm)	56 494 (*E*)	0.03	0.03	531	475	0.16	0.15
			*Z*: 98% (405 nm), 98% (365 nm)	38 505 (*Z*)						

a No thermal *E*→*Z* isomerisation was seen for APIs 1 and 4–6 after heating at 80 °C for 12–48 hours.

b In dioxane, *Φ*_f_ = 0.50 (*Z*) and 0.42 (*E*) and in chloroform, *Φ*_f_ = 0.47 (*Z*) and 0.27 (*E*).

As usually observed in *E*/*Z* switches with a group that donates electrons, the π–π* bands of both *Z*- and *E*-isomers of 4 shifted to longer wavelengths by 16 nm and 10 nm, respectively, compared to the parent switch 1. This is in agreement with the DFT calculations, which indicated that in comparison to the energy levels of FMOs of the unsubstituted compound 1, the HOMOs of both *E*- and *Z*-isomers of compounds carrying electron-donating groups, such as *p*-OMe in 4, are destabilised, while their LUMOs get stabilised to varying extents, resulting in the reduced π–π* excitation energies for both isomers (Fig. S42). Since both *Z*- and *E*-isomers experienced bathochromic shifts, the overall splitting of their π–π* bands increased marginally; however, the red-shifted absorption allowed us to use green light (550 nm, fwhm = 18 nm) for the selective excitation of the *Z*-isomer to obtain PSS with >98% *E*-isomer and to use violet light of *λ* = 405 nm (fwhm = 9 nm) for back irradiation to produce PSS with 97% *Z*-isomer. The *E* → *Z* photoconversion yield increased to >98% when UV light of *λ* = 365 nm (fwhm = 8 nm) was applied. For the compound 5 with an *ortho*-methoxy group, even though the separation of the π–π* bands for the two isomers was much smaller, the photoconversion yields in both directions remained (near) quantitative, since the photoirradiations were carried out in the tail regions (530 nm for *Z* → *E* and 405 nm for *E* → *Z* conversion) of the spectra where one isomer absorbs to a greater extent than the other isomer. In the case of the highly electron-rich API 6 containing three OMe substituents at *para*- and *meta*-positions of the phenyl ring, the *Z* → *E* isomerisation yield was reduced to 87%. Like 1-*E*, the *E*-isomers of switches 4–6 were exceptionally thermally stable. Heating them at 80 °C in toluene for 12 h could not induce considerable *E* → *Z* isomerisation, endowing them with P-type character like the parent API 1 (Fig. S19). Noticeably, these electron-rich switches 4–6 demonstrated better fluorescent properties than the parent unsubstituted compound 1. Both *E*- and *Z*-isomers experienced substantial Stokes shifts (70–94 nm), resulting in bathochromic shifts of their emission maxima in a range between 40 and 42 nm compared to that of 1. Most importantly, the fluorescence quantum yields of both isomers of 4–6, except 5-*Z* that holds an *ortho*-OMe-substituent, were increased substantially in contrast to those of the parent compound 1 (Table 2). The *Z*-isomer of 4, which has a *para*-OMe substituent, showed the strongest emission intensity (*Φ*_f_ = 0.48) in the API series, while its *E*-isomer had a much lower intensity (*Φ*_f_ = 0.22). The API 6 with the highest electron-rich phenyl ring exhibited slightly reduced emission intensity of the *Z*-isomer (*Φ*_f_ = 0.38) in addition to the comparatively low *Z* → *E* photoconversion.

The switches carrying electron-withdrawing substituents such as CO_2_Et (7) or CN (8) at the *para*-position did not have much effect on the absorption wavelengths of both isomers relative to those of the parent API 1. The HOMOs and LUMOs of both isomers are stabilised to the extent that π–π*excitation energies have remained the same as those of the parent compound 1 (Fig. S42). However, their absorption coefficients were found to be increased considerably (1.4–1.7 times). Both 7 and 8 demonstrated quantitative photoisomerisation (>98%) in both directions using 520 nm light (fwhm = 15 nm) for *Z* → *E* and 385 nm/365 nm light for *E* → *Z* conversions. The fluorescence quantum yields of these electron-deficient APIs were slightly reduced from those of the parent compound 1; switch 8 with the *p*-CN substituent that is known to have a stronger electron-withdrawing effect than the CO_2_Et substituent (*σ* = 0.66 for CN and 0.45 for ester)^34^ showed the lowest fluorescence quantum yields (*Φ*_f_ = 0.16 (*Z*) and 0.15 (*E*)) in the series. Nonetheless, these values are substantially greater than those of the recently reported push–pull hydrazone switch in the solution state.^20^

In general, the emission intensities of *E*-isomers of APIs, except 5, are less than those of their *Z*-isomers. The decrease in fluorescence emission of a monolithic photoswitchable fluorophore occurs when its electronically excited state quickly loses energy through non-radiative pathways, which usually involve isomerisation and/or kinetic motions of certain parts of the molecule. Since the C=C photoisomerisation quantum yields of both *E*- and *Z*-isomers of our APIs are nearly equal, the isomerisation path cannot account for the variation of fluorescence intensities of the two isomers, while the difference in kinetic movements of the two isomers due to the presence of the intramolecular H-bond only in the *Z*-isomer is likely to be responsible. Such a –C=O⋯H–N bond could inhibit the free rotation of the C–C bond between the pyrrole ring and the C=C unit in the *Z*-isomer and thereby rigidify its conformation, resulting in the enhancement of fluorescence emission *via* suppression of the rotation-mediated energy loss. In the *E*-isomer, however, the C–C bond rotation would not be restricted due to the absence of the –C=O⋯H–N bond, leading to a flexible conformation that could assist in deactivation of the excited state *via* rotation-mediated energy loss, therefore suppressing the fluorescence emission. These postulations are following the DFT calculations, which showed a significantly low rotational energy barrier for the C–C bond (between pyrrole and alkene) in the *E*-isomer of 4 than in the *Z*-isomer in the ground state (S^0^) (21.5 kcal mol^−1^ for *Z vs.* 10.1 kcal mol^−1^ for *E*) as well as in the first excited state (S^1^) (−0.8 kcal mol^−1^ for *Z vs.* −9.5 kcal mol^−1^ for *E*) (Fig. S44 and Table S1).

**Fig. 4:**
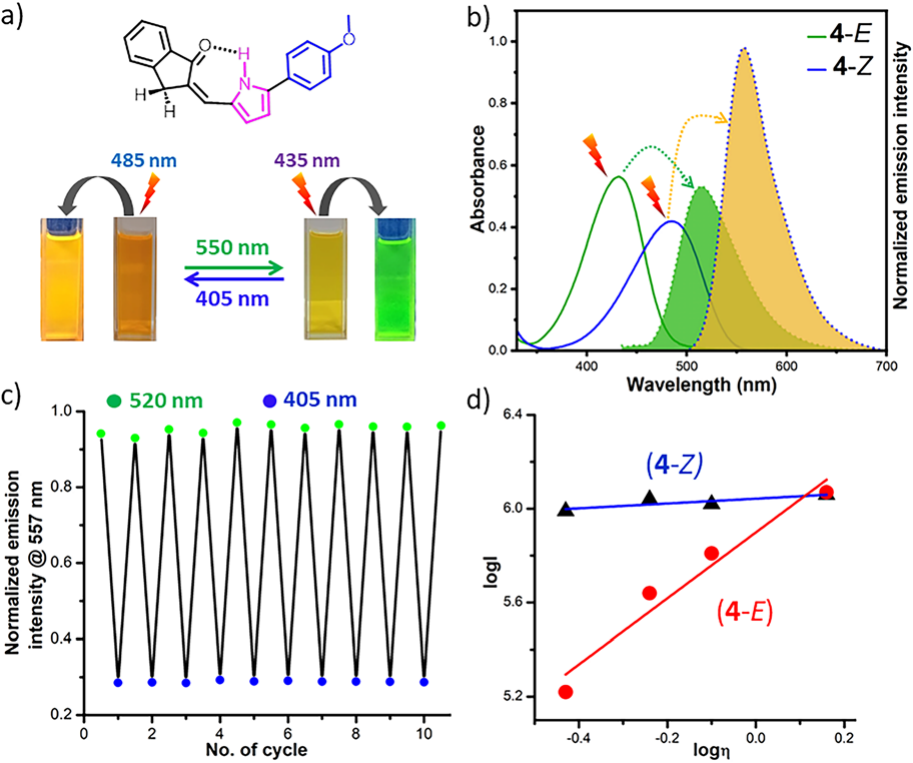
(a) Visually assessed absorption and fluorescence emission colours of *E*- and *Z*-isomers of representative API 4 in dichloromethane in a cuvette. (b) UV-vis adsorption spectra (blue solid line for the *Z*-isomer and green solid line for the *E*-isomer) and fluorescence emission spectra (yellow shaded region for the *Z*-isomer and green shaded region for the *E*-isomer) upon excitation at 485 nm for *Z* and 435 nm for *E* of representative API 4 (10 μM) in dichloromethane at 25 °C. (c) Multiple photoswitching cycles by alternating green (520 nm) and violet (405 nm) light irradiation followed with concomitant acquisition of fluorescence emission at 557 nm for both 4-*E* and 4-*Z* isomers with excitation at 485 nm and 435 nm, respectively, under aerobic conditions, indicating high phtofatigue resistance. (d) Plots of emission intensity (log *I*) of 10 μM solutions of 4-*Z* (solid triangle) and 4-*E* (solid circle) as a function of solvent viscosity (log *η*).

To experimentally investigate whether the C–C bond rotation indeed plays a role in the fluorescence emission, we measured emissions of both isomers of the representative compound 4 in organic solvents of different viscosities, such as acetonitrile (0.37 cP), chloroform (0.58 cP), 1,2-dichloroethane (0.79 cP), and 1,4-dioxane (1.44 cP). The plots of the fluorescence emissions against solvent viscosity are depicted in Fig. 4d. It shows only a subtle increase in the fluorescence intensity of the *Z*-isomer with the increase in solvent viscosity and a sharp increase in intensity in the case of the *E*-isomer. In going from the low-viscosity acetonitrile to the high-viscosity 1,4-dioxane solvent, the 4-*E* isomer presented a nearly 7-fold fluorescence intensity enhancement, which is attributed to the acquired rigid conformation due to the inhibition of the free rotation of the C–C bond in the viscous medium, facilitating radiative emission. Such rigidification of the conformation of the 4-*Z* happens intrinsically due to the occurrence of the intramolecular H-bond between the pyrrole NH and the indanone C=O, irrespective of the solvent viscosity. As a result, the fluorescence intensity of the *Z*-isomer remained independent of solvent viscosity. In dioxane solvent, we estimated the highest fluorescence quantum yields of 4-*Z* (*Φ*_f_ = 0.50 *vs.* 0.48 in DCM and 0.47 in chloroform) and 4-*E* (*Φ*_f_ = 0.42 *vs.* 0.22 in DCM and 0.27 in chloroform) (Table 2). These experimental findings suggested that the emission of the *E*-isomer can be intrinsically suppressed by the acceleration of the C–C bond rotation between pyrrole and C=C units.

**Scheme 2:**
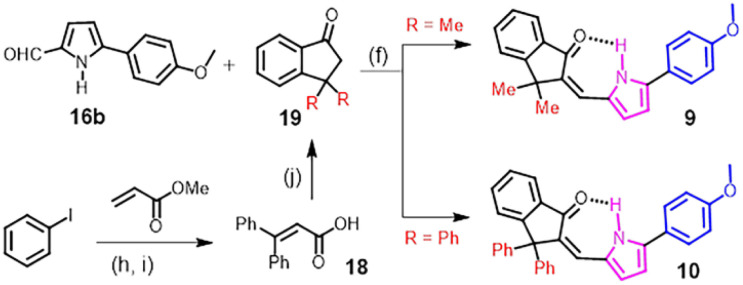
Structures of series III arylpyrrolylidene-indanones and their synthesis. Reagents and conditions: (h) Pd(OAc)_2_, AgOAc, AcOH, 110 °C, 6 h; (i) LiOH·H_2_O, THF/MeOH/H2O (3 : 1:1), rt, 3 h; (j) benzene, TfOH, DCE, reflux, 12 h.

**Fig. 5:**
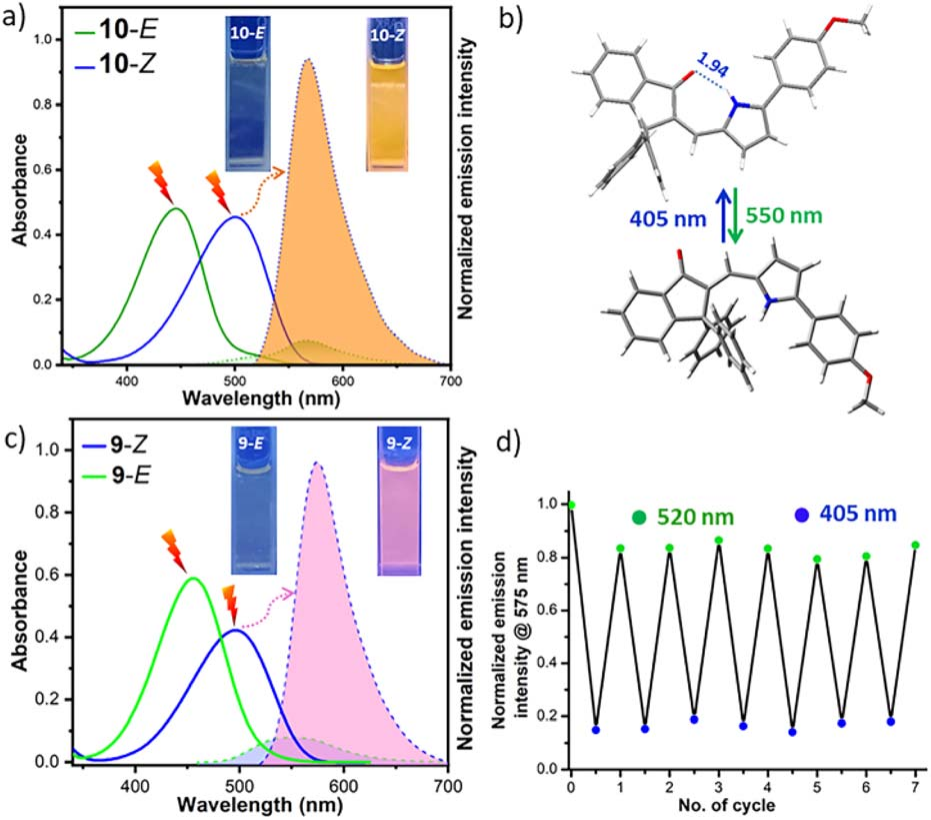
(a) UV-vis adsorption spectra (blue solid line for the *Z*-isomer and green solid line for the *E*-isomer) and fluorescence emission spectra (orange shade for *Z*, *λ*_ex_ = 500 nm and green shade for *E*, *λ*_ex_ = 450 nm) of both isomers of 10 (10 μM) in dichloromethane at 25 °C. (b) Photoisomers of representative API 10: *Z* (X-ray crystal structure) and *E* (geometry optimised structure). (c) UV-vis adsorption spectra (blue solid line for *Z* and green solid line for *E*) and fluorescence emission spectra (pink shade for *Z*, *λ*_ex_ = 495 nm and grey shade for *E*, *λ*_ex_ = 455 nm) of both isomers of 9 (15 μM) in 50% DMSO-PBS buffer (pH = 7.4) at 25 °C. (d) Multiple fluorescence photoswitching cycles of the representative API 9 (15 μM) in 50% DMSO-PBS buffer under aerobic conditions at 25 °C.

In order to design (on/off) single-colour fluorescent photoswitches *via* acceleration of the C–C bond rotation of the API 4, we envisaged the introduction of bulky substituents, such as methyl or phenyl groups, at the sp^3^-C near the C–C bond, which led to the design of compounds 9–10 of the API series III (Scheme 2). Theoretical calculations revealed *ca.* 2.5 kcal mol^−1^ and *ca.* 2.1 kcal mol^−1^ lower energy barriers of the C–C bond rotation of the 9-*E* in the S^0^ and S^1^ states, respectively, in comparison to those of the parent compound 4 (Fig. S44 and Table S1), which supported our assumption. Compounds 9 and 10 were readily prepared by the aldol condensation reaction of previously prepared 5-(4-methoxyphenyl)-2-formylpyrrole with 3,3-dimethyl-1-indanone (commercially available) and 3,3-diphenyl-1-indanone, respectively (Scheme 2). The latter indanone building block was prepared from methyl acrylate *via* a double Heck coupling and an ester hydrolysis reaction sequence, followed by a final intramolecular Friedel–Crafts acylation in the presence of benzene. The X-ray crystal structure of the *Z*-isomer of 10 (Fig. 5b), obtained by the slow evaporation of its solution in a 1 : 1 acetonitrile and DCM solvent mixture, was found to be comparable to the calculated structure, showing an intramolecular H-bond distance of 1.94 Å and a C=O angle of 115.1° against the computed values of 1.74 Å and 113.6° (Fig. S39), respectively. This constituted direct evidence in support of the existence of an intramolecular H-bond between pyrrole NH and indanone C=O. The UV-vis absorption spectral profiles of 9–10 acquired in dichloromethane are similar to that of the parent switch 4. In the case of the diphenyl-substituted API 10, the absorption maxima of both isomers showed bathochromic shifts of *ca.* 16 nm compared to those of 4. The *Z* → *E* isomerisation using green light at *λ*_max_ = 550 nm produced >98% 9-*E* and 95% 10-*E*, while the *E* → *Z* isomerisation using violet light (405 nm) regenerated 98% *Z*-isomers for both compounds (Table 3). Although this structural modification did not compromise the photoisomerisation yields, it reduced the thermal half-lives. The *t*_1/2_ of 35 h and 26 h at 80 °C in toluene were measured for *E*-isomers of 9 and 10, respectively (Table 3 and Fig. S19). These half-lives are significantly higher than that of the analogous hemithioindigo switch with a *t*_1/2_ of 4.5 h at 80 °C in toluene.^30^ Calculation revealed a free energy difference of *ca.* 10–11 kcal mol^−1^ between the *E*- and *Z*-isomers of 9 and 10 (Fig. S39), which is substantially higher than that of the API 4 (∼7 kcal mol^−1^). The enhanced driving force of the modified APIs 9 and 10, as revealed by the higher free energy difference between *E* and *Z* isomers, is indicative of a lower activation energy barrier for thermal *E* → *Z* isomerisation. To determine the free energy of activations, we performed transition state (TS) calculations for the thermal *E* → *Z* isomerisation of APIs 4, 9, and 10 at the B3LYP 6-31G* level of theory, and the results are shown in Table S2 and Fig. S45. Compared to 4, APIs 9 and 10, which carry two bulky groups at the sp^3^-C, indeed experienced a significant reduction in the activation energies (*ca.* 9–11 kcal mol^−1^) to result in the relatively rapid thermal *E* → *Z* isomerisation.

**Table 3:** Photophysical, photoswitching and thermal half-lives of APIs 9 and 10

API	Solvent	*λ*_max_/nm	*λ*_max_/nm	*E*% of PSS at 550 nm *hν*	*Φ*_*Z*→*E*_	*Z*% of PSS at 405 nm *hν*	*Φ*_*E*→*Z*_	*λ* ^max^_em_/nm (*Z*)	*Φ*_f_ (*Z*)	*Φ*_f_ (*E*)	*t*_1/2_ of *E* at 80 °C^a^
9	DCM	485 (*Z*)	429 (*E*)	>98%	0.01	98%	0.02	562	0.40	0.02	35 h
	DMSO	491 (*Z*)	445 (*E*)	>98%	—b	81%	—b	567	0.39	0.03	—b
	DMSO-aq. buffer	496 (*Z*)	457 (*E*)	98%	—b	79%	—b	574	0.25	0.02	—b
10	DCM	501 (*Z*)	445 (*E*)	95%	0.008	>98%	0.04	568	0.45	0.006	26 h

a Half-lives were measured in toluene.

b Not performed.

The fluorescence emissions measured in dichloromethane are shown in Fig. 5a and S33. The 9-*Z* and 10-*Z* were highly emissive like the *Z*-isomer of 4, showing yellow fluorescence emission at *λ*^max^_em_ = 562 nm with *Φ*_f_ = 0.40 and at *λ*^max^_em_ = 568 nm with *Φ*_f_ = 0.45, respectively, whereas their *E*-isomers showed very low to negligible quantum yields (*Φ*_f_ = 0.02 for 9-*E* and 0.006 for 10-*E*). The latter API, in particular, can be treated as a single-colour fluorescence switch (on/off). Noticeable fading of fluorescence emission intensity was not seen for both compounds after multiple rounds of fluorescence excitations followed by *E*/*Z* photoisomeisation (Fig. S35 and S36).

We then determined the photoisomerisation quantum yields (Table 3) and measured the fluorescence emissions of these APIs with the variation of solvent viscosity (Fig. S38). Concerning the emissive 4-*E*, *E*-isomers of 9 and 10 produced a marginal difference in the *E* → *Z* photoisomerisation quantum yields but demonstrated more pronounced viscosity-dependent fluorescence intensity, as evident by the higher slope of the emission *vs.* solvent viscosity plot (slope: 1.58 for 9*vs.* slope: 1.4 for 4, see Fig. S37 and S38). This implies that the flexibility in the conformation of the *E*-isomer due to the free rotation of the C–C bond between pyrrole and alkene moieties should be responsible for the solvent-dependent fluorescence emission of 9-*E* and 10-*E*, like that of 4-*E*. The low-viscosity dichloromethane solvent expedites the C–C bond rotation, enabling deactivation of the electronically excited state *via* rotation-mediated non-radiative heat release to become weakly fluorescent or non-fluorescent, whereas the highly viscous 1,4-dioxane solvent restricts the C–C bond rotation such that the conformation of the *E*-isomer becomes rigid, therefore promoting radiative emission indirectly. The *E*-isomers of APIs 9, 10, and 4 may therefore also serve as viscosity sensors.

Since these visible-light-responsive single- or dual-colour fluorescent photoswitches may possess applications in the super-resolution fluorescence imaging of biological matters, we perform fluorescence photoswitching essentially in aqueous media. It is worth noting that several fluorescent photoswitches based on DAE^35^ and spiropyrans^36^ lose their photoswitching performance and fluorescence emission in aqueous solvents because they tend to degrade or aggregate. In 50% DMSO-PBS buffer (pH = 7.4), our representative API 9 retained the photoisomerisation and fluorescence emission without compromising their efficiencies substantially (Table 3 and Fig. 5c). The emission quantum yield of 9-*Z* (*Φ*_f_ = 0.25) was reduced by 1.6 times compared to those estimated in DMSO and DCM solvents, but its *E*-isomer did not see considerable variation in *Φ*_f_ (Table 3). While the *Z* → *E* photoisomerisation remained unchanged irrespective of the solvent, the *E* → *Z* photoconversion yield in the 50% DMSO-PBS buffer was found to be reduced by *ca.* 2% compared to those measured in pure DMSO (Fig. S14). Development of fully water-soluble APIs for the super-resolution fluorescence imaging of biological samples is needed.

## Conclusions

We designed and readily synthesised a series of new arylpyrrolylidene-indanones. APIs 1 and 4-8 with the free N–H of the pyrrole ring showed high to (near) quantitative photoisomerisation under visible light in both directions (520–550 nm for *Z* → *E* and 405 nm for *E* → *Z* conversions). The presence of an intramolecular H-bond between pyrrole NH and indanone C=O in the *Z*-isomer (typically metastable) not only transformed it into a more stable isomer than the *E*-isomer, such that these compounds behave as P-type photoswitches at ambient temperature, but also enhanced the fluorescence emission of the *Z*-isomer. Notably, both isomers of an arylpyrrolylidene-indanone that contains a *para* electron-donating substituent, such as compound 4, were found to be highly fluorescent—the *Z*-isomer emitted orange light with *Φ*_f_ = 0.48–0.50, *λ*_max_ = 557 nm, and the *E*-isomer fluoresced green, having *Φ*_f_ = 0.22–0.42, *λ*_max_ = 515 nm. The higher fluorescence intensity of the *Z*-isomer in comparison to the *E*-isomer was ascribed to its rigid conformation earned by the intramolecular H-bond, which was evident by the solvent viscosity-dependent and viscosity-independent fluorescence emissions of the *E*- and *Z*-isomers of 4, respectively. Expediting the rotation of the C–C bond between pyrrole and C=C upon introduction of dimethyl or diphenyl substitution at the sp^3^-C adjacent to the C=C bond led to (near) complete elimination of the fluorescence emission of the *E*-isomers, rendering these compounds (on/off) single-colour fluorescence photoswitches. The fluorescence photoswitching of the representative API 9 was found to be preserved in 50% DMSO-PBS buffer (pH = 7.4) with a slight reduction in the photoisomerisation and emission efficiencies. We anticipate that these dual- and single-colour fluorescence-switching molecular constructs will find numerous applications in both life and materials science.

## Author contributions

S. Samanta designed the research, performed DFT calculations, and wrote the manuscript. S. Bera conducted most of the syntheses, photophysical, and photoswitching experiments, analysed X-ray crystal structure, and wrote the initial draft of the Ms S. Bhunia and A. Dolai performed photoswitching experiments in the NMR tubes. A. Das and Sk M. Box made few APIs and carried out some of the photoisomerization and thermal isomerisation studies. All authors discussed the results and reviewed the manuscript.

## Conflicts of interest

The authors declare no conflict of interest

## Supplementary Material

SC-016-D5SC04551G-s001

SC-016-D5SC04551G-s002

## Data Availability

The data supporting this article have been included as part of the supplementary information. It includes experimental procedures, characterization data of the compounds (NMR, MS, IR, UV-vis, emission spectra, and single-crystal data) and computational data. See DOI: https://doi.org/10.1039/d5sc04551g. CCDC 2450471 contains the supplementary crystallographic data for this paper.^37^

## References

[cit1] Russew M. M., Hecht S. (2010). Adv. Mater..

[cit2] Balzani V., Credi A., Venturi M. (2009). Chem. Soc. Rev..

[cit3] Le M., Han G. G. D. (2022). Acc. Mater. Res..

[cit4] Kawata S., Kawata Y. (2000). Chem. Rev..

[cit5] Beharry A. A., Wong L., Tropepe V., Woolley G. A. (2011). Angew. Chem., Int. Ed..

[cit6] Chozinski T. J., Gagnon L. A., Vaughan J. C. (2014). FEBS Lett..

[cit7] Fukaminato T. (2011). J. Photochem. Photobiol., C.

[cit8] Kim D., Park S. Y. (2018). Adv. Opt. Mater..

[cit9] Putri R. M., Fredy J. W., Cornelissen J. J. L. M., Koay M. S. T., Katsonis N. (2016). ChemPhysChem.

[cit10] Mo S., Meng Q., Wan S., Su Z., Yan H., Tang B. Z., Yin M. (2017). Adv. Funct. Mater..

[cit11] Fu Y. X., Han H. H., Zhang J. J., He X. P., Feringa B. L., Tian H. (2018). J. Am. Chem. Soc..

[cit12] Chen J., Zhong W., Xue M., Wang H., Yu M., Zhang P., Yi P. (2017). Polym. Chem..

[cit13] Fukaminato T., Ishida S., Métivier R. (2018). NPG Asia Mater..

[cit14] Soh N., Yoshida K., Nakajima H., Nakano K., Imato T., Fukaminato T., Irie M. A. (2007). Chem. Commun..

[cit15] Cheong W.-F., Prahl S. A., Welch A. J. (1990). IEEE J. Quantum Electron.

[cit16] Herder M., Schmidt B. M., Grubert L., Pa’tzel M., Schwarz J., Hecht S. (2015). J. Am. Chem. Soc..

[cit17] Kim D., Aktalay A., Jensen N., Uno K., Bossi M. L., Belov V. N., Hell S. W. (2022). J. Am. Chem. Soc..

[cit18] Rau H. (1973). Angew. Chem., Int. Ed..

[cit19] Saltiel J., Waller A. S., Sears D. F., Garrett C. Z. (1993). J. Phys. Chem..

[cit20] Shao B., Baroncini M., Qian H., Bussotti L., Di Donato M., Credi A., Aprahamian I. (2018). J. Am. Chem. Soc..

[cit21] Llamosí A., Olejnik M. A., Danylyuk O., Rode M. F., Szumna A. (2025). Chem.–Eur. J..

[cit22] Li J., Ma X., Wang Y., Cheng Y., Qin Y., Zhai J., Xie X. (2023). Anal. Chem..

[cit23] Berdnikova D. V., Steup S., Bolte M., Suta M. (2023). Chem.–Eur. J..

[cit24] Han Z., He M., Wang G., Lehn J.-M., Li Q. (2024). Angew. Chem., Int. Ed..

[cit25] Li J., Zheng H., Lu H., Li J., Yao L., Wang Y., Zhou X., Nie J., Zhu X., Fu Z. (2022). Eur. Polym. J..

[cit26] Zweig J. E., Ko T. A., Huang J., Newhouse T. R. (2019). Tetrahedron.

[cit27] Etter M. C. (1990). Acc. Chem. Res..

[cit28] Gagliardi L., Orlandi G., Bernardi F., Cembran A., Garavelli M. (2004). Theor. Chem. Acc..

[cit29] Beale R. N., Roe E. M. F. (1953). J. Chem. Soc..

[cit30] Zweig J. E., Newhouse T. R. (2017). J. Am. Chem. Soc..

[cit31] Bandara H. M. D., Burdette S. C. (2012). Chem. Soc. Rev..

[cit32] Josef V., Hampel F., Dube H. (2022). Angew. Chem., Int. Ed..

[cit33] Kortekaas L., Chen J., Jacquemin D., Browne W. R. (2018). J. Phys. Chem. B.

[cit34] Hansch C., Leo A., Taft R. W. (1991). Chem. Rev..

[cit35] Roubinet B., Bossi M. L., Alt P., Leutenegger M., Shojaei H., Schnorrenberg S., Nizamov S., Irie M., Belov V. N., Hell S. W. (2016). Angew. Chem., Int. Ed..

[cit36] Hammarson M., Nilsson J. R., Li S., Beke-Somfai T., Andréasson J. (2013). J. Phys. Chem. B.

[cit37] Bera S. Bhunia S. , Dolai A., Sk M., Das A. and Samanta S., CCDC 2450471, Experimental Crystal Structure Determination, 2025, 10.5517/ccdc.csd.cc2n7xg9

