# Supplementary material for: Arylpyrrolylidene-indanones as readily synthesised photoswitches offering dual- and single-colour fluorescence toggling with nearly quantitative E/Z isomerisation under visible light
Source: Chem Sci. 2025 Aug 4;16(35):16196–204. doi: 10.1039/d5sc04551g (PMC12341562; doi:10.1039/d5sc04551g)
Supplement: SC-016-D5SC04551G-s001 [file SC-016-D5SC04551G-s001.pdf]

Supplementary Information (SI) for Chemical Science.
This journal is © The Royal Society of Chemistry 2025

Electronic Supplementary Information

Arylpyrrolylidene-Indanones as Readily Synthesized Photoswitches Offering Dual- and Single-Color Fluorescence Toggling with Nearly Quantitative E/Z Isomerisation under Visible Light

Satyajit Bera[a], Supriya Bhunia[a], Anirban Dolai[a], Sk Majid Box[a], Arpan Das[a] and Subhas Samanta[a]*

[a]Department of Chemistry, University of Calcutta, 92 A.P.C Road, Kolkata-700009, West Bengal, India.

Table of Contents — ***Page no.***

**1**	**Materials and methods**	S2
**2**	**Synthesis**	S2-S11
**3**	**Photoswitching studies**	S12
3.1	General method	S12-S14
3.2	UV-Vis absorption spectra of APIs **1, 3-10**	
3.3	Determination of the compositions of photostationary states using ^{1}H NMR with acquisition of corresponding UV-Vis absorption spectra	S14-S24
3.4	Photobleaching experiments	S25-S26
3.5	Determination of thermal half-lives of *E*-isomers	S27-S28
3.6	Photoisomerisation quantum yield estimation	S28-S31
**4**	**Fluorescence emission properties**	S32-S38
4.1	General method	S32
4.2	Fluorescence spectra of *E*-and *Z*-isomers	S32-S34
4.3	Fluorescence switching cycles	S35-S36
4.4	Determination of the fluorescence quantum yield	S36
4.5	Fluorescence response to solvent viscosity	S37-S38
**5**	**Computational details**	S39-S46
**6**	**Crystallography**	S47
**7**	**NMR spectroscopy**	S48-S80
**8**	**Cartesian coordinates**	S81-S88
**9**	**References**	S88

1. Materials and Methods:

Reagents were bought from commercially available Sigma Aldrich, TCI, Avra, BLD pharm, Spectrochem and used without further purification. Solvents were distilled for the use of column chromatography. Anhydrous condition reactions were conducted in oven-dried glassware under argon atmosphere. UV-Vis spectroscopic and photoswitching experiments were done by the use of HPLC grade solvent (Chloroform). Silica gel 100-200 mesh size was used for purification of compounds by column chromatography. Progress of reactions was checked by thin-layer chromatography (TLC) using Merck silica gel 60 F254 plates (0.25 mm). Solvent evaporation was done by rotary evaporator and the compounds further dried using high vacuum pump. For TLC plate visualization, UV chamber (λ = 254 nm or 365 nm) was used. UV-Vis absorption spectra before and after irradiation and kinetics experiments were performed on a HITACHI UH4150 spectrophotometer or SHIMADZU UV-1900 UV-Vis spectrophotometer. Steady state emission spectra were recorded on Horiba Fluoromax-Plus-c fluorimeter. ^{1}H and ^{13}C NMR were recorded on a Bruker Avance-III 300 MHz NMR spectrometer at 300 MHz and 75 MHz respectively or Bruker Avance Neo 400 MHz NMR spectrometer at 400 MHz and 100 MHz respectively. Chemical shifts were reported in parts per million (ppm) relative to the solvent peak and coupling constants were signified in Hz.

Abbreviations: DCM: dichloromethane, ACN: acetonitrile, DMF: dimethylformamide, THF: tetrahydrofuran, EtOAc: ethyl acetate, DME: 1,2-dimethoxyethane

2. Synthesis:

tert-Butyl 1*H*-pyrrole-1-carboxylate (12):[1]

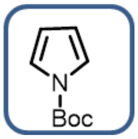

Di-*tert*-butyl dicarbonate (8.3 mL, 7.85 g, 36 mmol), triethylamine (5 mL, 3.64 g, 36 mmol) and 4-dimethylaminopyridine (0.37 g, 3 mmol) were added to a stirred solution of pyrrole (2 g, 30 mmol) in 50 mL DCM. Then the resulting reaction mixture was allowed to stir at room temperature for 6 hours. After the completion of reaction as judged by the TLC analysis, the organic residue was extracted with DCM for three times. The combined organic layer was dried over anhyd. Na_2SO_4, filtered and concentrated in *vacuo*. The organic contents were subjected to silica gel column chromatography to furnish tert-butyl 1*H*-pyrrole-1-carboxylate as a colorless liquid (4 g, yield: 80%, eluent: Hexane).
^{1}H NMR (300 MHz, $CDCl_3$) δ 7.24 (t, 2H, J = 3 Hz), 6.22 (t, 2H, J = 3 Hz), 1.60 (s, 9H).

1-(*tert*-Butoxycarbonyl)pyrrole-2-boronic acid (13):[2]

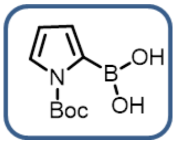

Hexane solution of *n*-BuLi (2M, 9 mL, 18.0 mmol) was added dropwise to a solution of diisopropylamine (2.75 ml, 19.5 mmol) in 30 mL dry THF under argon atmosphere at -78 ^{0}C and the reaction mixture was stirred for 15 min. Then it was warmed up to 0 ^{0}C and kept at the same temp for 15 min. Then the reaction mixture was again cooled down to -78 ^{0}C and a solution of *tert*-butyl 1*H*-pyrrole-1-carboxylate (2.5 g, 15.0 mmol) in 10 mL THF was added dropwise to that reaction mixture. After 1 hour of stirring at the same temp, trimethyl borate (2.5 ml, 22.5 mmol) was introduced dropwise into the reaction mixture and the reaction was allowed to stir for another 1 hour. Then it was gradually warmed up to room temperature and left for stirring for 18 hours. The reaction was quenched by adding 20 mL of HCl (0.25 N) and stirred for 1 hour. Then the

reaction was concentrated in *vacuo* and the organic residue was extracted with EtOAc for three times. The combined organic layer was dried over anhyd. Na_2SO_4, filtered and concentrated in *vacuo*. The organic contents were subjected to silica gel column chromatography to yield 1-(tert-butoxycarbonyl) pyrrole-2-boronic acid **13** as a white solid (1.9 g, yield: 60%, eluent: 7% EtOAc/Hexane).^{1}H NMR (300 MHz, DMSO-d_6) δ 7.33 (m, 1H), 6.42 (m, 1H), 6.22 (t, 1H, J = 3 Hz), 1.54 (s, 9H); ^{13}C NMR (100 MHz, DMSO-d_6) δ 150.3, 123.3, 120.8, 112.2, 84.5, 27.9

General procedure of Suzuki cross coupling reaction[3] for making compounds 14a-f:

At first a flame-dried pressure-tube equipped with a magnetic bar was degassed by argon gas, then 1-(tert-butoxycarbonyl) pyrrole-2-boronic acid (0.464 g, 2.2 mmol), bromobenzene (0.314 g, 2.0 mmol) as the representative compound, Na_2CO_3 (420 mg, 4.0 mmol), $Pd(PPh_3)_4$ (231 mg, 0.2 mmol) and 15 mL of DME/H_2O (3:1) were introduced under argon atm. Next, the reaction vessel was evacuated, backfilled with argon gas three times, and sealed using screw cap. The reaction mixture was then heated at 90 ^{0}C for 18 hours. After that it was allowed to cool at room temperature and extracted with EtOAc for three times. The combined organic layer was dried over anhyd. Na_2SO_4, filtered and concentrated in *vacuo*. The residue was purified using silica gel column chromatography employing 1% EtOAc in hexane. Same method was applied to prepare compounds **14b-f**.

***tert*-Butyl 2-phenyl-1*H*-pyrrole-1-carboxylate (14a):**

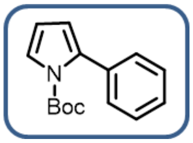

Colorless liquid (0.37 g, eluent: 1% EtOAc in hexane), yield: 76%.
^{1}H NMR (300 MHz, $CDCl_3$) δ 7.38-7.31 (m, 6H), 6.26-6.20 (m, 2H), 1.37 (s, 9H); ^{13}C NMR (75 MHz, $CDCl_3$) δ 149.4, 135.1, 134.5, 129.2, 127.6, 127.31, 127.2, 122.5, 120.0, 114.4, 111.9, 110.6, 83.6, 27.6; HRMS (ESI) m/z calcd for $C_{15}H_{18}NO_2$: 244.1332 $[M+H]^+$; found: 244.133

***tert*-butyl 2-(4-methoxyphenyl)-1*H*-pyrrole-1-carboxylate (14b):**

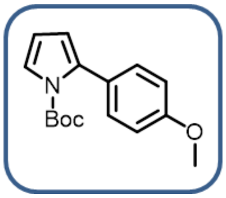

Colorless liquid (0.29 g, eluent: 2% EtOAc in hexane), yield: 50%.
^{1}H NMR (300 MHz, $CDCl_3$) δ 7.33-7.27 (m, 3H), 6.89 (d, 2H, J = 9 Hz), 6.20 (t, 1H, J = 3 Hz), 6.13 (d, 1H, J = 3 Hz), 3.83 (s, 3H), 1.39 (s, 9H); ^{13}C NMR (75 MHz, $CDCl_3$) δ 158.9, 149.4, 134.9, 130.4, 126.9, 122.1, 114, 113, 110.4, 83.4, 55.3, 27.7; HRMS (ESI) m/z calcd for $C_{16}H_{20}NO_3$: 274.1438 $[M+H]^+$; found: 274.1432.

***tert*-butyl 2-(2-methoxyphenyl)-1*H*-pyrrole-1-carboxylate (14c):**

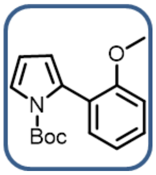

Colorless liquid (0.30 g, eluent: 3% EtOAc in hexane), yield: 55%.
^{1}H NMR (300 MHz, $CDCl_3$) δ 7.39-7.28 (m, 3H), 6.99 (t, 1H, J = 9 Hz), 6.89 (d, 1H, J = 9 Hz), 6.28 (t, 1H, J = 3Hz), 6.19-6.17 (m, 1H) 3.78 (s, 3H), 1.36 (s, 9H); ^{13}C NMR (75 MHz, $CDCl_3$) δ 157.4, 149.5, 131.2, 130.3, 129.0, 124.2, 121.9, 120.3, 113.8, 110.4, 109.9, 82.8, 55.3, 27.5; HRMS (ESI): m/z Calcd for $C_{16}H_{20}NO_3$: 274.1438 $[M+H]^+$; found 274.1445.

tert-Butyl 2-(3,4,5-trimethoxyphenyl)-1_H_-pyrrole-1-carboxylate (14d):

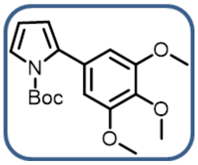

Colorless liquid (0.21 g, eluent: 3% EtOAc in hexane), yield: 63%. ^{1}H NMR (300 MHz, $CDCl_3$) δ 7.34-7.62 (m, 1H), 6.57 (s, 2H), 6.23-6.18 (m, 2H), 3.87 (s, 3H), 3.85 (s, 6H), 1.38 (s, 9H); ^{13}C NMR (100 MHz, $CDCl_3$) δ 152.5, 149.3, 134.8, 130.0, 129.0, 128.2, 122.4, 114.2, 110.5, 106.6, 83.5, 60.9, 56.1, 27.7; HRMS (ESI): m/z Calcd for $C_{18}H_{24}NO_5$: 334.1649 $[M+H]^+$; found 334.1643.

tert-Butyl 2-(4-(ethoxycarbonyl)phenyl)-1_H_-pyrrole-1-carboxylate (14e):

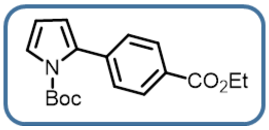

Grey solid (0.34 g, eluent: 10% EtOAc in hexane), yield: 54%. ^{1}H NMR (300 MHz, $CDCl_3$) δ 8.03 (d, 2H, J = 9 Hz), 7.41 (d, 2H, J = 9 Hz), 7.38 (t, 1H, J = 3 Hz), 6.27-6.23 (m, 2H,), 4.39 (q, 2H, J = 9Hz), 1.40 (t, 3H, J = 9 Hz), 1.38 (s, 9H); ^{13}C NMR (100 MHz, $CDCl_3$) δ 166.5, 149.2, 138.8, 134.0, 128.9, 128.9, 123.5, 115.4, 110.8, 84.1, 60.9, 27.7, 14.4; HRMS (ESI): m/z Calcd for $C_{18}H_{22}NO_4$: 316.1543 $[M+H]^+$; found 316.1538.

tert-Butyl 2-(4-cyanophenyl)-1_H_-pyrrole-1-carboxylate (14f):

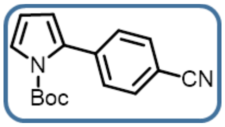

White solid (0.31 g, eluent: 12% EtOAc in hexane), yield: 59%. ^{1}H NMR (400 MHz, $CDCl_3$) δ 7.63(d, 2H, J = 8Hz), 7.45(d, 2H, J =12Hz), 7.39-7.38(m, 1H), 6.28-6.25(m, 2H), 1.42(s, 9H); ^{13}C NMR (100 MHz, $CDCl_3$) δ 143.6, 133.5, 127.8, 126.1, 124.3, 118.7, 113.7, 110.8, 105.8, 105.2, 79.1, 22.4; HRMS (ESI): m/z Calcd for $C_{16}H_{17}N_2O_2$: 269.1285 $[M+H]^+$; found 269.1288.

General procedure for Boc-deprotection to prepare compounds 15a-f:[4]

Sodium ethoxide (0.15 g, 1.5 mmol) was added slowly to the **14a** (0.37 g, 1 mmol), as the representative compound, in dry 5 mL THF and reaction mixture was stirred at room temperature for 2-3 hours. After the completion of reaction as judged by the TLC analysis, the excess sodium ethoxide was quenched by adding saturated NH_4Cl solution. Then the reaction mixture was dried in *vacuo* and extracted with EtOAc for three times. The combined organic layer was dried over anhyd. Na_2SO_4, filtered and concentrated in *vacuo*. The residue was purified using silica gel column chromatography employing 7% EtOAc in hexane to yield **15a.** Same method was applied to prepare compounds **15b-f**.

2-Phenyl-1_H_-pyrrole (15a):

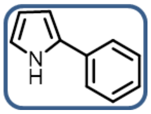

Grey solid (0.155 g, 7% EtOAc in hexane), yield: 71%. ^{1}H NMR (400 MHz, $CDCl_3$) δ 8.44 (brs, 1H), 7.50-7.47(m, 2H), 7.40-7.35 (m, 2H), 7.24-7.20 (m, 1H), 6.88-6.86 (m, 1H) 6.55-6.53 (m, 1H) 6.33-6.30 (m, 1H); ^{13}C NMR (75 MHz, $CDCl_3$) δ 132.8, 132.2, 128.9, 126.2, 123.8, 118.9, 110.2, 106.0; HRMS (ESI) m/z calcd for $C_{10}H_{10}N$: 144.0808 $[M+H]^+$; found: 144.0802.

2-(4-Methoxyphenyl)-1*H*-pyrrole (15b):

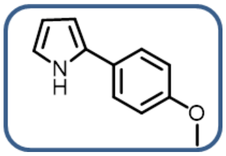

White solid (0.115 g, 10% EtOAc in hexane), yield: 73%. ^{1}H NMR (300 MHz, $CDCl_3$) δ 8.33(brs, 1H), 7.405 (d, 2H, J = 9 Hz), 6.925 (d, 2H, J = 9Hz) 6.83 (s, 1H), 6.42 (s, 1H), 6.29 (s, 1H), 3.83 (s, 3H); ^{13}C NMR (75 MHz, $CDCl_3$) δ 158.3, 132.2, 125.9, 125.3, 118.2, 114.4, 110.0, 104.9, 55.4; HRMS (ESI): m/z Calcd for $C_{11}H_{12}NO$: 174.0913 $[M+H]^+$; found 174.091.

2-(2-Methoxyphenyl)-1*H*-pyrrole (15c):

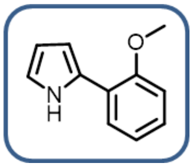

White solid (0.125 g, 10% EtOAc in hexane), yield: 70%. ^{1}H NMR (300 MHz, $CDCl_3$) δ 9.83 (brs, 1H), 7.69-7.66 (m, 1H), 7.19-7.14 (m, 1H), 7.02-6.96 (m, 2H), 6.88-6.86 (m, 1H) 6.64-6.61 (m, 1H) 6.31-6.28 (m, 1H), 3.97(s, 3H); ^{13}C NMR (100 MHz, $CDCl_3$) δ 154.7, 129.9, 126.7, 126.6, 121.5, 121.1, 117.8, 111.6, 108.9, 106.1, 55.7; HRMS (ESI): m/z Calcd for $C_{11}H_{12}NO$: 174.0913 $[M+H]^+$; found 174.0916.

2-(3,4,5-Trimethoxyphenyl)-1*H*-pyrrole (15d):

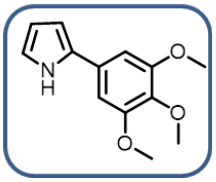

White solid (0.095 g, 8% EtOAc in hexane), yield: 68%. ^{1}H NMR (300 MHz, $CDCl_3$) δ 8.48 (brs, 1H), 6.87-6.85 (m, 1H), 6.68 (s, 2H), 6.46-6.43 (m, 1H), 6.31-6.28 (m, 1H), 3.90(s, 6H), 3.86 (s, 3H); ^{13}C NMR (75 MHz, $CDCl_3$) δ 153.6, 136.8, 132.3, 129.0, 118.7, 110.1, 105.9, 101.7, 61.0, 56.2; HRMS (ESI): m/z Calcd for $C_{13}H_{16}NO_3$: 234.1125 $[M+H]^+$; found 234.1121.

Ethyl 4-(1*H*-pyrrol-2-yl)benzoate (15e):

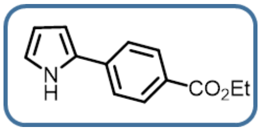

Grey solid (0.14 g,15% EtOAc in hexane), yield: 65%. ^{1}H NMR (400 MHz, $CDCl_3$) δ 8.60 (brs, 1H), 8.01 (d, 2H, J = 8 Hz), 7.52 (d, 2H, J = 8Hz), 6.94-6.92 (m, 1H), 6.67-6.65 (m, 1H), 6.35-6.32 (m, 1H), 4.38 (q, 2H, J = 8 Hz), 1.40 (t, 3H, J = 8 Hz); ^{13}C NMR (75 MHz, $CDCl_3$) δ 166.6, 136.8, 131.0, 130.3, 127.6, 123.1, 120.3, 110.6, 107.9, 61.0, 14.4; HRMS (ESI): m/z Calcd for $C_{13}H_{14}NO_2$: 216.1019 $[M+H]^+$; found 216.1015.

4-(1*H*-Pyrrol-2-yl)benzonitrile (15f):

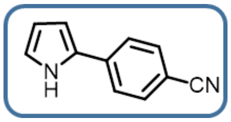

White solid (0.115 g, 15% EtOAc in hexane), yield: 68%. ^{1}H NMR (300 MHz, $CDCl_3$) δ 8.79 (brs, 1H), 7.61(d, 2H, J = 9 Hz), 7.53(d, 2H, J = 9Hz), 6.97-6.95(m, 1H), 6.69-6.67(m,1H), 6.36-6.33(m,1H); ^{13}C NMR (75 MHz, $CDCl_3$) δ 136.9, 132.8, 130.1, 123.7, 121.1, 119.4, 111.0, 108.8, 108.5; HRMS (ESI): m/z Calcd for $C_{11}H_9N_2$: 169.0760 $[M+H]^+$; found 169.0766.

General procedure for Vilsmeier-heck reaction to prepare compounds 16a-f:

Freshly distilled $POCl_3$ (109 µL, 1.2 mmol) was added to a solution of **15a** (0.143 g., 1 mmol), as the representative compound, in 2 mL of DMF at 0 ^{0}C. Reaction mixture was allowed to reach at room temperature and stirred for 3 hours. After that, it was poured into ice-cold water, basified with NaOH solution to adjust pH of the solution to ca. 12. The mixture was then extracted with ethyl acetate and the combined organic layer was dried over anhyd. Na_2SO_4, filtered and concentrated in *vacuo*. The residue was purified using silica gel column chromatography employing 10% EtOAc in hexane to furnish the desired product **16a**. Identical method was employed to synthesize compounds **16b-f**.

5-Phenyl-1*H*-pyrrole-2-carbaldehyde (16a):

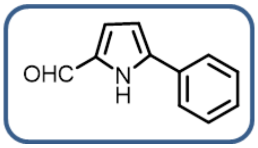

Pink solid (0.12 g, 10% EtOAc in hexane), yield: 72%. ^{1}H NMR (400 MHz, $CDCl_3$) δ 9.82(brs, 1H), 9.52(s, 1H), 7.60-7.56(m, 2H), 7.01(dd, 1H, J = 2.4 Hz, 4 Hz), 6.98-6.94(m, 2H), 6.55(dd, 1H, J = 2.4 Hz, 4 Hz); ^{13}C NMR (100 MHz, $CDCl_3$) δ 178.9, 140.0, 133.3, 130.6, 129.2, 128.6, 125.3, 122.8, 109.0; HRMS (ESI): m/z Calcd for $C_{11}H_{10}NO$: 172.0757 $[M+H]^+$; found 172.0752.

5-(4-Methoxyphenyl)-1*H*-pyrrole-2-carbaldehyde (16b):

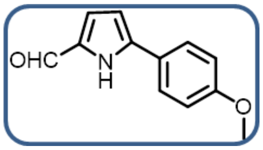

Grey solid (0.16 g, 15% EtOAc in hexane), yield: 81%. ^{1}H NMR (400 MHz, $CDCl_3$) δ 9.85(brs, 1H), 9.47(s, 1H), 7.65-7.62(m, 2H), 7.46-7.42(m, 2H), 7.38-7.34(m, 1H), 7.03(dd, 1H, J = 2.4 Hz, 4 Hz), 6.65(dd, 1H, J = 2.4 Hz, 4 Hz); ^{13}C NMR (100 MHz, $CDCl_3$) δ 178.5, 160.0, 140.4, 132.9, 126.8, 123.4, 123.3, 114.6, 108.2, 55.4; HRMS (ESI): m/z Calcd for $C_{12}H_{12}NO_2$: 202.0863 $[M+H]^+$; found 202.0867.

5-(2-Methoxyphenyl)-1*H*-pyrrole-2-carbaldehyde (16c):

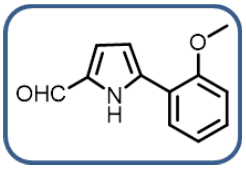

White solid (0.155 g, 10% EtOAc in hexane), yield: 74%. ^{1}H NMR (400 MHz, $CDCl_3$) δ 10.56(brs, 1H), 9.49(s, 1H), 7.72(dd, 1H, J = 1.6 Hz, 7.6 Hz), 7.33-7.29(m, 1H), 7.06-6.99(m, 3H), 6.72(dd, 1H, J = 2.4 Hz, 4 Hz); ^{13}C NMR (100 MHz, $CDCl_3$) δ 178.5, 156.0, 137.7, 132.3, 129.5, 128.1, 121.5, 121.4, 118.7, 111.7, 109.1, 55.8; HRMS (ESI): m/z Calcd for $C_{12}H_{12}NO_2$: 202.0863 $[M+H]^+$; found 202.0865.

5-(3,4,5-Trimethoxyphenyl)-1*H*-pyrrole-2-carbaldehyde (16d):

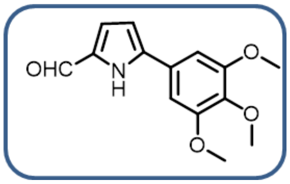

Yellow solid (0.13 g, 12% EtOAc in hexane), yield: 75%. ^{1}H NMR (300 MHz, $CDCl_3$) δ 10.5(brs, 1H), 9.41(s, 1H), 7.08-7.06(m, 1H), 6.95(s, 2H), 6.62-6.60(m, 1H), 3.94(s, 6H), 3.89(s, 3H); ^{13}C NMR (100 MHz, $CDCl_3$) δ 178.5, 153.8, 140.9, 138.7, 138.7, 133.1, 126.3, 123.7, 109.1, 102.9, 61.0, 56.3; HRMS (ESI): m/z Calcd for $C_{14}H_{16}NO_4$: 262.1074 $[M+H]^+$; found 262.107.

Ethyl 4-(5-formyl-1*H*-pyrrol-2-yl)benzoate (16e):

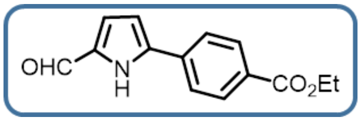

White solid (0.11 g, 20% EtOAc in hexane), yield: 80%. ^{1}H NMR (400 MHz, $CDCl_3$) δ 10.13(brs, 1H), 9.55(s, 1H), 8.10(d, 2H, J = 8Hz), 7.72(d, 2H, J = 8Hz), 7.07-7.05(m, 1H), 6.75-6.73(m, 1H), 4.40(q, 2H, J = 8Hz), 1.41(t, 3H, J = 8Hz); ^{13}C NMR (100 MHz, $CDCl_3$) δ 179.3, 166.1, 138.6, 134.6, 133.9, 130.4, 130.2, 125.0, 122.7, 110.2, 61.2, 14.3; HRMS (ESI): m/z Calcd for $C_{14}H_{14}NO_3$: 244.0968 $[M+H]^+$; found 244.0965.

4-(5-Formyl-1H-pyrrol-2-yl)benzonitrile (16f):

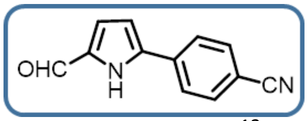

White solid (0.088 g, 20% EtOAc in hexane), yield: 76%. ^{1}H NMR (400 MHz, $CDCl_3$) δ ^{1}H NMR (400 MHz, $CDCl_3$) δ 10.59(brs, 1H), 9.58(s, 1H), 7.82(d, 2H, J = 8Hz), 7.72(d, 2H, J = 8Hz), 7.10-7.08(m, 1H), 6.77-6.76(m, 1H); ^{13}C NMR (75 MHz, $CDCl_3$) δ 179.7, 137.9, 134.9, 134.3, 132.9, 125.7, 123.1, 118.6, 111.6, 110.7; HRMS (ESI): m/z Calculated for $C_{12}H_9N_2O$: 197.0709 $[M+H]^+$; found 197.0705.

General procedure for the synthesis of arylpyrrolylidene-indanones (1, 3-10):

Synthesis of a representative arylpyrrolylidene-indanone **1** was described as follow: to a 5 mL toluene solution of 5-phenyl-2-formylpyrrole (0.04 g, 0.234 mmol) and 1-indanone (0.031 g, 0.234 mmol), piperidine (36 µL, 0.35 mmol) was added dropwise under argon gas atmosphere, and the resulting solution was refluxed for 18 hours. The reaction was cooled down to room temperature, evaporated the solvent and extracted with ethyl acetate. Organic layer was combined, dried over anhyd. sodium sulphate, filtered and concentrated. The crude product was subjected to silica-gel column chromatography using EtOAc/pet to obtain pure *Z*- and *E*-isomers of the desired compound **1**. Identical method was applied to prepare *E* and *Z*-isomers of **4-10** using respective aldehydes.

(*Z*)-2,3-Dihydro-2-((5-phenyl-1H-pyrrol-2-yl)methylene)inden-1-one (1):

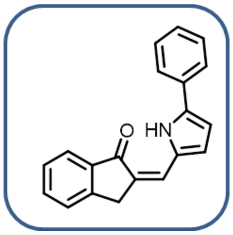

Z-isomer: red-orange solid (35 mg, 1% EtOAc/hexane), yield: 70%.
E-isomer: brick red (8 mg, 10% EtOAc/hexane), yield: 16%.
^{1}H NMR (300 MHz, $CDCl_3$) δ 14.04 (brs, 1H), 7.945 (d, 1H, J = 9 Hz), 7.815 (d, 2H, J = 9 Hz) 7.60-7.44 (m, 5H), 7.31 (d, 1H, J = 9 Hz), 6.95 (s, 1H), 6.74 (s, 1H), 6.69 (s, 1H), 3.87 (s, 2H); ^{13}C NMR (75 MHz, $CDCl_3$) δ 192.6, 148.9, 140.7, 137.1, 133.6, 132.1, 131.5, 129.6, 129.1, 127.6, 127.4, 125.7, 125.6, 124.6, 123.9, 120.3, 109.5, 34.7; HRMS (ESI) m/z calcd for $C_{20}H_{16}NO$: 286.1226 $[M+H]^+$; found 286.1222.

(Z)-2-((1*H*-pyrrol-2-yl)methylene)-2,3-dihydro-1*H*-inden-1-one (3):

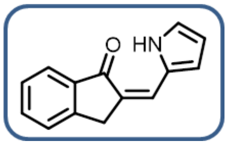

Z-isomer: greenish yellow (27 mg, 1% EtOAc/hexane), yield: 72%.
E-isomer: canary yellow (7 mg, 15% EtOAc/hexane), yield: 15%.
^{1}H NMR (300 MHz, $CDCl_3$) δ 13.4(brs, 1H), 7.9(d, 1H, *J* = 9Hz), 7.64-7.59(m, 1H), 7.52(d, 1H, *J* = 9Hz), 7.46(t, 1H, *J* = 9Hz), 7.14-7.12(m, 1H), 6.97(s, 1H), 6.65-6.63(m, 1H), 6.40-6.37(m, 1H), 3.87(s, 2H); ^{13}C NMR (100 MHz, $CDCl_3$) δ 193.0, 149.1, 140.5, 133.8, 131.0, 129.9, 127.4, 125.8, 124.0, 123.5, 118.5, 111.4, 34.5; HRMS (ESI): m/z Calcd for $C_{20}H_{16}NO$: 210.0913 $[M+H]^+$; found 210.0911.

(*Z*)-2,3-Dihydro-2-((5-(4-methoxyphenyl)-1*H*-pyrrol-2-yl)methylene)inden-1-one (**4**):

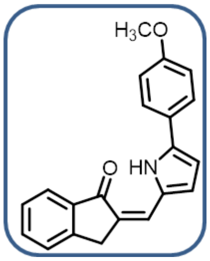

Z-isomer: dark red (33 mg, 3% EtOAc/hexane), yield: 70%.
E-isomer: red solid (4 mg, 20% EtOAc/hexane), yield: 9%.
^{1}H NMR (400 MHz, DMSO-d_6) δ 14.03(brs, 1H), 7.875(d, 1H, *J* = 4Hz), 7.77-7.64(m, 4H), 7.51(t, 1H, *J* = 8 Hz), 7.16-7.11(m, 3H), 6.86-6.83(m, 2H), 3.91(s, 2H), 3.84 s, 3H); ^{13}C NMR (75 MHz, $CDCl_3$) δ 192.6, 148.9, 140.7, 137.1, 133.6, 132.1, 131.5, 129.6, 129.1, 127.6, 127.4, 125.7, 125.5, 124.6, 123.9, 120.3, 109.5, 34.7; HRMS (ESI): m/z Calcd for $C_{21}H_{18}NO_2$: 316.1332 $[M+H]^+$; found 316.1331.

(*Z*)-2,3-Dihydro-2-((5-(2-methoxyphenyl)-1*H*-pyrrol-2-yl)methylene)inden-1-one (**5**):

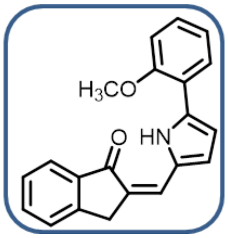

Z-isomer: crimson red (30 mg, 3% EtOAc/hexane), yield: 71%.
E-isomer: orange solid (3 mg, 15% EtOAc/hexane), yield: 7%.
^{1}H NMR (300 MHz, $CDCl_3$) δ 14.51(brs, 1H), 8.30(d, 1H, *J* = 6 Hz), 8.145(d, 1H, *J* = 9 Hz) 7.98-7.78(m, 3H), 7.78-7.62(m, 2H), 7.44-7.38(m, 2H), 7.20-7.18(m, 1H), 7.04-7.02(m, 1H), 4.57(s, 3H), 4.22(s, 2H); ^{13}C NMR (100 MHz, $CDCl_3$) δ 192.51, 156.40, 148.87, 140.97, 134.84, 133.25, 131.29, 129.09, 128.40, 127.43, 127.21, 125.69, 125.15, 123.80, 120.97, 120.38, 119.77, 111.70, 110.57, 55.62, 34.64; HRMS (ESI): m/z Calcd for $C_{21}H_{18}NO_2$: 316.1332 $[M+H]^+$; found 316.1332.

(*Z*)-2-((5-(3,4,5-Trimethoxyphenyl)-1*H*-pyrrol-2-yl)methylene)-2,3-dihydro-1*H*-inden-1-one (**6**):

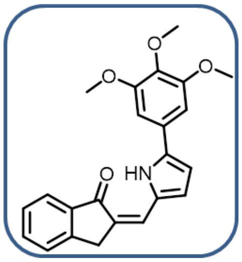

Z-isomer: red red (25 mg, 10% EtOAc/hexane), yield: 68%.
E-isomer: yellow solid (5 mg, 30% EtOAc/hexane), yield: 13%.
^{1}H NMR (400 MHz, $CDCl_3$) δ 14.09(brs, 1H), 7.92(d, 1H, *J* = 8 Hz), 7.59(t, 1H, *J* = 8 Hz), 7.51 (d, 1H, *J* = 8 Hz), 7.44(t, 1H, *J* = 8 Hz), 7.02(s, 2H), 6.93(s, 1H), 6.67-6.66 (m, 2H), 4.02 (s, 6H), 3.90(s, 3H), 3.87(s, 2H); ^{13}C NMR (100 MHz, $CDCl_3$) δ 192.5, 153.7, 149.0, 140.7, 138.0, 137.1, 133.6, 132.1, 129.4, 127.6, 127.4, 125.7, 125.6, 124.1, 120.1, 109.3, 102.1, 61.1, 56.3, 34.7; HRMS (ESI): m/z Calcd for $C_{23}H_{22}NO_4$: 376.1543 $[M+H]^+$; found 376.1542.

(*Z*)-Ethyl 4-(5-((1-oxo-1*H*-inden-2(3*H*)-ylidene)methyl)-1*H*-pyrrol-2-yl)benzoate (7):

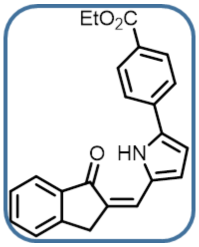

Z-isomer: red solid (30 mg, 7% EtOAc/hexane), yield: 55%.
E-isomer: yellow solid (6 mg, 30% EtOAc/hexane), yield: 11%.
^{1}H NMR (400 MHz, $CDCl_3$) δ 14.13(brs, 1H), 8.12(d, 1H, J = 8 Hz), 7.95(d, 2H, J = 8Hz), 7.84(d, 2H, J = 8 Hz), 7.61(t, 1H, J = 8Hz), 7.51(d, 1H, J = 8Hz), 7.46(t, 1H, J = 8Hz), 6.95(s, 1H), 6.83-6.81(m, 1H), 6.69-6.68(m, 1H), 4.40(q, 2H, J = 8Hz), 3.88(s, 2H). 1.42(t, 3H, J = 8Hz); ^{13}C NMR (100 MHz, $CDCl_3$) δ 187.6, 161.1, 143.7, 135.2, 130.3, 130.2, 128.6, 127.6, 125.1, 124.0, 123.6, 122.2, 121.5, 120.5, 118.8, 118.8, 114.8, 105.4, 55.7, 29.4, 9.1; HRMS (ESI): m/z Calcd for $C_{23}H_{20}NO_3$: 358.1438 $[M+H]^+$; found 358.1439.

(Z)-4-(5-((1-Oxo-1*H*-inden-2(3*H*)-ylidene)methyl)-1*H*-pyrrol-2-yl)benzonitrile (8):

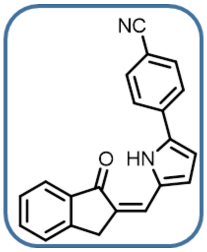

Z-isomer: orange solid (28 mg, 7% EtOAc/hexane), yield: 60%.
E-isomer: yellow solid (5 mg, 30% EtOAc/hexane), yield: 10%.
^{1}H NMR (400 MHz, $CDCl_3$) δ 14.18 brs, 1H), 7.945(d, 1H, J = 4 Hz), 7.84(d, 2H, J = 8Hz), 7.71(d, 2H, J = 12Hz), 7.63(t, 1H, J = 8Hz), 7.52(d, 1H, J = 8 Hz), 7.47(t, 1H, J = 8 Hz), 6.96 (s,1H), 6.82-6.81(m, 1H), 6.69-6.67(m, 1H), 3.89(s, 2H); ^{13}C NMR (100 MHz, $CDCl_3$) δ 187.9, 143.8, 135.0, 130.4, 128.9, 128.9, 128.0, 127.6, 124.0, 122.4, 122.3, 120.5, 119.3, 118.9, 114.6, 113.8, 105.8, 104.7, 29.4; HRMS (ESI): m/z Calcd for $C_{21}H_{15}N_2O$: 311.1179 $[M+H]^+$; found 311.1176.

Methyl 3,3-diphenylacrylate (17):[5]

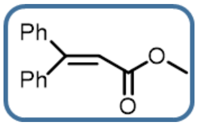

Iodobenzene (0.6 g, 3.0 mmol, 330 µl) and methyl acrylate (0.09 gm., 1.0 mmol) were added to a suspension of AgOAc (0.5 g, 3.0 mmol) and $Pd(OAC)_2$ (0.007 g, 0.01 mmol) in 3 mL AcOH. The reaction mixture was stirred under argon atmosphere at 110 ^{0}C for 6 hours. After that the reaction mixture was allowed to cool at room temperature, diluted with EtOAc, and filtered through celite pad. The filtrate was extracted with EtOAc for three times. The combined organic layer was dried over anhyd. Na_2SO_4, filtered and concentrated in *vacuo*. The organic contents were subjected to silica gel column chromatography to get methyl 3,3-diphenylacrylate as a white solid (0.2 gm., 84%, eluent: 5% EtOAc/Hexane). ^{1}H NMR (300 MHz, $CDCl_3$) δ 7.42-7.28 (m, 8H), 7.24-7.20 (m, 2H), 6.37 (s, 1H), 3.62 (s, 3H); ^{13}C NMR (100 MHz, $CDCl_3$) δ 166.5, 157.1, 140.8, 138.8, 129.5, 129.1, 128.4, 128.3, 128.2, 127.9, 116.8, 51.3.

3,3-Diphenylacrylic acid (18):

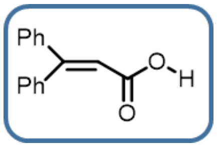

To a solution of methyl 3,3-diphenylacrylate (0.2 g, 0.84 mmol) in a 3:1:1 mixture of THF/MeOH/H_2O solvents, LiOH.H_2O (0.07g, 1.6 mmol) was introduced and the resulting reaction mixture was stirred at room temperature. After completion of the reaction, as judged by the TLC analysis, the reaction mixture was concentrated in *vacuo*, neutralized with 2N HCl solution, and extracted with EtOAc for three times. The combined organic layer was dried over anhyd. Na_2SO_4, filtered and concentrated in *vacuo*. The organic contents were subjected to silica gel column chromatography to yield **18** as a white solid (0.17 g, yield: 88%, eluent: 25% EtOAc/Hexane). ^{1}H NMR (300 MHz, $CDCl_3$) δ 7.39-7.27 (m, 8H), 7.23-7.20 (m, 2H), 6.33 (s, 1H); ^{13}C NMR (100 MHz, $CDCl_3$) δ 170.9, 159.0, 140.8, 138.4, 129.8, 129.3, 128.5, 128.4, 127.9, 116.4.

3,3-Diphenyl-2,3-dihydro-1*H*-inden-1-one (19):[6]

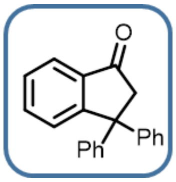

Triflic acid (140 µL, 0.24 g, 1.6 mmol) was carefully added to a stirred solution of 3,3-diphenylacrylic acid (0.12 gm., 0.5 mmol) and benzene (576 µL, 0.5 g, 6.4 mmol) in 5 mL of dichloroethane under argon atmosphere under ice-cold condition. Then the reaction mixture was allowed to stir at 70 ^{0}C for 12 hours. After the completion of reaction, as judged by the TLC analysis, it was quenched by adding cursed ice carefully. The organic residue was extracted with dichloromethane for three times. The combined organic layer was dried over anhyd. Na_2SO_4, filtered and concentrated in *vacuo*. The organic contents were subjected to silica gel column chromatography to obtain 3,3-diphenyl-2,3-dihydro-1H-inden-1-one as a light-yellow solid (0.12 g, yield: 75%, eluent: 5% EtOAc/Hexane). ^{1}H NMR (300 MHz, $CDCl_3$) δ 7.80 (d, 1H, *J* = 9 Hz), 7.60 (t, 1H, *J* = 9 Hz), 7.43 (t, 1H, *J* = 9 Hz), 7.36 (d, 1H, *J* = 9 Hz), 7.32-7.21 (m, 6H), 7.20-7.14 (m, 4H), 3.49 (s, 2H); ^{13}C NMR (75 MHz, $CDCl_3$) δ 205.1, 159.5, 146.8, 135.8, 134.9, 128.5, 128.1, 128.0, 126.6, 123.7, 56.1.

(*Z*)-2-((5-(4-Methoxyphenyl)-1*H*-pyrrol-2-yl)methylene)-3,3-dimethyl-2,3-dihydro-1*H*-inden-1-one (9):

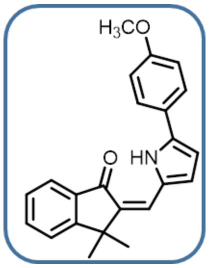

Compound **10** was prepared by aldol condensation reaction of 3,3-dimethyl-2,3-dihydro-1*H*-inden-1-one and 5-(4-methoxyphenyl)-1*H*-pyrrole-2-carbaldehyde (**16b**) by following the synthetic method of the preparation of compound **1,** as described before. Compound **10** appeared as an orange-red solid, (35 mg, eluent: 3% EtOAc/Hexane), yield: 55%. ^{1}H NMR (400 MHz, $CDCl_3$) δ 13.98 (brs, 1H), 7.89 (d, 1H, J = 8 Hz), 7.75 (d, 2H, *J* = 8 Hz), 7.62 (t, 1H, *J* = 8 Hz), 7.50 (d, 1H, *J* = 8 Hz), 7.42 (t, 1H, *J* = 8 Hz), 7.00 (d, 2H, *J* = 12 Hz), 6.83 (s, 1H), 6.71-6.70 (m, 1H), 6.67-6.65 (m, 1H), 3.87(s, 2H), 1.54 (s, 6H); ^{13}C NMR (100 MHz, $CDCl_3$) δ 192.3, 159.3, 158.3, 137.7, 137.4, 135.9, 134.1, 131.8, 127.7, 126.1, 124.5, 123.7, 123.2, 120.5, 114.5, 108.7, 55.4, 43.4, 29.2; HRMS (ESI): m/z Calculated for $C_{23}H_{22}NO_2$ $[M+H]^+$ 344.1645, found 344.1648.

(*Z*)-2-((5-(4-Methoxyphenyl)-1*H*-pyrrol-2-yl)methylene)-3,3-diphenyl-2,3-dihydro-1*H*-inden-1-one (10):

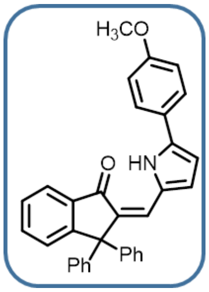

Similar method as that of the preparation of **10** was employed for the synthesis of compound **11** from 3,3-diphenyl-2,3-dihydro-1*H*-inden-1-one and 5-(4-methoxyphenyl)-1*H*-pyrrole-2-carbaldehyde (**16b**). Compound **11** appeared as a red solid (30 mg, eluent: 5% EtOAc in hexane), yield: 53%. ^{1}H NMR (300 MHz, $CDCl_3$) δ 14.07 (brs, 1H), 7.94 (d, 1H, J = 6 Hz), 7.74 (d, 1H, J = 9 Hz), 7.55 (t, 1H, J = 9 Hz), 7.47-7.40 (m, 2H), 7.35-7.21 (m, 11H), 7.01 (d, 1H, J = 9 Hz), 6.75 (s, 1H), 6.65-6.58 (m, 2H), 3.87(s, 3H); ^{13}C NMR (100 MHz, $CDCl_3$) δ 192.5, 159.5, 156.2, 146.3, 138.6, 138.3, 134.4, 134.0, 132.7, 131.8, 128.6, 128.3, 128.0, 126.7, 126.4, 126.2, 124.3, 123.6, 122.1, 114.6, 109.2, 62.1, 55.4; HRMS (ESI): m/z Calculated for $C_{33}H_{26}NO_2$ $[M+H]^+$ 468.1958, found 468.1957.

(*E*)-2-((1-methyl-5-phenyl-1*H*-pyrrol-2-yl)methylene)-2,3-dihydro-1*H*-inden-1-one (2):

Sodium hydride (4.5 mg, 0.18 mmol) was added portion wise to a solution of compound **2** (25

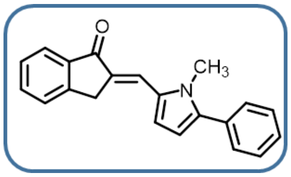

mg, 0.09 mmol) in dry DMF at 0 ^{0}C under argon atm. After 15 min methyl iodide (20 mg, 0.13 mmol) was added to that reaction mixture at 0 ^{0}C and it was allowed to stir at room temperature for 3 hours. After the completion of reaction as judged by the TLC analysis, excess sodium hydride was quenched by adding cursed ice carefully. The organic residue was extracted with DCM for multiple times. The combined organic layer was dried over anhyd. Na_2SO_4, filtered and concentrated in *vacuo*. The organic contents were subjected to silica gel column chromatography to get compound **3** as a light-yellow solid (15 mg, 58%, eluent: 10% EtOAc/Hexane). ^{1}H NMR (400 MHz, $CDCl_3$) δ 7.90 (d, 1H, J = 8 Hz), 7.75 (s, 1H) 7.62-7.60 (m, 2H), 7.58-7.37 (m, 6H), 6.855 (d, 1H, J = 4 Hz), 6.83 (d, 1H, J = 4 Hz), 3.9 (s, 2H), 3.79 (s, 3H); ^{13}C NMR (100 MHz, $CDCl_3$) δ 193.8, 149.0, 140.1, 139.1, 134.0, 132.3, 131.4, 130.0, 129.1, 128.6, 128.0, 127.5, 126.0, 124.0, 121.5, 115.1, 111.5, 33.0, 32.2; HRMS (ESI): m/z Calculated for $C_{21}H_{18}NO$ $[M+H]^+$ 300.1383, found 300.1381.

3. Photoswitching studies

3.1. General method: UV-Vis absorption spectra were recorded on a Shimadzu UV-1900 spectrophotometer or HITACHI UH4150 spectrophotometer, coupled to a temperature-controlled cuvette holder. Spectra were collected in a quartz cuvette with a 1 cm path length (1, 2, or 3 mL), at an angle of 90 ^{0} to the measuring beam. The sample solutions in dichloromethane were exposed to LEDs of wavelengths 365 nm (HOUKEM-3W-365nm), 385 nm (HOUKEM-3W-375), 400 nm (SN-UV-400nm-1W, power 1-3W, Shining-led/Houkem), 415 nm (SN-UV-415nm-1W, power 1-3W, Shining-led/Houkem), 450 nm (HOUKEM-3W-450nm), 475 nm (HOUKEM-3W-475nm), 530 nm (CREEXPE2-GRN-1, XPEBGRL1-0000-00D01, 1part up, P = 3W) or 550 nm (HOUKEM-3W-550).

3.2. UV-Vis absorption spectra of APIs 1, 3-10

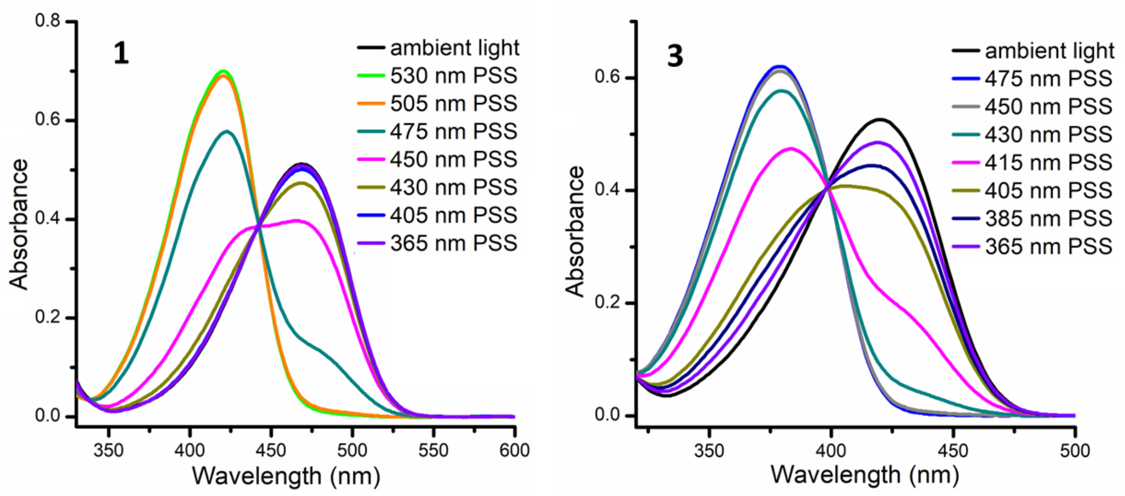

Fig. S1. UV-Vis absorption spectra of compound **1, 3** after irradiations with various wavelengths of light in dichloromethane.

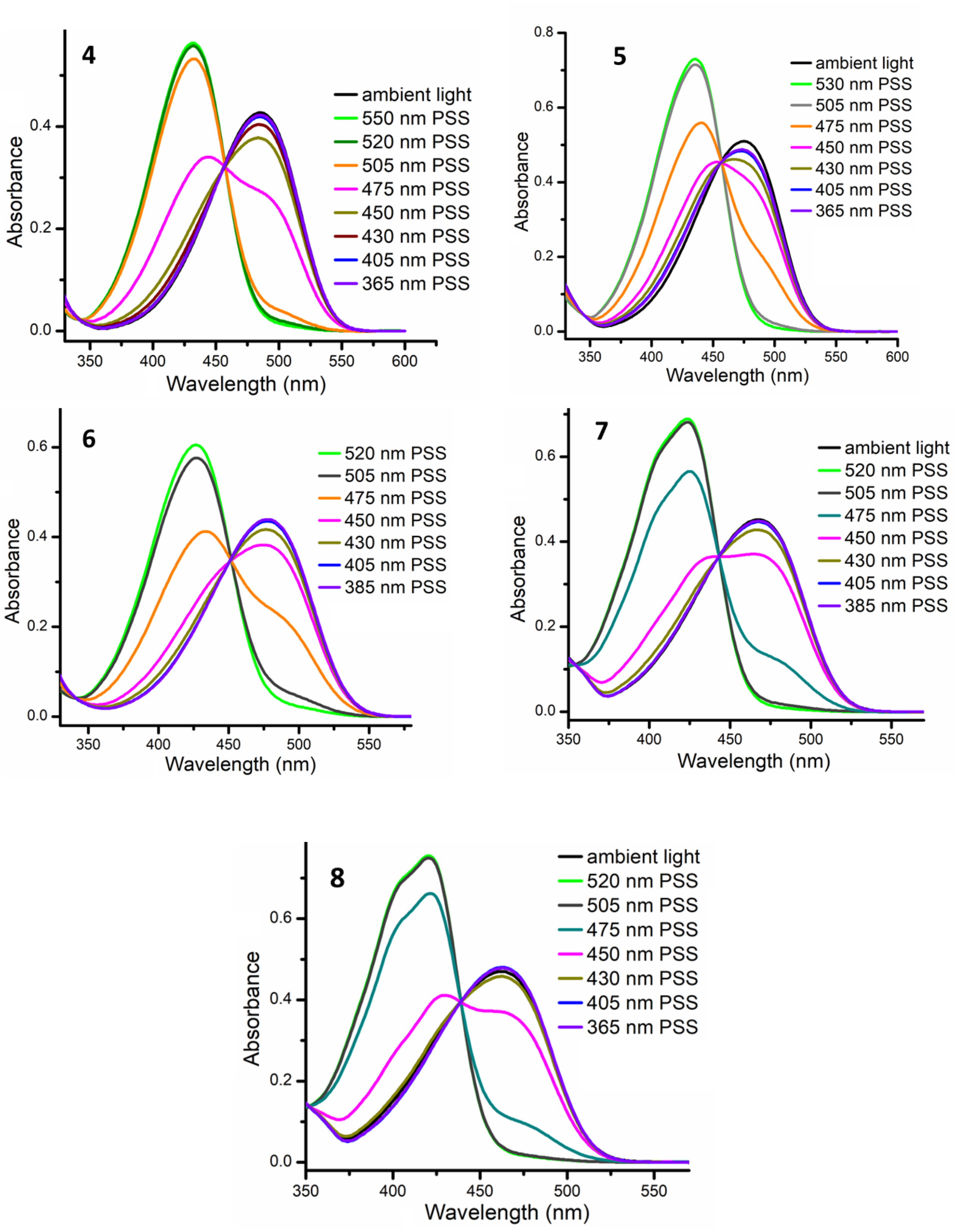

Fig. S2. UV-Vis absorption spectra of compound **4**-**8** after irradiations with various wavelengths of light in dichloromethane.

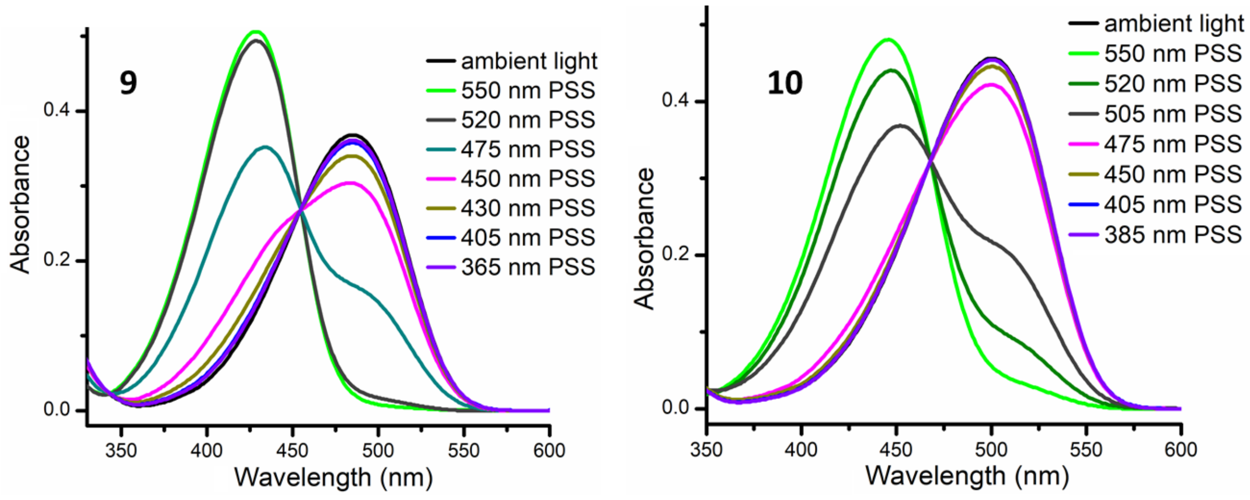

Fig. S3. UV-Vis absorption spectra of compound **9**, **10** after irradiations with various wavelengths of light in dichloromethane.

3.3. Determination of the compositions of photostationary states using ^{1}H NMR with acquisition of corresponding UV-Vis absorption spectra.

^{1}H NMR spectroscopy was used to determine the population of PSSs for the *Z*-*E* and *E*-*Z* isomerisations. $CDCl_3$ solutions of **1**-**11** (ca. 500 μM) were irradiated with LEDs of wavelengths 475 nm, 520 nm, 530 nm or 550 nm for the forward *Z*-*E* isomerisation, and 365 nm, 385 nm or 405 nm for the reverse *Z*-*E* isomerisation and ^{1}H NMR spectra of the attained PSSs were recorded immediately. From the signal integration of distinct aliphatic or aromatic protons of *E*- and *Z*-isomers, their percentages were determined. These samples in the PSSs were diluted to 15-20 μM in dichloromethance and immediately recorded the corresponding absorption spectra. Again these DCM solutions were subjected to *E*-*Z* and *Z*-*E* isomerisation to reestablish the PSSs and recorded the absorption spectra. For almost all compounds, no change in the absorption spectra was noticed, indicating that the compositions of PSSs in $CDCl_3$ and DCM are identical.

a)

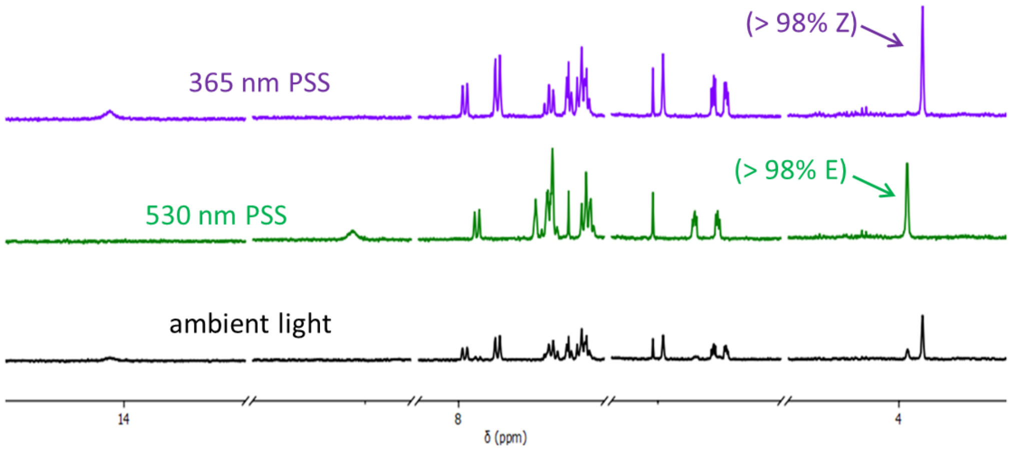

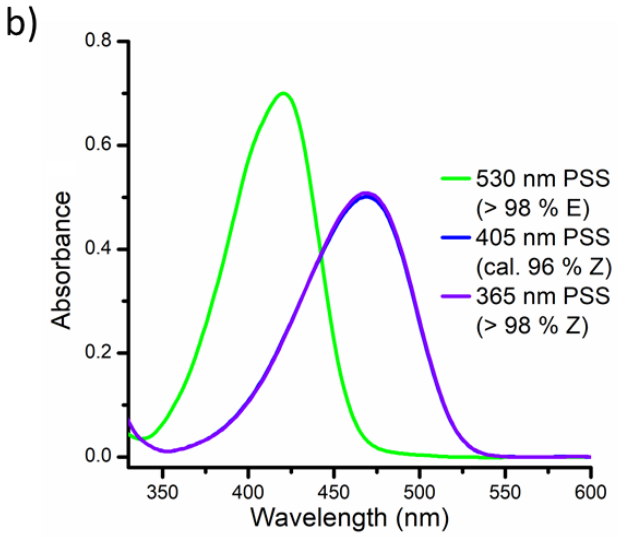

Fig. S4. a) ^{1}H NMR spectra of the PSSs of compound **1** (400 MHz, $CDCl_3$) after irradiation with 505 nm and 385 nm LED light. B) UV-Vis absorption spectra of the PSSs of **1** obtained by irradiations with 505 nm and 385 nm light in DCM.

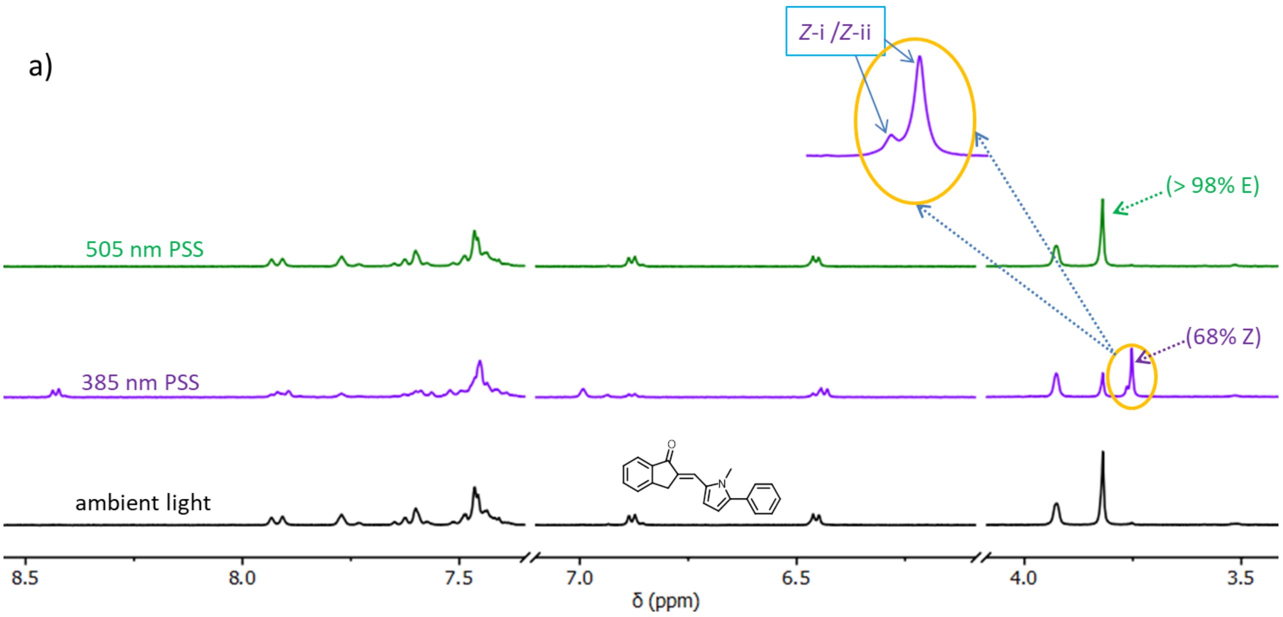

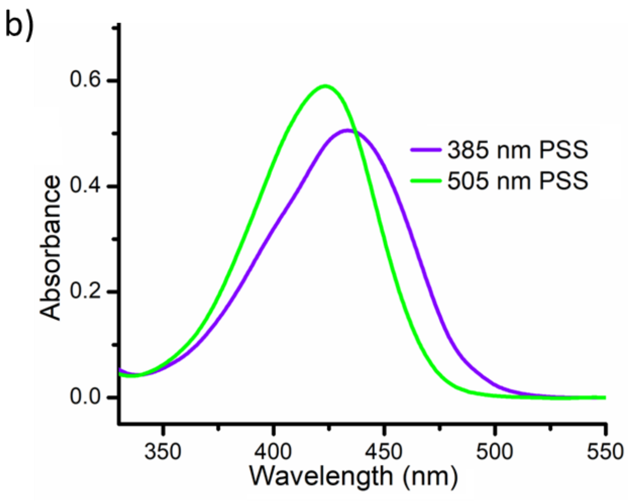

Fig. S5. a) ^{1}H NMR spectra of the PSSs of compound **2** (400 MHz, $CDCl_3$) after irradiation with 505 nm and 385 nm LED light. B) UV-Vis absorption spectra of the PSSs of **2** obtained by irradiations with 505 nm and 385 nm light in DCM.

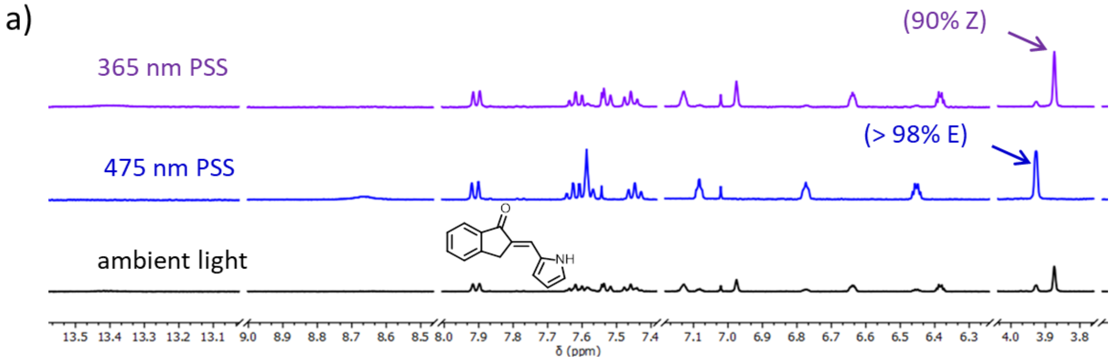

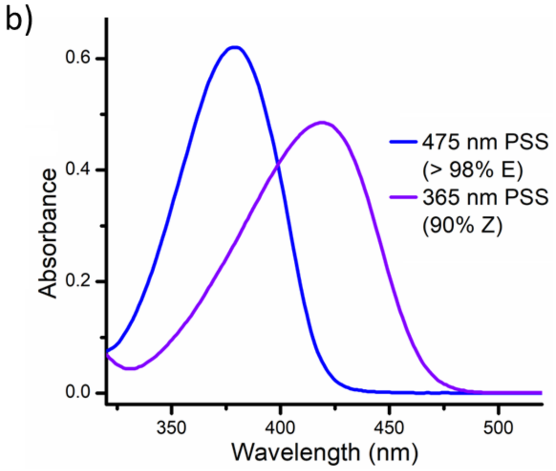

Fig. S6. a) ^{1}H NMR spectra of the PSSs of compound **3** (400 MHz, $CDCl_3$) after irradiation with 475 nm and 365 nm LED light. b) UV-Vis absorption spectra of the PSSs of **3** obtained by irradiations with 475 nm and 365 nm light in DCM.

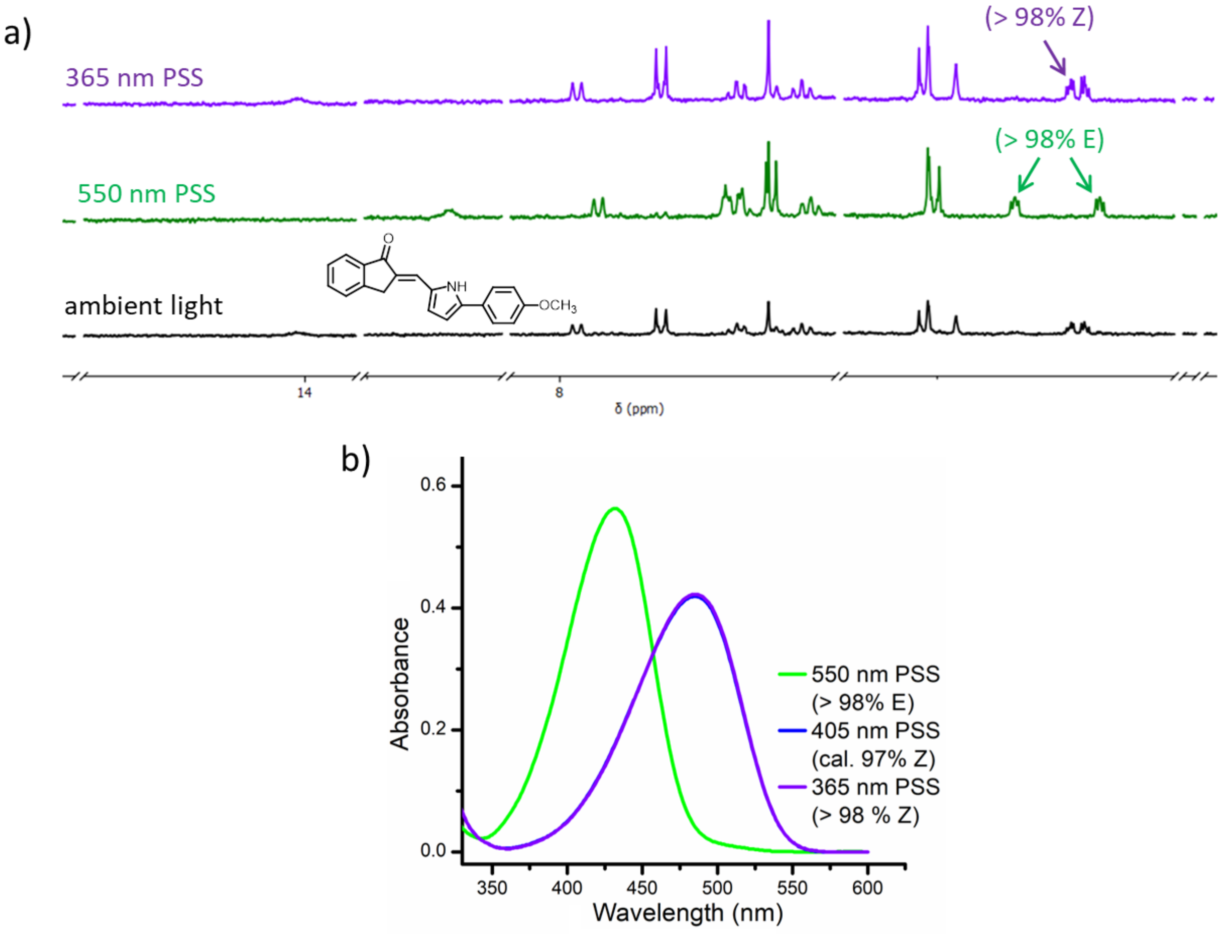

Fig. S7. a) ^{1}H NMR spectra of the PSSs of compound **4** (400 MHz, $CDCl_3$) after irradiation with 550 nm and 365 nm LED light. b) UV-Vis absorption spectra of the PSSs of **4** obtained by irradiations with 550 nm and 365 nm light in DCM.

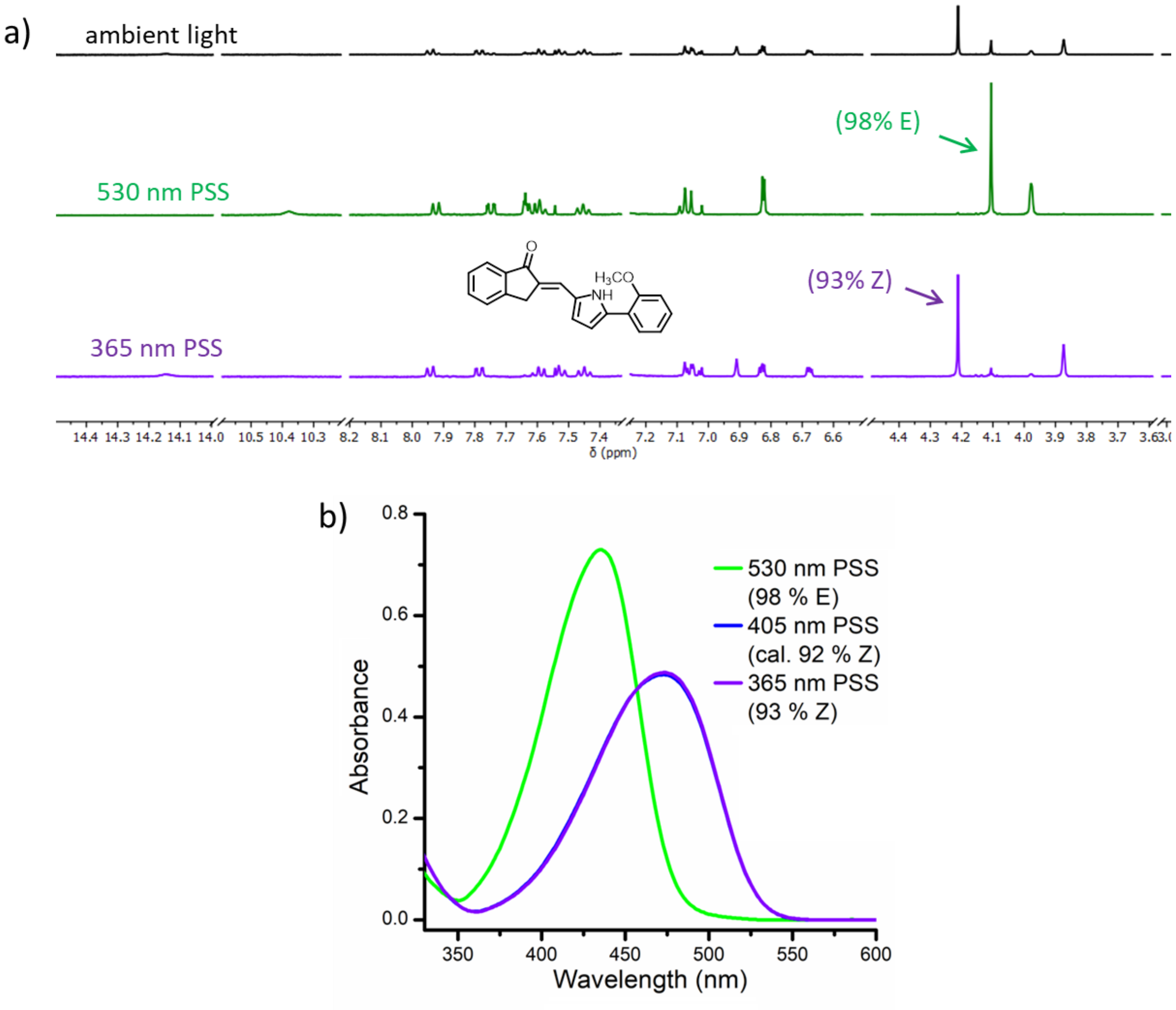

Fig. S8. a) ^{1}H NMR spectra of the PSSs of compound **5** (400 MHz, $CDCl_3$) after irradiation with 530 nm and 365 nm LED light. b) UV-Vis absorption spectra of the PSSs of **5** obtained by irradiations with 530 nm and 365 nm light in DCM.

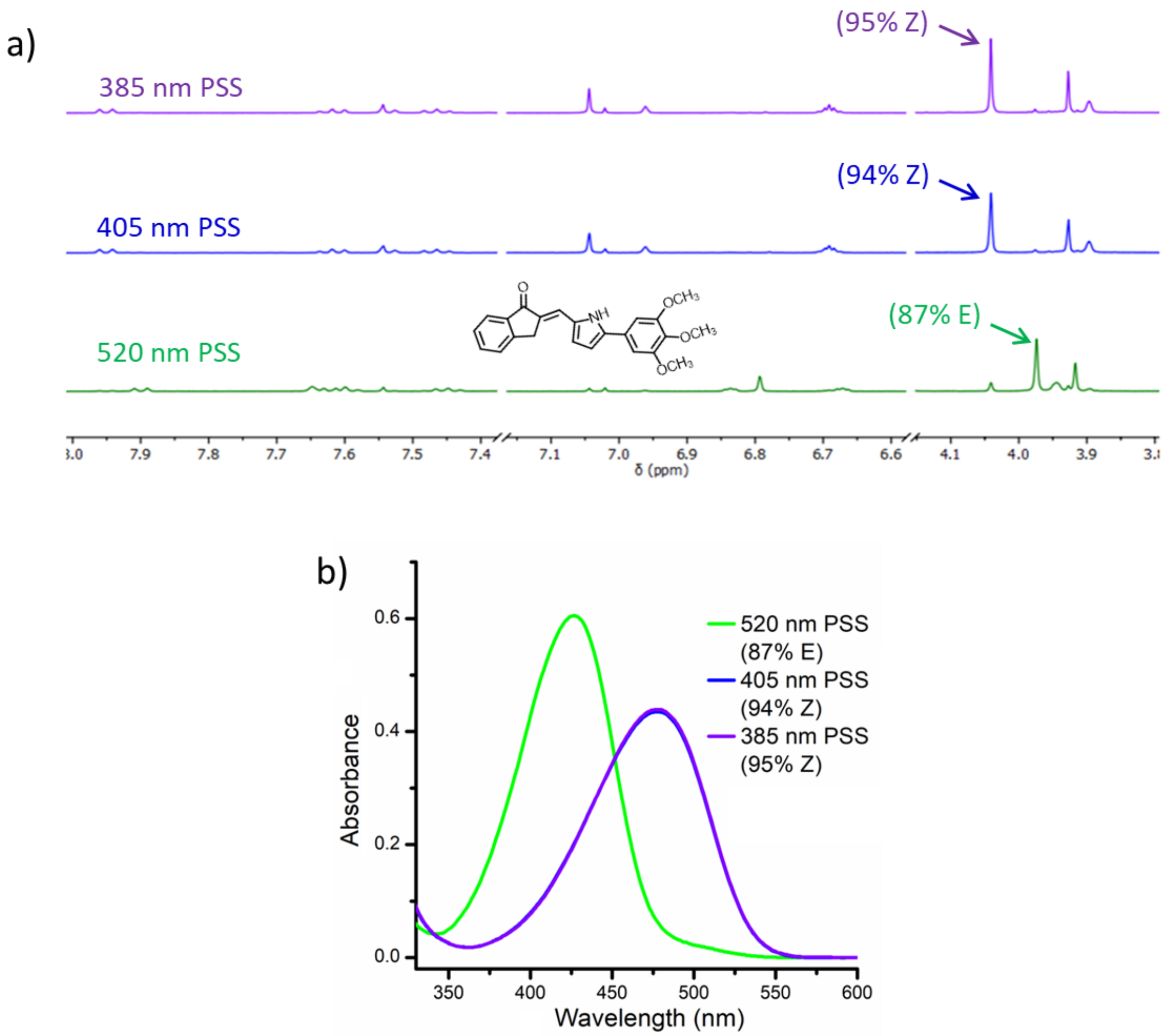

Fig. S9. a) ^{1}H NMR spectra of the PSSs of compound **6** (400 MHz, $CDCl_3$) after irradiation with 520 nm, 405 nm and 385 nm LED light. b) UV-Vis absorption spectra of the PSSs of **6** obtained by irradiations with 520 nm, 405 nm and 385 nm light in DCM.

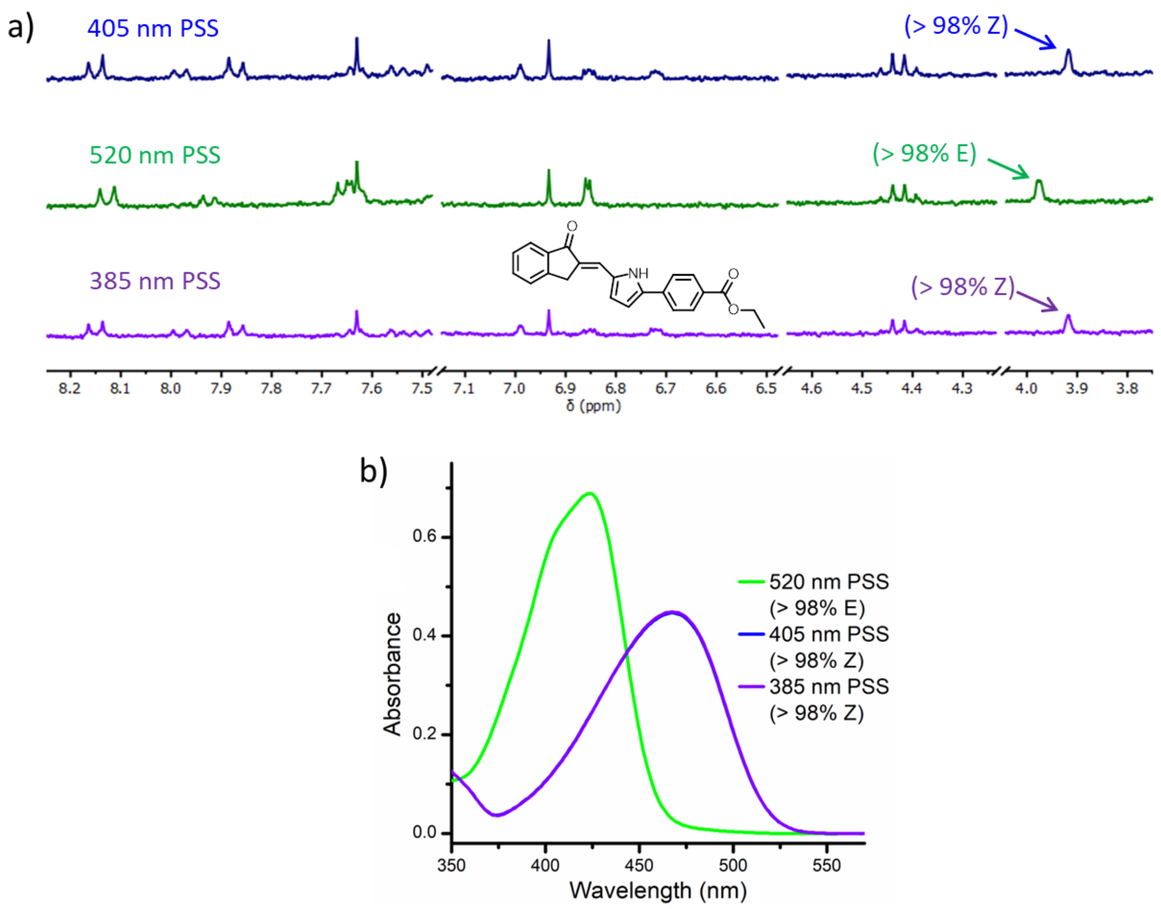

Fig. S10. a) ^{1}H NMR spectra of the PSSs of compound **7** (400 MHz, $CDCl_3$) after irradiation with 520 nm, 405 nm and 385 nm LED light. b) UV-Vis absorption spectra of the PSSs of **7** obtained by irradiations with 520 nm, 405 nm and 385 nm light in DCM.

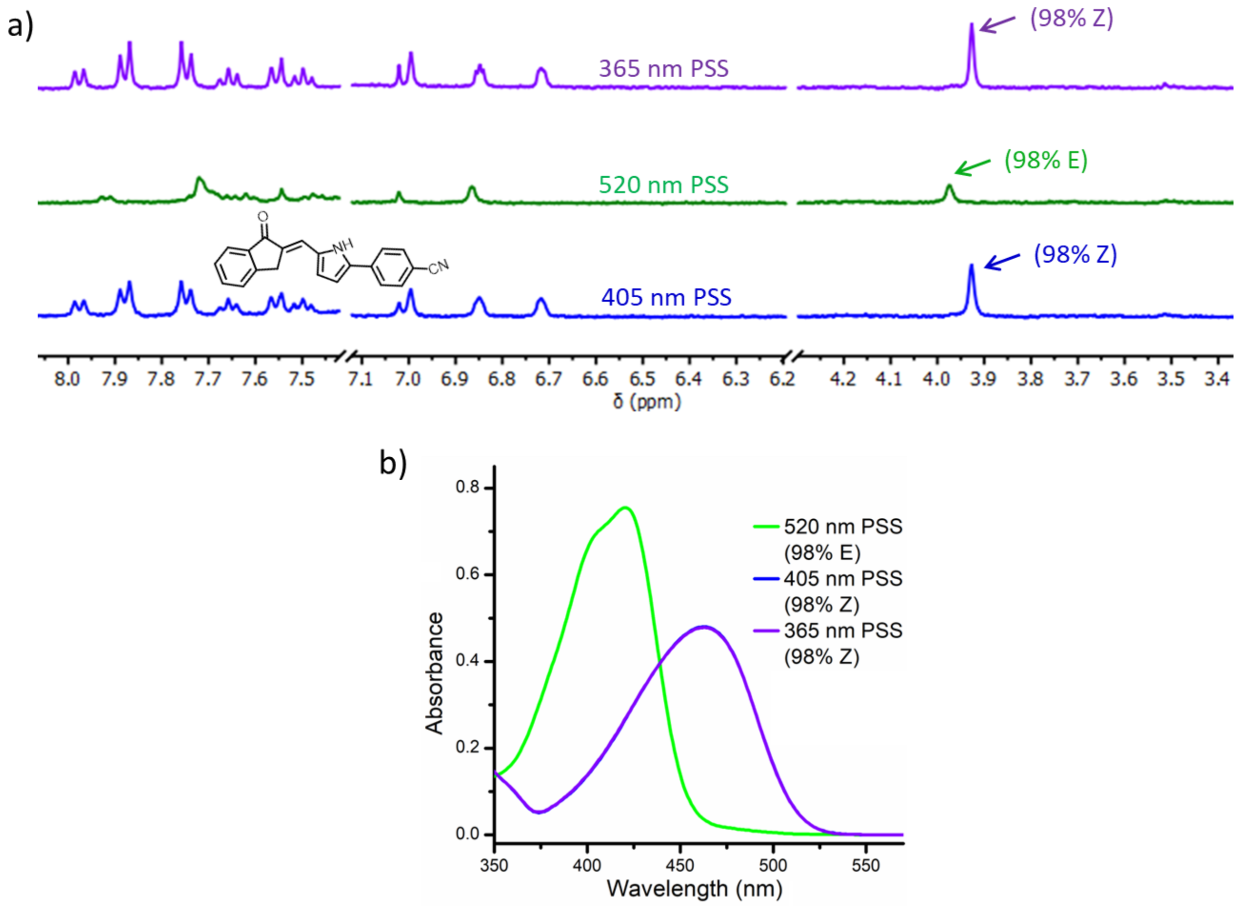

Fig. S11. a) ^{1}H NMR spectra of the PSSs of compound **8** (400 MHz, $CDCl_3$) after irradiation with 520 nm, 405 nm and 365 nm LED light. b) UV-Vis absorption spectra of the PSSs of **8** obtained by irradiations with 520 nm, 405 nm and 365 nm light in DCM.

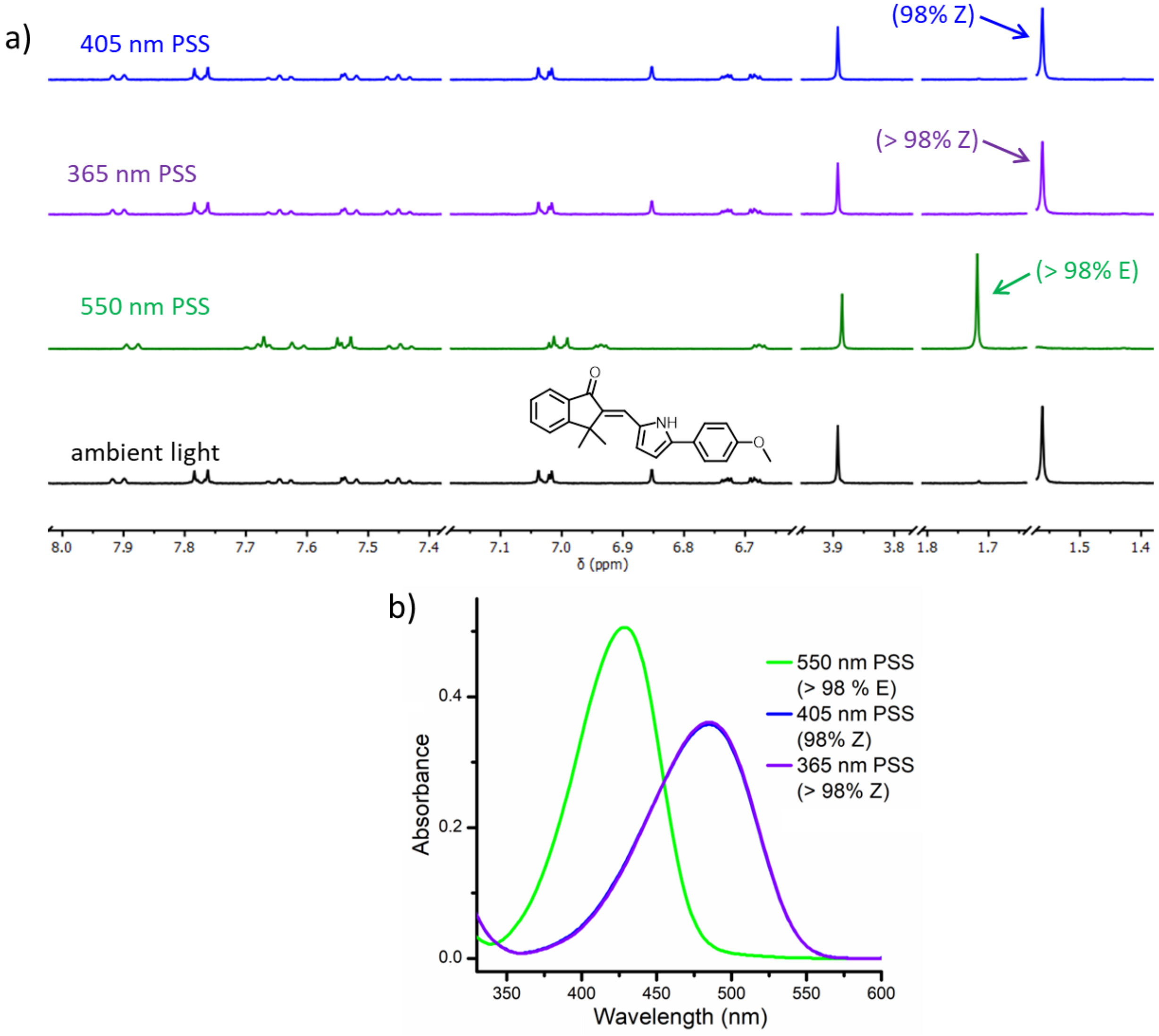

Fig. S12. a) ^{1}H NMR spectra of the PSSs of compound **9** (400 MHz, $CDCl_3$) after irradiation with 550 nm, 405 nm and 365 nm LED light. b) UV-Vis absorption spectra of the PSSs of **8** obtained by irradiations with 550 nm, 405 nm and 365 nm light in DCM.

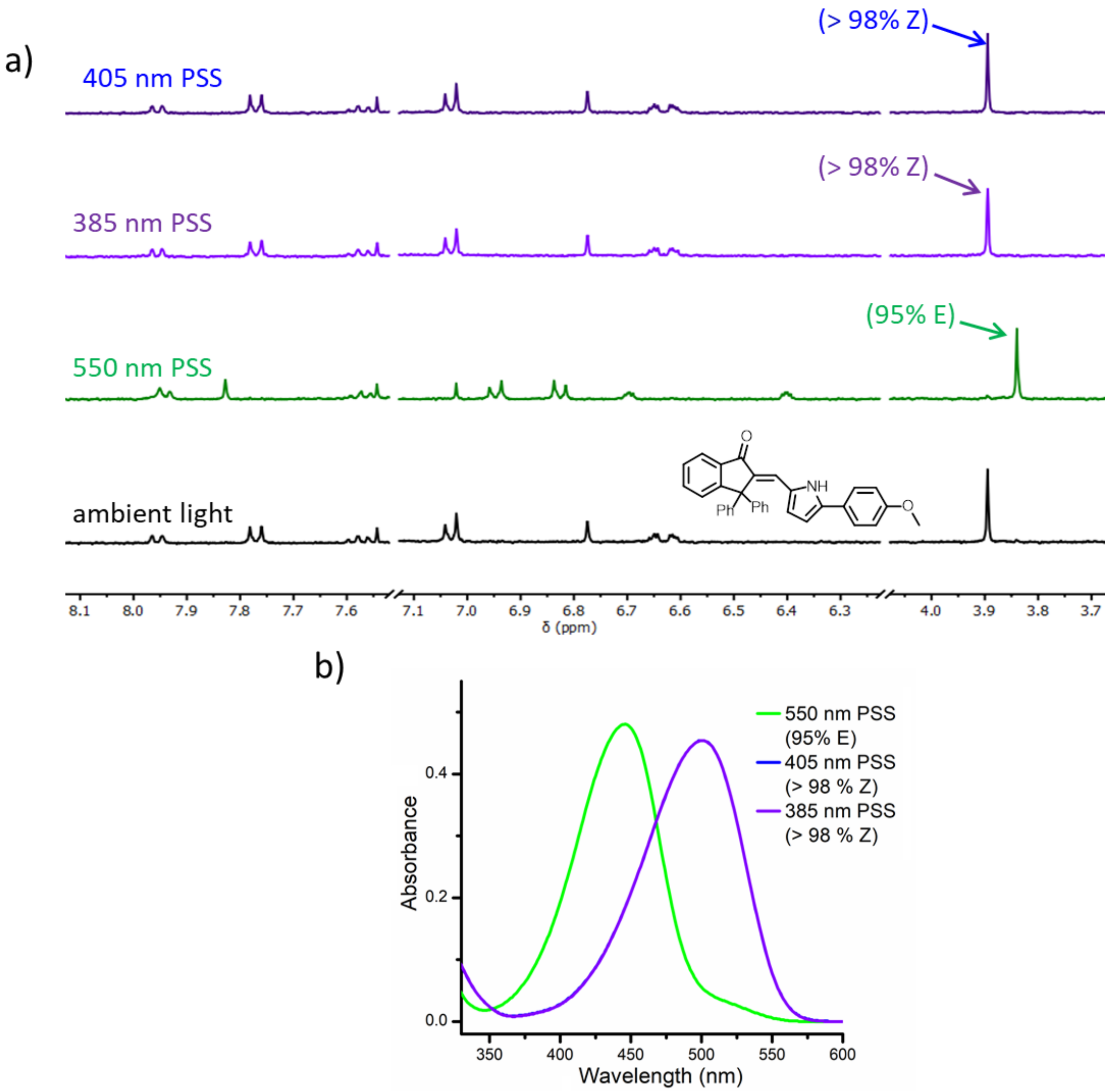

Fig. S13. a) ^{1}H NMR spectra of the PSSs of compound **10** (400 MHz, $CDCl_3$) after irradiation with 550 nm, 405 nm and 385 nm LED light. b) UV-Vis absorption spectra of the PSSs of **10** obtained by irradiations with 550 nm, 405 nm and 385 nm light in DCM.

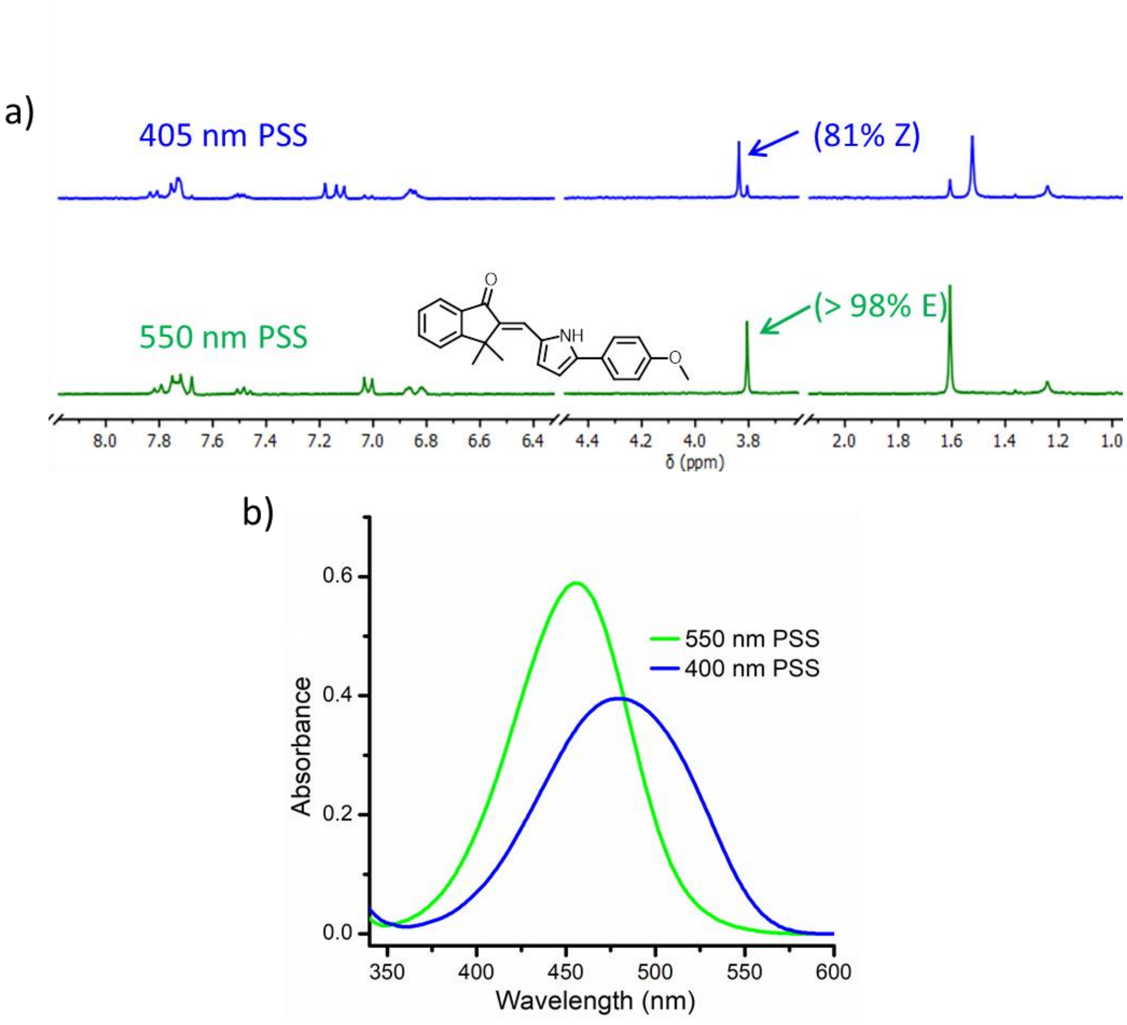

Fig. S14. a) ^{1}H NMR spectra of the PSSs of compound **9** (400 MHz, dmso-d_6) after irradiation with 550 nm and 405 nm LED light. b) UV-Vis absorption spectra of the PSSs of **9** obtained by irradiations with 550 nm and 405 nm light in 50% DMSO-PBS buffer solution.

3.4. Photobleaching experiment:

The 15-30 µM sample solutions in dichloromethane were irradiated alternately with light of wavelengths 365 nm or 405 nm and 520 nm, or 475 nm for multiple cycles and the changes in absorption were measured in a diod-array UV-Vis spectrophotometer.

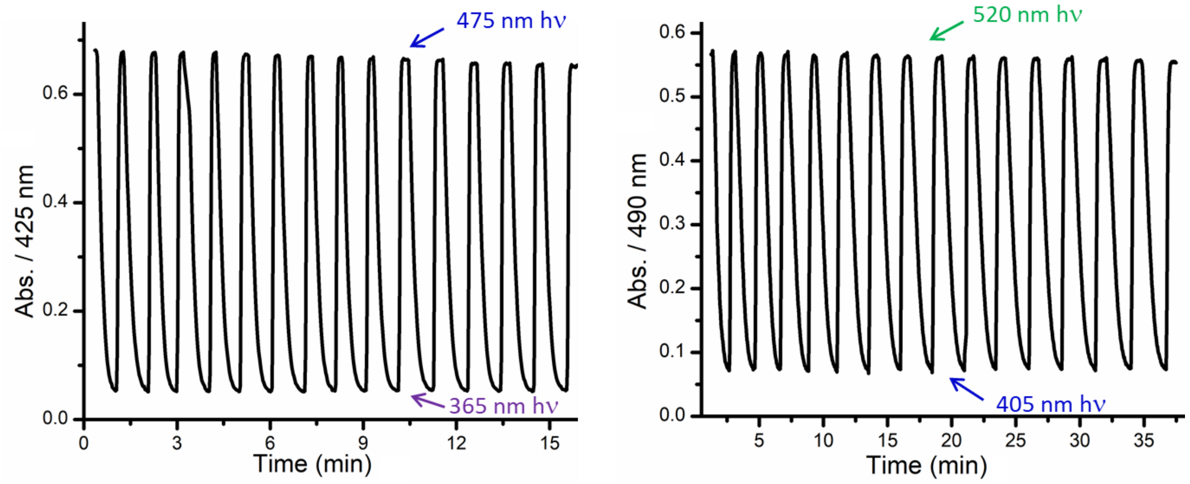

Fig. S15. Photobleaching of compounds **3** (left) and **4** (right) in dichloromethane solvent.

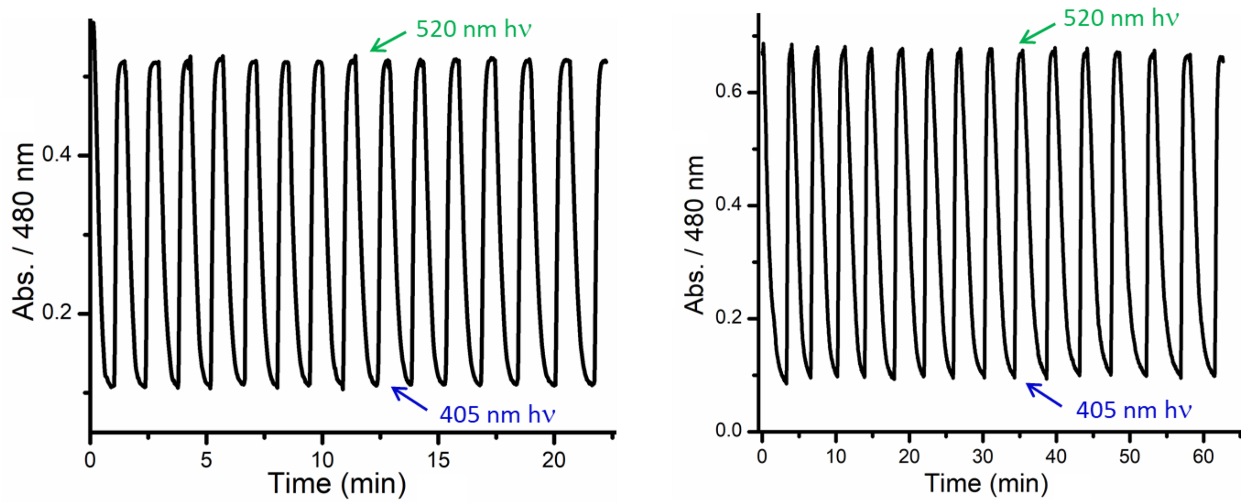

Fig. S16. Photobleaching of compounds **5** (left) and **6** (right) in dichloromethane.

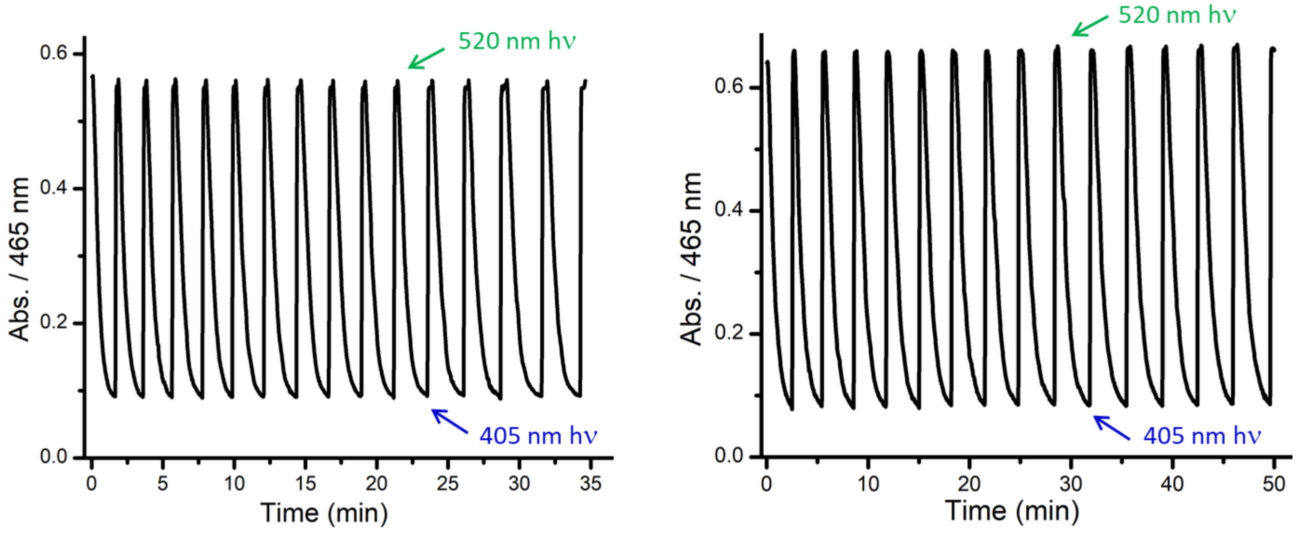

Fig. S17. Photobleaching of compounds **7** (left) and **8** (right) in dichloromethane.

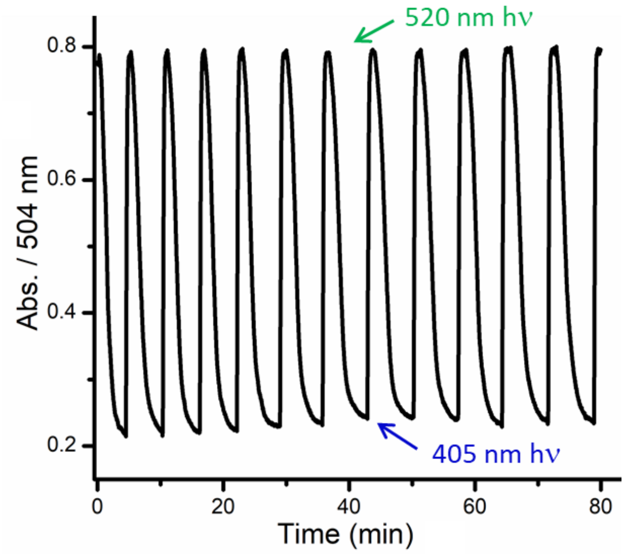

Fig. S18. Photobleaching of compound **10** in dichloromethane.

3.5. Determination of thermal half-lives of *E*-isomers:

The E-enriched PSSs of compounds **1** and **4**-**6** in toluene were heated at 80^0 C for 12-48 hours while acquiring there absorption spectra at a regular interval of 2 or 12 hours. For compounds **1** and **4**-**6**, negligible changes in the absorption were noticed, indicating extremely high and comparable thermal stability of both isomers. Since changes in absorption were noticed for compounds **9** and **10**, absorptions at 430 nm (for **9**) and 445 nm (for **10**) were plotted as a function of time. These curves were nicely fitted with the mono-exponential function, which led to the estimation of *E* half-lives.

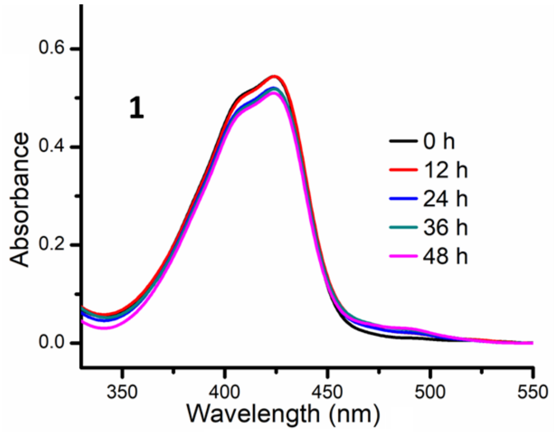

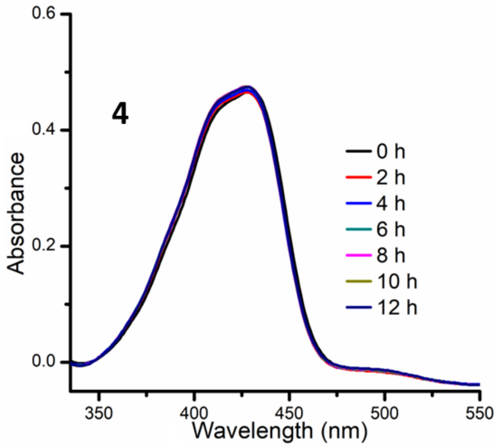

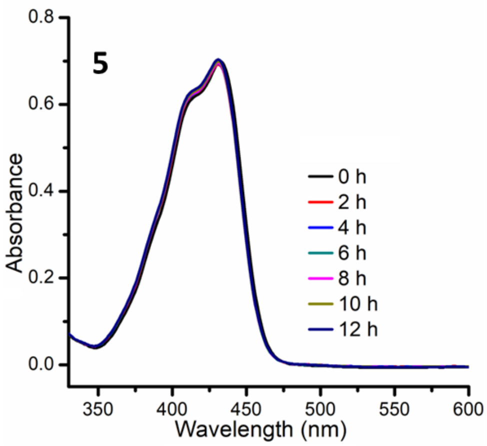

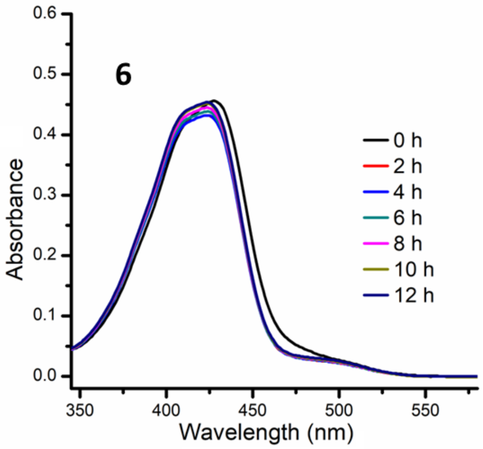

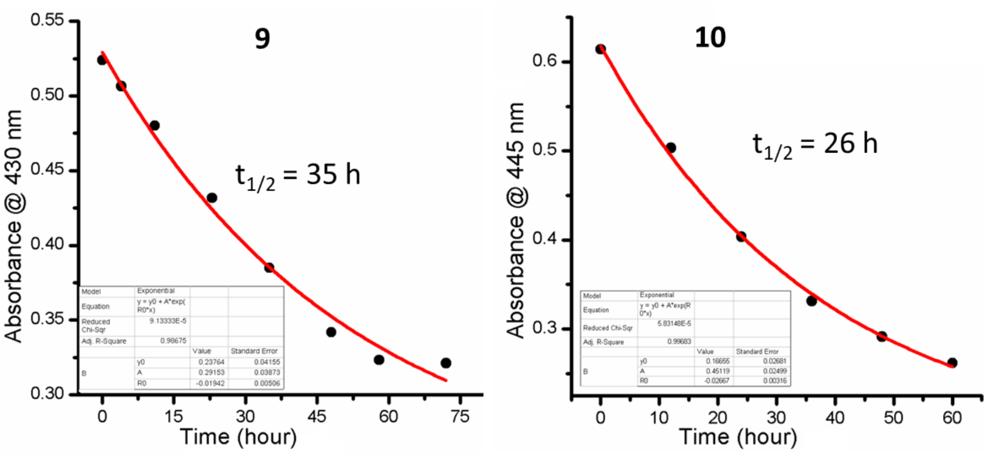

Fig. S19. Thermal *E*-to-*Z* relaxation rates for **9** and **10** at 80^0 C in toluene solvent.

3.6. The photoisomerisation quantum yield estimation:

The quantum yields for the *Z-E* and the *E-Z* isomerisations of switches **1** and **4-10** were determined by following the method reported by G. Gescheidt et al.[7] The change in the absorbance spectra of ca. 0.005-0.01 mM solutions of **1** and **4-10** in DCM upon 520 nm light irradiation was monitored until the photostationary states arrived. Then, the above samples were back irradiated by 385 nm light and the absorption spectra were recorded until reached the PSSs. The absorbance at respective λ_{max} of the absorption spectra was plotted against the irradiation time for **1** and **4-10**, and curves were fitted to a single exponential function to yield the rate constants. The intensities of the 520 nm and 385 nm LEDs were measured subsequently. Then, by using the resulting rate constants, irradiation light intensity and sample concentrations, we determined the quantum yields using the equation below:

$$\Phi = k_{fit}\, C_0 / I_0\, (1\text{-}10^{-A_i})$$

Φ = quantum yield
A'_i = the initial absorbance at the irradiation wavelength
C_0 = the concentrations of the stock solutions
I_0 = photon flux
K_{fit} = rate constant of reaction.

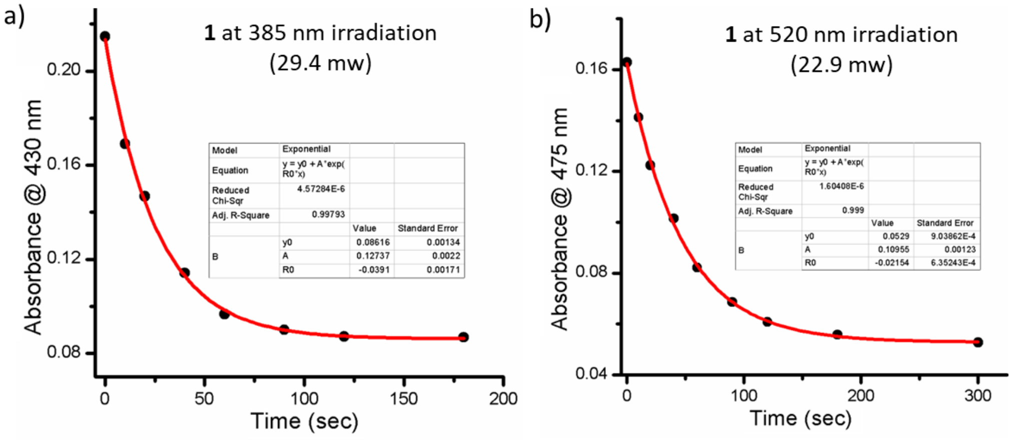

Fig. S20. Kinetics of the photoisomerisation for comp. **1** (0.006 mM) used for quantum yield calculation.

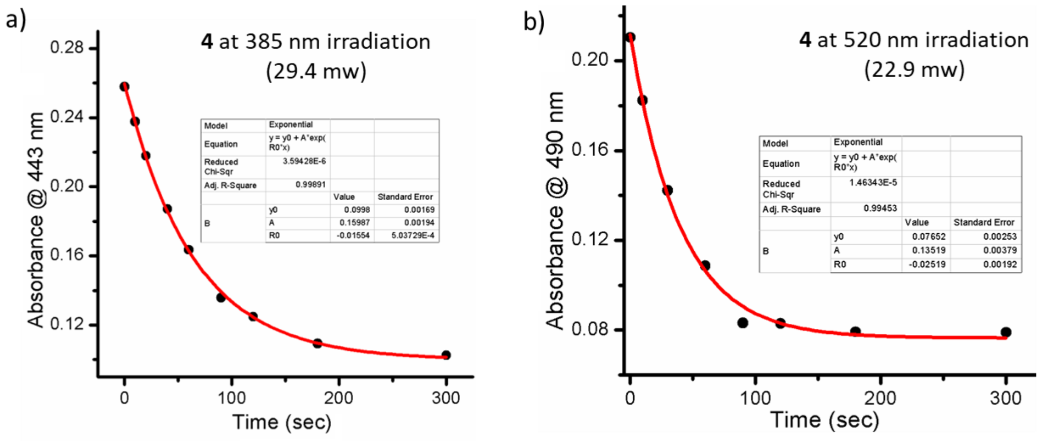

Fig. S21. Kinetics of the photoisomerisation for comp. **4** (0.009 mM) used for quantum yield calculation.

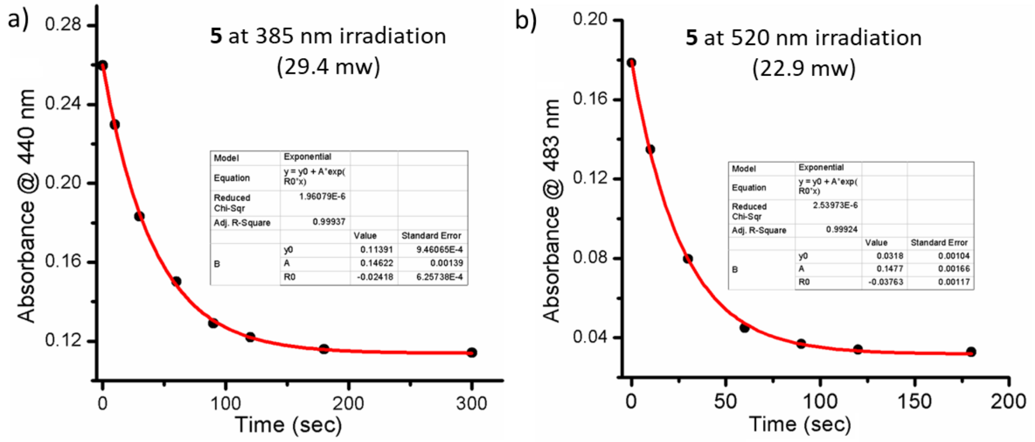

Fig. S22. Kinetics of the photoisomerisation for comp. **5** (0.0068 mM) used for quantum yield calculation.

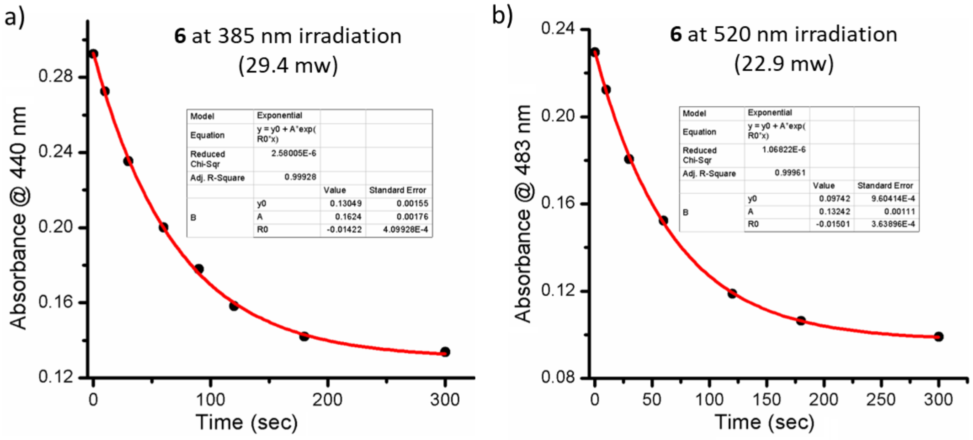

Fig. S23. Kinetics of the photoisomerisation for comp. **6** (0.006 mM) used for quantum yield calculation.

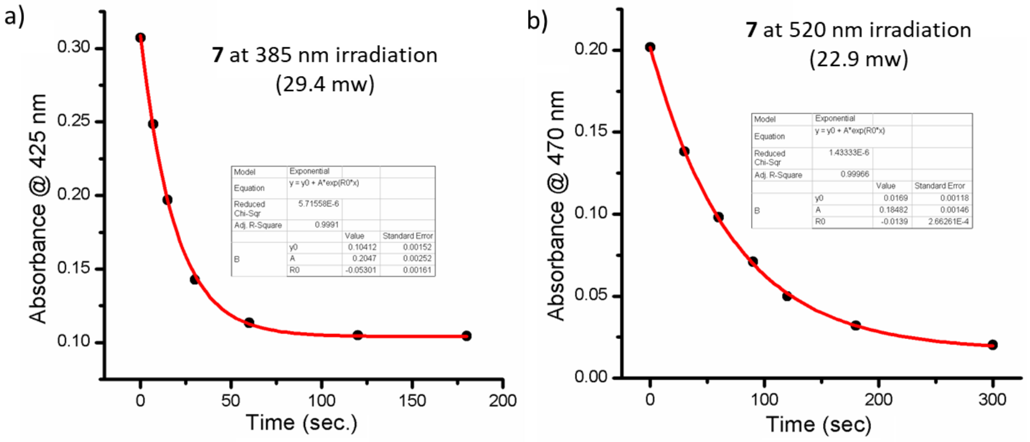

Fig. S24. Kinetics of the photoisomerisation for comp. **7** (0.006 mM) used for quantum yield calculation.

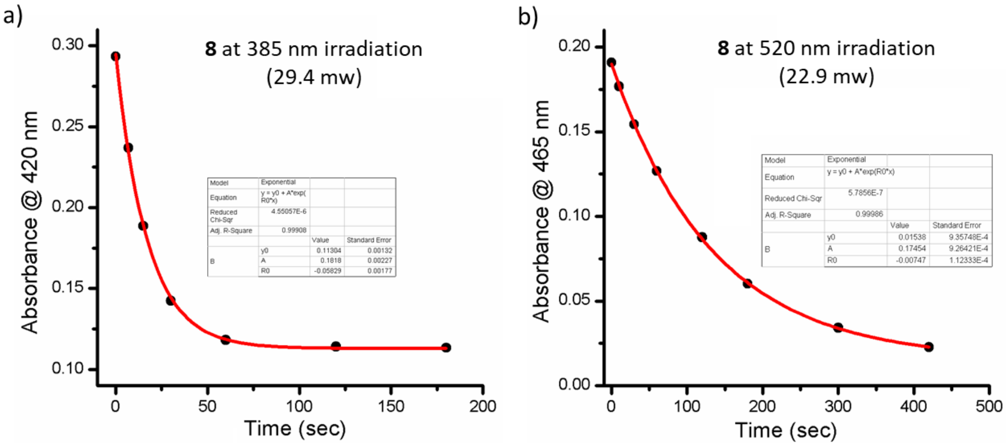

Fig. S25. Kinetics of the photoisomerisation for comp. **8** (0.005 mM) used for quantum yield calculation.

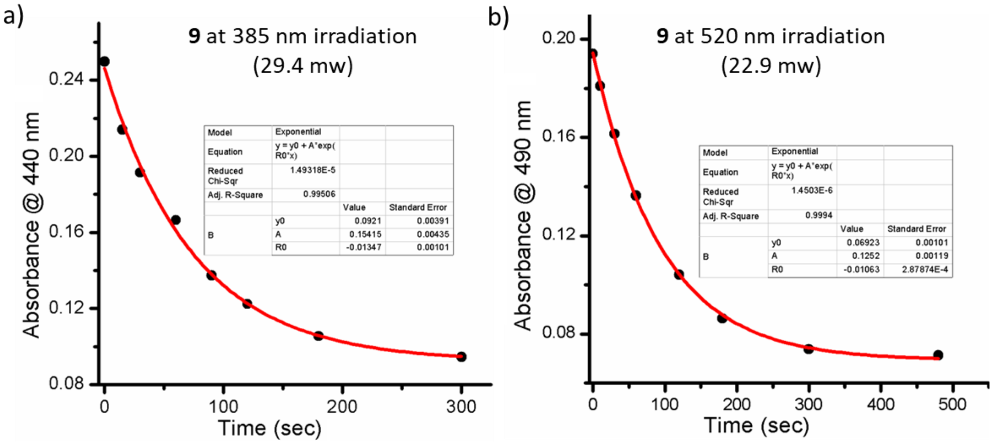

Fig. S26. Kinetics of the photoisomerisation for comp. **9** (0.008 mM) used for quantum yield calculation.

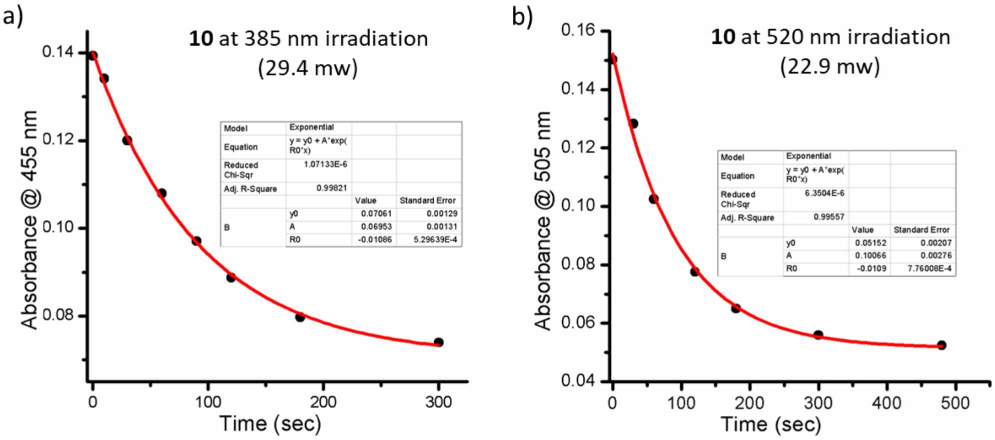

Fig. S27. Kinetics of the photoisomerisation for comp. **10** (0.006 mM) used for quantum yield calculation

4. Fluorescence emission properties:

4.1. General method: Fluorescence emissions were acquired on a Horiba Fluoromax-Plus-c fluorimeter spectrophotometer in a quartz cell with 1.0 cm pathlength. Slit width of 1.5 nm bandpass was selected for both excitation and emission beams. Solvent background spectra were subtracted from the spectra of samples. Sample solutions were prepared by dilutions from the stock solutions. The final concentration of the solutions prepared for fluorescence experiments was ca. 7-12 µM. The compounds were excited at their absorption maxima (λ_{max}), determined using UV/Vis absorption measurements.

4.2. Fluorescence spectra of *E*-and *Z*-isomers:

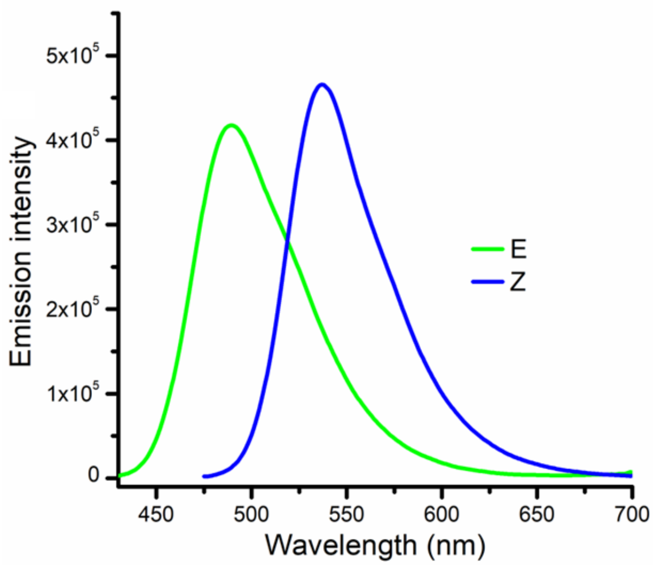

Fig. S28. Fluorescence emission spectra of Z-isomer (λ_{ex} = 470 nm) and *E*-isomer (λ_{ex} = 420 nm) of **1** (10 µM) in dichloromethane under aerobic conditions at room temperature.

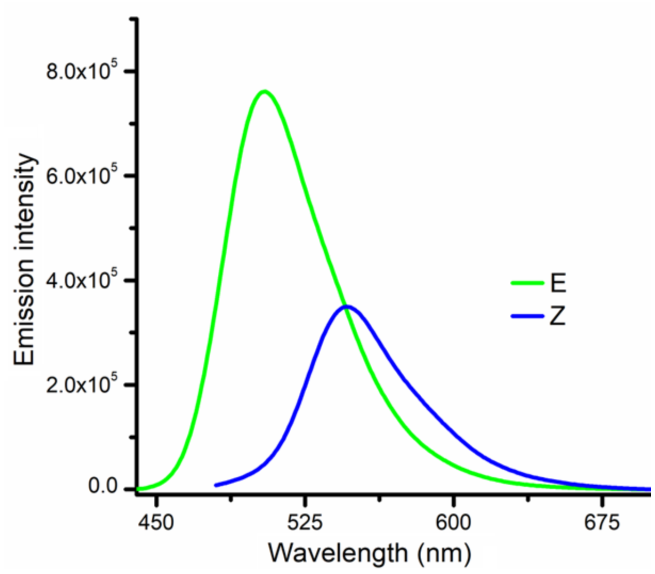

Fig. S29. Fluorescence emission spectra of Z-isomer (λ_{ex} = 475 nm) and *E*-isomer (λ_{ex} = 435 nm) of **5** (10 µM) in dichloromethane under aerobic conditions at room temperature.

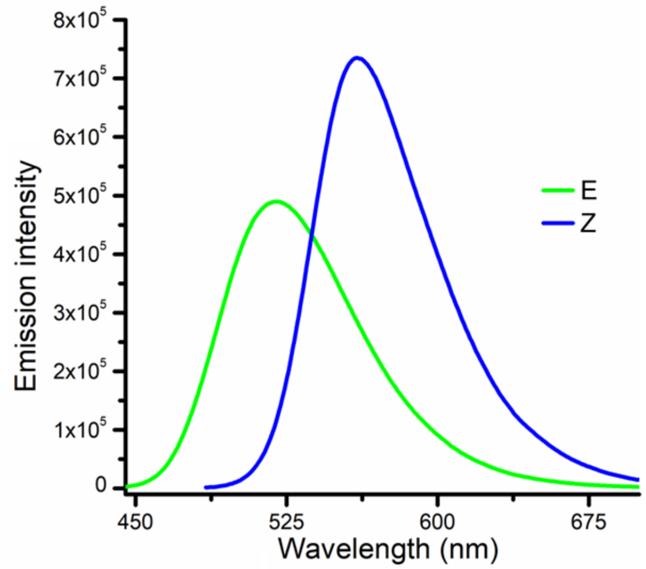

Fig. S30. . Fluorescence emission spectra of Z-isomer (λ_{ex} = 475 nm) and *E*-isomer (λ_{ex} = 425 nm) of **6** (12 µM) in dichloromethane under aerobic conditions at room temperature.

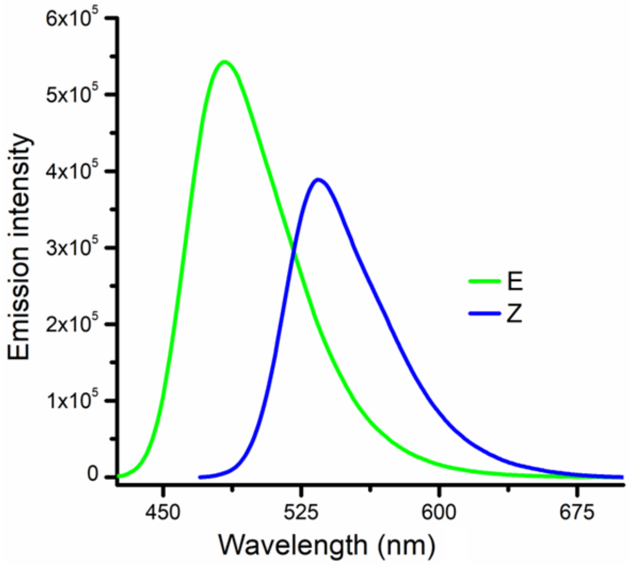

Fig. S31. . Fluorescence emission spectra of Z-isomer (λ_{ex} = 465 nm) and *E*-isomer (λ_{ex} = 425 nm) of **7** (7 µM) in dichloromethane under aerobic conditions at room temperature.

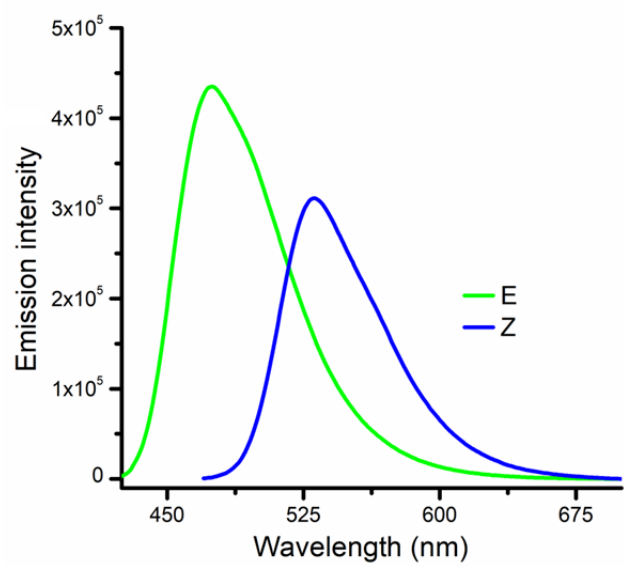

Fig. S32. . Fluorescence emission spectra of Z-isomer (λ_{ex} = 465 nm) and *E*-isomer (λ_{ex} = 420 nm) of **8** (7 µM) in dichloromethane under aerobic conditions at room temperature.

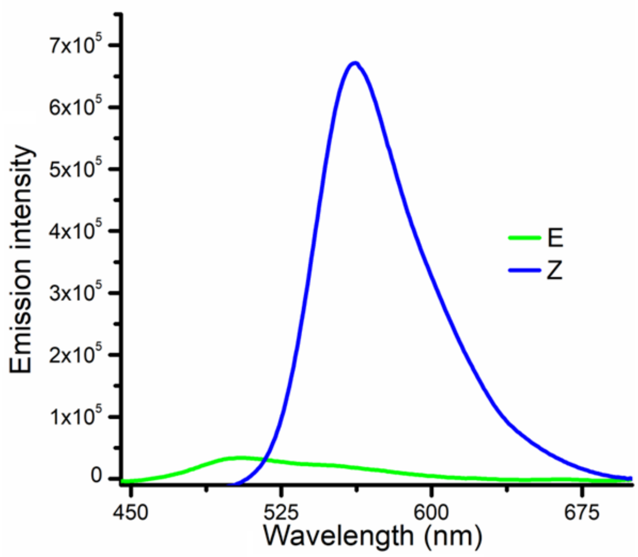

Fig. S33. . Fluorescence emission spectra of Z-isomer (λ_{ex} = 485 nm) and *E*-isomer (λ_{ex} = 430 nm) of **9** (10 µM) in dichloromethane under aerobic conditions at room temperature.

4.3. Fluorescence switching cycles:

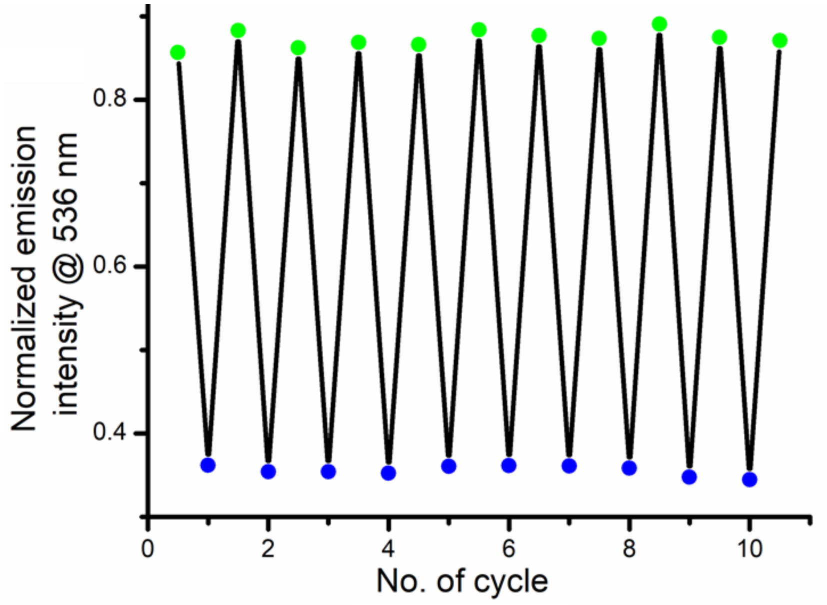

Fig. S34. The fluorescence switching cycles of switch **1** in dichloromethane. The fluorescence intensity change at λ = 536 nm was monitored using a fluorimeter while altering the irradiation wavelengths between 520 and 405 nm.

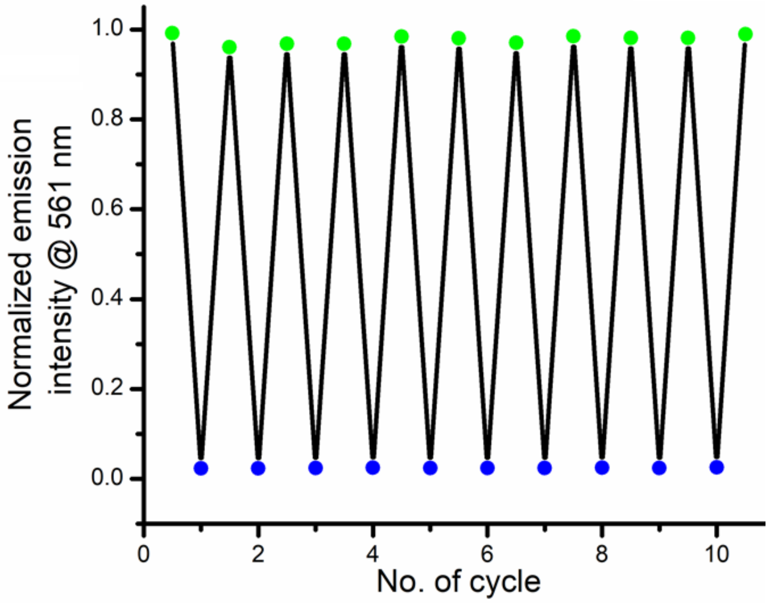

Fig. S35. The fluorescence switching cycles of switch **9** in dichloromethane. The fluorescence intensity change at λ = 561 nm was monitored using a fluorimeter while alternating the irradiation wavelength between 520 and 405 nm.

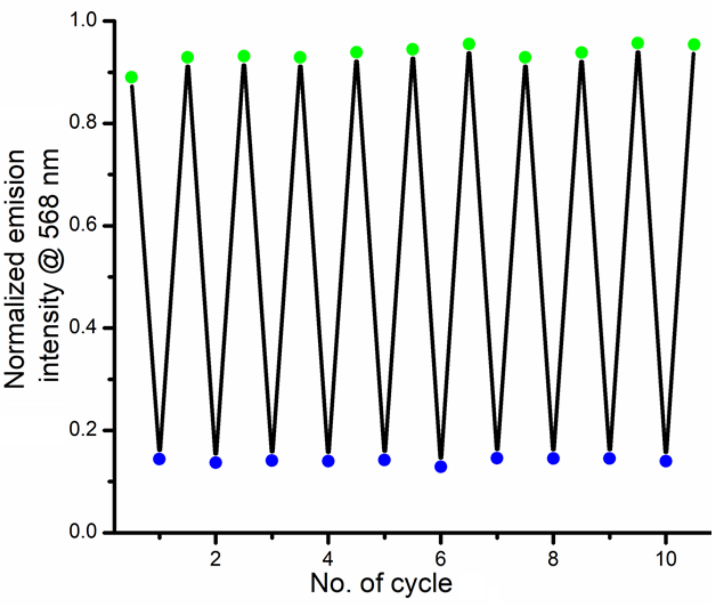

Fig. S36. The fluorescence switching cycles of switch **10** in dichloromethane. The fluorescence intensity change at λ = 568 nm was monitored using a fluorimeter while alternating the irradiation wavelength between 520 and 405 nm.

4.4. Determination of the fluorescence quantum yield ϕ_S

All compounds were dissolved in DCM. Fluorescein dissolved in 0.1M NaOH aqueous solution was used as the reference dye. The solutions were diluted such that the absorbance was <0.1.The fluorescence quantum yield was calculated according to Equation 1.

$$\phi_S = \phi_R \frac{A_R}{A} \frac{F}{F_R} \frac{\eta^2}{{\eta_R}^2} \quad \ldots\ldots\ldots\ldots\ldots\ldots\ldots\ldots\ldots\ldots\ldots\ldots\ldots\ldots (1)$$

ϕ_R: fluorescence quantum yield of the reference dye

F, F_R: integration of the emission spectra of the sample and the reference dye, respectively

A, A_R: absorbance of the sample and the reference dye, respectively

η, η_R: refractive index of the sample and the reference solution, respectively

4.5. Fluorescence response to solvent viscosity

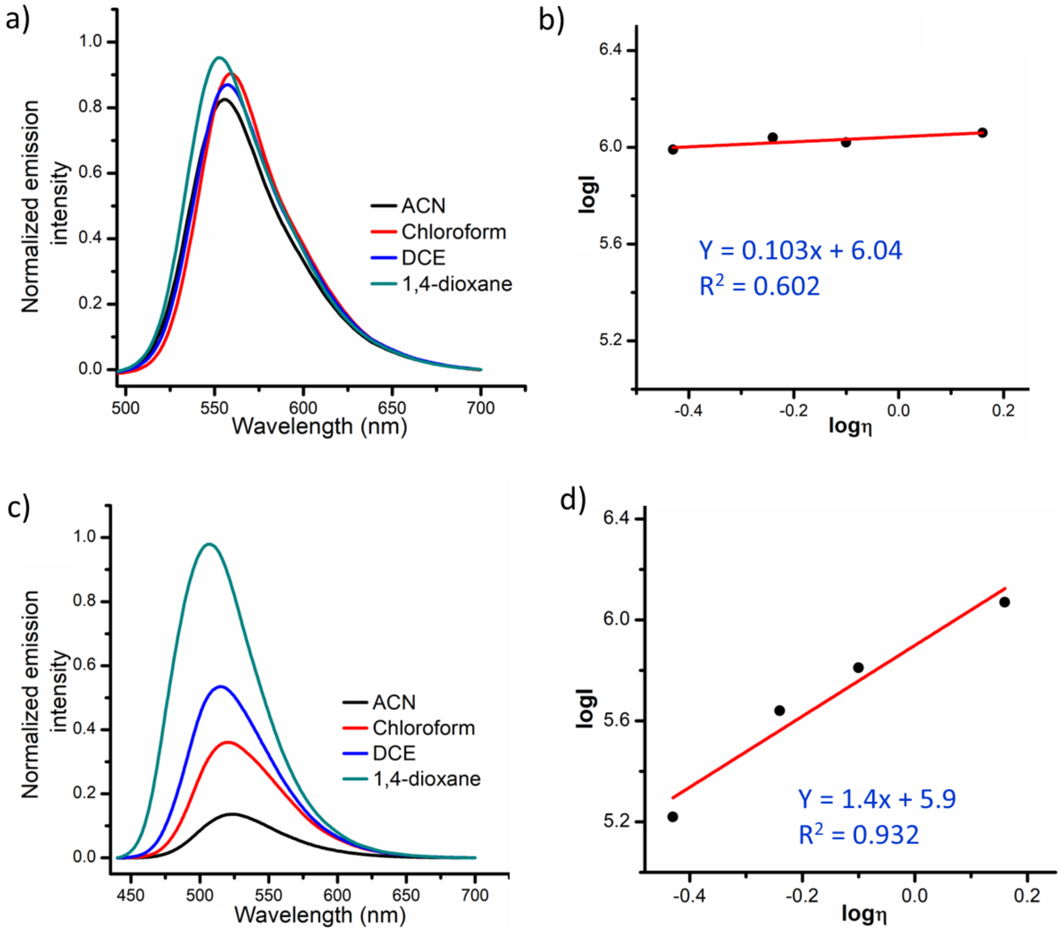

Fig. S37. Variation in the normalized fluorescence spectra of a) **4**-Z, and c) **4**-E (10 μM) in several viscous organic solvents (sample excitations at 490 nm for Z and 430 nm for E). Log-log plots of fluorescence intensity (I) of b) **4**-*Z,* and d) **4**-*E* as a function of solvent viscosity (η).

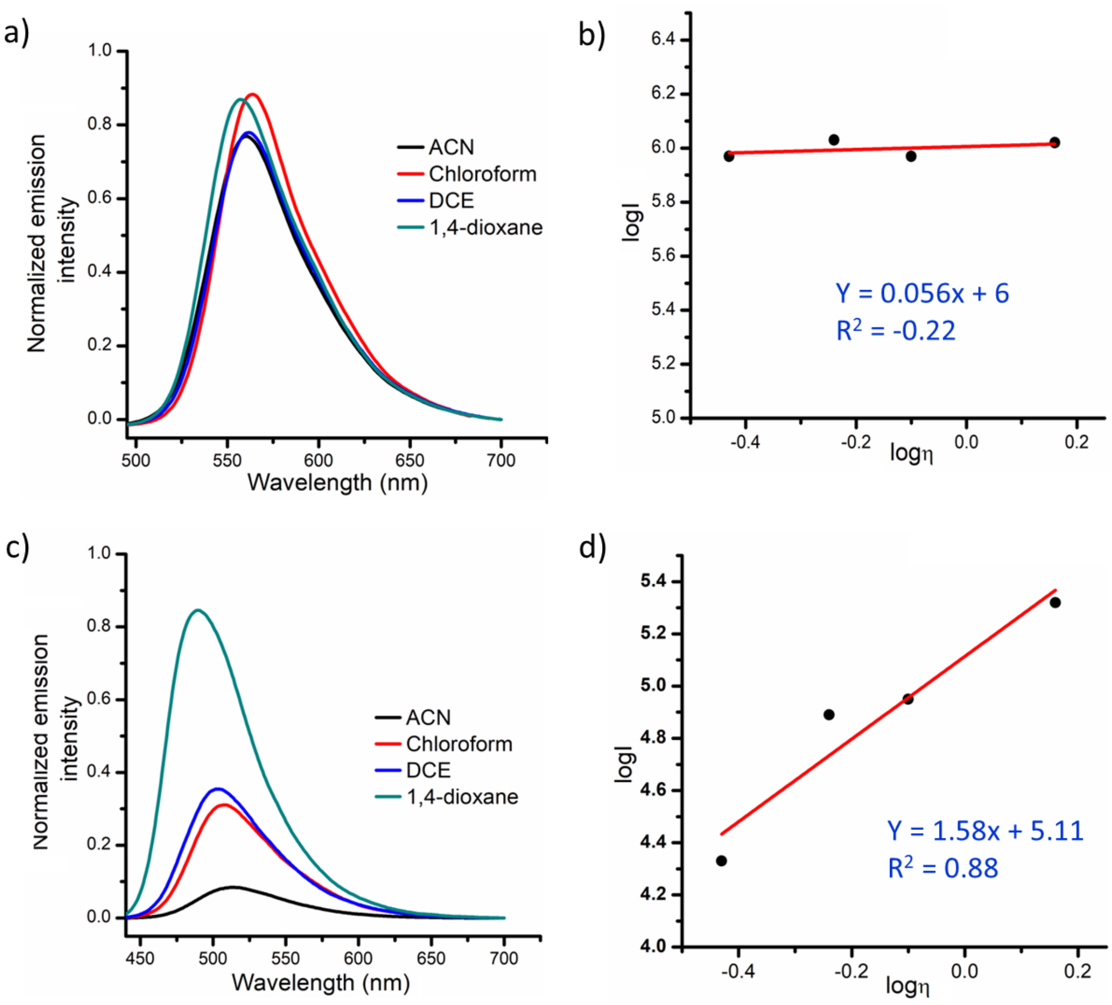

Fig. S38. Variation in the normalized fluorescence spectra of a) **9**-*Z*, and c) **9**-*E* (10 μM) in several viscous organic solvents (sample excitations at 490 nm for *Z* and 430 nm for *E*). Log-log plots of fluorescence intensity (I) of b) **9**-*Z,* and d) **9**-*E* as a function of solvent viscosity (η).

5. Computational Details:

Geometry optimization of all *Z*- and *E*-isomers and the transition states (TS) of thermal E→Z isomerisation were performed using density functional theory[8] with the nonlocal hybrid Becke three-parameter Lee-Yang-Parr (B3LYP)[9] functional and the 6-31G*[10] basis set. Gaussian 09 suit of program was used.[11] All calculations were carried out in gas phase. The TD-DFT (time-dependent density functional theory) calculations were carried out to obtain UV-Vis spectral data.[12] The 15 lowest-lying singlet excited states were considered for *Z*- and *E*-isomers at the B3LYP/6-31G* level of theory. The energy barrier of the C-C rotation (between pyrrole ring and alkene unit) in the ground state (S^o) and the first excited state (S^1) were calculated using same calculation method.

1-*Z*

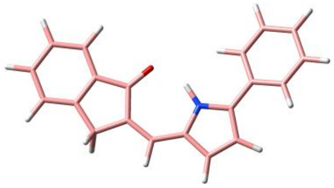

Relative E = 0.0 kcal/mol

H---O=C: 1.74 Å, 112.9°

C=C-C-C: -179.8 °

1-*E*-i

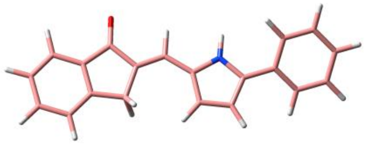

Relative E = 6.4 kcal/mol

C=C-C-C: -0.2 °

1-*E*-ii

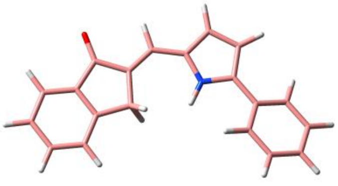

Relative E = 6.7 kcal/mol

C=C-C-C: -178.8 °

4-*Z*

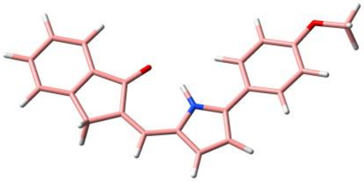

Relative E = 0.0 kcal/mol

H---O=C: 1.74 Å, 112.7°

C=C-C-C: -179.6 °

4-*E*-i

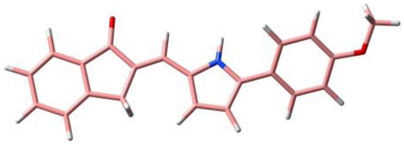

Relative E = 6.7 kcal/mol

C=C-C-C: -0.4 °

4-*E*-ii

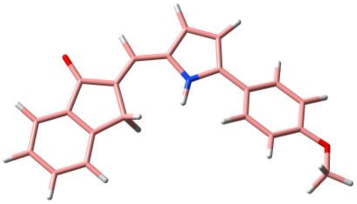

Relative E = 6.8 kcal/mol

C=C-C-C: -178.8 °

8-*Z*

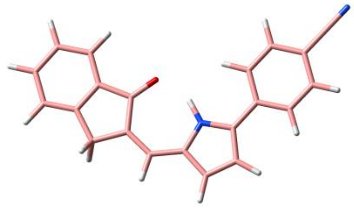

Relative E = 0.0 kcal/mol

H---O=C: 1.73 Å, 112.8^{o}

C=C-C-C: -180.0 ^{o}

8-*E*-i

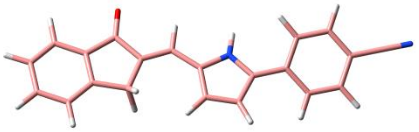

Relative E = 7.7 kcal/mol

C=C-C-C: -0.1 ^{o}

8-*E*-ii

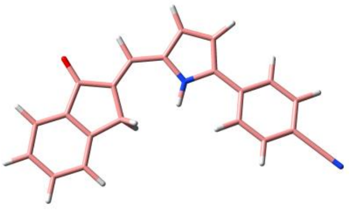

Relative E = 8.1 kcal/mol

C=C-C-C: -178.8^{o}

9-*Z*

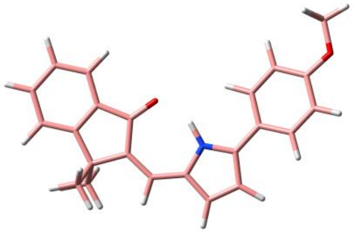

Relative E = 0.0 kcal/mol

H---O=C: 1.74 Å, 113.2^{o}

C=C-C-C: -179.7 ^{o}

9-*E*-i

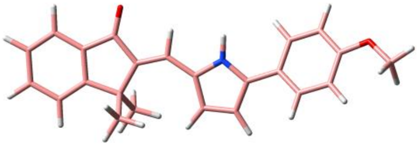

Relative E = 10.3 kcal/mol

C=C-C-C: 0.6^{o}

9-*E*-ii

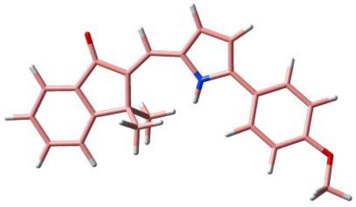

Relative E = 10.6 kcal/mol

C=C-C-C: -179.2 ^{o}

10-*Z*

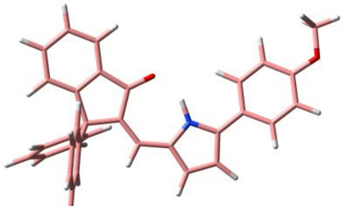

Relative E = 0.0 kcal/mol

H---O=C: 1.74 Å, 113.6^{o}

C=C-C-C: -179.7 ^{o}

10-*E*-i

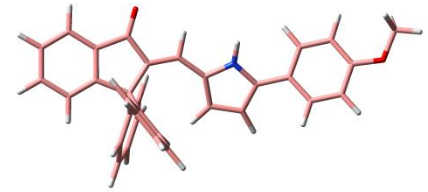

Relative E = 11.6 kcal/mol

C=C-C-C: 16.5 ^{o}

10-*E*-ii

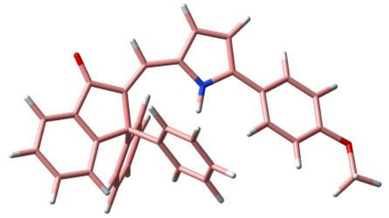

Relative E = 9.9 kcal/mol

C=C-C-C: -176.5 ^{o}

Figure S39: Geometry optimized structures of *E*-and *Z*-isomers of representative compounds **1, 4, 8, 9** and **10** in the gas phase.

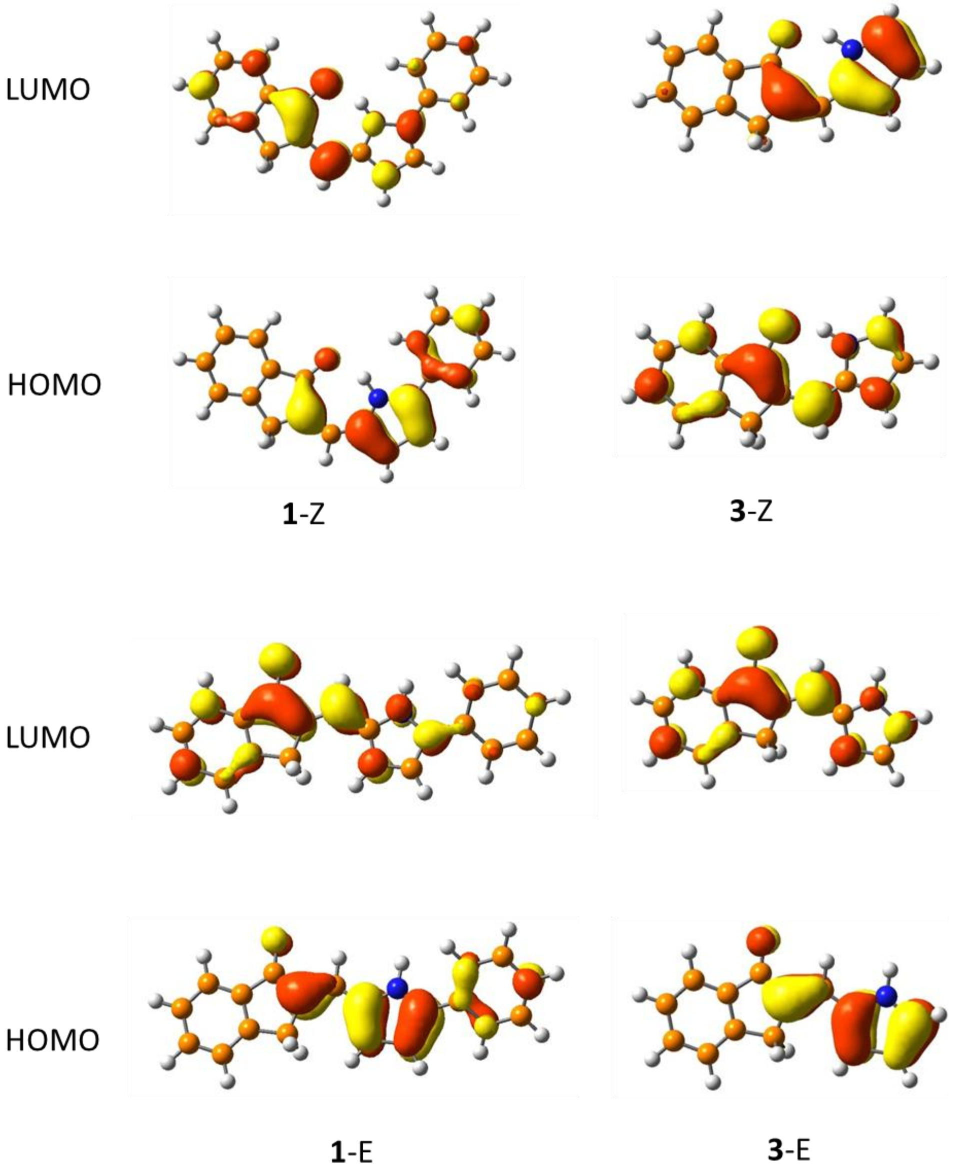

Fig. S40. Energetic diagram of frontier MOs of Z (top) and E (bottom) isomers of **1**, **3** and representations of LUMO (top) and HOMO (bottom).

LUMO

HOMO

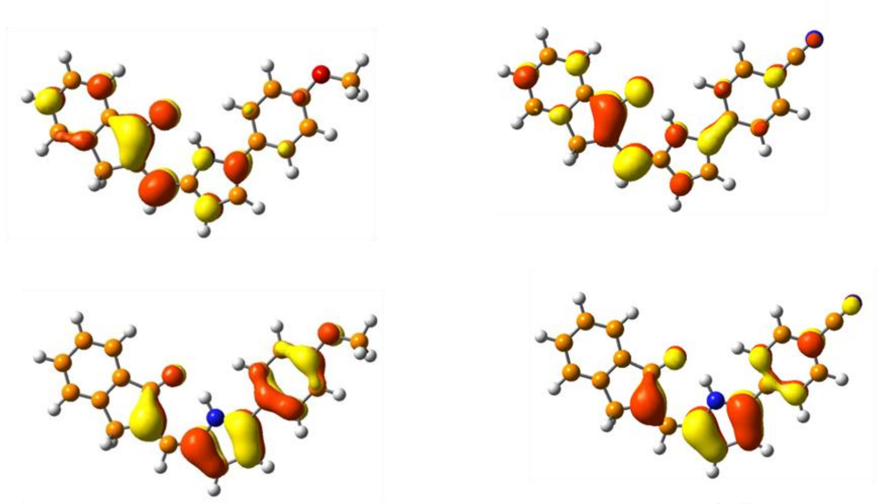

4-Z **8**-Z

LUMO

HOMO

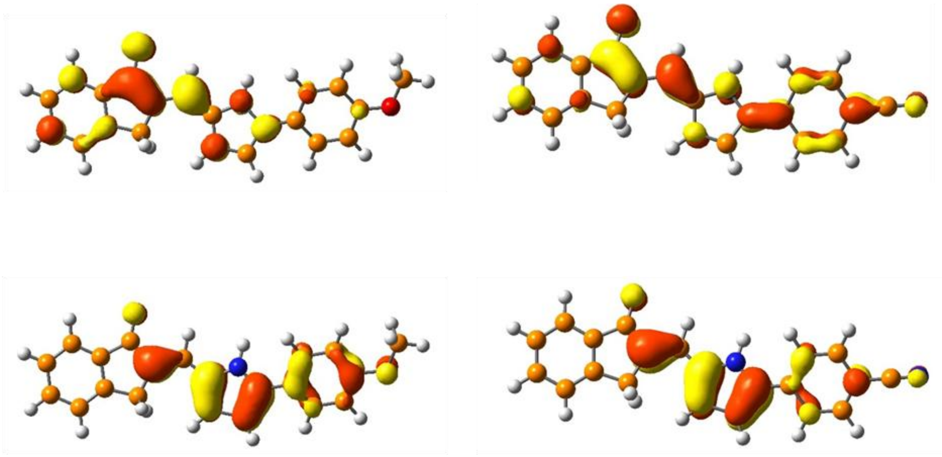

4-E **8**-E

Fig. S41. Energetic diagram of frontier MOs of Z (top) and E (bottom) isomers of **4**, **8** and representations of LUMO (top) and HOMO (bottom).

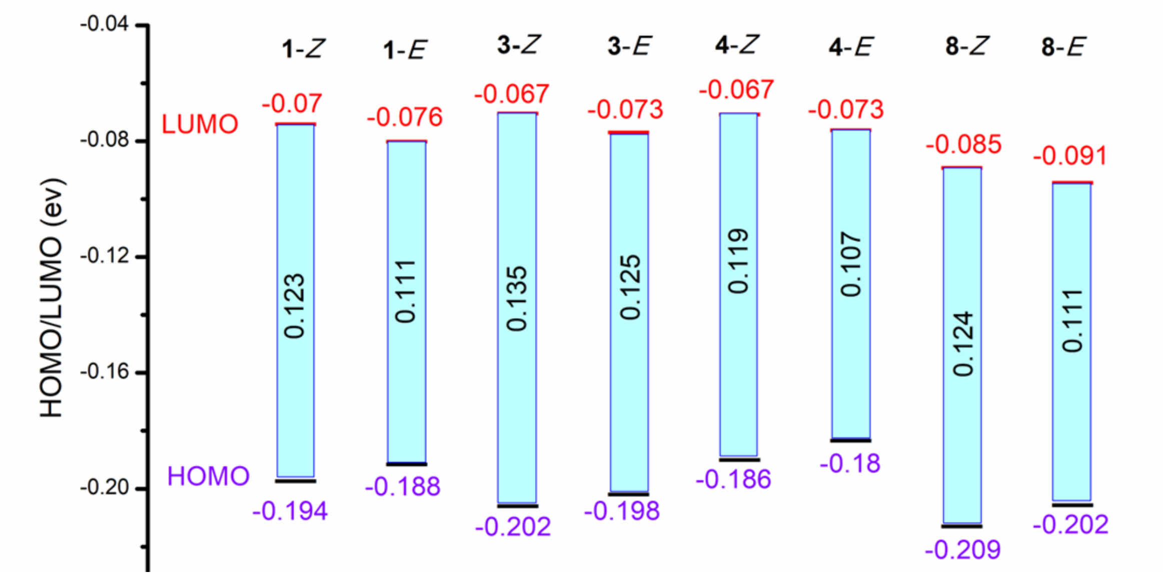

Fig. S42. HOMO-LUMO energy levels of **1**, **3**, **4** & **8**

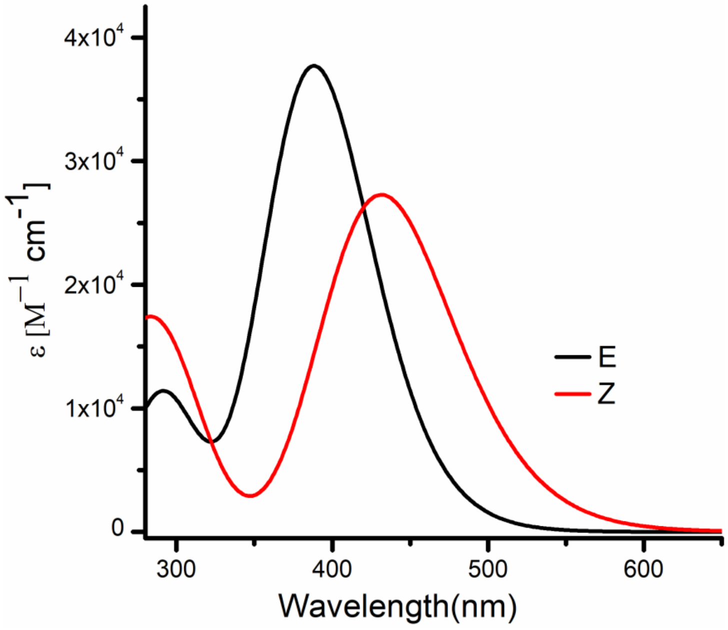

Fig. S43. Calculated UV-Vis absorption spectra of Z (red line) and cis (black line) of switches **1.**

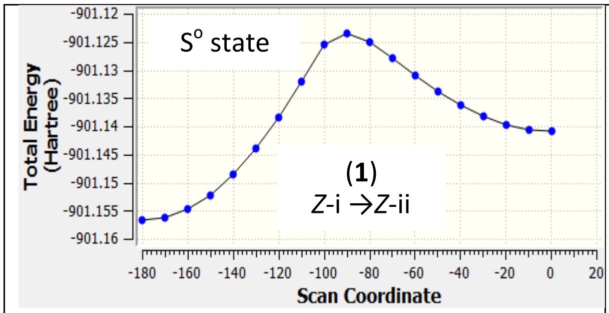
S^{o} state
(1)
Z-i →Z-ii
Total Energy (Hartree)
Scan Coordinate

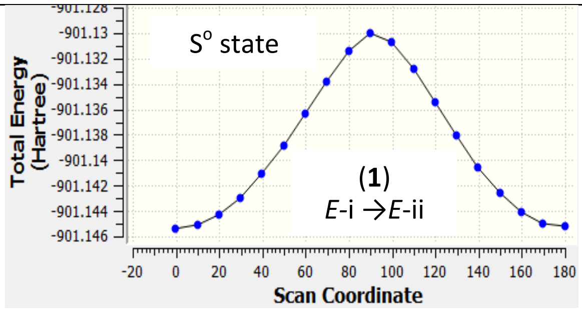
S^{o} state
(1)
E-i →E-ii
Total Energy (Hartree)
Scan Coordinate

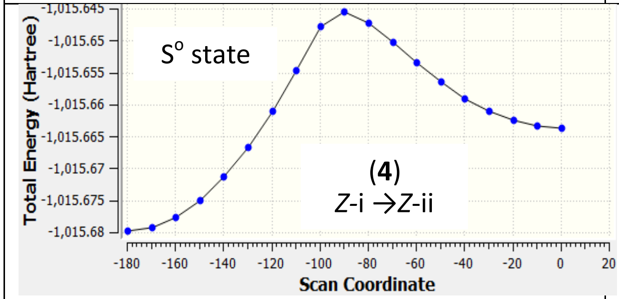
S^{o} state
(4)
Z-i →Z-ii
Total Energy (Hartree)
Scan Coordinate

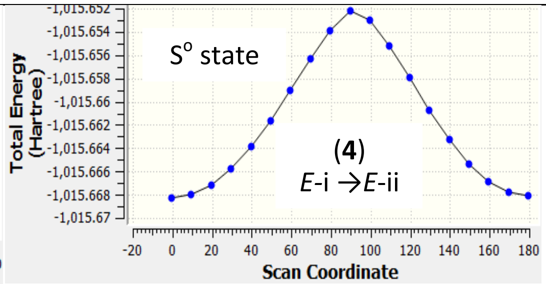
S^{o} state
(4)
E-i →E-ii
Total Energy (Hartree)
Scan Coordinate

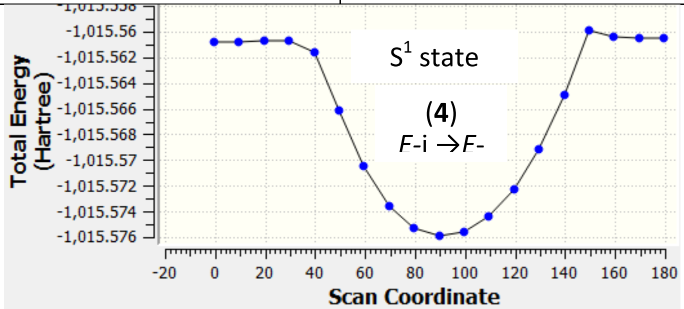
S^{1} state
(4)
F-i →F-
Total Energy (Hartree)
Scan Coordinate

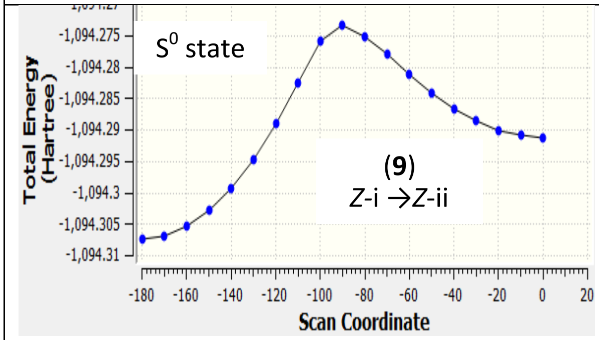
S^{0} state
(9)
Z-i →Z-ii
Total Energy (Hartree)
Scan Coordinate

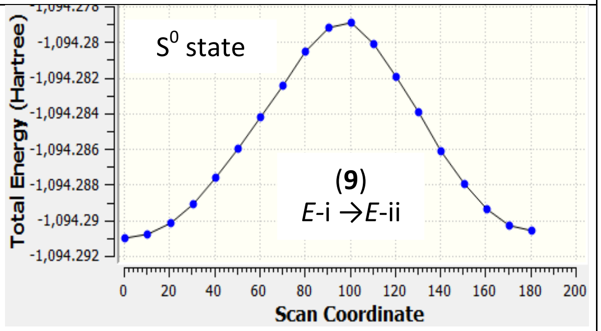
S^{0} state
(9)
E-i →E-ii
Total Energy (Hartree)
Scan Coordinate

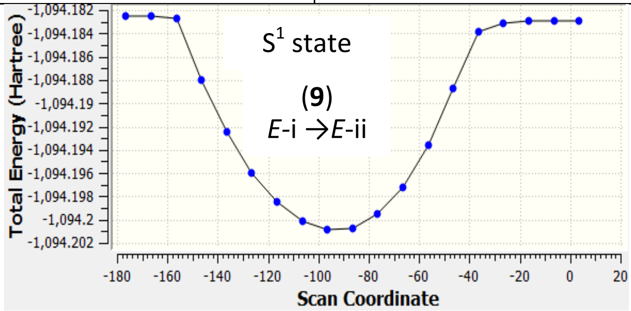
S^{1} state
(9)
E-i →E-ii
Total Energy (Hartree)
Scan Coordinate

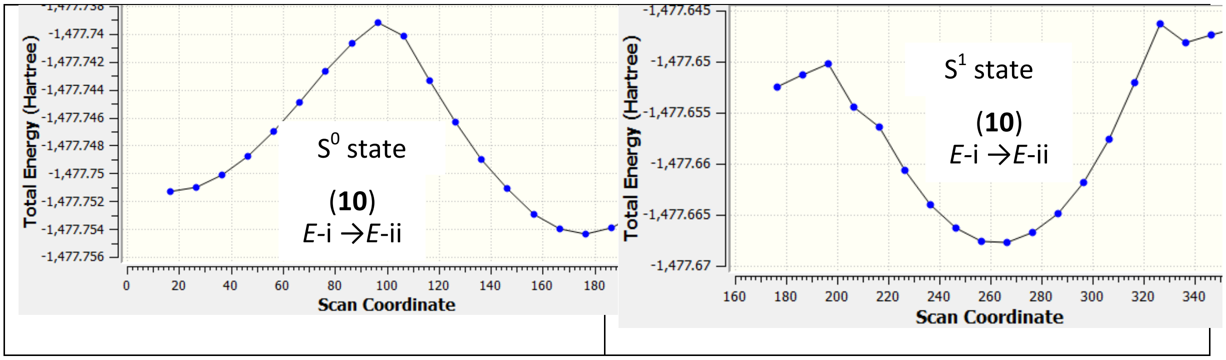

Figure S44: Rotational scan of the C-C bond between the pyrrole ring and the alkene unit in the ground states (s^0) and the first excited states (S^1) for representative compounds **1, 4, 9,** and **10**.

Table S1: Calculated energy barrier of the C-C bond rotation between pyrrole and alkene of both *E*- and Z-isomers in the ground states and excited states for representative compounds **1, 4, 9,** and **10**.

**API**		***Z*-i →*Z*-ii**	***Z*-ii →*Z*-i**	***E*-i →*E*-ii**	***E*-ii →*E*-i**
**1**	S^0	20.9	10.9	9.7	9.5
	S^1	-	-	-9.3	-
**4**	S^0	21.5	10.0	10.1	10.0
	S^1	-	-	-9.4	-
**9**	S^0	21.3	11.1	7.6	7.2
	S^1	-	-	-11.5	-
**10**	S^0	21.4	11.5	7.6	9.5
	S^1	-	-	-11.0	-

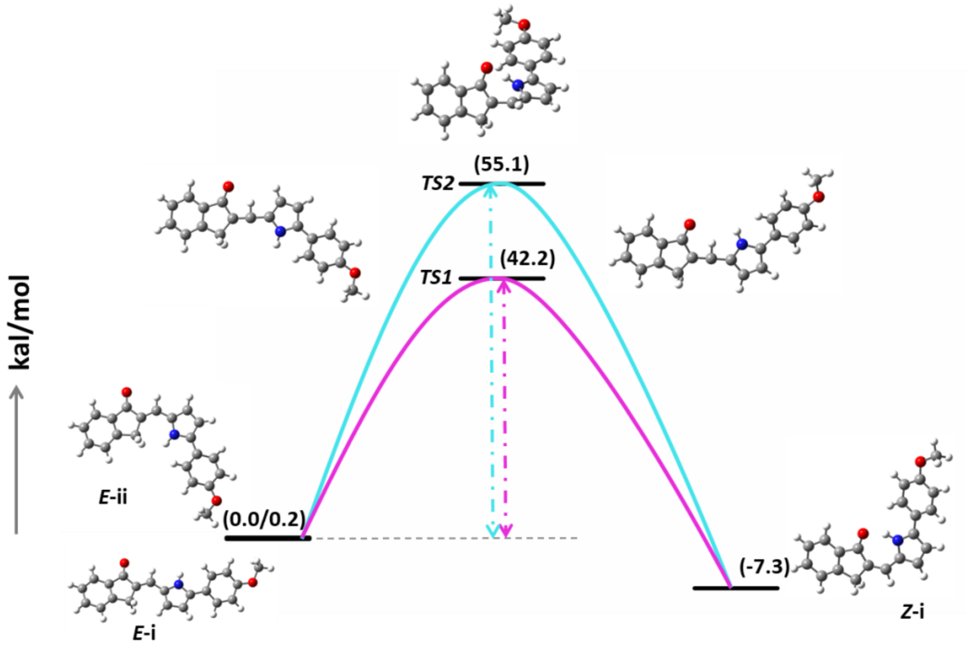

Figure S45. Transition state (TS) calculations of the representative API **4** at b3lyp 6-31G* level of theory TS1 and TS2 represent transition states obtained by inversion and rotation mechanisms around -C=C- bond, respectively. The inversion mechanism appears as the lowest energy pathway than the rotation mechanism for our APIs.

.

Table S2: Calculated energy barrier of the C=C bond rotation and inversion mechanisms of APIs **4**, **9** and **10**.

API	activation energy of rotation mechanism	activation energy of inversion mechanism	$t_{1/2}$[a] at 80 °C (hours)
**4**	55.1 kcal/mol	42.2 kcal/mol	-[b]
**9**	54.5 kcal/mol	32.7 kcal/mol	35
**10**	52.1 kcal/mol	30.8 kcal/mol	26

[a]Half-life measured in toluene solvent, [b]Not determined

Table S3. Crystallographic data for **10**-*Z*

Identification code	SB
Empirical formula	CHNO
Formula weight	467.54
Temperature/K	130.0
Crystal system	triclinic
Space group	P-1
a/Å	10.405(2)
b/Å	11.265(2)
c/Å	11.901(2)
α/°	103.318(5)
β/°	104.698(5)
γ/°	109.450(5)
Volume/Å^3	1194.1(4)
Z	2
ρ_{calc}g/cm^3	1.300
μ/mm^{-1}	0.630
F(000)	492.0
Crystal size/mm^3	0.32 × 0.12 × 0.10
Radiation	CuKα (λ = 1.54178)
2Θ range for data collection/°	20.422 to 150.334
Index ranges	-13 ≤ h ≤ 13, -14 ≤ k ≤ 14, -14 ≤ l ≤ 14
Reflections collected	25838
Independent reflections	4411 [R_{int} = 0.0599, R_{sigma} = 0.0475]
Data/restraints/parameters	4411/0/327
Goodness-of-fit on F^2	1.053
Final R indexes [I>=2σ (I)]	R_1 = 0.0801, wR_2 = 0.2222
Final R indexes [all data]	R_1 = 0.0844, wR_2 = 0.2289
Largest diff. peak/hole / e Å^{-3}	0.42/-0.44

^{1}H and ^{13}C - NMR spectra:

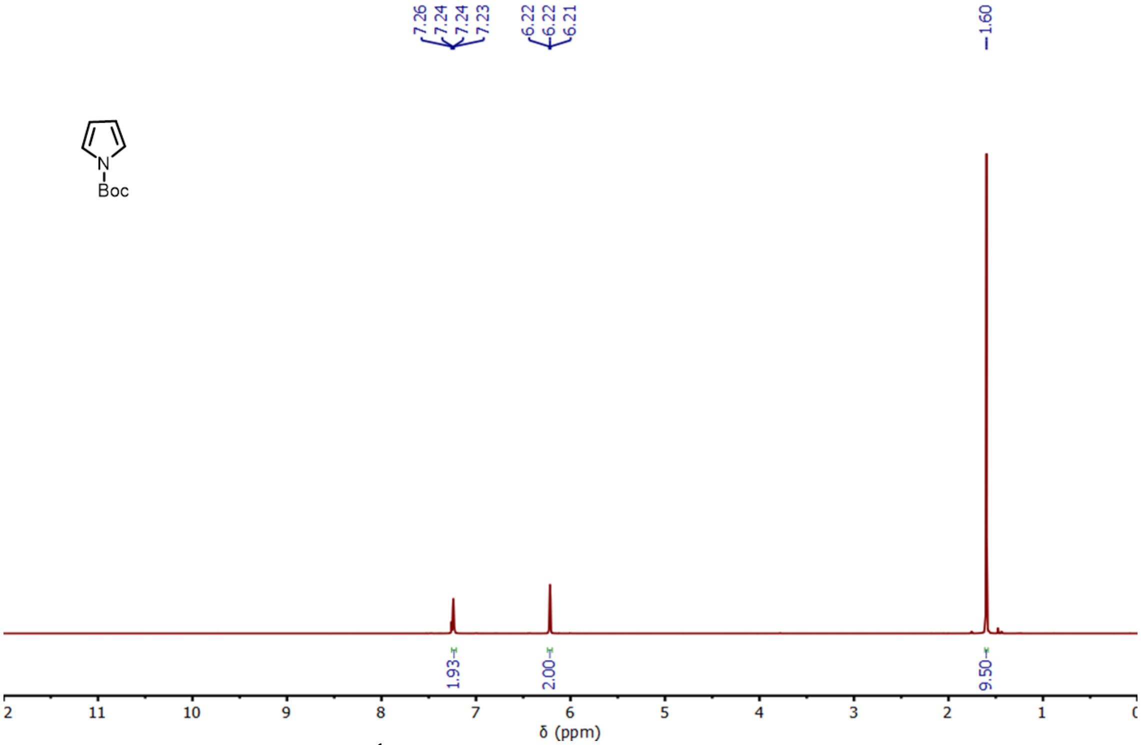

Fig. S46. ^{1}H NMR spectrum (300 MHz, $CDCl_3$) of **12**.

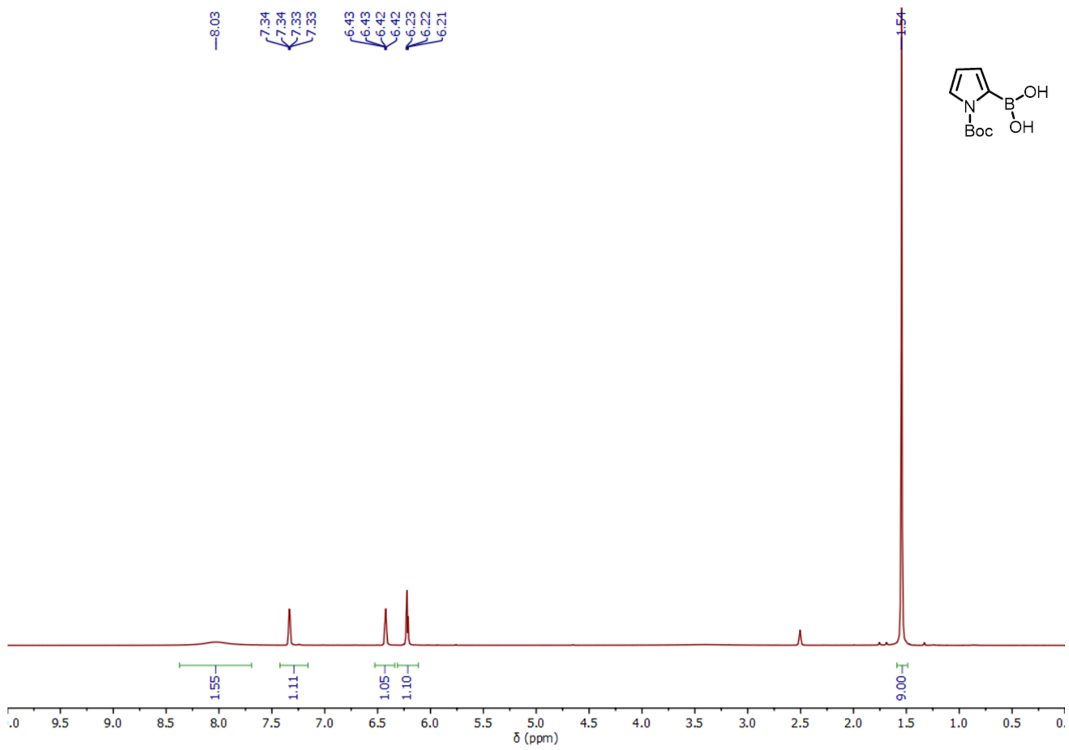

Fig. S47. ^{1}H NMR spectrum (300 MHz, DMSO-d_6) of **13**.

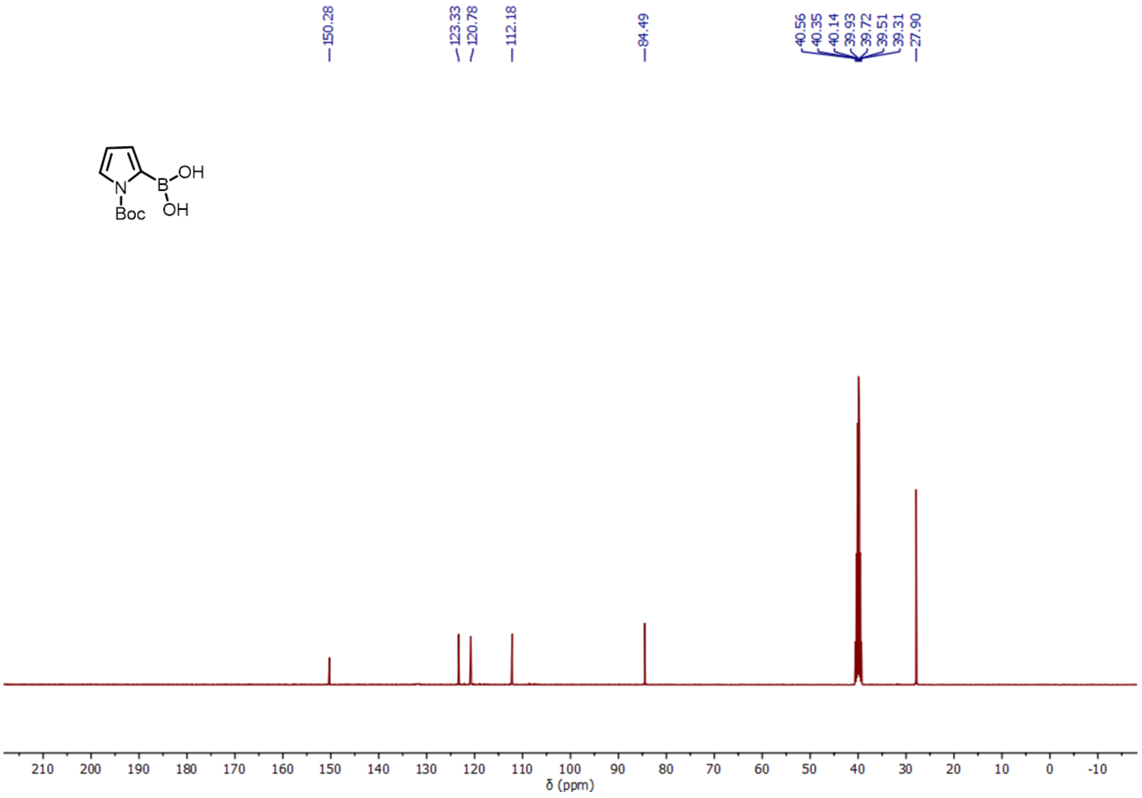

Fig. S48. ^{13}C NMR spectrum (100 MHz, DMSO-d_6) of **13**.

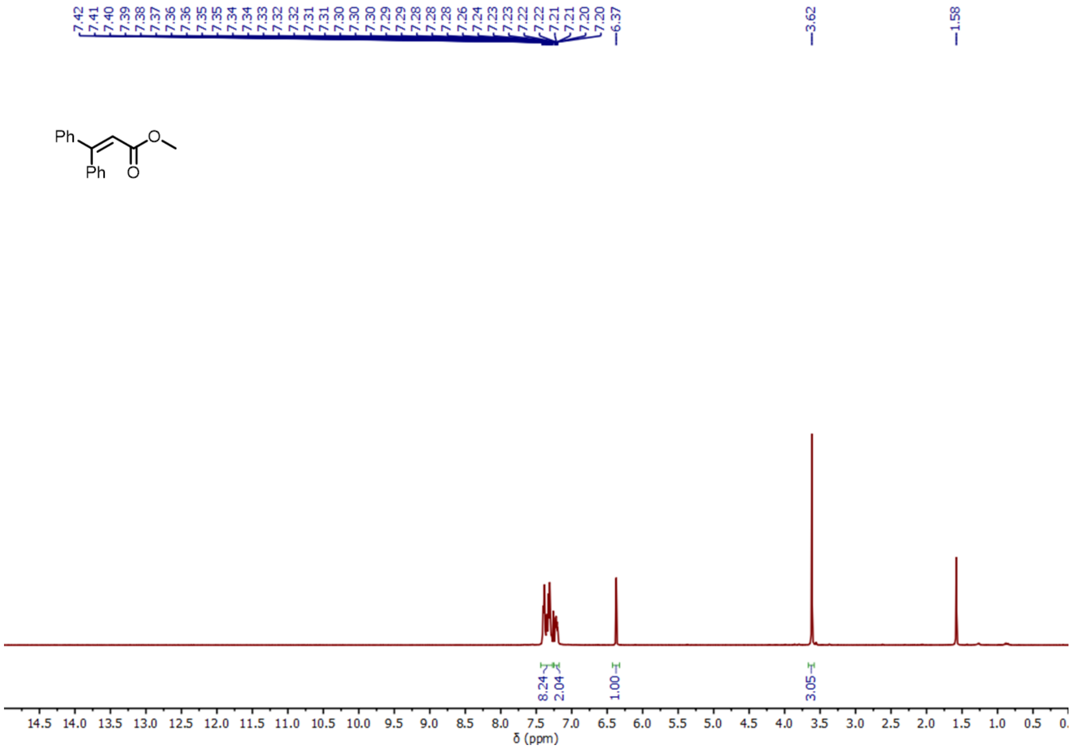

Fig. S49. ^{1}H NMR spectrum (300 MHz, $CDCl_3$) of **17**.

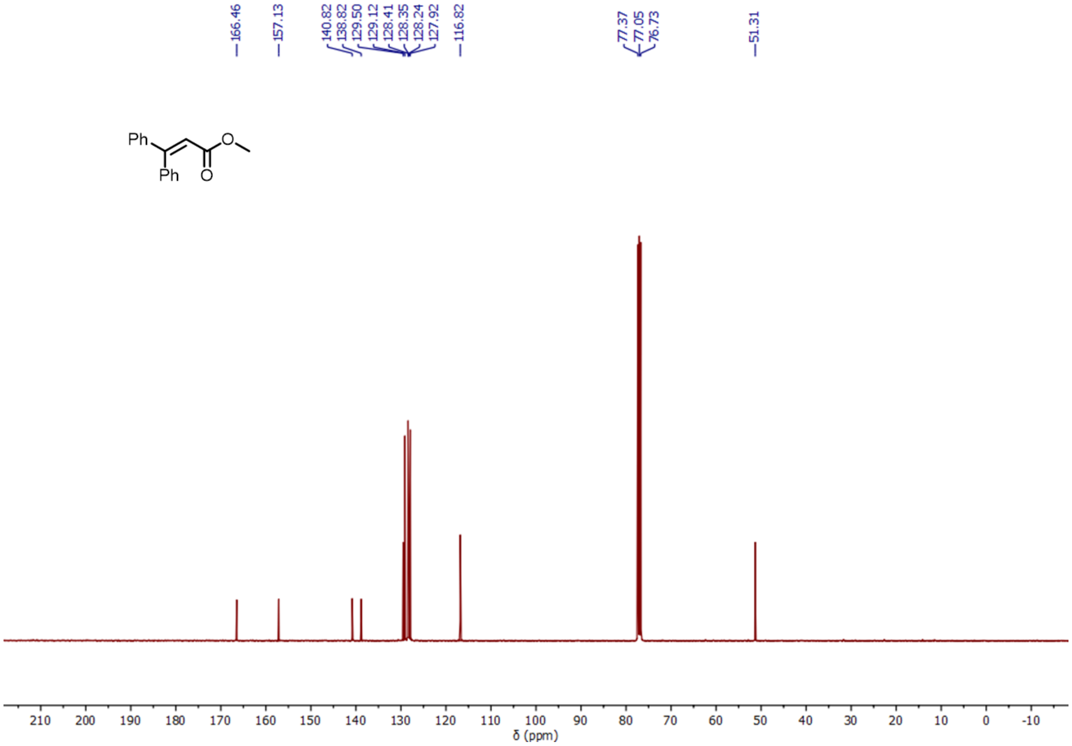

Fig. S50. ^{13}C NMR spectrum (100 MHz, $CDCl_3$) of **17**.

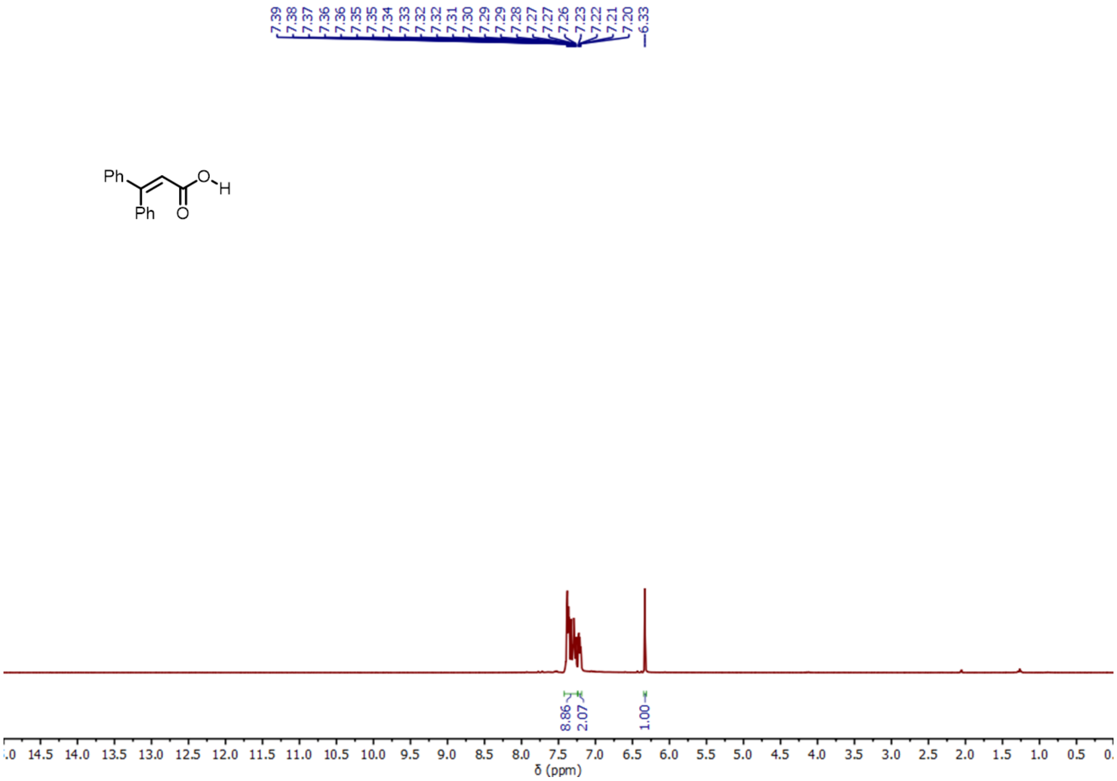

Fig. S51. ^{1}H NMR spectrum (300 MHz, $CDCl_3$) of **18**.

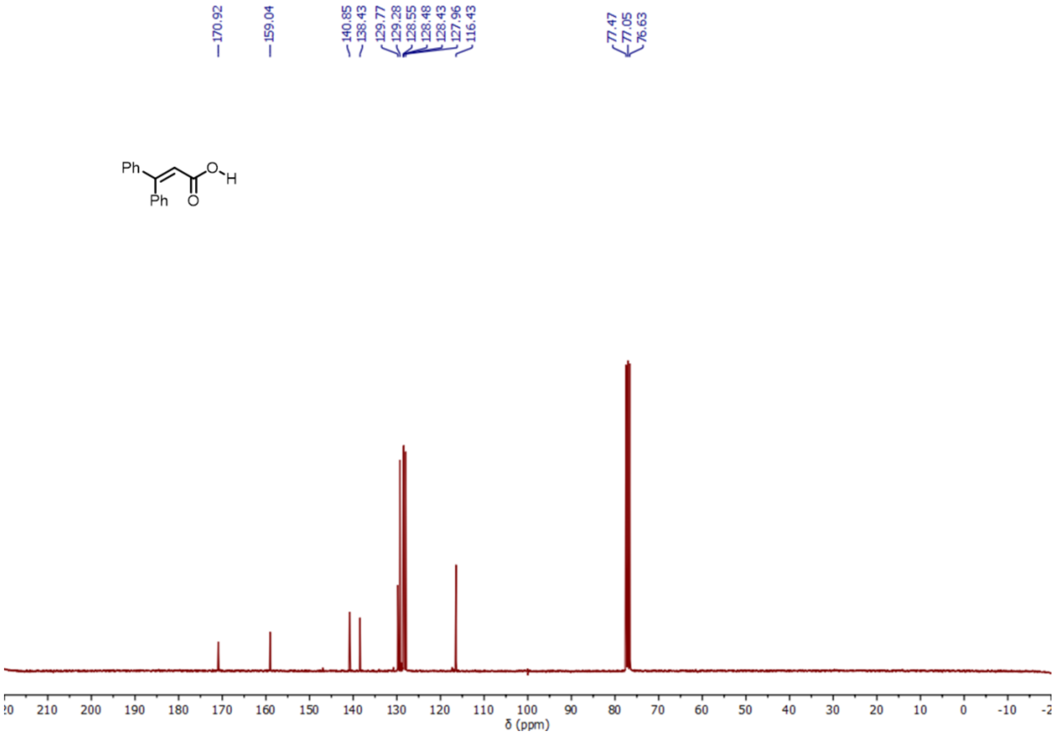

Fig. S52. ^{13}C NMR spectrum (100 MHz, $CDCl_3$) of **18**.

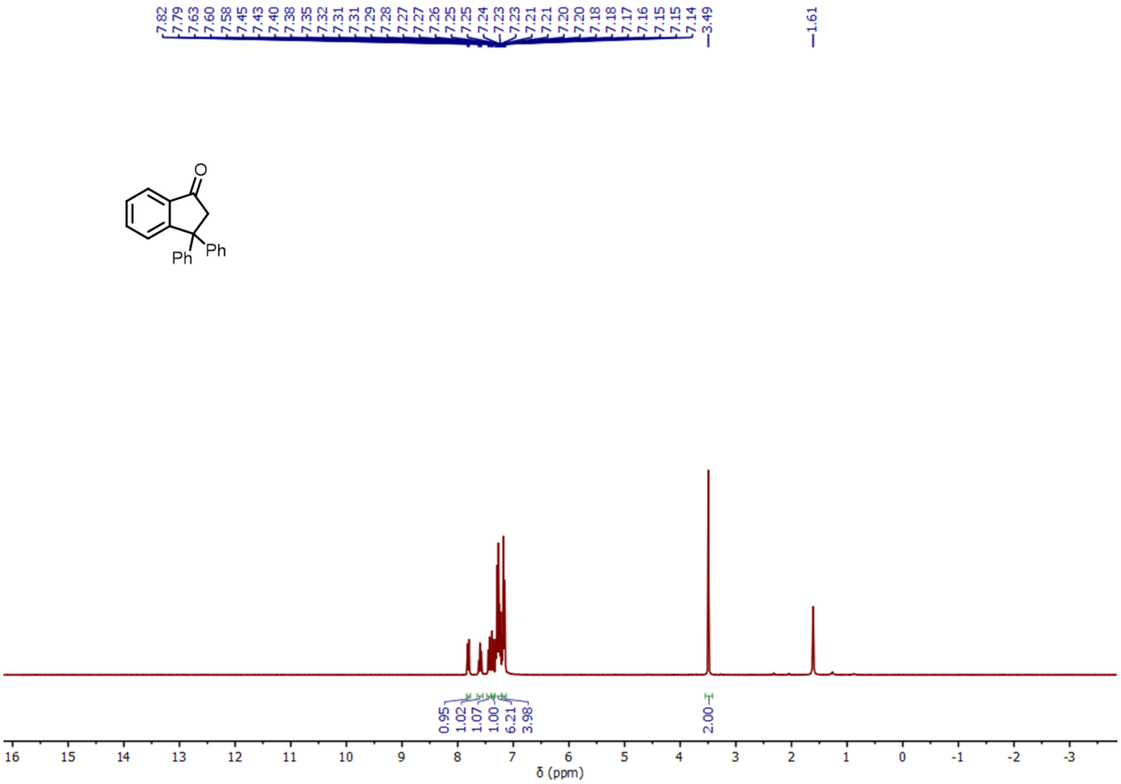

Fig. S53. ^{1}H NMR spectrum (300 MHz, $CDCl_3$) of **19**.

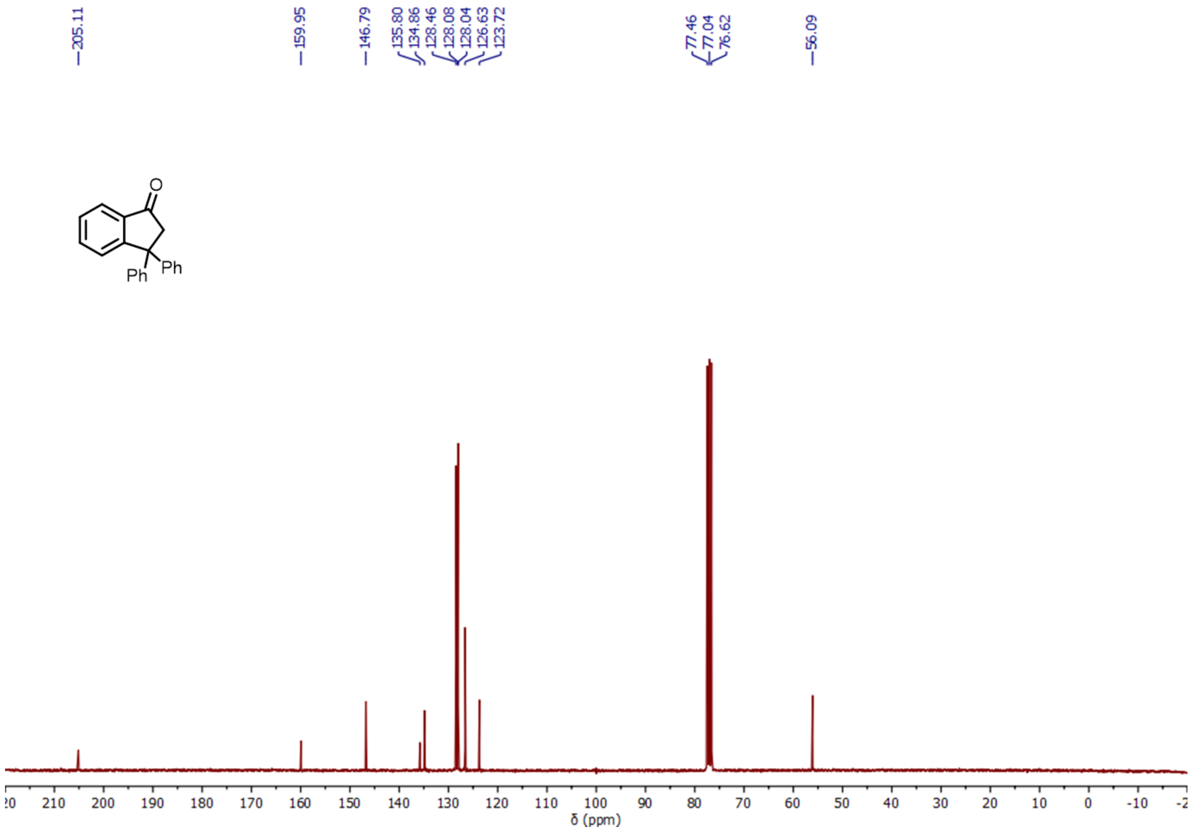

Fig. S54. ^{13}C NMR spectrum (75 MHz, $CDCl_3$) of **19**.

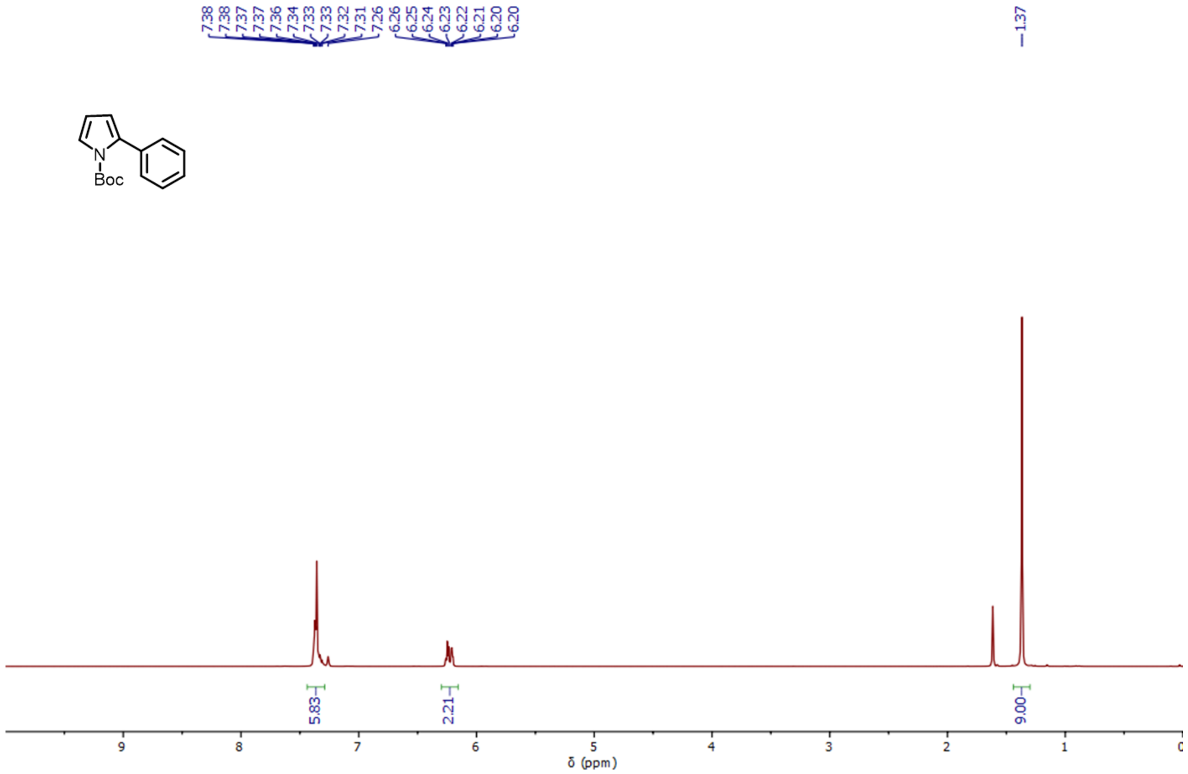

Fig. S55. ^{1}H NMR spectrum (300 MHz, $CDCl_3$) of **14a**.

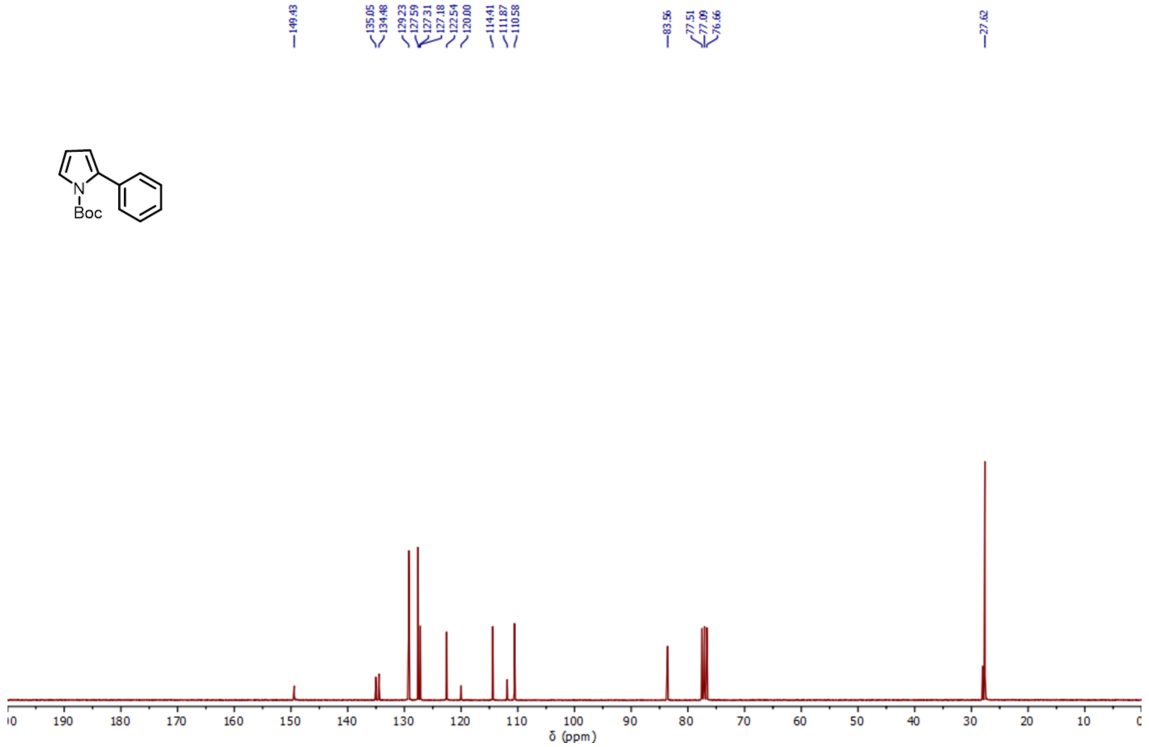

Fig. S56. ^{13}C NMR spectrum (75 MHz, $CDCl_3$) of **14a.**

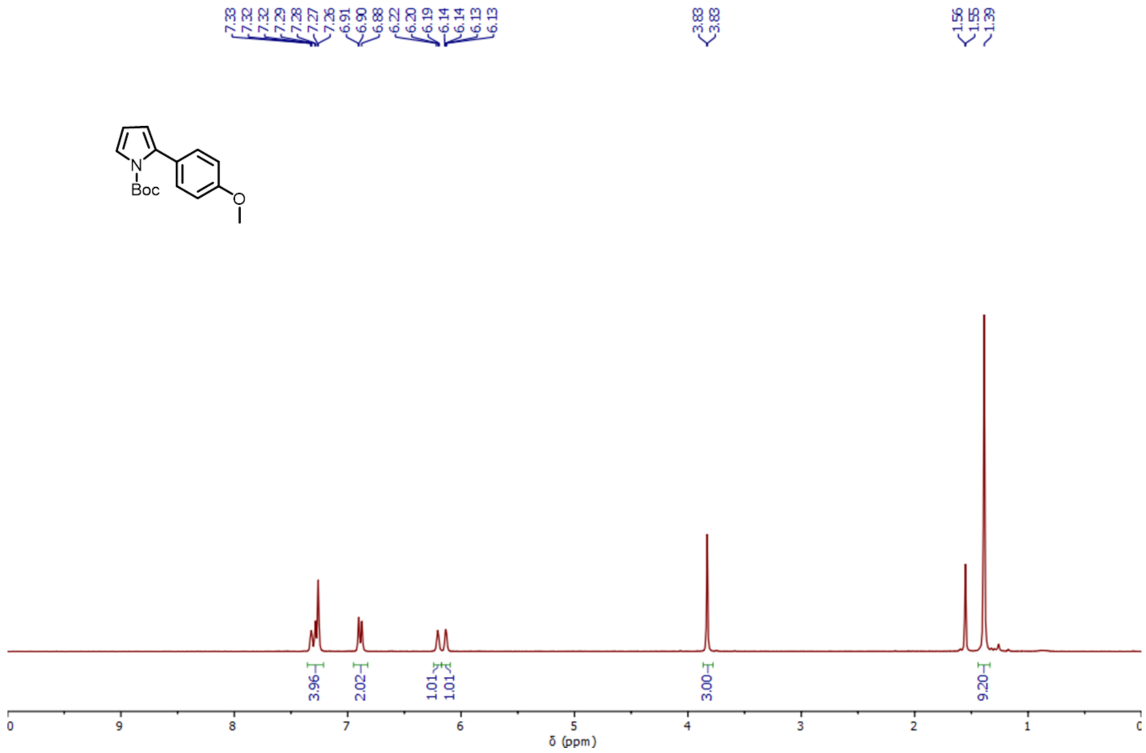

Fig. S57. ^{1}H NMR spectrum (300 MHz, $CDCl_3$) of **14b.**

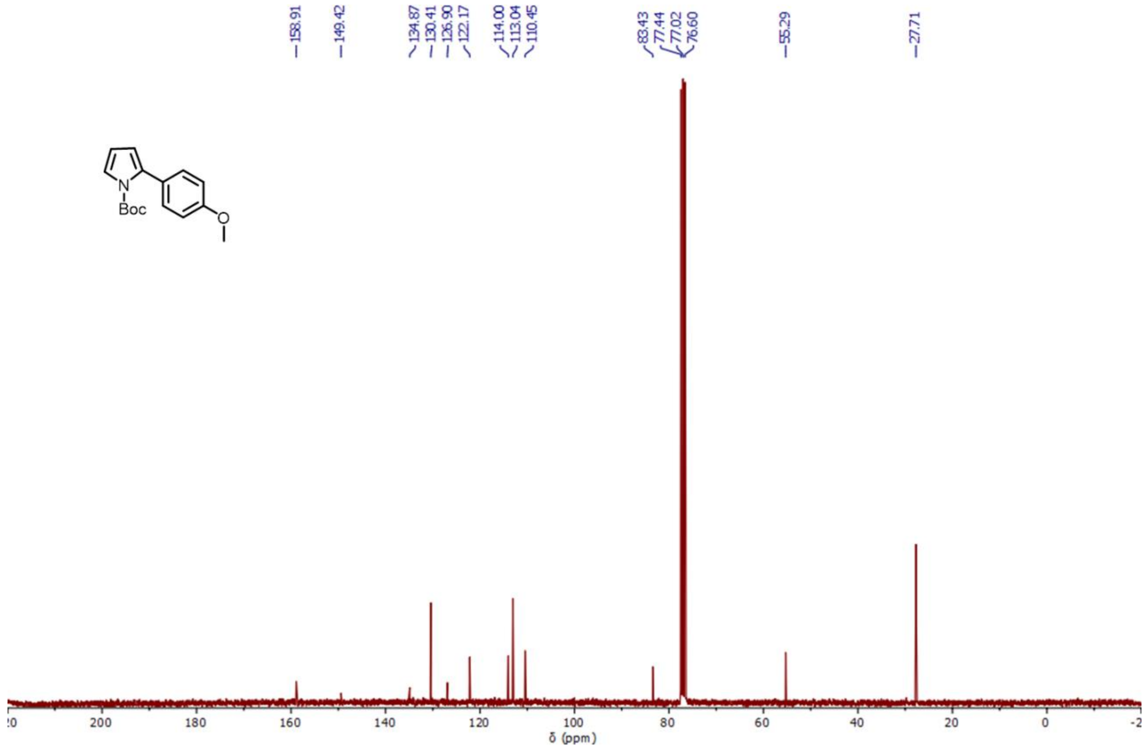

Fig. S58. ^{13}C NMR spectrum (75 MHz, $CDCl_3$) of **14b.**

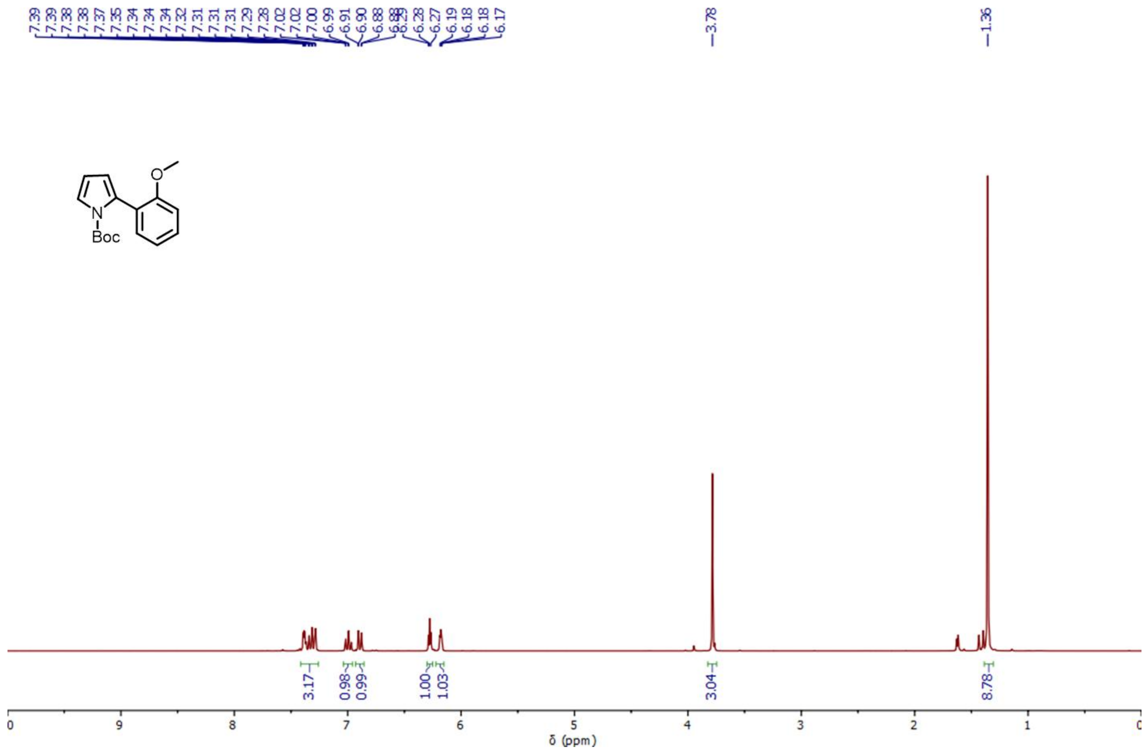

Fig. S59. ^{1}H NMR spectrum (300 MHz, $CDCl_3$) of **14c.**

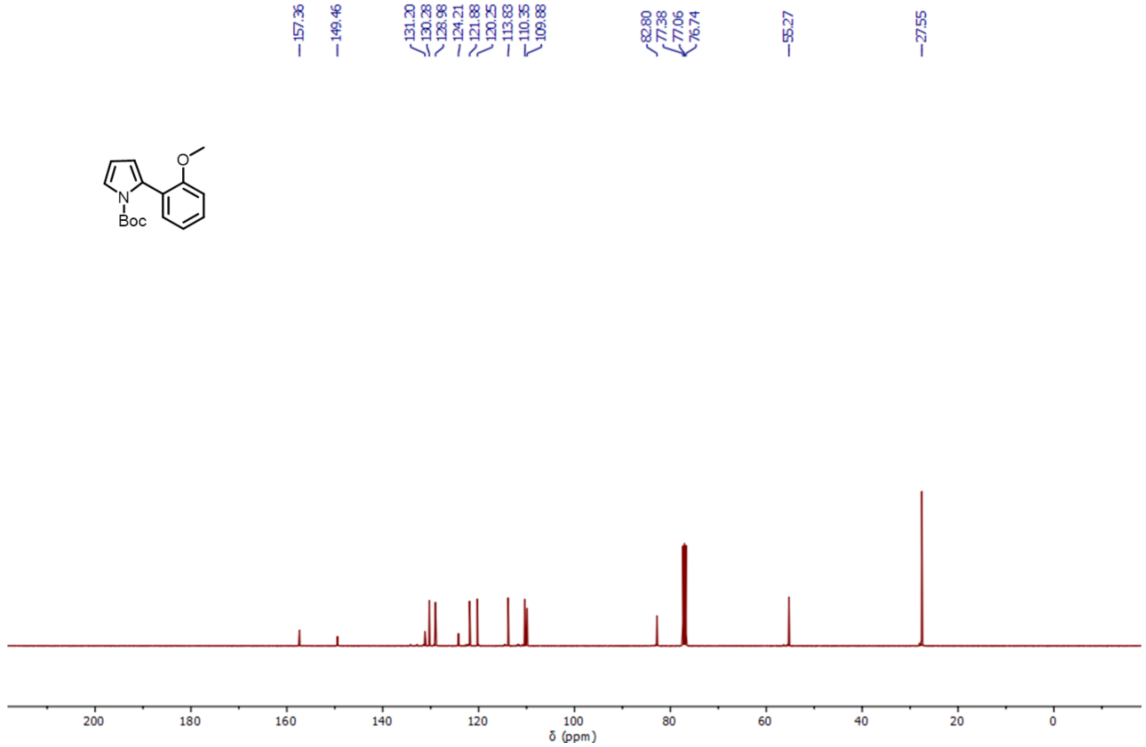

Fig. S60. ^{13}C NMR spectrum (100 MHz, $CDCl_3$) of **14c.**

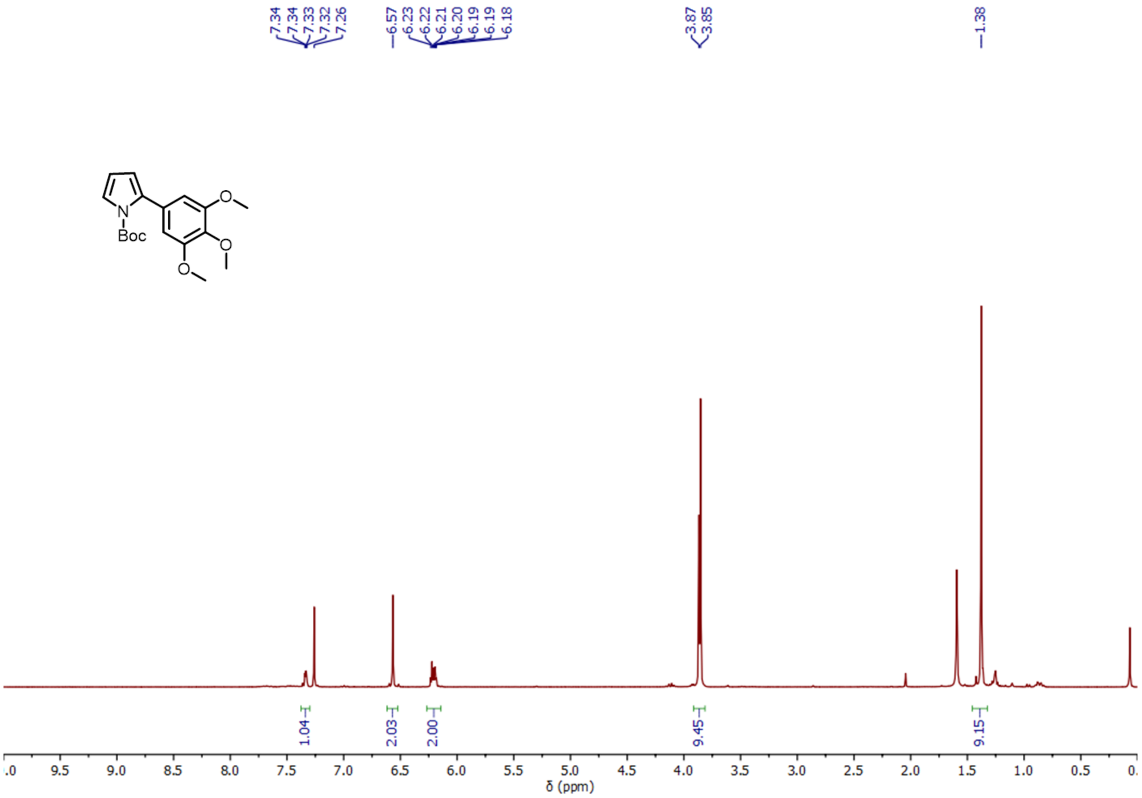

Fig. S61. ^{1}H NMR spectrum (300 MHz, $CDCl_3$) of **14d.**

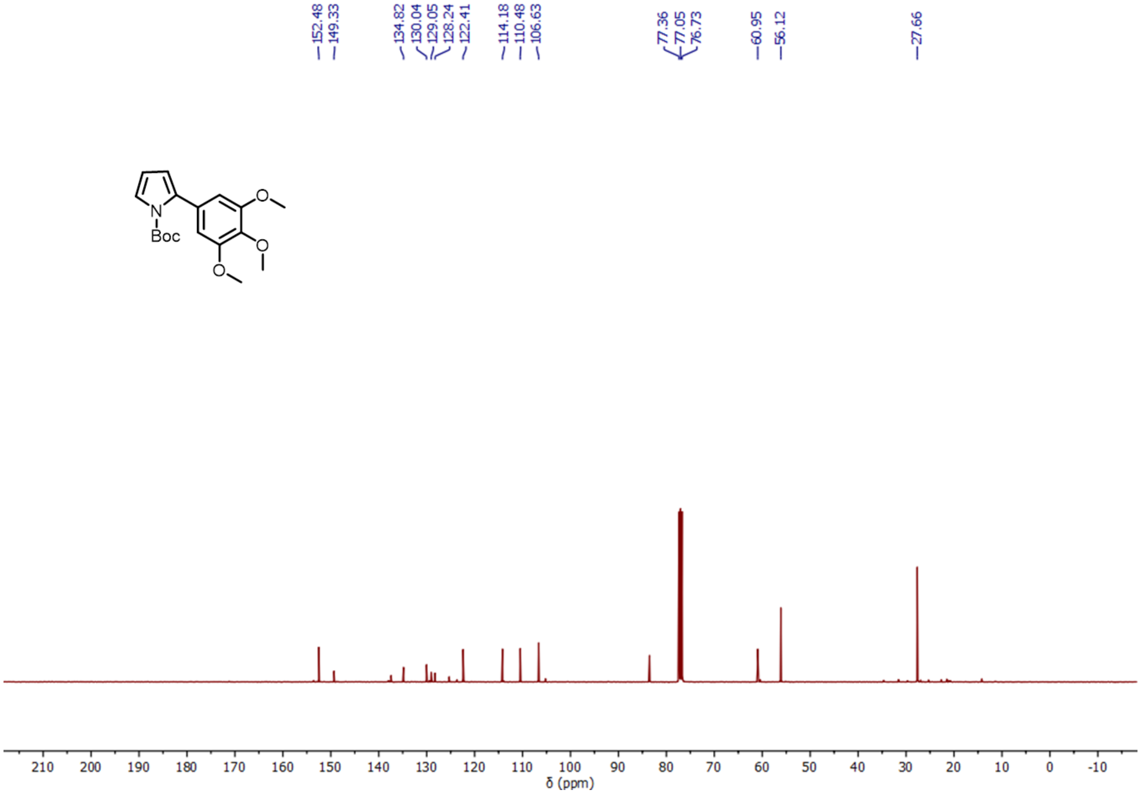

Fig. S62. ^{13}C NMR spectrum (100 MHz, $CDCl_3$) of **14d.**

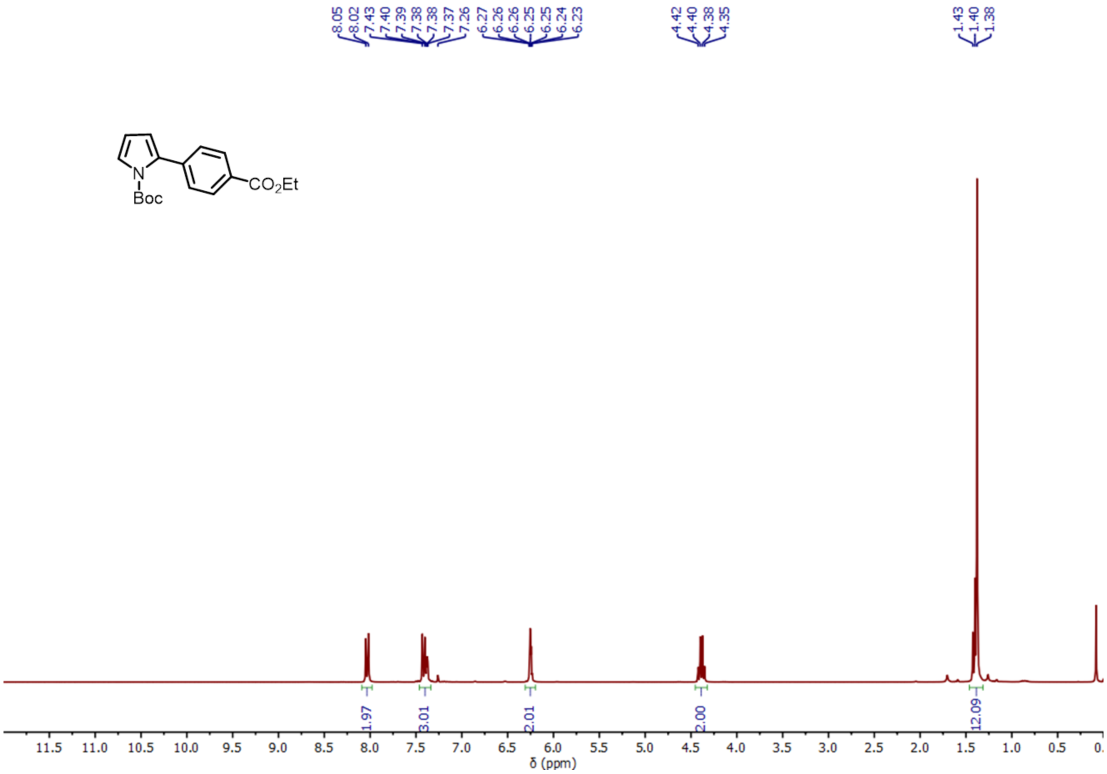

Fig. S63. ^{1}H NMR spectrum (300 MHz, $CDCl_3$) of 14e.

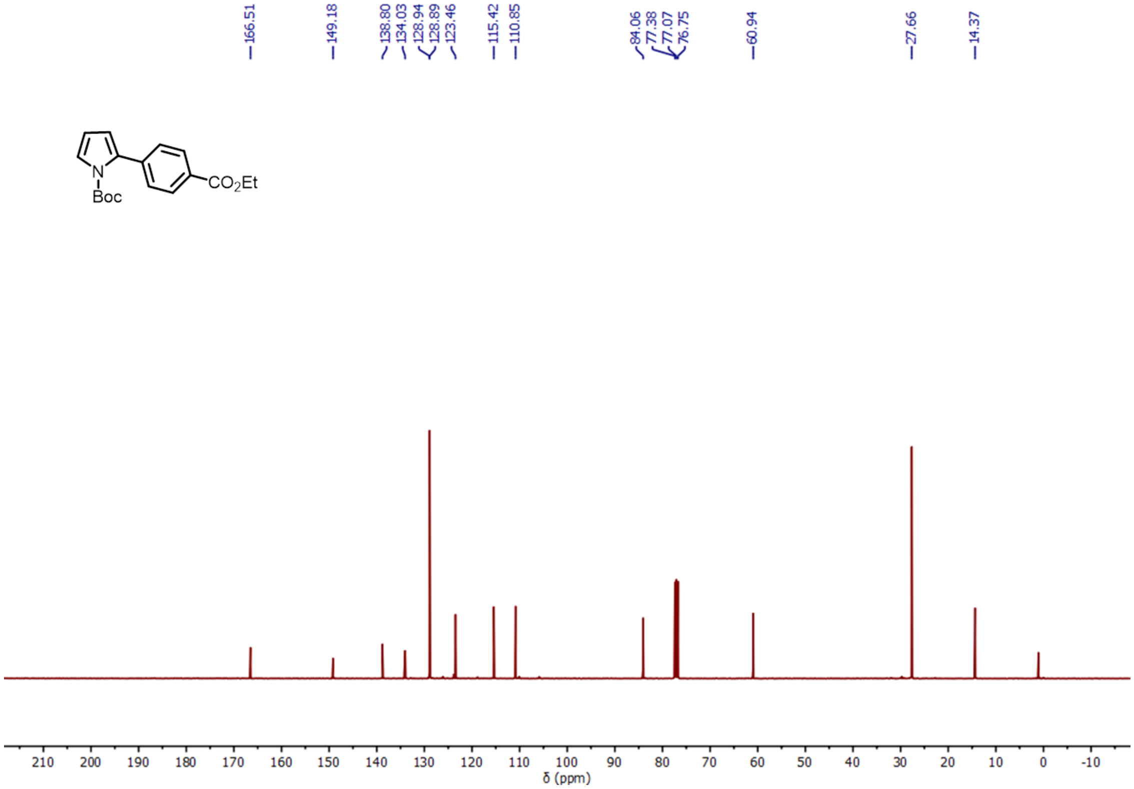

Fig. S64. ^{13}C NMR spectrum (100 MHz, $CDCl_3$) of 14e.

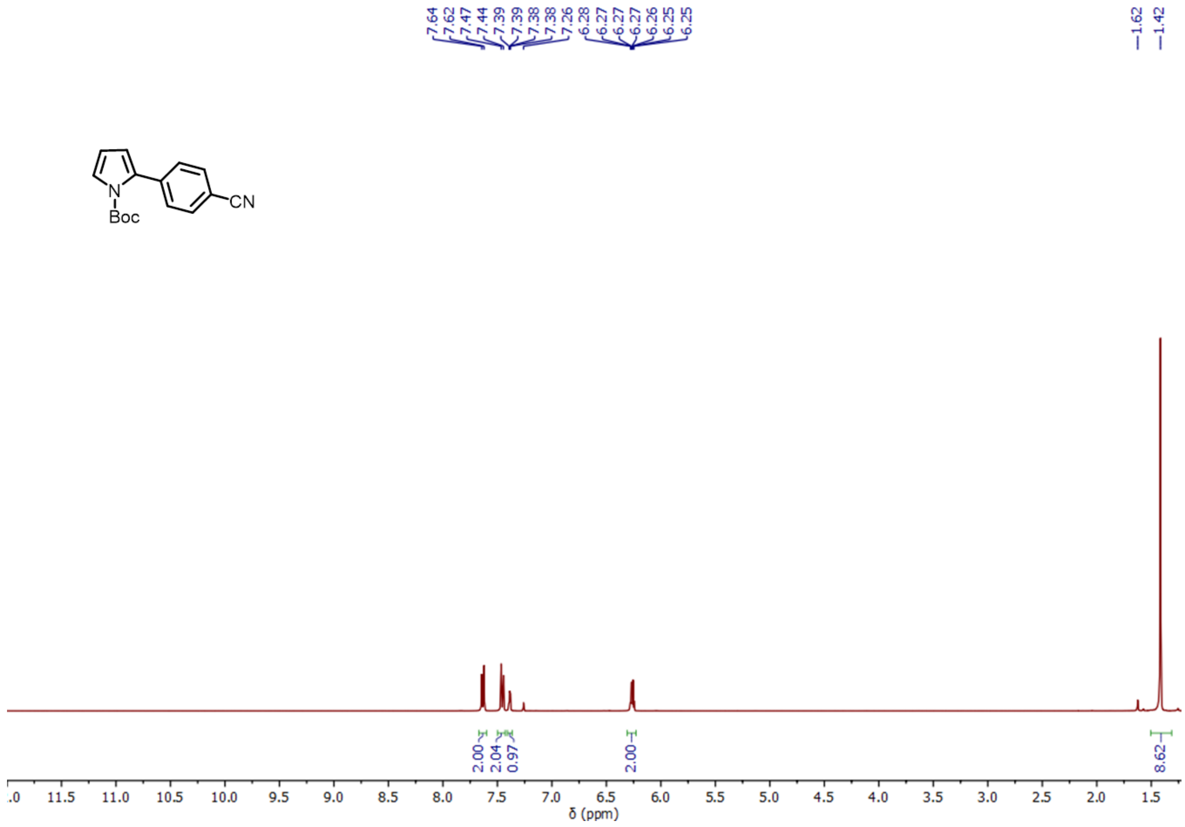

Fig. S65. ^{1}H NMR spectrum (400 MHz, $CDCl_3$) of 14f.

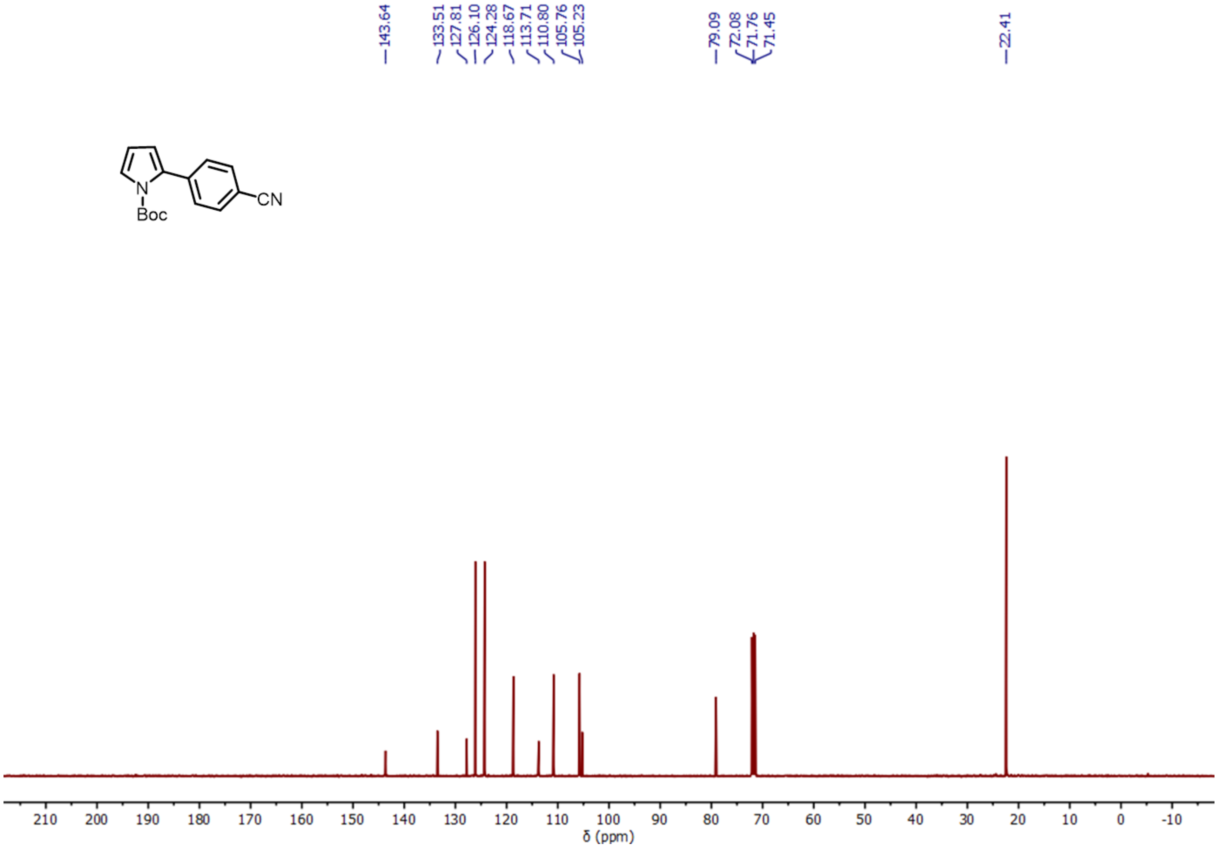

Fig. S66. ^{13}C NMR spectrum (100 MHz, $CDCl_3$) of 14f.

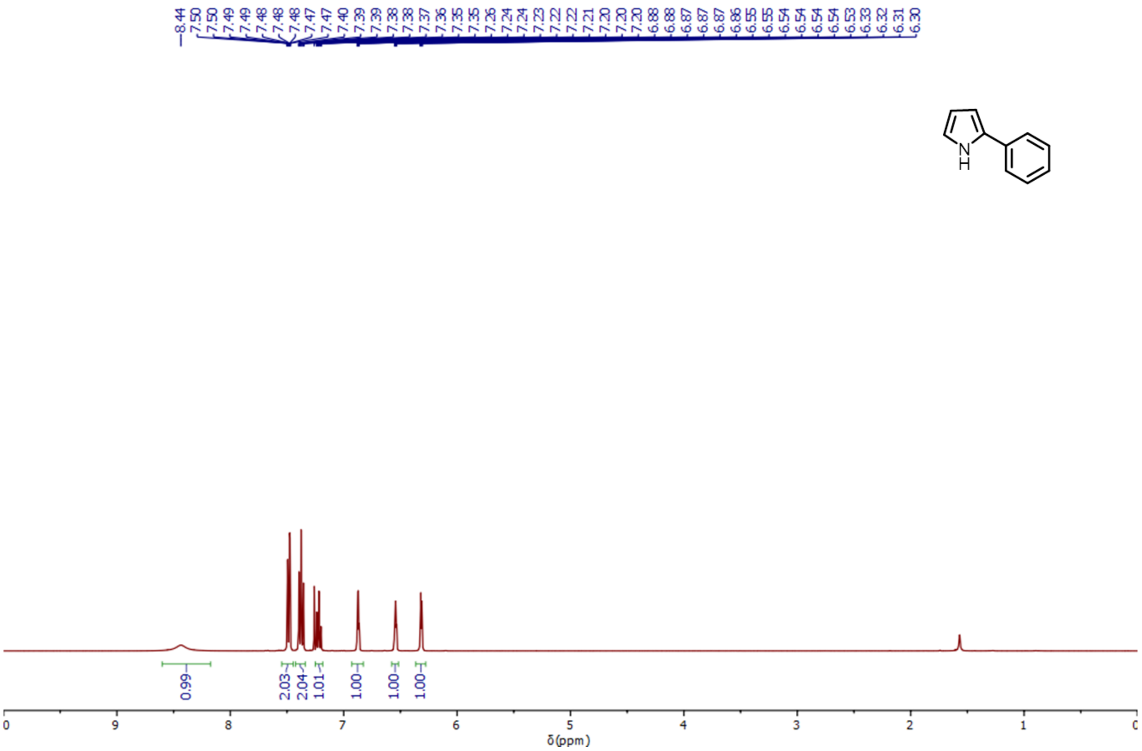

Fig. S67. ^{1}H NMR spectrum (400 MHz, $CDCl_3$) of **15a.**

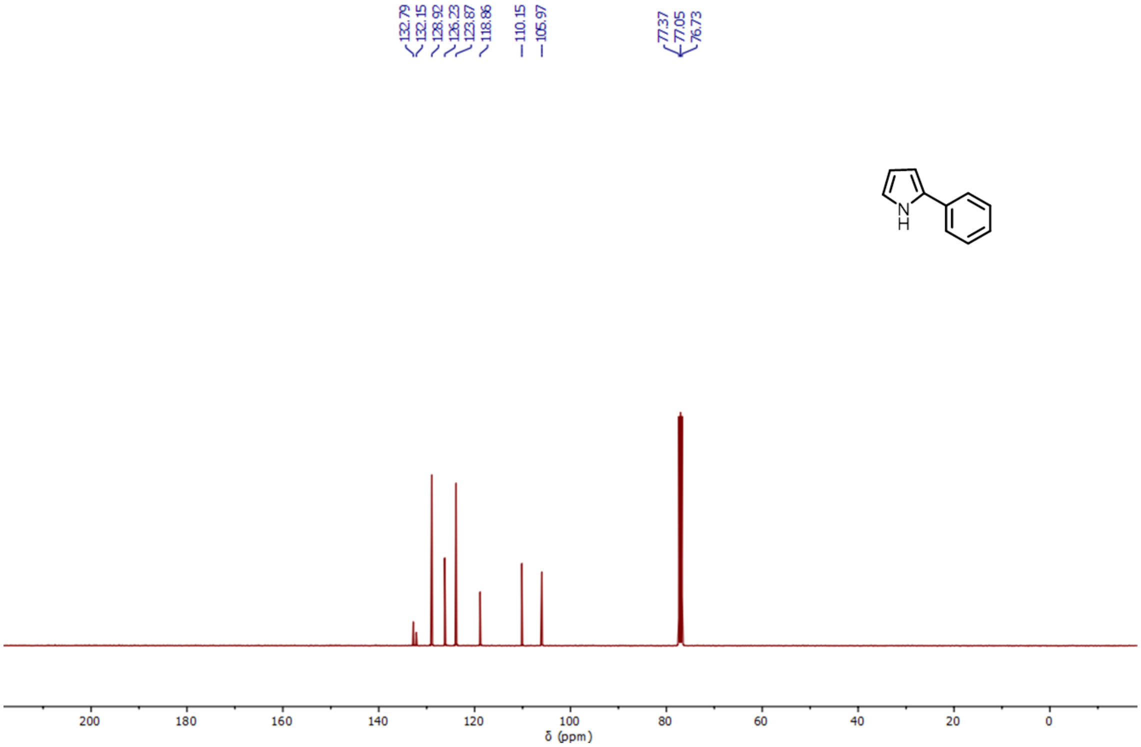

Fig. S68. ^{13}C NMR spectrum (100 MHz, $CDCl_3$) of **15a.**

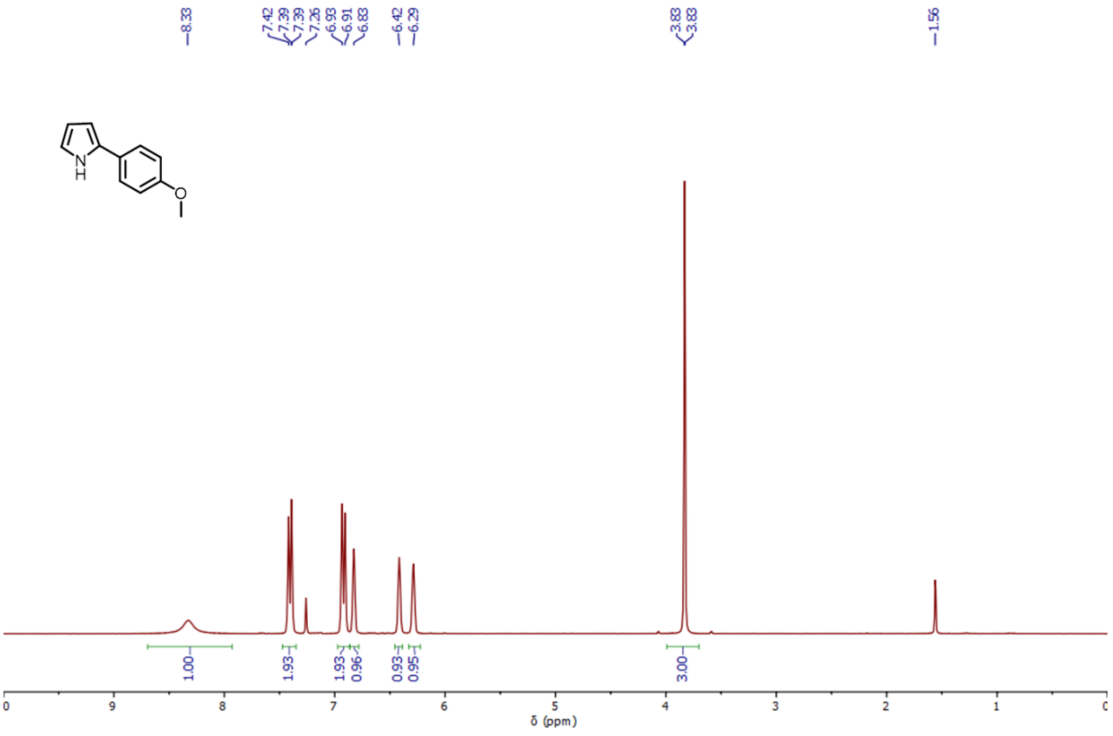

Fig. S69. ^{1}H NMR spectrum (300 MHz, $CDCl_3$) of **15b.**

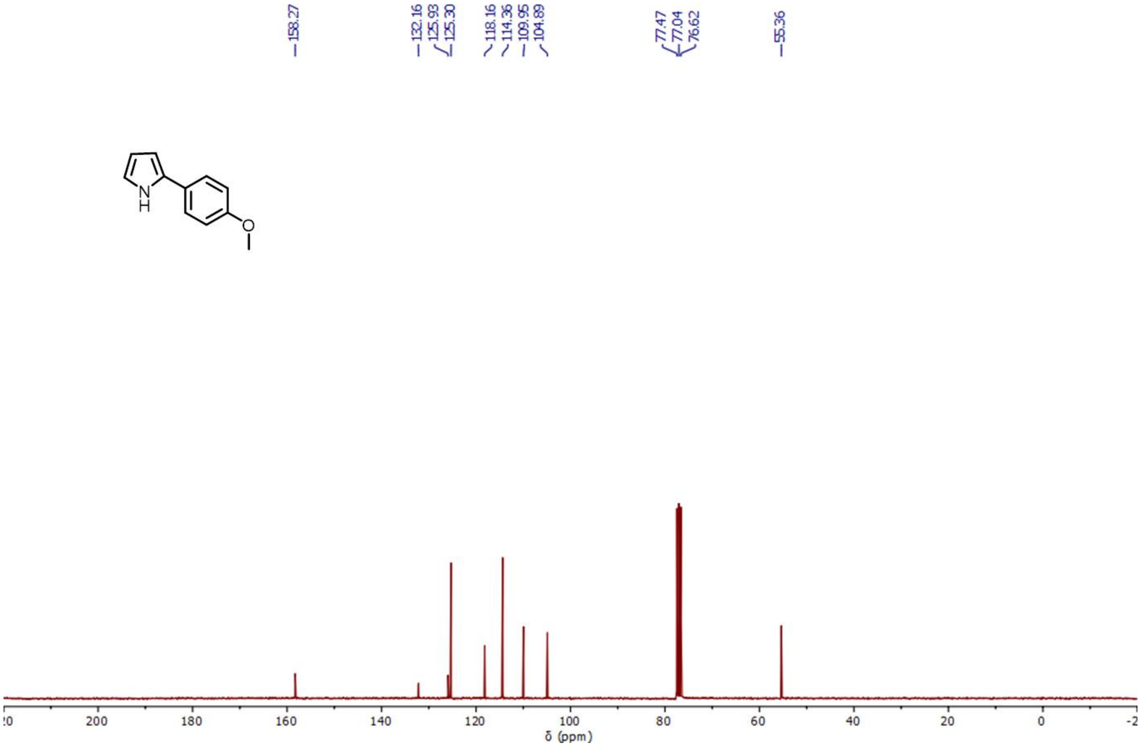

Fig. S70. ^{13}C NMR spectrum (75 MHz, $CDCl_3$) of **15b.**

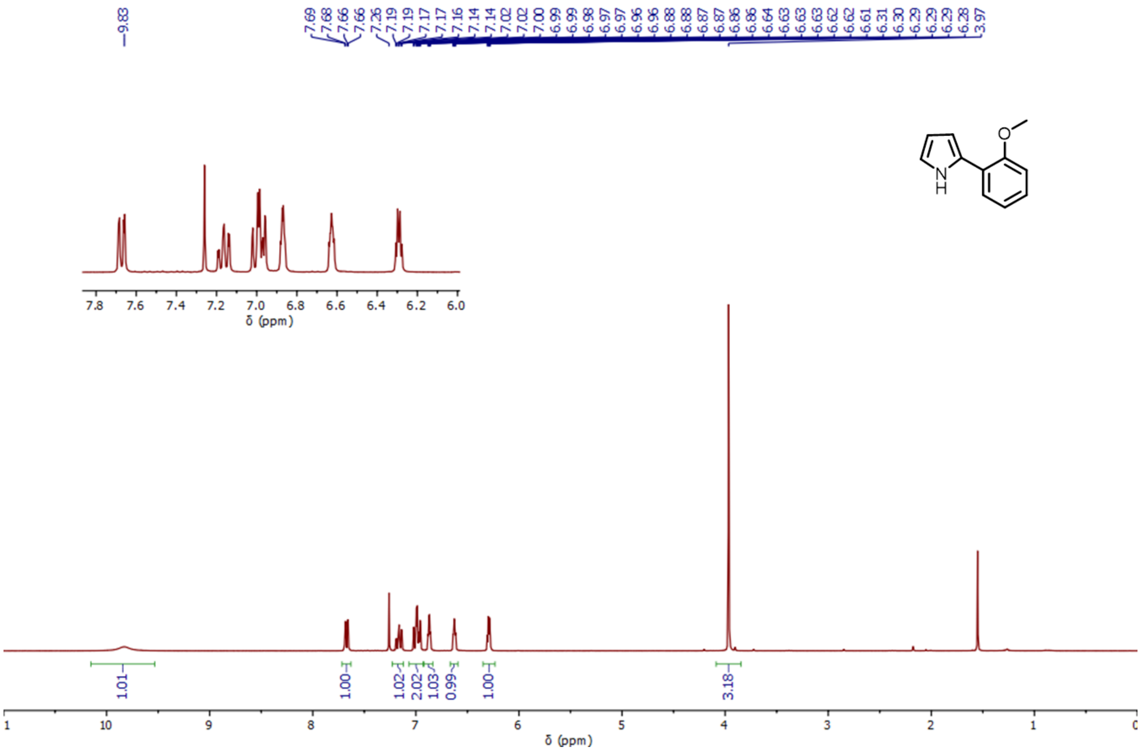

Fig. S71. ^{1}H NMR spectrum (300 MHz, $CDCl_3$) of **15c.**

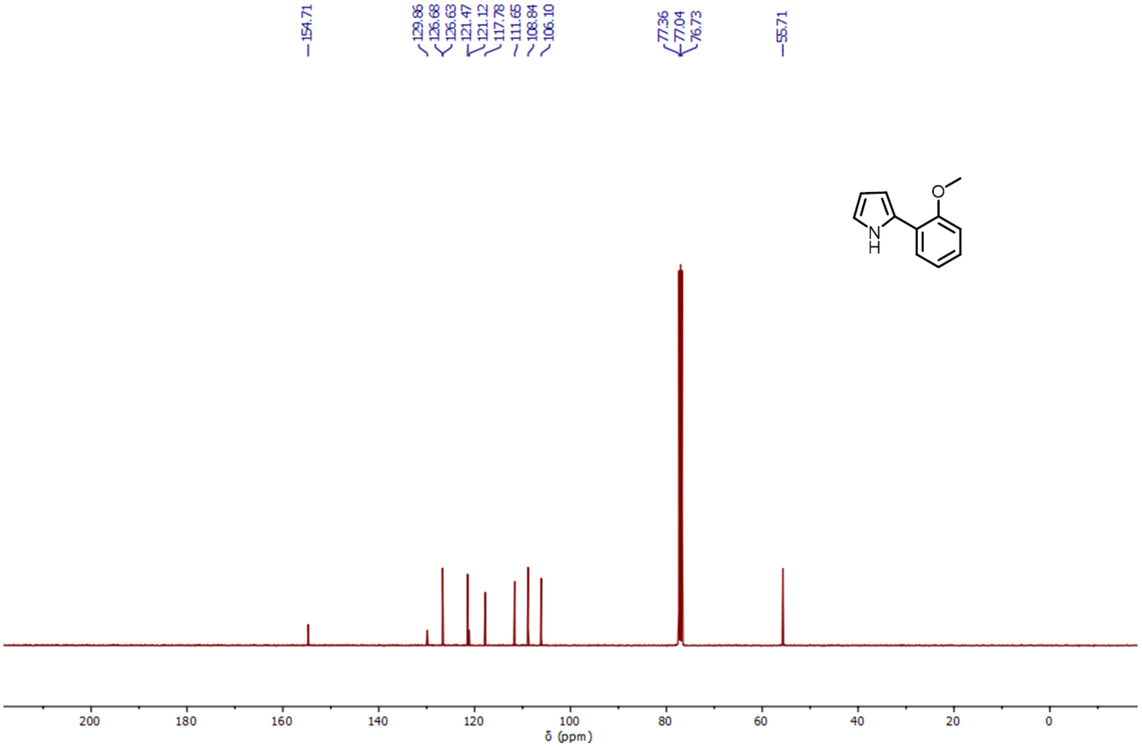

Fig. S72. ^{13}C NMR spectrum (100 MHz, $CDCl_3$) of **15c.**

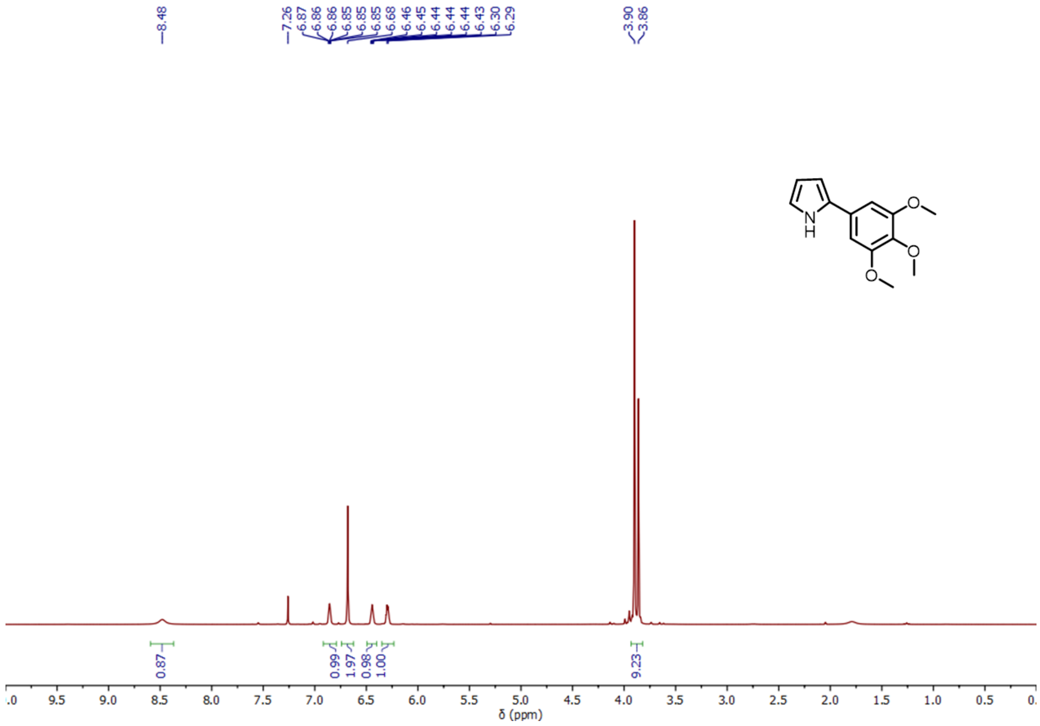

Fig. S73. ^{1}H NMR spectrum (300 MHz, $CDCl_3$) of **15d.**

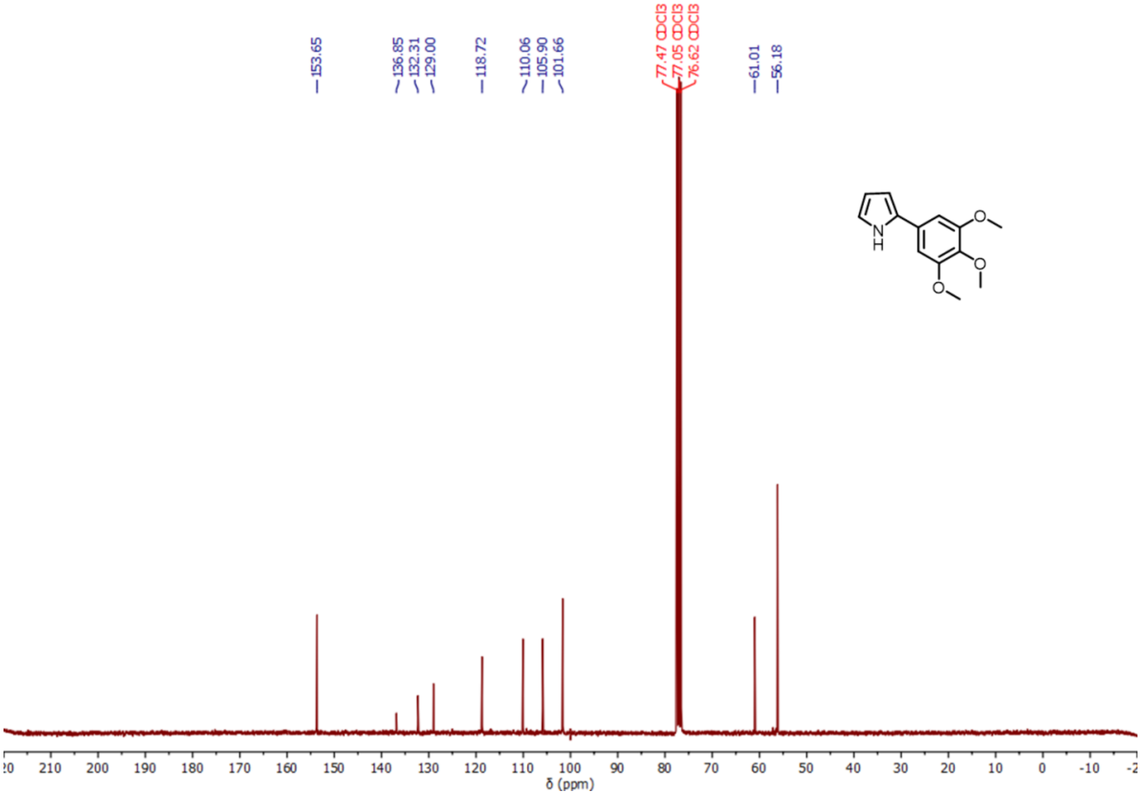

Fig. S74. ^{13}C NMR spectrum (75 MHz, $CDCl_3$) of **15d.**

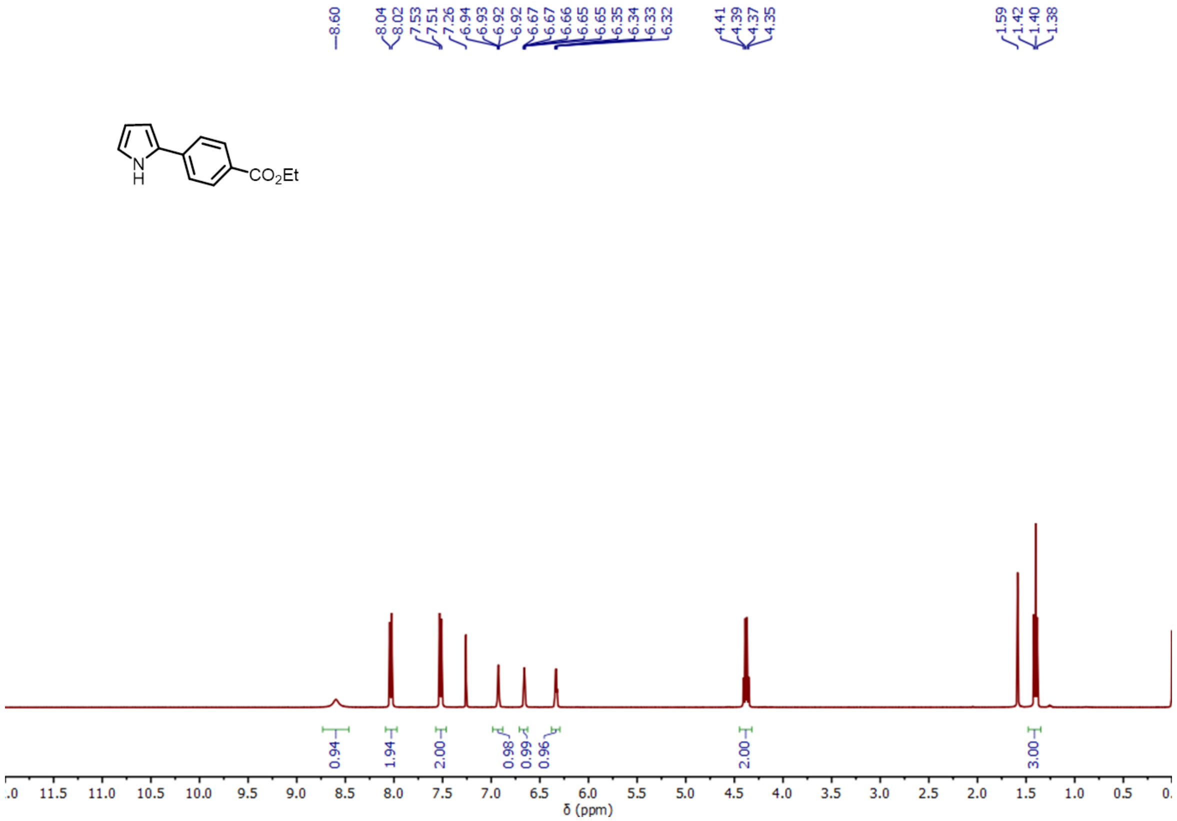

Fig. S75. ^{1}H NMR spectrum (400 MHz, $CDCl_3$) of 15e.

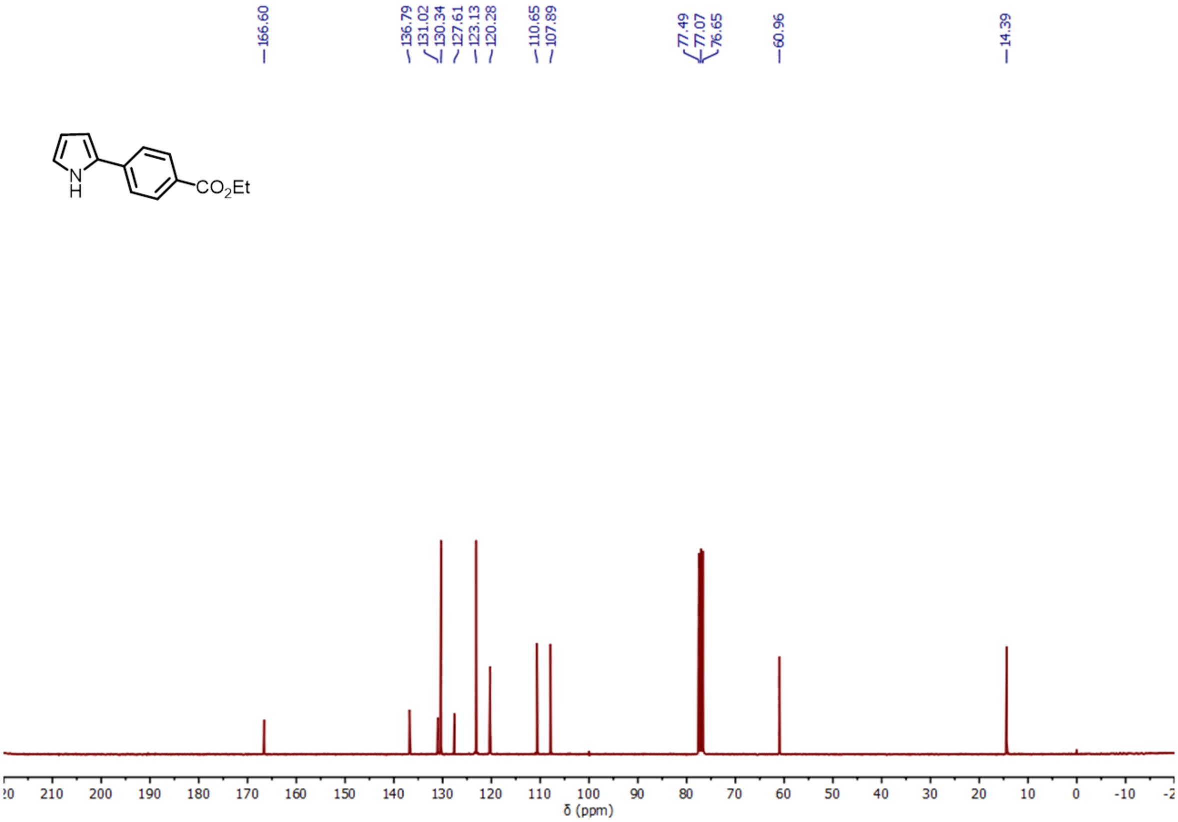

Fig. S76. ^{13}C NMR spectrum (75 MHz, $CDCl_3$) of **15e.**

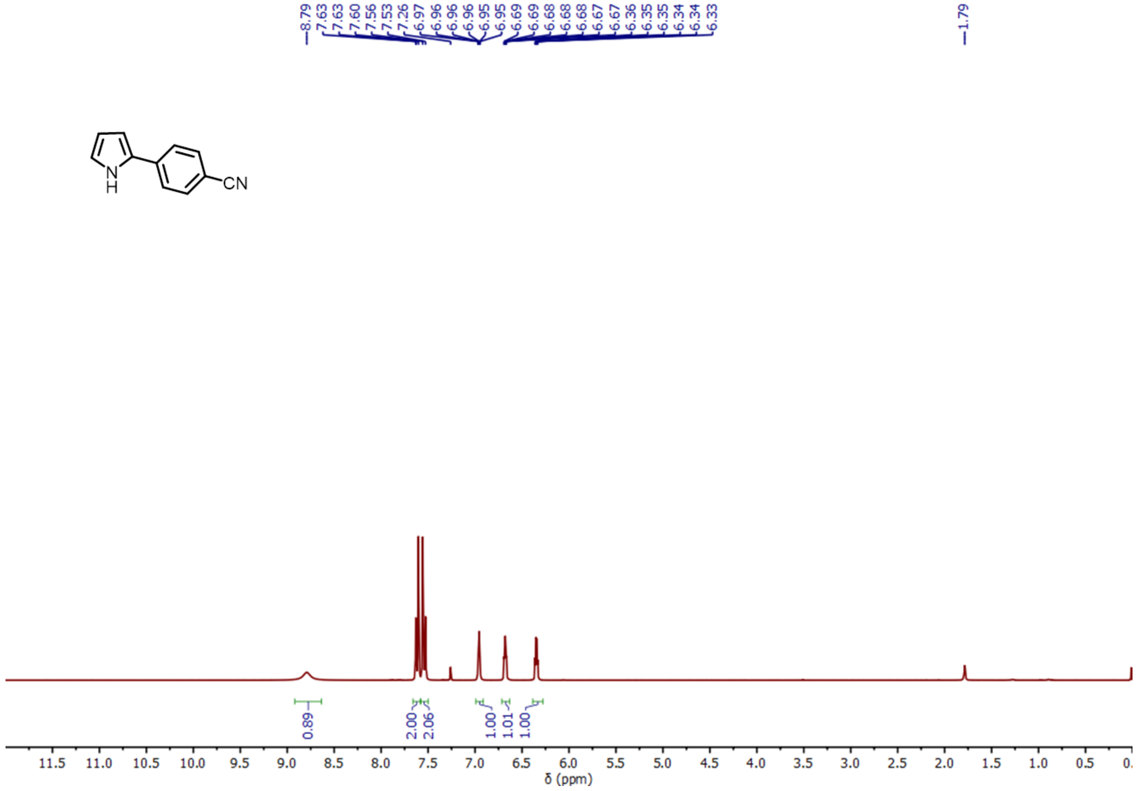

Fig. S77. ^{1}H NMR spectrum (300 MHz, $CDCl_3$) of **15f.**

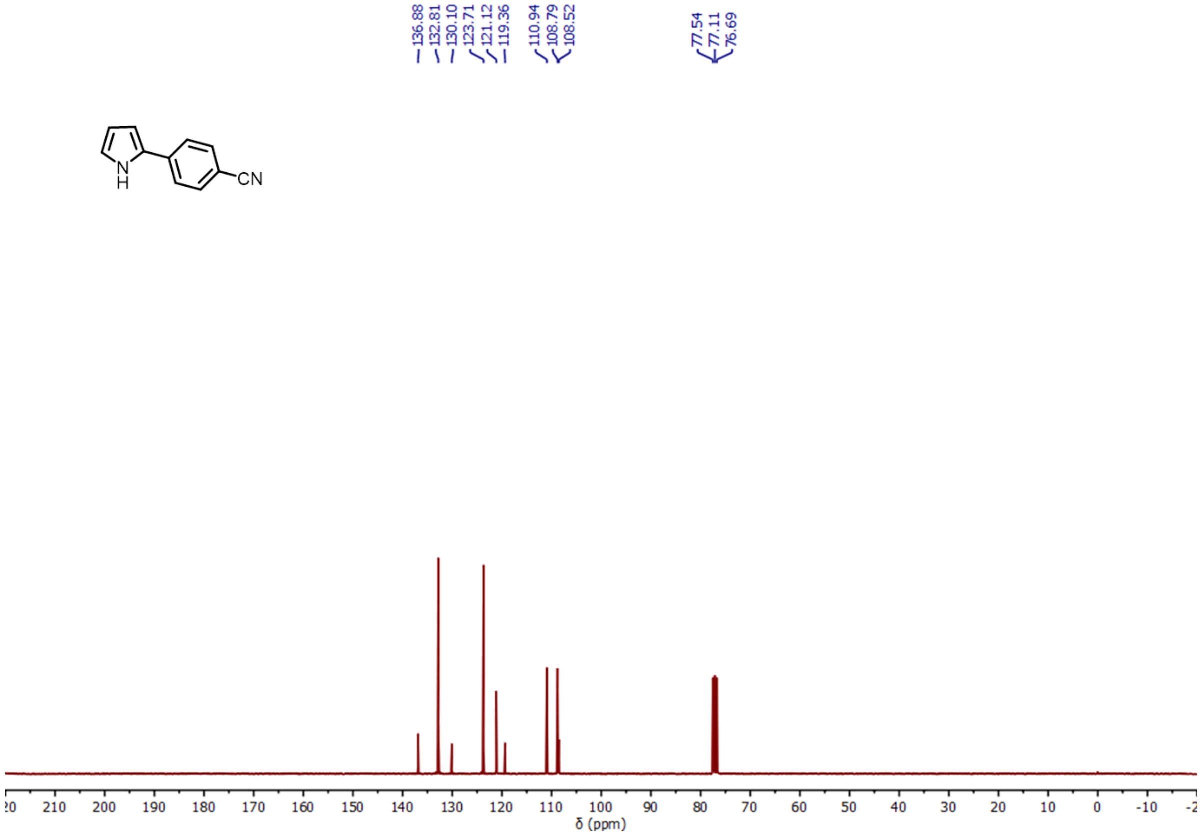

Fig. S78. ^{13}C NMR spectrum (75 MHz, $CDCl_3$) of 15f.

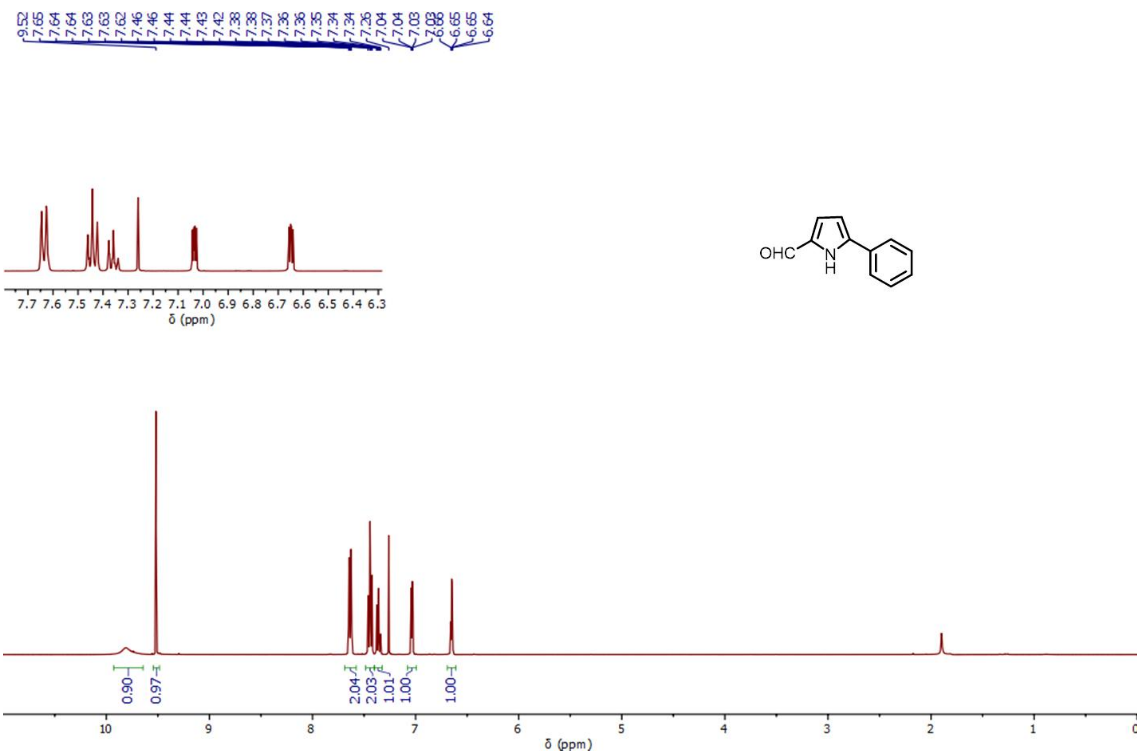

Fig. S79. ^{1}H NMR spectrum (400 MHz, $CDCl_3$) of **16a.**

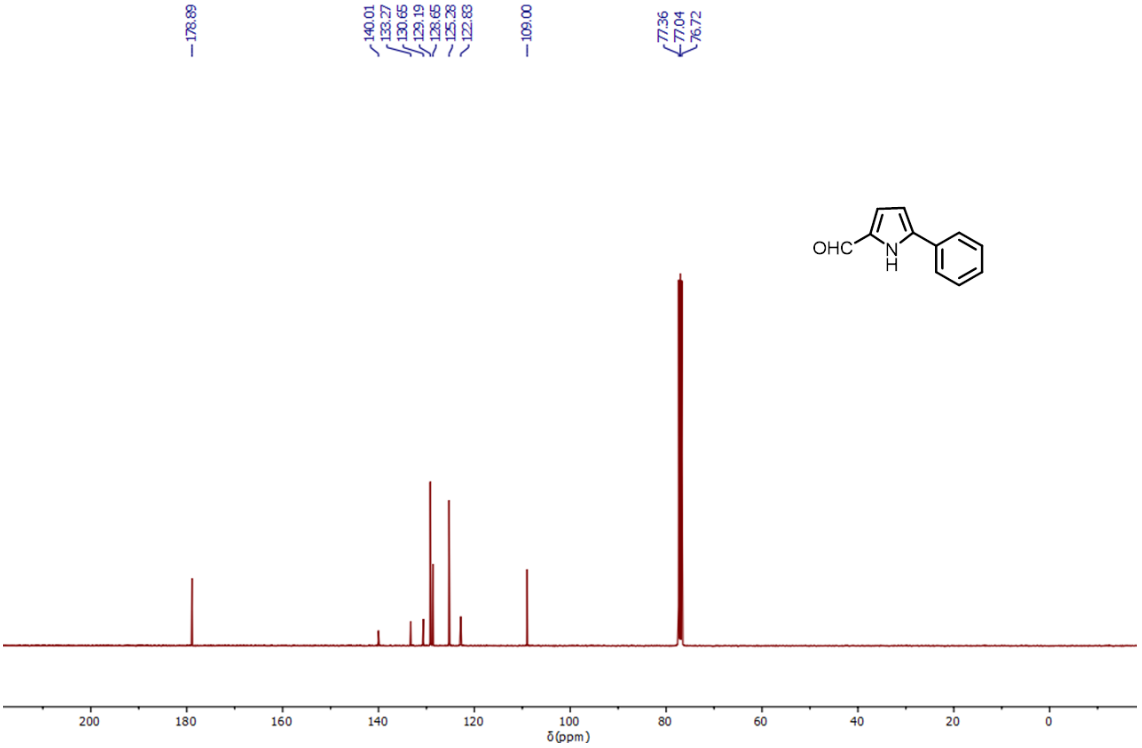

Fig. S80. ^{13}C NMR spectrum (100 MHz, $CDCl_3$) of **16a.**

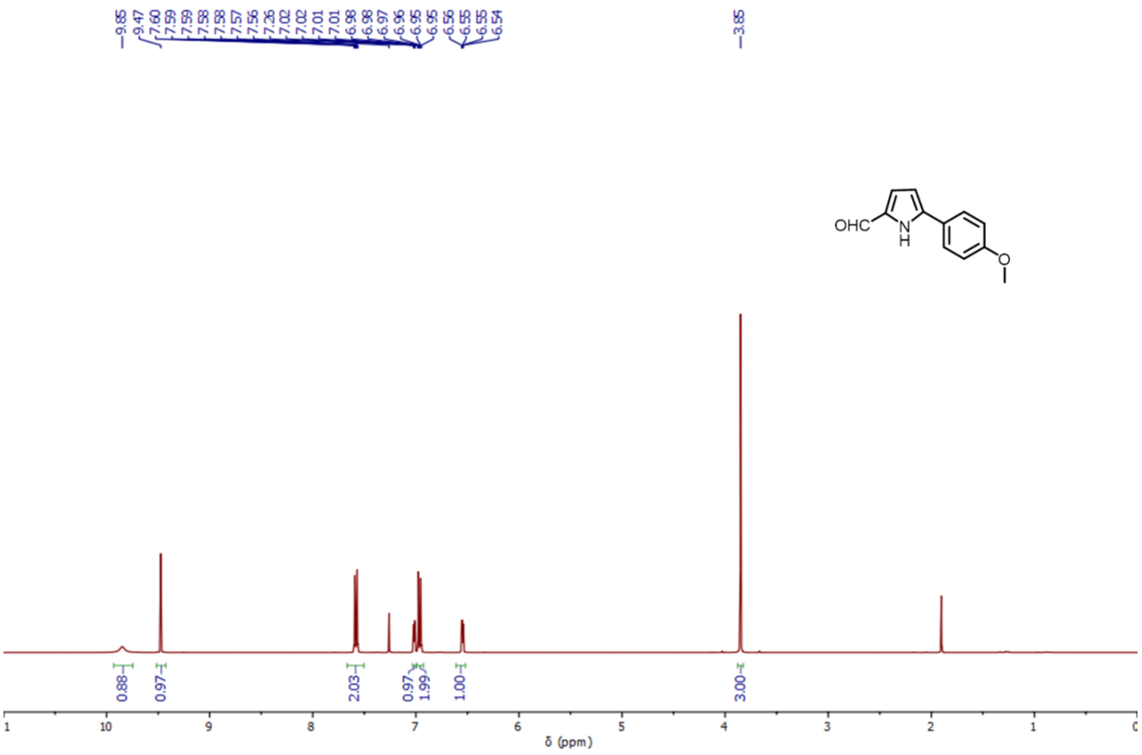

Fig. S81. ^{1}H NMR spectrum (400 MHz, $CDCl_3$) of **16b.**

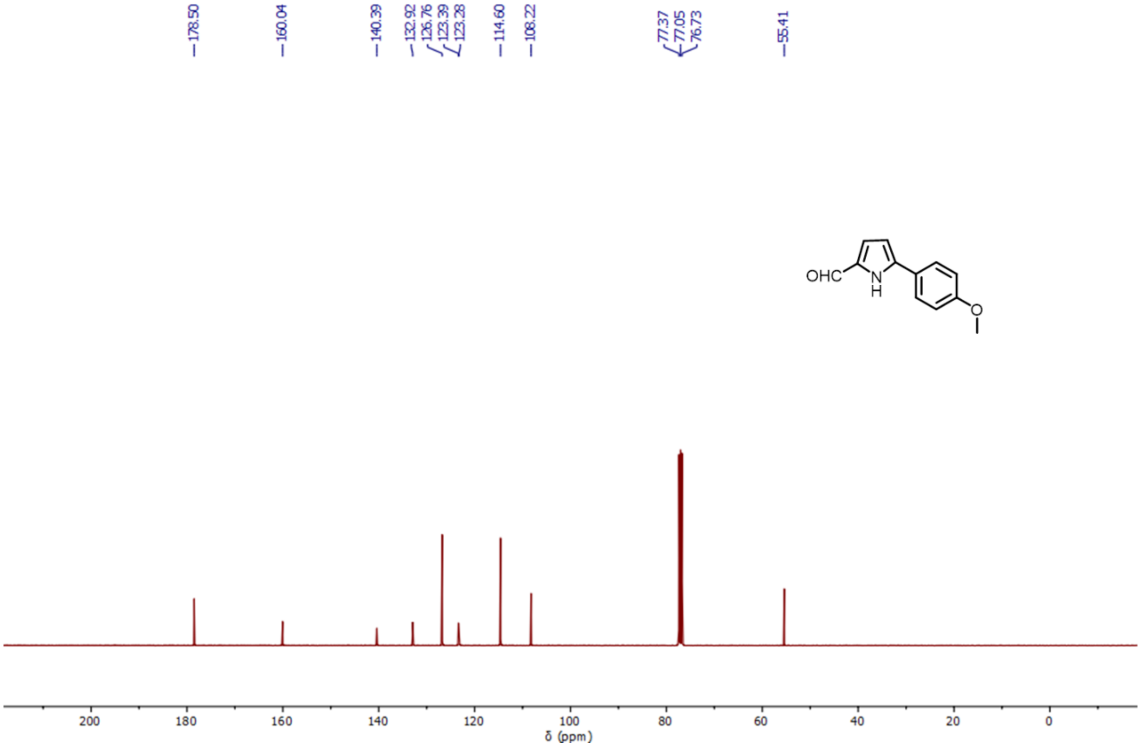

Fig. S82. ^{13}C NMR spectrum (100 MHz, $CDCl_3$) of **16b.**

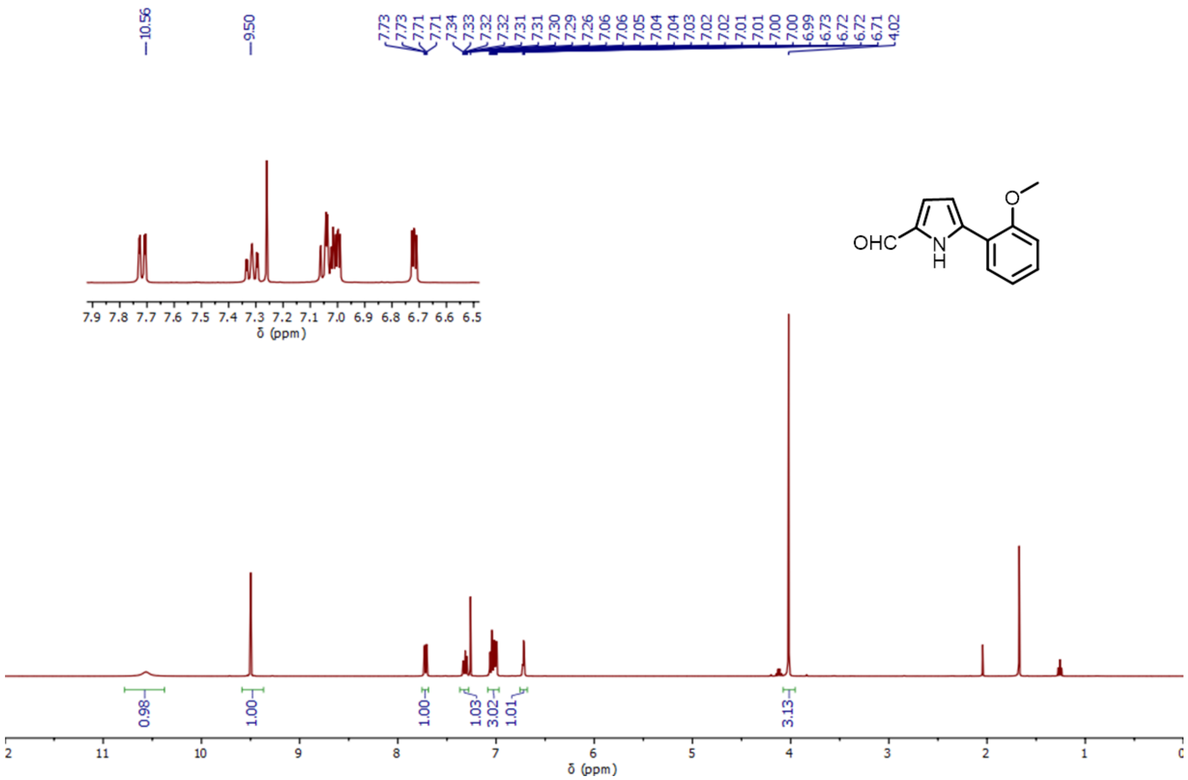

Fig. S83. ^{1}H NMR spectrum (400 MHz, $CDCl_3$) of **16c.**

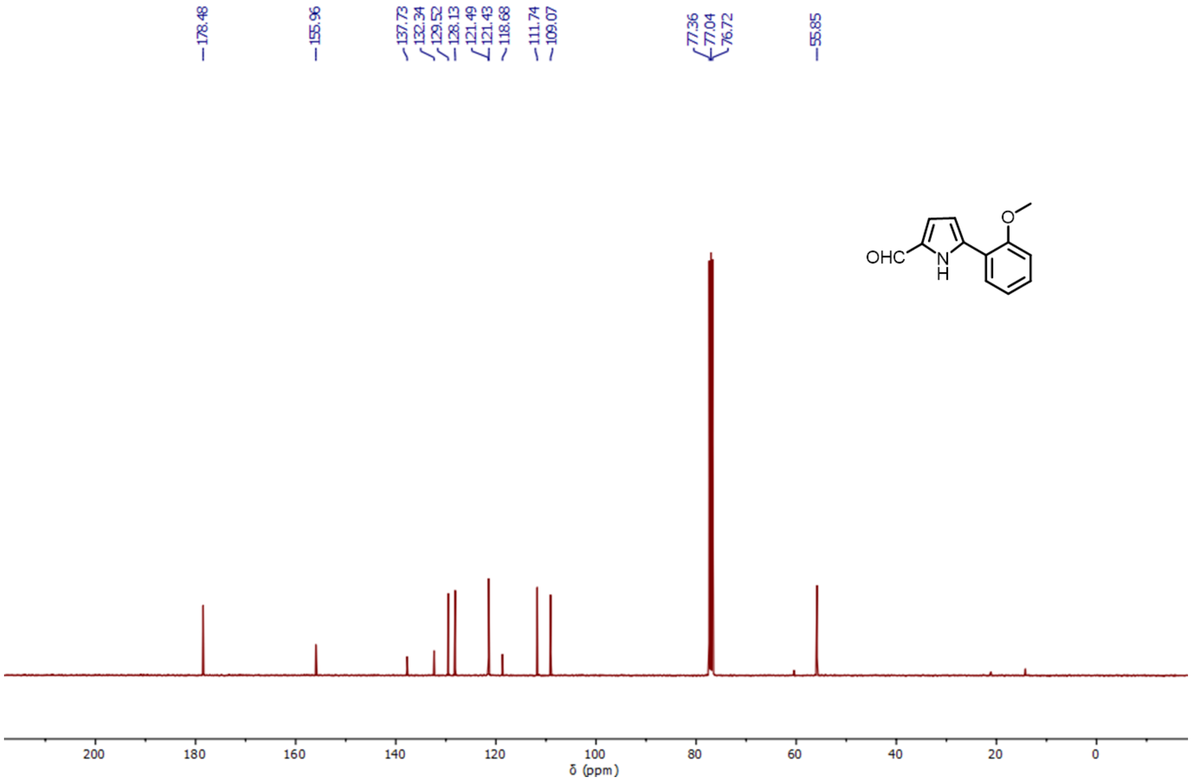

Fig. S84. ^{13}C NMR spectrum (100 MHz, $CDCl_3$) of **16c.**

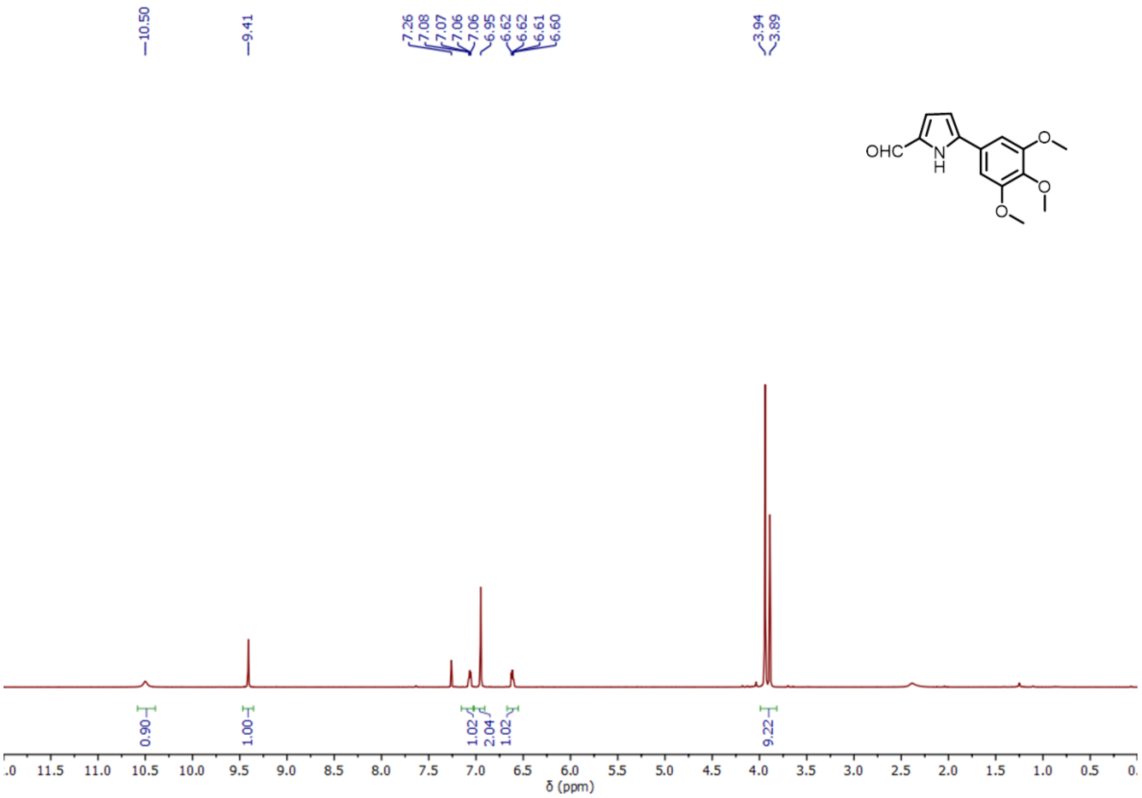

Fig. S85. ^{1}H NMR spectrum (300 MHz, $CDCl_3$) of **16d.**

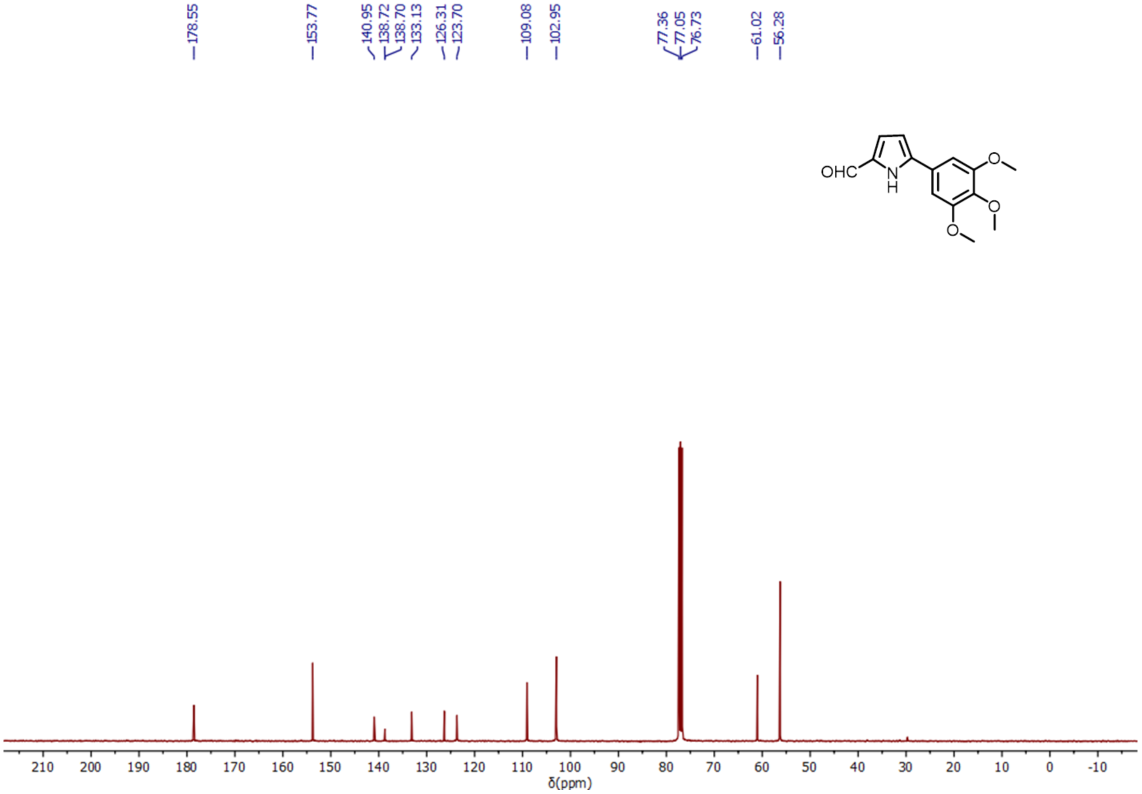

Fig. S86. ^{13}C NMR spectrum (100 MHz, $CDCl_3$) of **16d.**

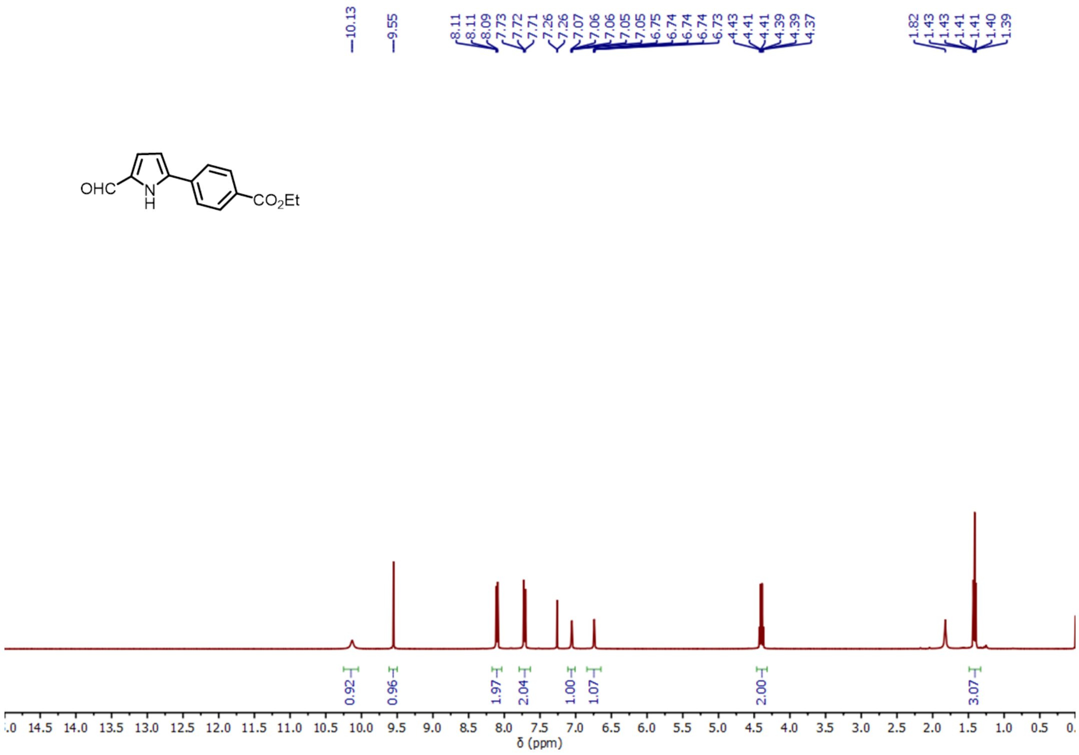

Fig. S87. ^{1}H NMR spectrum (400 MHz, $CDCl_3$) of **16e.**

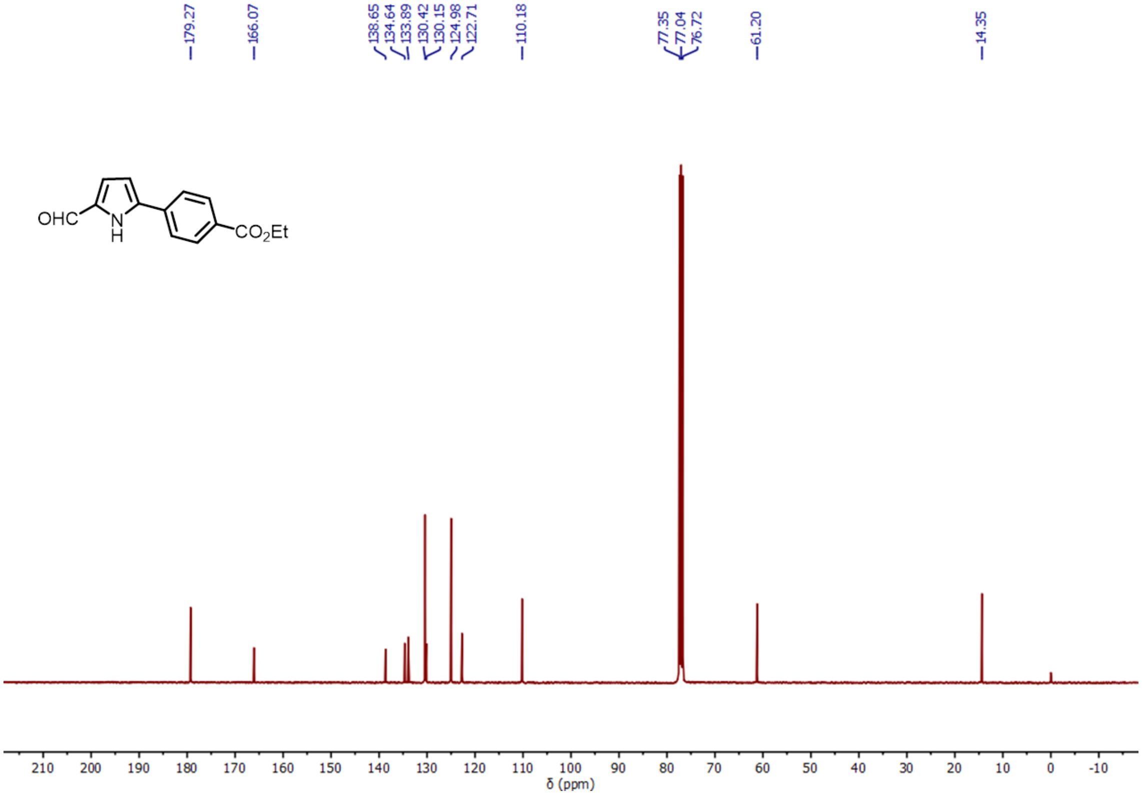

Fig. S88. ^{13}C NMR spectrum (100 MHz, $CDCl_3$) of **16e.**

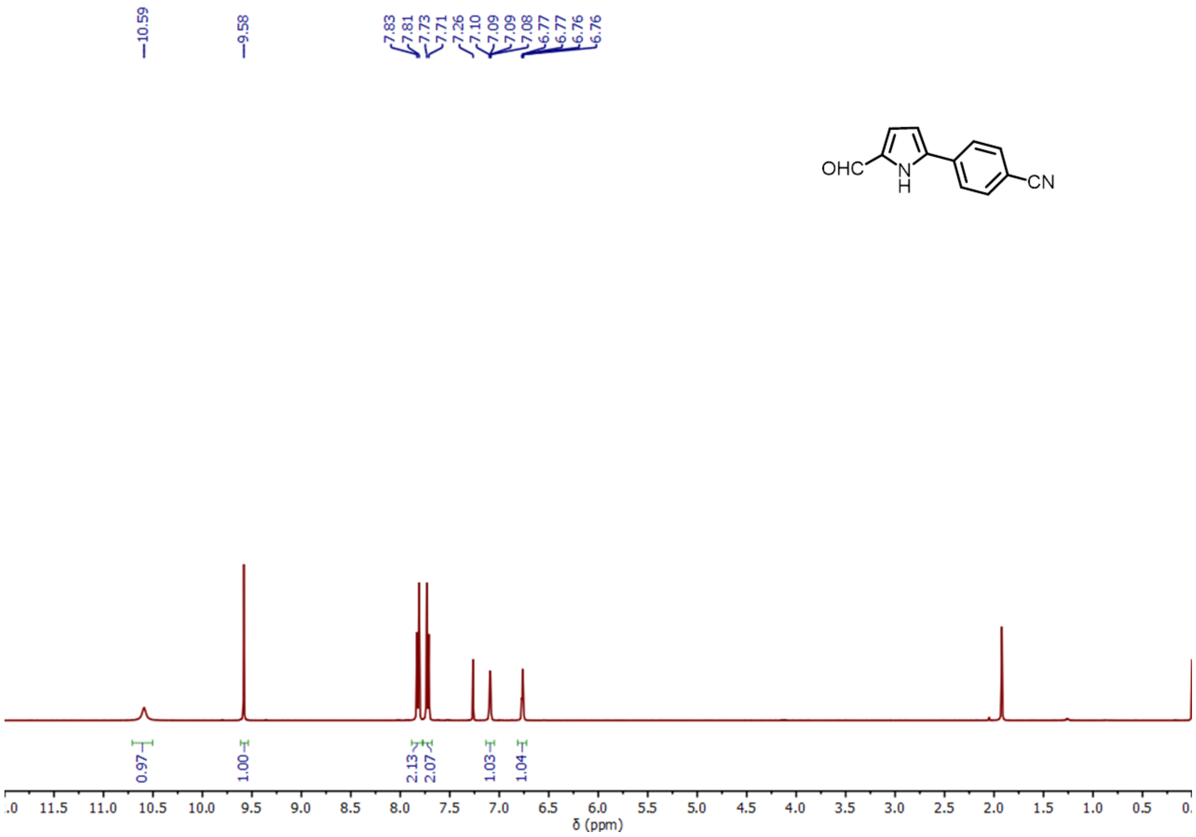

Fig. S89. ^{1}H NMR spectrum (400 MHz, $CDCl_3$) of **16f.**

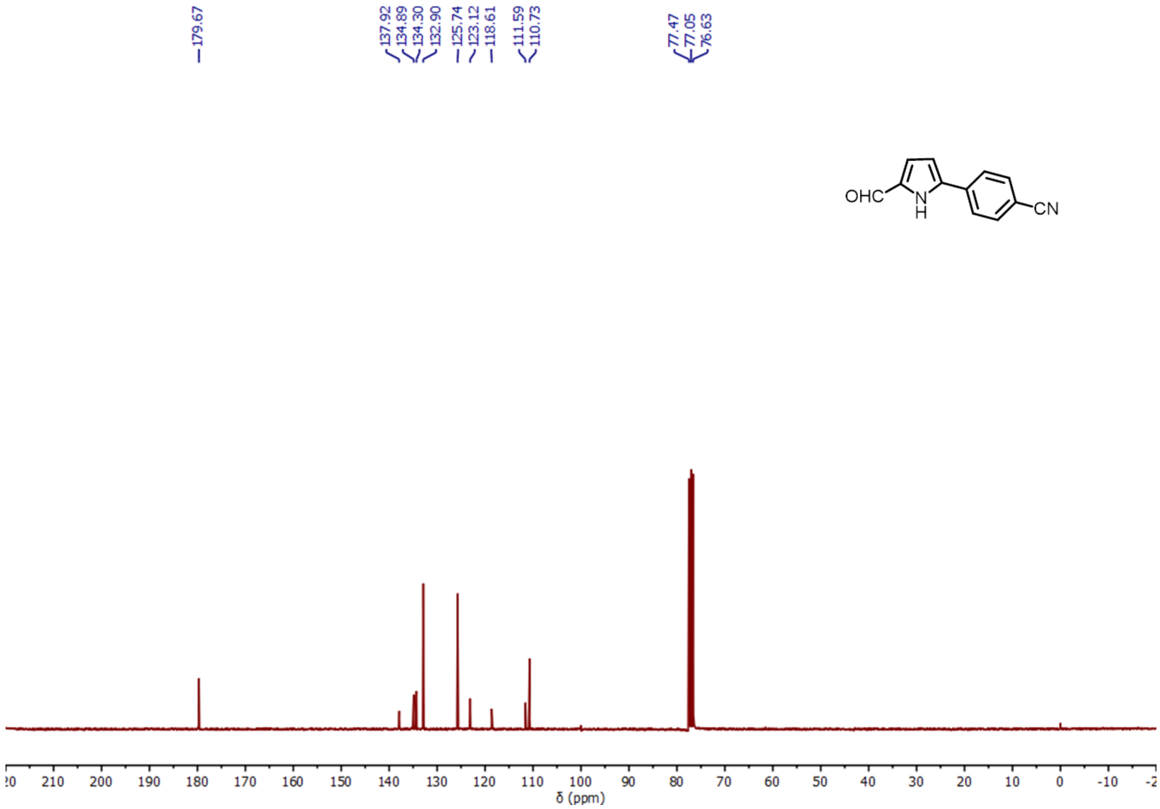

Fig. S90. ^{13}C NMR spectrum (75 MHz, $CDCl_3$) of **16f.**

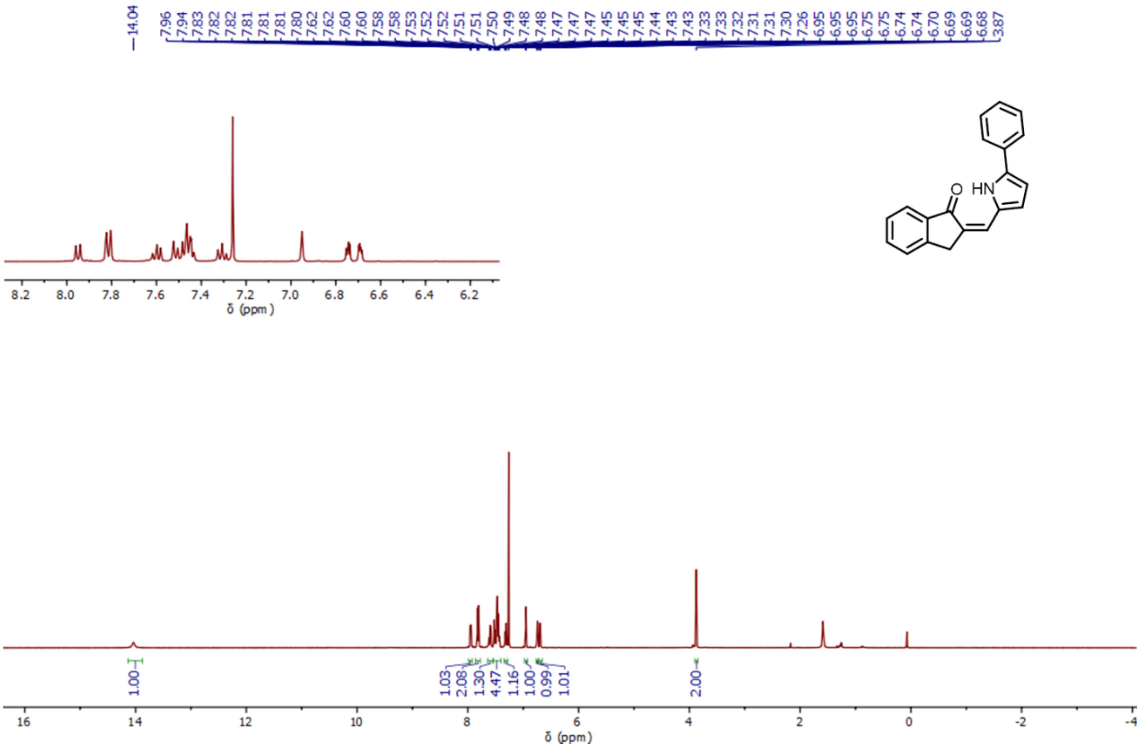

Fig. S91. ^{1}H NMR spectrum (400 MHz, $CDCl_3$) of **1.**

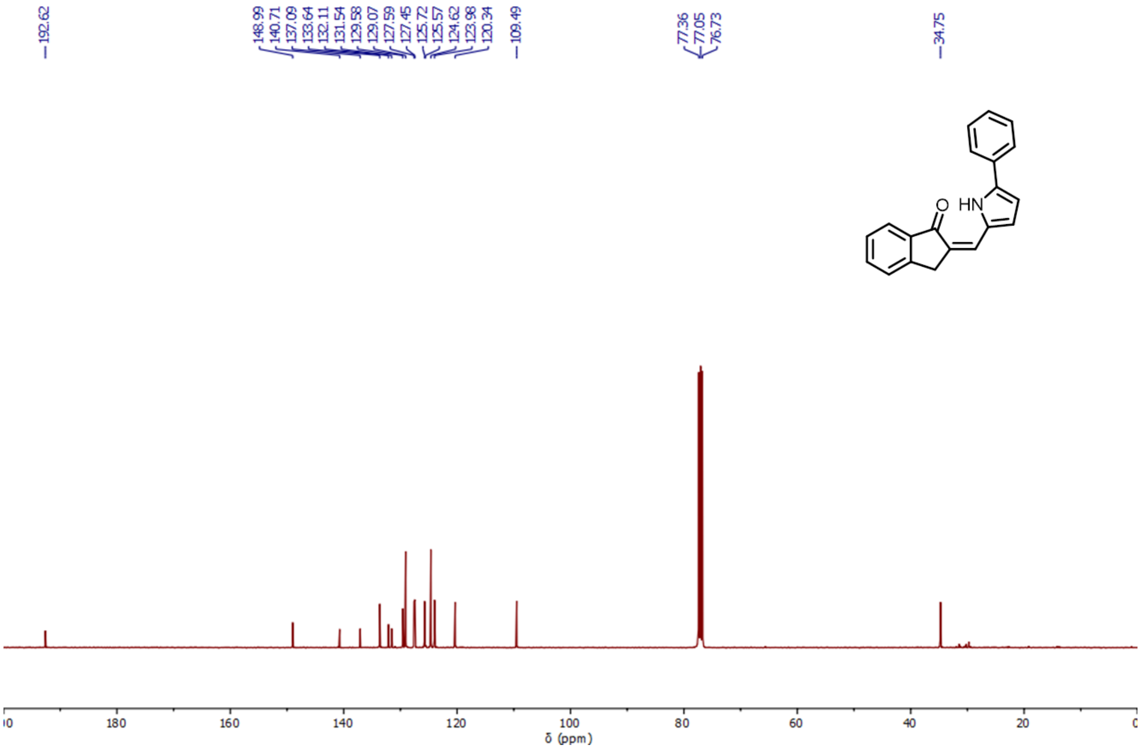

Fig. S92. ^{13}C NMR spectrum (100 MHz, $CDCl_3$) of **1.**

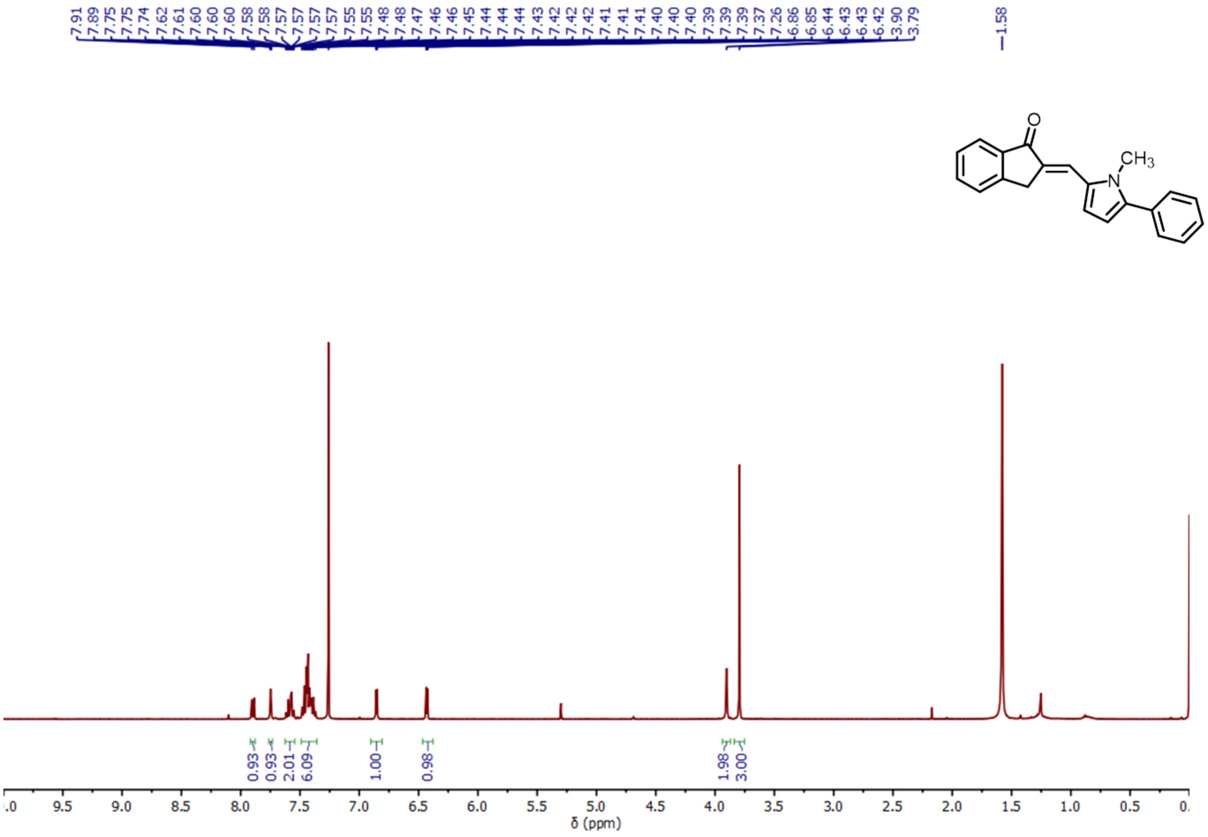

Fig. S93. ^{1}H NMR spectrum (400 MHz, $CDCl_3$) of **2.**

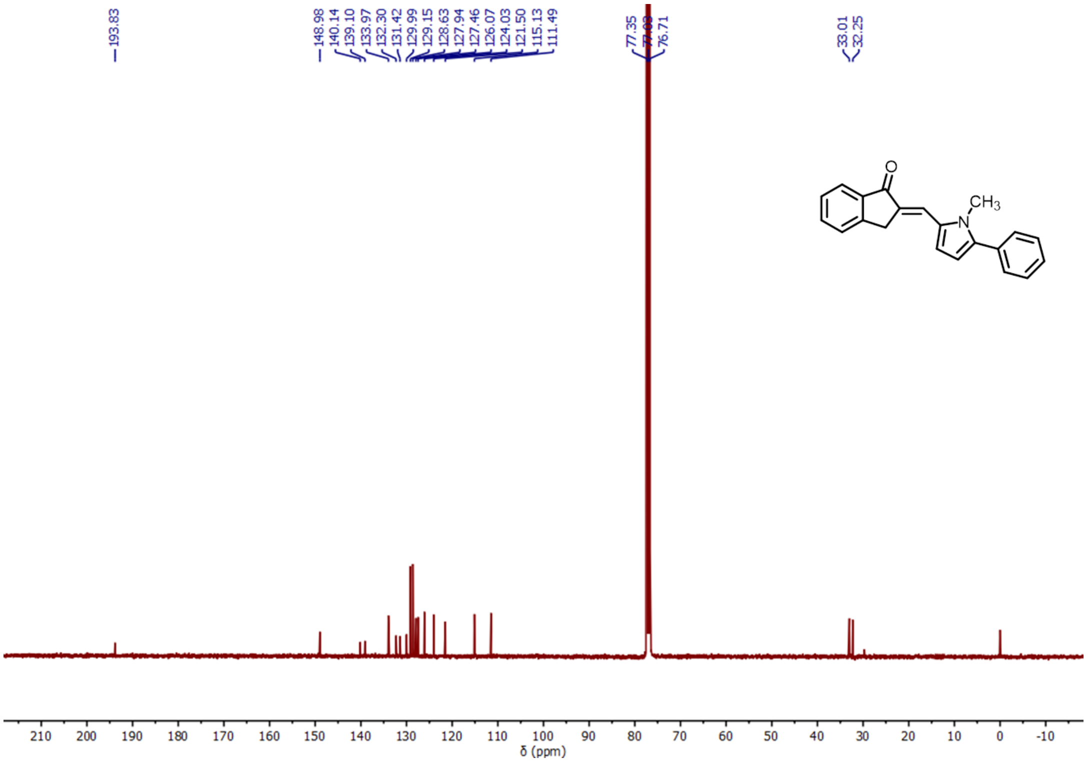

Fig. S94. ^{13}C NMR spectrum (100 MHz, $CDCl_3$) of **2.**

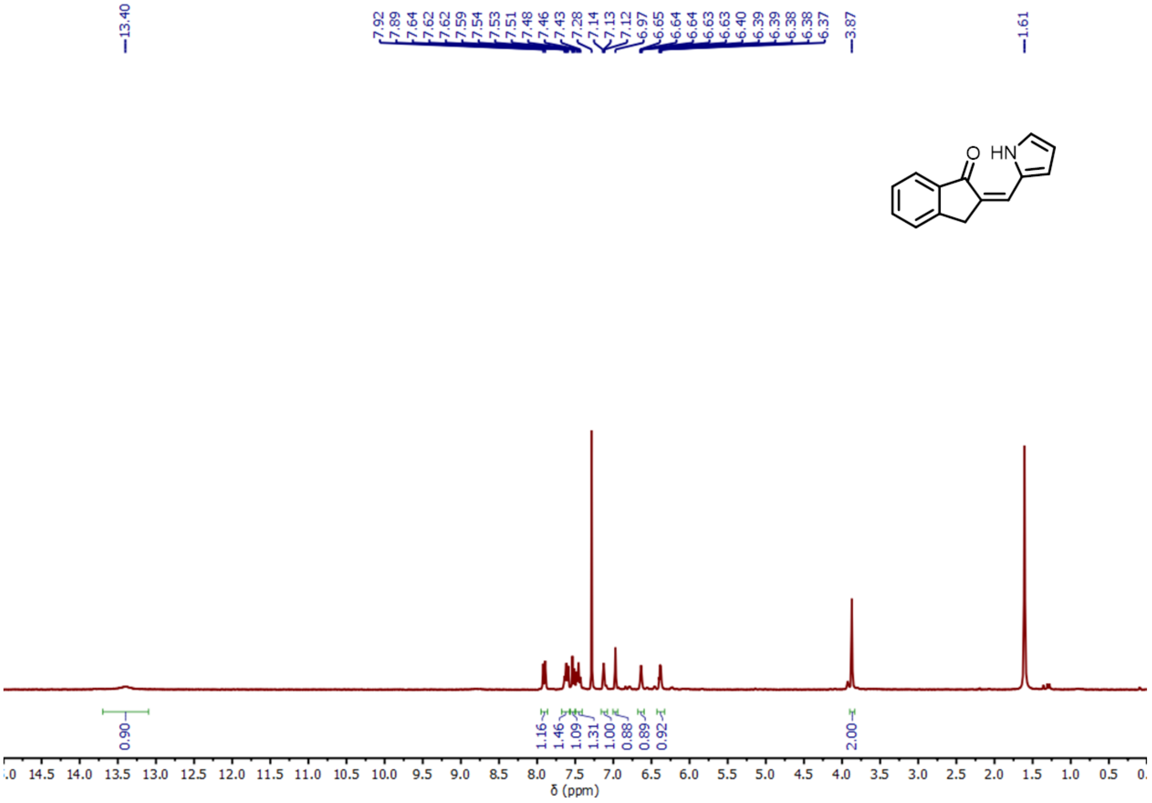

Fig. S95. ^{1}H NMR spectrum (300 MHz, $CDCl_3$) of **3.**

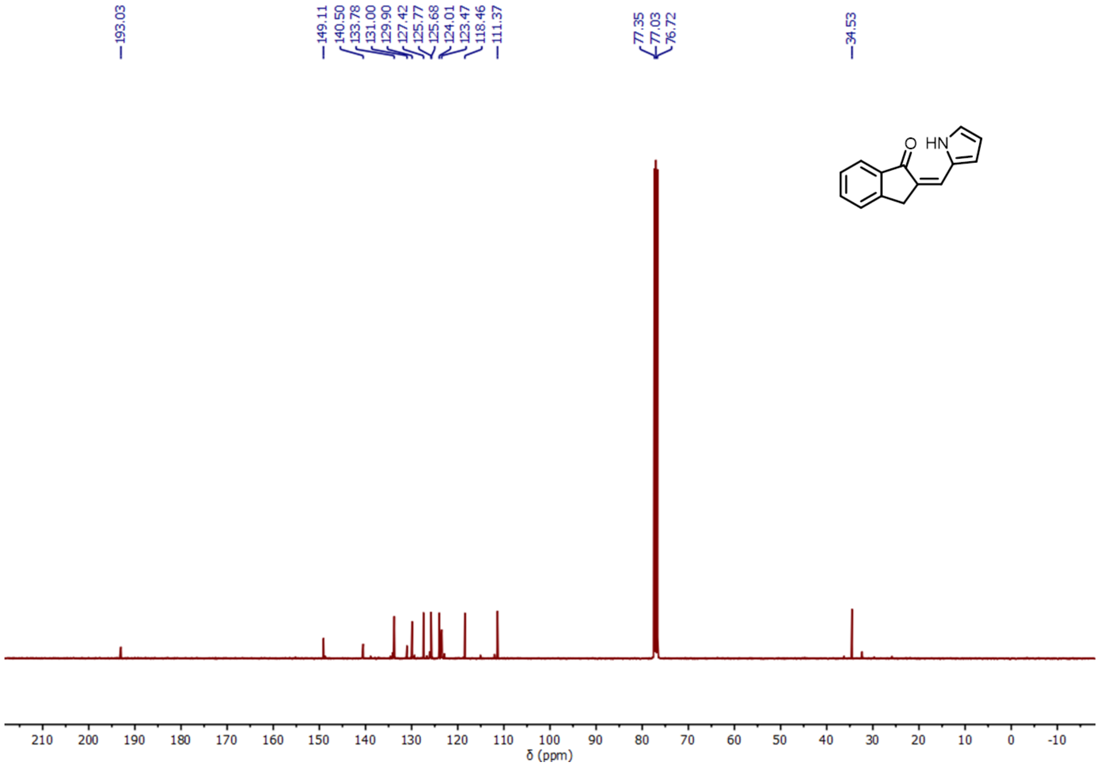

Fig. S96. ^{13}C NMR spectrum (100 MHz, $CDCl_3$) of **3.**

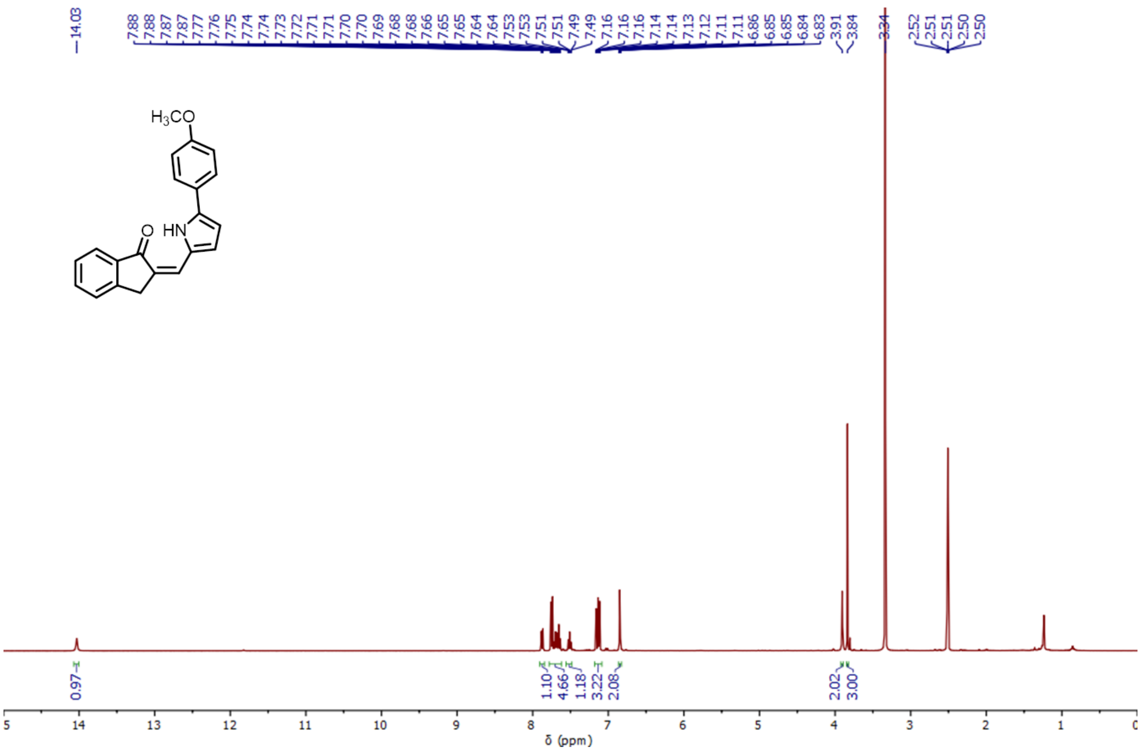

Fig. S97. ^{1}H NMR spectrum (400 MHz, DMSO-d_6) of **4.**

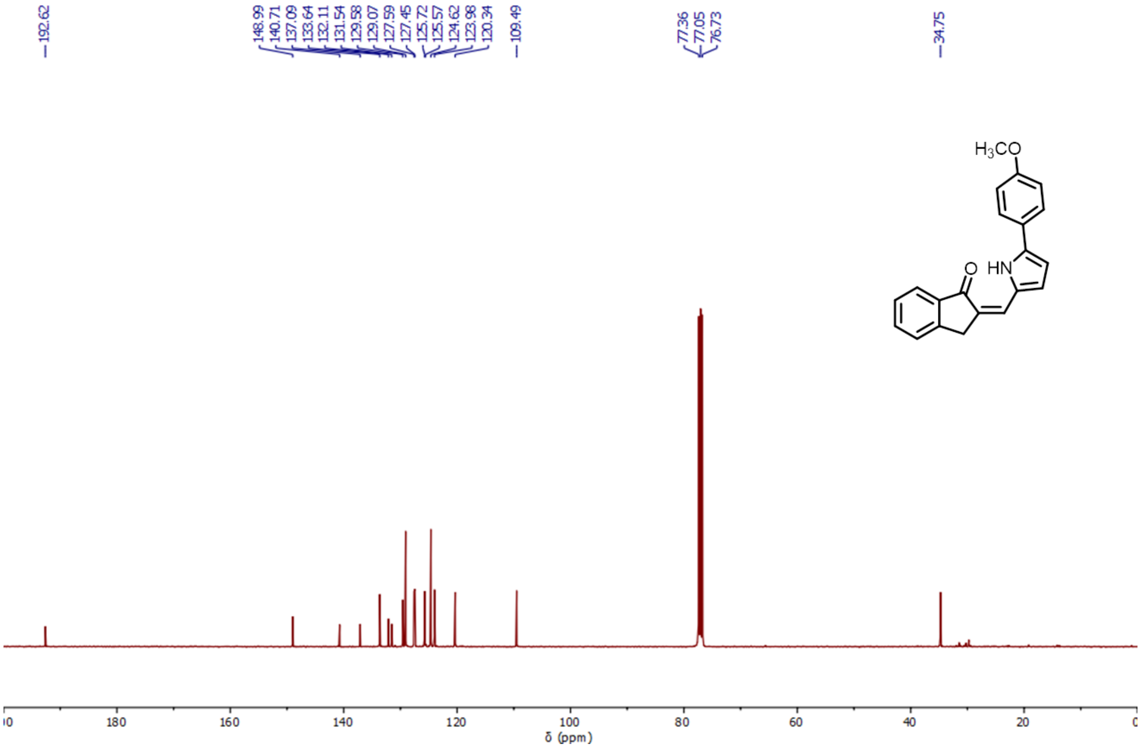

Fig. S98. ^{13}C NMR spectrum (75 MHz, $CDCl_3$) of **4.**

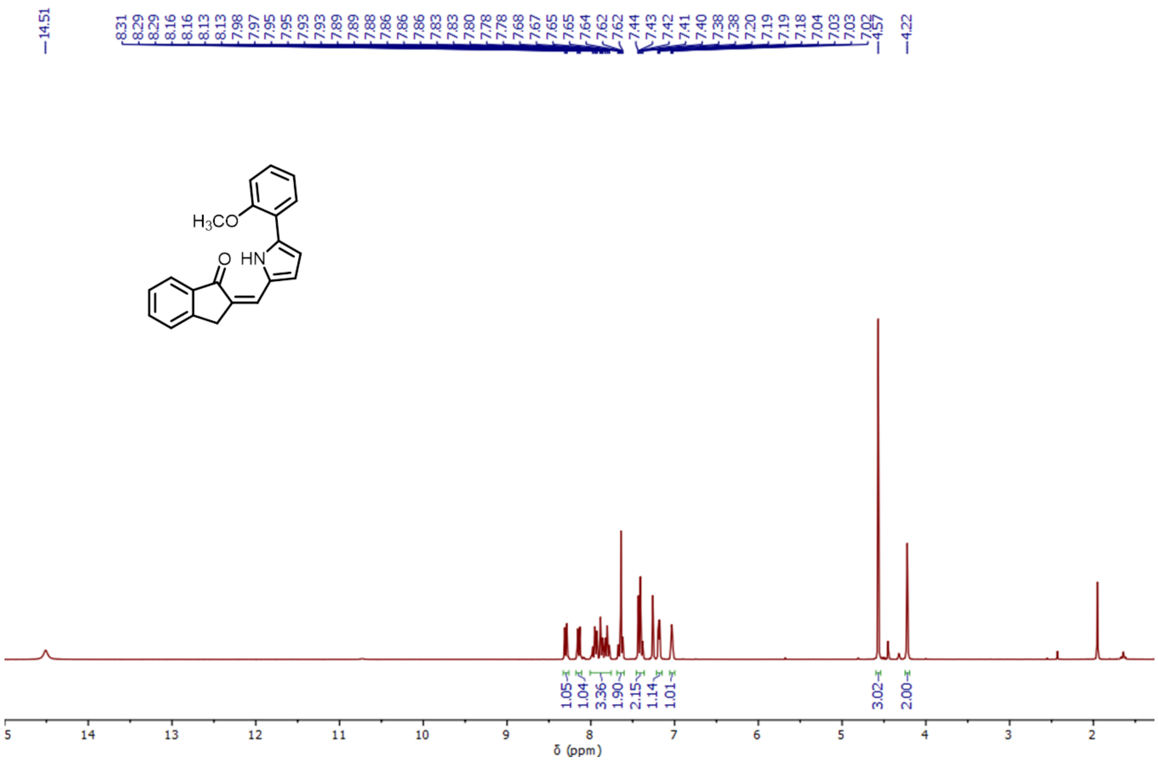

Fig. S99. ^{1}H NMR spectrum (300 MHz, $CDCl_3$) of **5.**

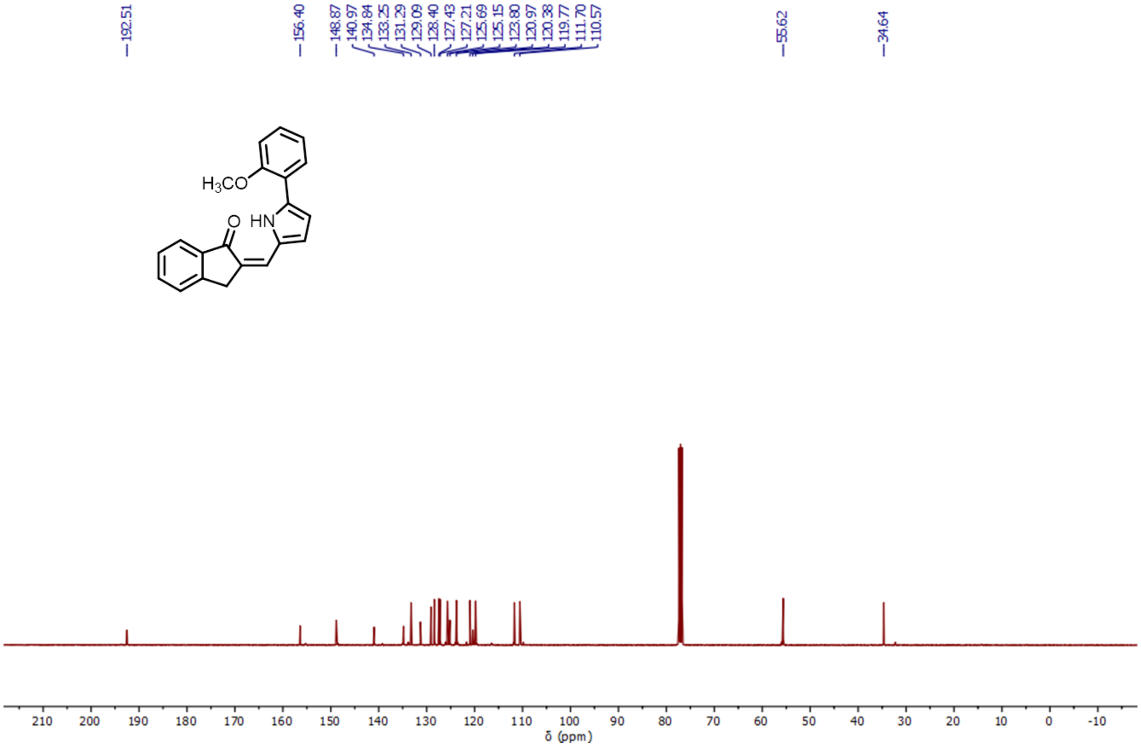

Fig. S100. ^{13}C NMR spectrum (100 MHz, $CDCl_3$) of **5.**

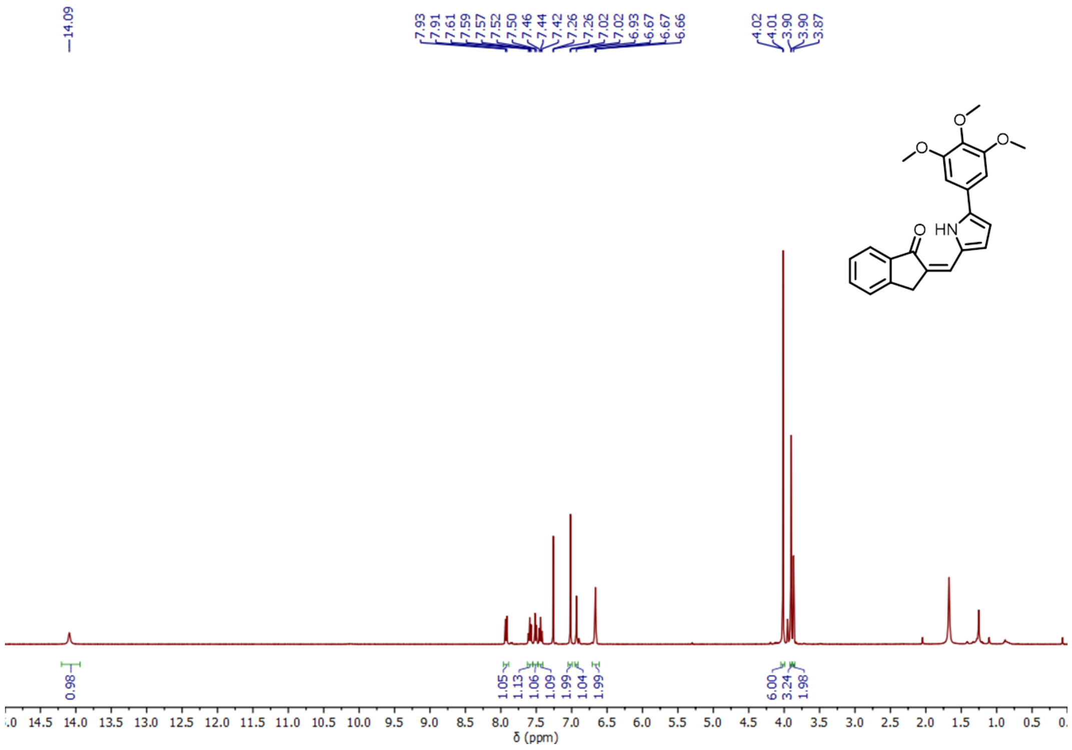

Fig. S101. ^{1}H NMR spectrum (400 MHz, $CDCl_3$) of **6.**

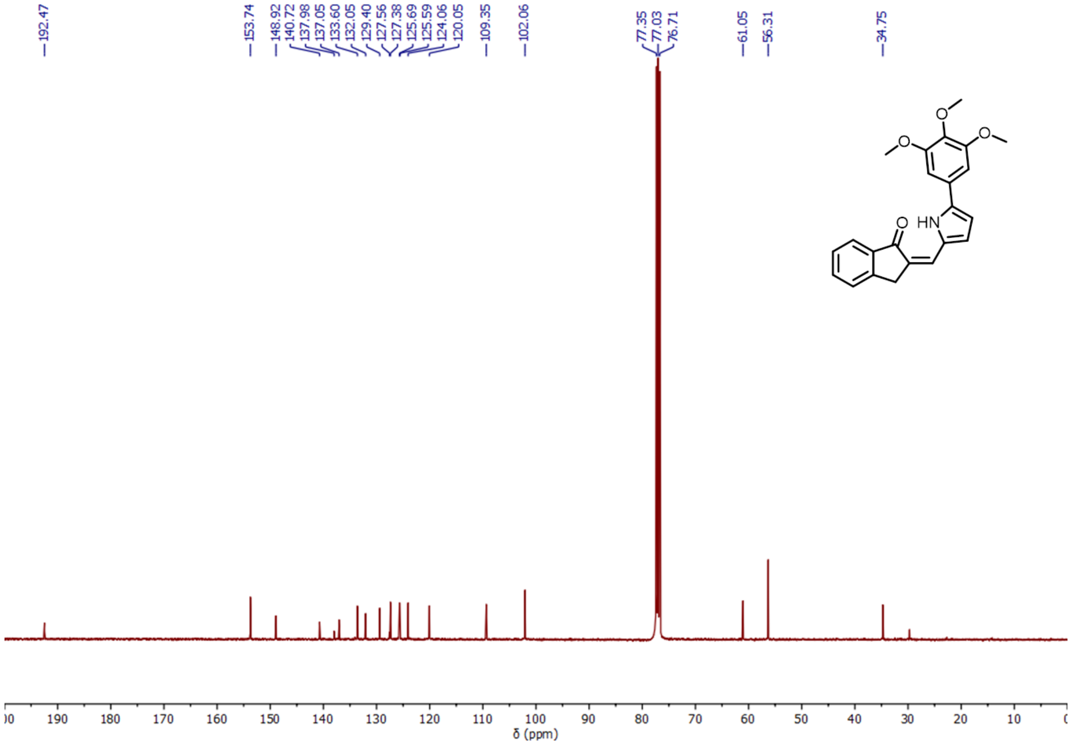

Fig. S102. ^{13}C NMR spectrum (100 MHz, $CDCl_3$) of **6.**

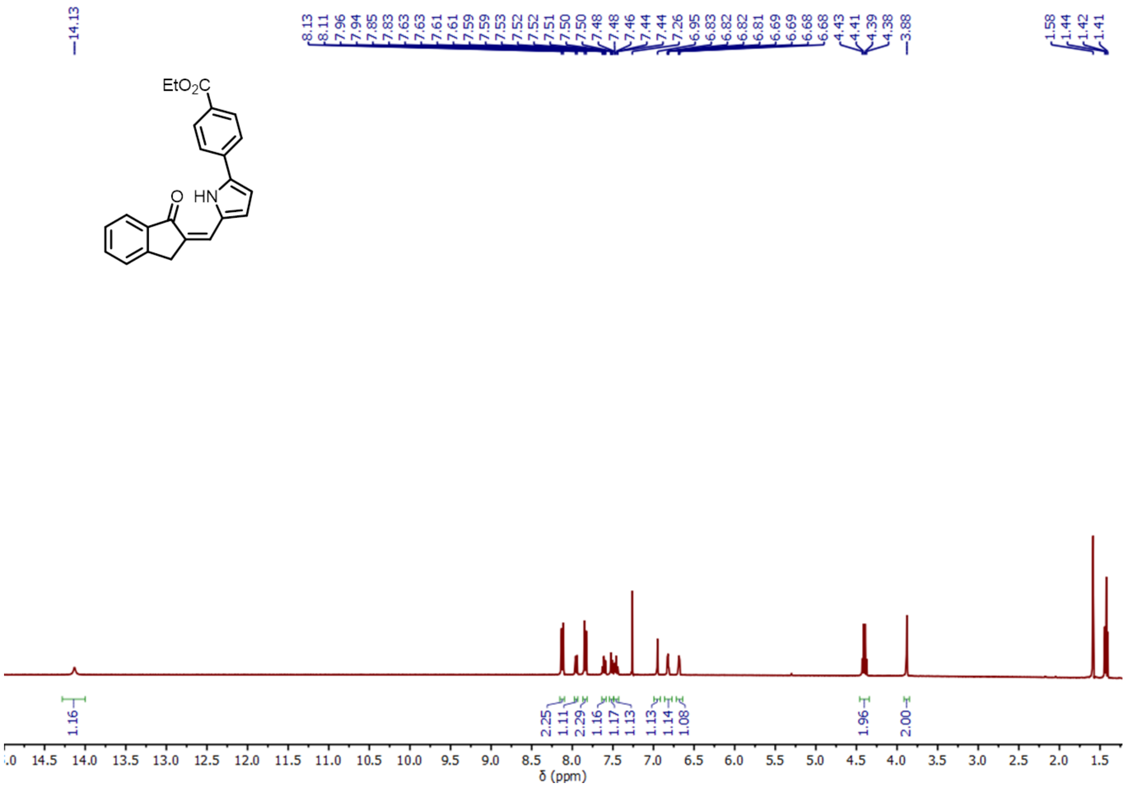

Fig. S103. ^{1}H NMR spectrum (400 MHz, $CDCl_3$) of **7.**

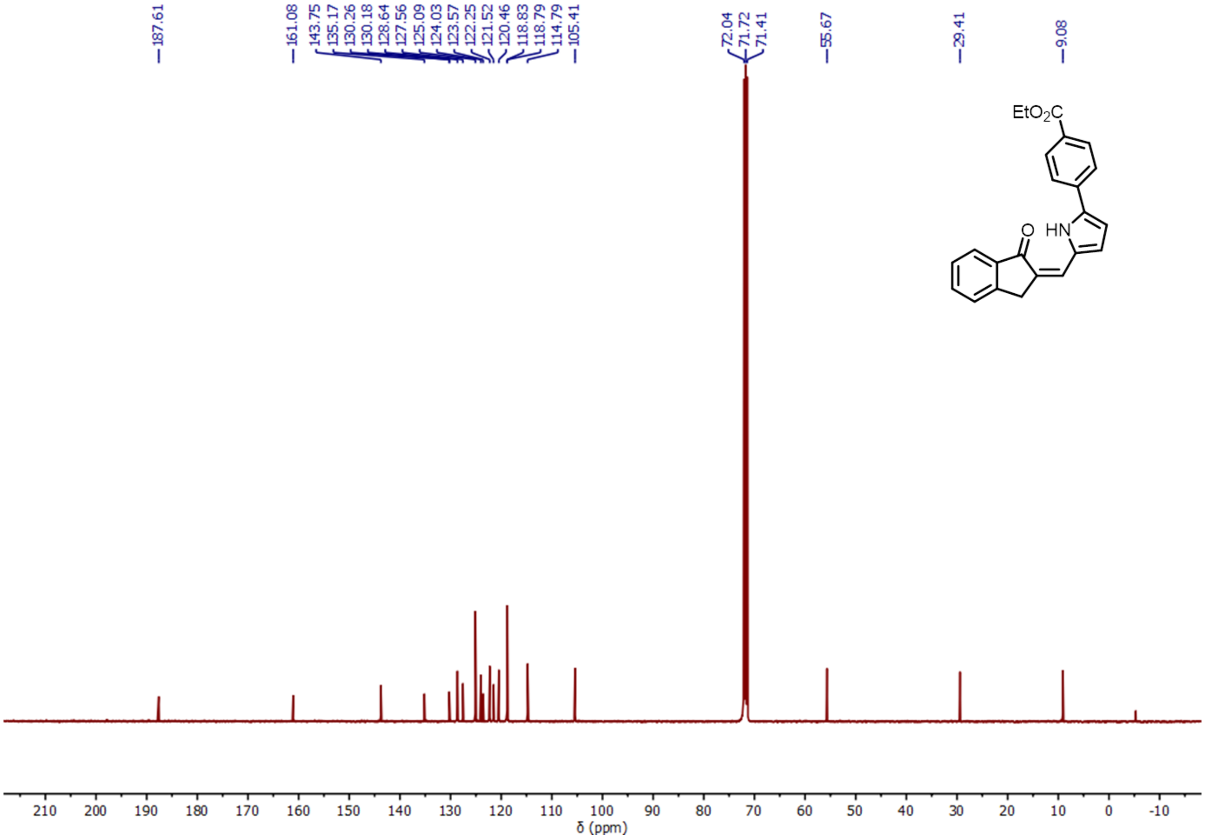

Fig. S104. ^{13}C NMR spectrum (100 MHz, $CDCl_3$) of **7.**

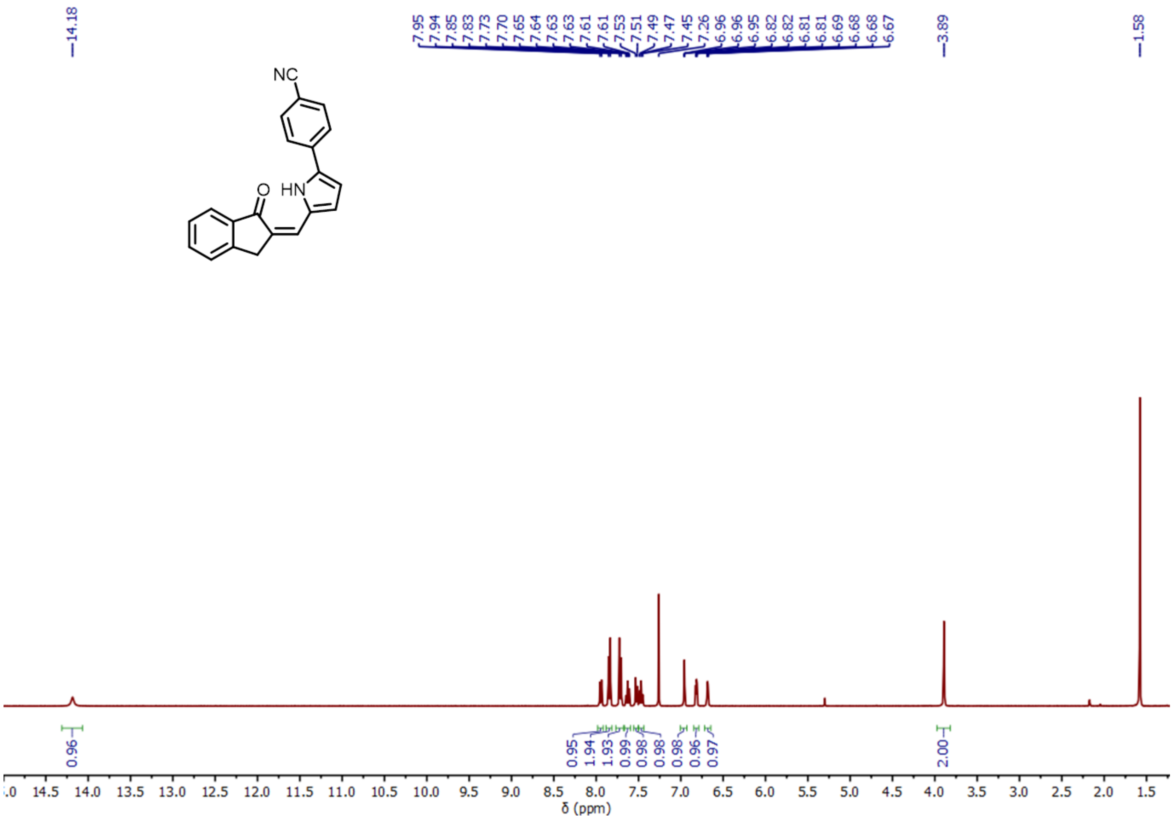

Fig. S105. ^{1}H NMR spectrum (400 MHz, $CDCl_3$) of **8.**

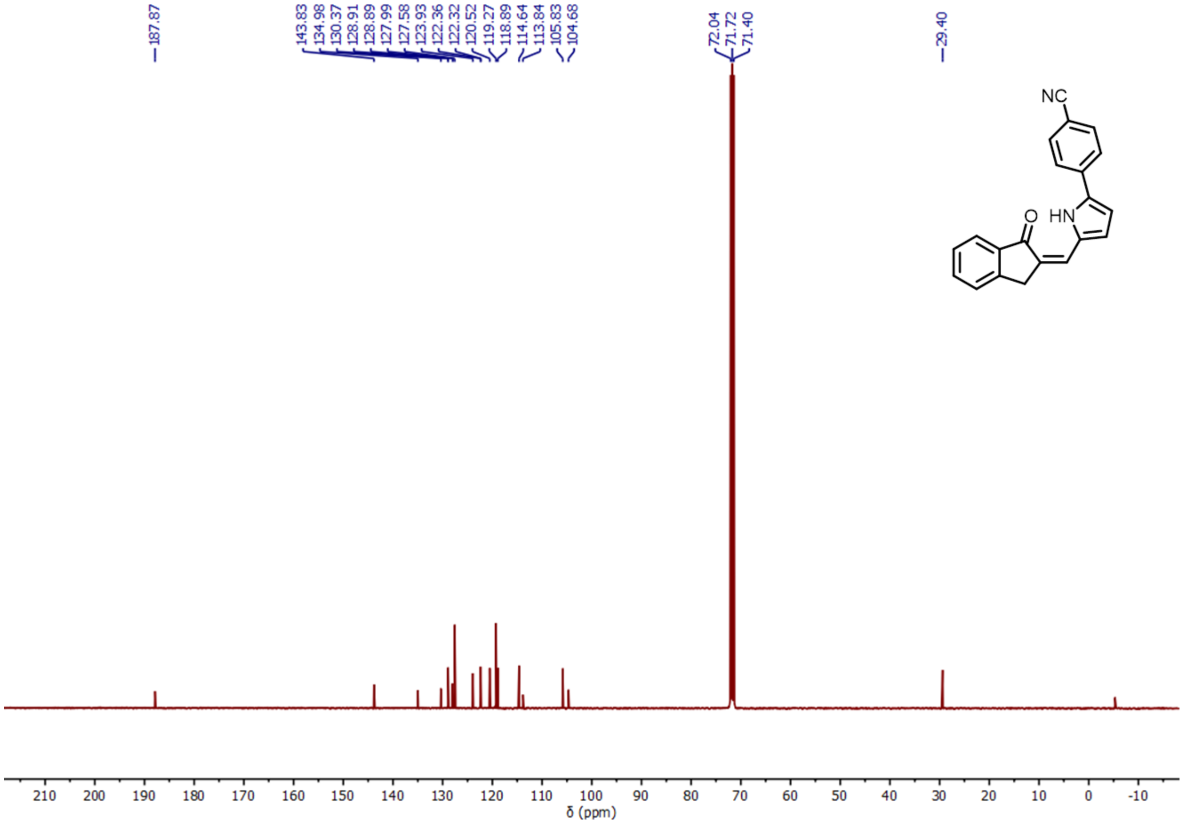

Fig. S106. ^{13}C NMR spectrum (100 MHz, $CDCl_3$) of **8.**

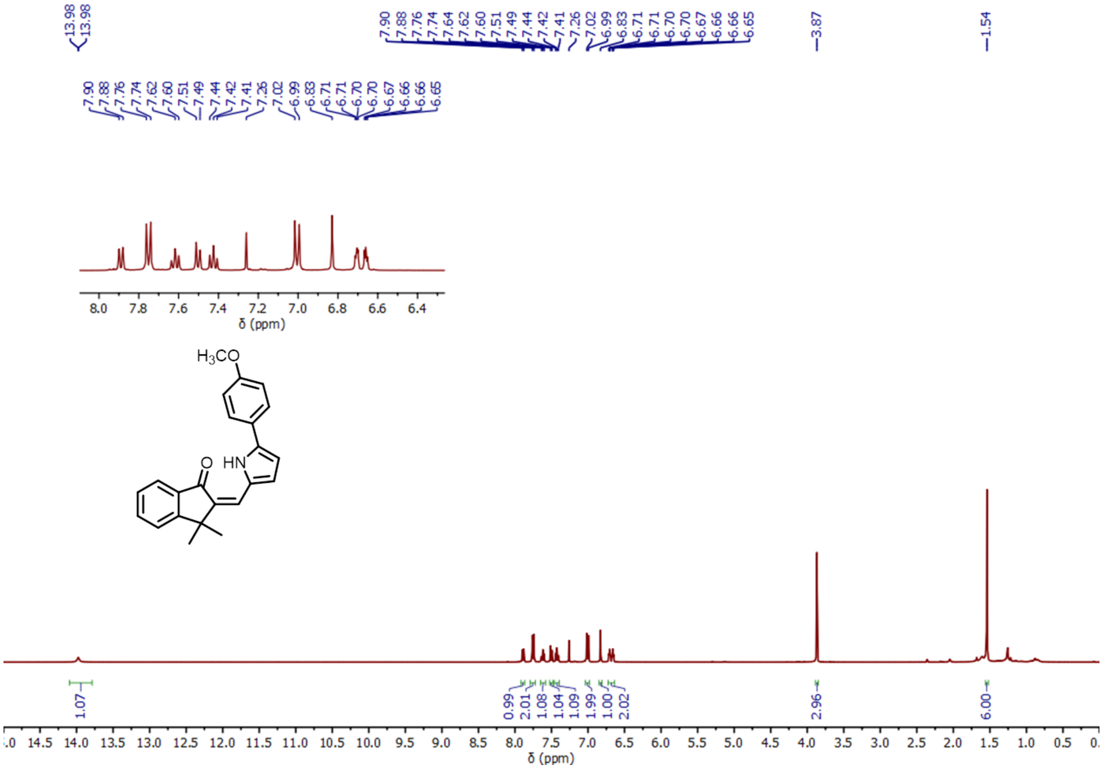

Fig. S107. ^{1}H NMR spectrum (400 MHz, $CDCl_3$) of **9.**

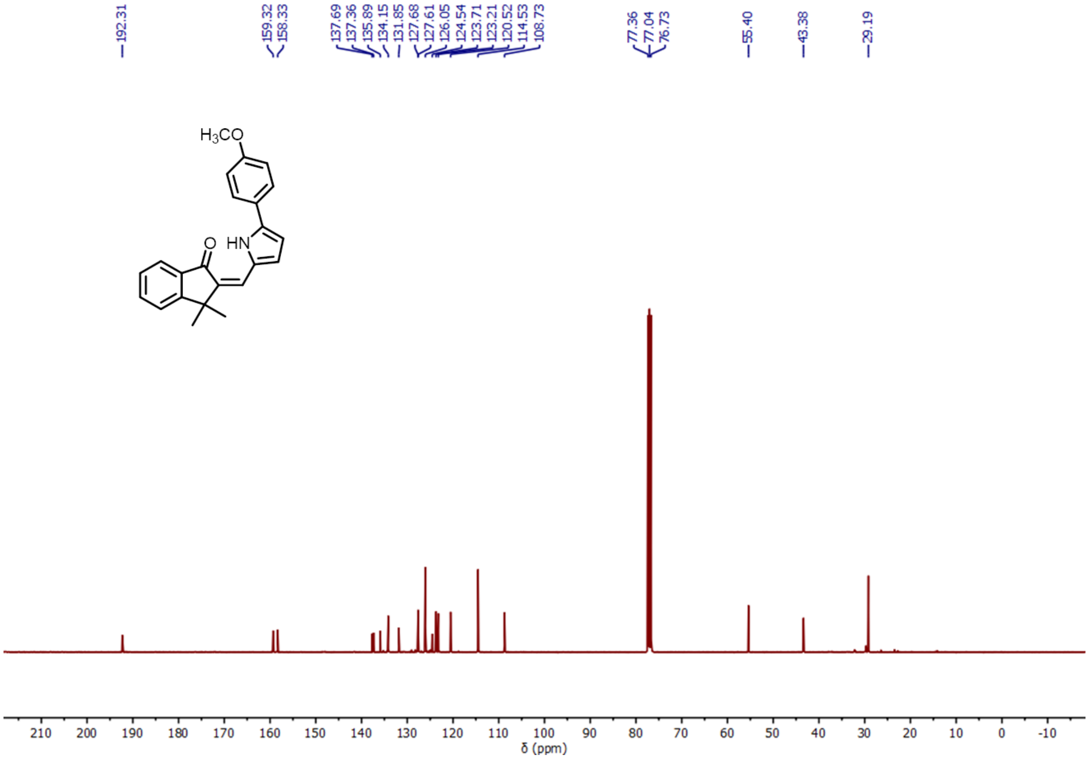

Fig. S108. ^{13}C NMR spectrum (100 MHz, $CDCl_3$) of **9.**

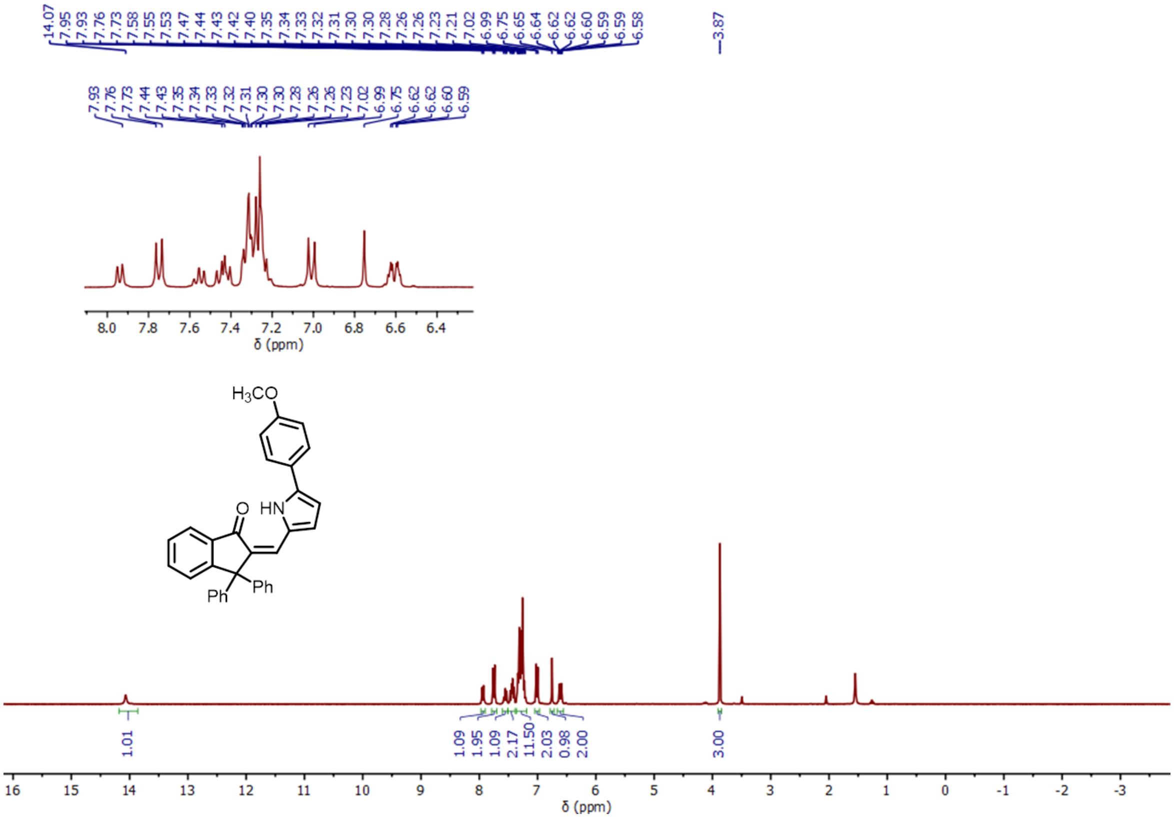

Fig. S109. ^{1}H NMR spectrum (300 MHz, $CDCl_3$) of **10.**

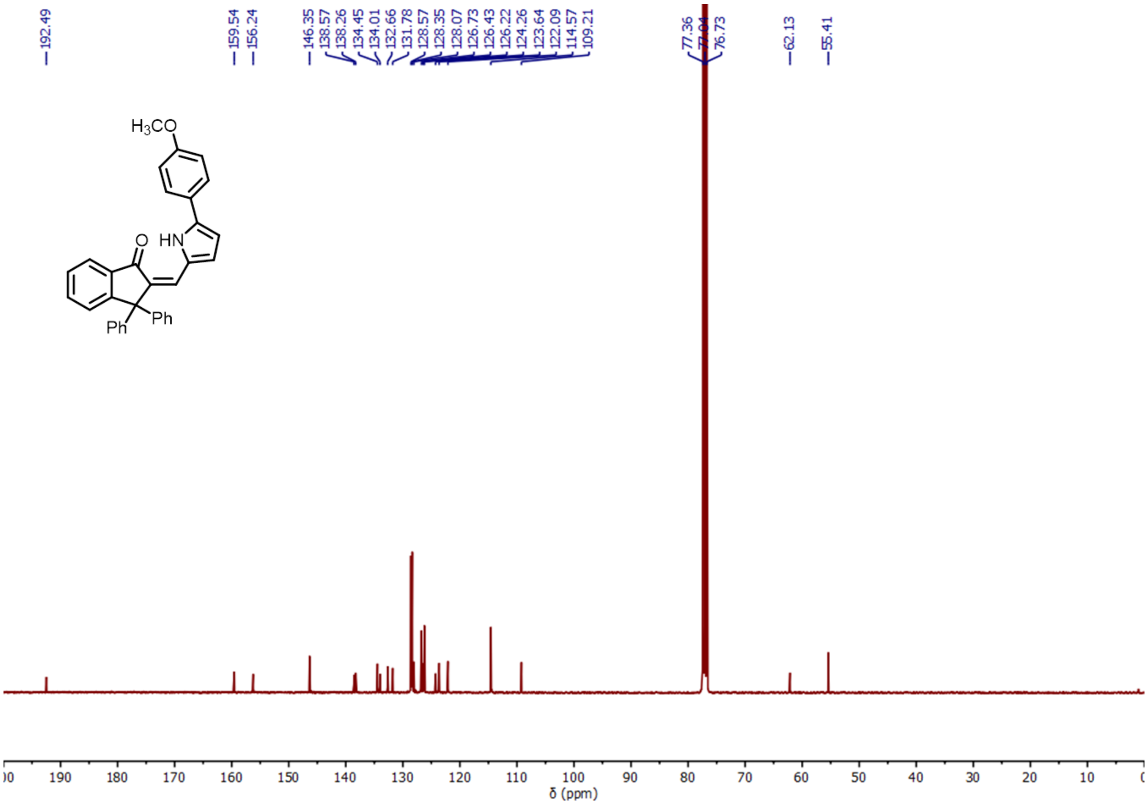

Fig. S110. ^{13}C NMR spectrum (100 MHz, $CDCl_3$) of **10.**

Table S4. Cartesian coordinates:

**1-(*Z*-i)**	**1-(*E*-i)**	**1-(*E*-ii)**
C -2.2577 -1.04416 0.56662	C -4.21334 1.41009 0.52529	C 6.63049 -0.06263 -0.02421
C -1.88019 0.19328 -0.21679	C -4.08166 0.19362 1.1497	C 6.26457 -1.41138 0.10024
C 0.15201 -0.79583 0.74719	C -2.78194 -0.63543 0.98774	C 4.92054 -1.79416 0.1477
C -0.37106 0.38855 -0.09014	C -1.80536 -0.12251 0.21618	C 3.93965 -0.80778 0.06889
O 0.31338 1.31725 -0.59277	C -1.91558 1.26369 -0.37861	C 4.31162 0.53605 -0.05611
C 2.54969 -0.31787 0.18339	C -3.04181 1.9939 -0.3019	C 5.65087 0.92509 -0.10379
N 2.30387 0.86956 -0.67125	C 0.37249 0.43041 -0.70698	C 1.93122 0.47139 -0.02887
H 1.60654 1.48128 -0.29772	C -0.56492 1.62021 -0.99527	C 3.09897 1.39438 -0.12281
C -3.49357 -1.57255 0.49245	O -0.26838 2.68564 -1.59568	O 3.07033 2.61388 -0.23333
H -3.73501 -2.48844 0.99019	C 1.71866 0.43809 -0.86299	C 0.66678 0.94507 -0.06674
C -4.5579 -0.80164 -0.32957	C 2.53927 -0.82261 -0.53307	C -0.58562 0.22819 0.0082
H -5.55326 -1.18705 -0.40452	C 2.02286 -1.98506 -0.08089	C -2.8435 -0.14412 0.03152
C -4.2327 0.37533 -0.95887	C 4.32024 -2.14694 0.13912	H 7.68247 0.20754 -0.05855
H -4.98335 0.91827 -1.49425	N 4.01338 -0.935 -0.66013	H 7.03933 -2.17155 0.16074
C -2.78311 0.91661 -0.90162	H -5.12788 1.95837 0.61425	H 4.65346 -2.84406 0.24462
H -2.50912 1.82599 -1.39441	H -4.8828 -0.18567 1.74903	H 5.90361 1.9772 -0.20105
C 3.87425 -0.56868 0.25689	H -2.66825 -1.58585 1.46595	H 0.58616 2.02766 -0.16838
H 4.33395 -1.43633 0.68207	H -3.12253 2.94448 -0.78642	C 2.42779 -0.95347 0.09928
C 4.59251 0.62522 -0.35739	H 2.21138 1.32009 -1.2154	H 2.06748 -1.59281 -0.72061
H 5.65124 0.75329 -0.44453	H 0.98534 -2.24116 -0.02724	H 2.09014 -1.42746 1.03326
C 3.64389 1.50091 -0.75241	H 4.27045 -1.07466 -1.61638	C -1.8791 0.84039 -0.05999
C 1.45508 -1.14023 0.88848	C 3.19199 -2.85443 0.36038	H -2.06471 1.89622 -0.20406
H 1.72338 -1.98353 1.48994	H 3.12985 -3.83582 0.78217	C -2.16222 -1.3475 0.14743
C -1.04843 -1.53725 1.39612	C -0.46632 -0.77503 -0.2018	C -2.73619 -2.77166 0.26538
H -1.15658 -1.22001 2.41228	H 0.005 -1.30619 0.59861	C -3.00232 -3.31453 1.52273
H -0.94015 -2.60165 1.38088	H -0.63528 -1.45574 -1.00989	C -2.9901 -3.51882 -0.88477
C 3.91301 2.94536 -1.21363	C 5.72046 -2.53764 0.64741	C -3.52161 -4.60452 1.62985
C 2.84611 3.76791 -1.59971	C 6.82333 -1.72267 0.3586	H -2.8013 -2.72552 2.42932
C 5.22481 3.43726 -1.24728	C 5.89179 -3.70813 1.39874	C -3.51048 -4.80885 -0.77783
C 3.09101 5.08236 -2.01943	C 8.09753 -2.07818 0.82113	H -2.78058 -3.09105 -1.87585
H 1.84452 3.39234 -1.57402	H 6.69251 -0.82897 -0.21507	C -3.77612 -5.35183 0.47921
C 5.46971 4.75172 -1.667	C 7.166 -4.06367 1.86124	H -3.73081 -5.03272 2.6209
H 6.03942 2.80923 -0.9525	H 5.04973 -4.33037 1.61927	H -3.71096 -5.39757 -1.68485
C 4.40281 5.57426 -2.05309	C 8.26887 -3.24869 1.57244	H -4.18562 -6.3689 0.56392
H 2.2764 5.7104 -2.31421	H 8.93959 -1.45592 0.60064	N -0.8142 -1.14191 0.14774
H 6.4713 5.12729 -1.69269	H 7.29683 -4.95738 2.43488	H -0.56398 -1.60298 0.9991
H 4.5898 6.57787 -2.37356	H 9.24176 -3.52015 1.92558	H -3.91211 -0.09316 0.0125

1-(*Z*-i)

C -2.27372 -1.0211 0.35018	H -2.6621 2.11084 -1.12859	C 5.17065 5.2082 -1.72781
C -1.95688 0.34531 -0.21554	C 4.49235 1.167 -0.004	H 5.83851 3.39488 -0.79284
C -0.46298 0.59844 -0.02624	C 3.52874 1.94979 -0.53421	C 4.06802 5.90957 -2.23396
O 0.17417 1.63171 -0.35786	C -1.04922 -1.57613 1.11573	H 1.94947 5.86865 -2.57808
C 2.48934 0.01528 0.19372	H -0.88064 -2.61664 0.93181	H 6.14541 5.64832 -1.76014
C -3.47663 -1.59626 0.16504	H -1.18819 -1.43381 2.16708	N 3.95718 -0.19137 0.26094
H -3.67218 -2.59229 0.50356	H 5.49457 1.48164 0.19965	H 4.25205 -0.82672 -0.45277
C -4.57227 -0.76089 -0.54528	C 3.71997 3.35396 -1.13696	C 2.21389 1.19363 -0.40465
H -5.54316 -1.18209 -0.70294	C 2.61734 4.05534 -1.64312	H 1.24971 1.53864 -0.71485
C -4.30621 0.51818 -0.96979	C 4.99663 3.9304 -1.1793	C 0.11554 -0.67595 0.62086
H -5.07947 1.09942 -1.4271	C 2.79136 5.33314 -2.19162	C 1.43411 -0.96805 0.73329
C -2.89045 1.12028 -0.79461	H 1.64258 3.61522 -1.61079	H 1.74199 -1.88189 1.19696

H	4.20089	6.8852	-2.65276

4-(*Z*-i)

C	-2.2577	-1.04416	0.56662
C	-1.88019	0.19328	-0.21679
C	0.15201	-0.79583	0.74719
C	-0.37106	0.38855	-0.09014
O	0.31338	1.31725	-0.59277
C	2.54969	-0.31787	0.18339
N	2.30387	0.86956	-0.67125
H	1.60654	1.48128	-0.29772
C	-3.49357	-1.57255	0.49245
H	-3.73501	-2.48844	0.99019
C	-4.5579	-0.80164	-0.32957
H	-5.55326	-1.18705	-0.40452
C	-4.2327	0.37533	-0.95887
H	-4.98335	0.91827	-1.49425
C	-2.78311	0.91661	-0.90162
H	-2.50912	1.82599	-1.39441
C	3.87425	-0.56868	0.25689
H	4.33395	-1.43633	0.68207
C	4.59251	0.62522	-0.35739
H	5.65124	0.75329	-0.44453
C	3.64389	1.50091	-0.75241
C	1.45508	-1.14023	0.88848
H	1.72338	-1.98353	1.48994
C	-1.04843	-1.53725	1.39612
H	-1.15658	-1.22001	2.41228
H	-0.94015	-2.60165	1.38088
C	3.91301	2.94536	-1.21363
C	2.84611	3.76791	-1.59971
C	5.22481	3.43726	-1.24728
C	3.09101	5.08236	-2.01943
H	1.84452	3.39234	-1.57402
C	5.46971	4.75172	-1.667
H	6.03942	2.80923	-0.9525
C	4.40281	5.57426	-2.05309
H	2.2764	5.7104	-2.31421
H	6.4713	5.12729	-1.69269
O	4.65271	6.91554	-2.48139
C	4.86457	6.93271	-3.8955
H	3.99418	6.55635	-4.39115
H	5.05156	7.93632	-4.21597
H	5.70651	6.31831	-4.13749

4-(*E*-i)

C	-4.21334	1.41009	0.52529
C	-4.08166	0.19362	1.1497
C	-2.78194	-0.63543	0.98774
C	-1.80536	-0.12251	0.21618
C	-1.91558	1.26369	-0.37861
C	-3.04181	1.9939	-0.3019
C	0.37249	0.43041	-0.70698
C	-0.56492	1.62021	-0.99527
O	-0.26838	2.68564	-1.59568
C	1.71866	0.43809	-0.86299
C	2.53927	-0.82261	-0.53307
C	2.02286	-1.98506	-0.08089
C	4.32024	-2.14694	0.13912
N	4.01338	-0.935	-0.66013
H	-5.12788	1.95837	0.61425
H	-4.8828	-0.18567	1.74903
H	-2.66825	-1.58585	1.46595
H	-3.12253	2.94448	-0.78642
H	2.21138	1.32009	-1.2154
H	0.98534	-2.24116	-0.02724
H	4.27045	-1.07466	-1.61638
C	3.19199	-2.85443	0.36038
H	3.12985	-3.83582	0.78217
C	-0.46632	-0.77503	-0.2018
H	0.005	-1.30619	0.59861
H	-0.63528	-1.45574	-1.00989
C	5.72046	-2.53764	0.64741
C	6.82333	-1.72267	0.3586
C	5.89179	-3.70813	1.39874
C	8.09753	-2.07818	0.82113
H	6.69251	-0.82897	-0.21507
C	7.166	-4.06367	1.86124
H	5.04973	-4.33037	1.61927
C	8.26887	-3.24869	1.57244
H	8.93959	-1.45592	0.60064
H	7.29683	-4.95738	2.43488
O	9.56908	-3.61148	2.04439
C	10.21986	-4.42849	1.06773
H	10.31215	-3.88383	0.15137
H	11.19276	-4.69993	1.42084
H	9.64161	-5.31306	0.90018

4-(*E*-ii)

C	-4.30867	1.45328	0.39202
C	-4.17237	0.32395	1.16213
C	-2.85718	-0.49575	1.1278
C	-1.87188	-0.06311	0.31903
C	-1.98992	1.23863	-0.44191
C	-3.12835	1.95293	-0.47675
C	0.31696	0.41186	-0.62136
C	-0.63169	1.54135	-1.07057
O	-0.33819	2.53116	-1.79009
C	1.66594	0.42412	-0.75055
C	2.49797	-0.7727	-0.25362
C	4.33023	-2.09528	0.24247
H	-5.23303	1.99221	0.39564
H	-4.98056	0.00614	1.78721
H	-2.73967	-1.37906	1.72013
H	-3.21277	2.83621	-1.07473
H	2.15292	1.26537	-1.19781
C	-0.51446	-0.73796	0.00996
H	-0.05273	-1.15983	0.87814
H	-0.65562	-1.51444	-0.71256
C	3.10262	-2.89945	0.46174
C	4.01303	-0.90749	-0.31516
H	4.80467	-0.29408	-0.69191
C	2.8905	-4.39918	0.73998
C	2.79941	-4.85873	2.05413
C	2.78934	-5.29808	-0.32176
C	2.60787	-6.217	2.3064
H	2.87998	-4.1499	2.89097
C	2.59675	-6.65666	-0.06959
H	2.86093	-4.93603	-1.35757
C	2.50615	-7.11625	1.24422
H	2.53669	-6.57939	3.34221
H	2.51661	-7.36512	-0.90693
H	5.32184	-2.40921	0.49357
O	2.30969	-8.50881	1.5032
C	3.57928	-9.15429	1.63123
H	3.43178	-10.1971	1.82012
H	4.13503	-9.03104	0.72522
H	4.121	-8.7177	2.44415
N	2.02225	-1.89013	0.33585
H	1.69542	-1.83141	1.27911

8-(Z-i)

C	4.06941	0.36955	0.00985
C	3.1779	-0.70977	0.02769
C	1.84595	1.24916	-0.01029
C	1.78148	-0.22062	0.01738
O	0.78637	-0.96689	0.0317
C	-0.59082	1.99749	-0.03921
N	-1.25272	0.78809	-0.01488
H	-0.7191	-0.08995	0.03988
C	5.44289	0.13543	0.016

8-(E-i)

C	6.59768	0.20525	0.05273
C	6.34609	-1.16985	-0.06724
C	5.03906	-1.66379	-0.12329
C	3.97903	-0.76234	-0.05752
C	4.23703	0.60899	0.06216
C	5.5389	1.10859	0.11854
C	1.87462	0.34477	0.01772
C	2.95724	1.36238	0.1138
O	2.81991	2.57652	0.21604

8-(E-ii)

C	-5.77924	-1.48635	-0.07661
C	-4.87833	-2.56185	-0.07963
C	-3.49678	-2.34676	-0.06065
C	-3.02449	-1.03666	-0.03822
C	-3.92753	0.03238	-0.0354
C	-5.30735	-0.17544	-0.05429
C	-1.74216	0.9737	0.00529
C	-3.18893	1.32266	-0.00945
O	-3.67681	2.44672	-0.00007
C	-0.80043	1.94774	0.04468

H	6.15201	0.95972	0.00262
C	5.89801	-1.18607	0.03983
H	6.96697	-1.38312	0.04471
C	4.99862	-2.26386	0.05777
H	5.38039	-3.28099	0.0763
C	3.62537	-2.03272	0.05185
H	2.9066	-2.84686	0.06559
C	-1.57926	3.00003	-0.06843
H	-1.38672	4.0645	-0.10163
C	-2.82514	2.36838	-0.0569
H	-3.79303	2.84833	-0.09528
C	-2.60045	0.97817	-0.02242
C	0.82184	2.15982	-0.03355
H	1.1212	3.20829	-0.05277
C	3.31017	1.67643	-0.01379
H	3.55937	2.30018	0.85672
H	3.56352	2.27135	-0.90302
C	-3.55275	-0.13124	-0.01101
C	-3.13975	-1.45467	-0.25767
C	-4.91607	0.10363	0.2477
C	-4.05909	-2.50078	-0.24294
H	-2.09765	-1.66987	-0.47616
C	-5.83211	-0.94416	0.25693
H	-5.25534	1.11363	0.45761
C	-5.40923	-2.25303	0.01275
H	-3.71795	-3.51406	-0.43748
H	-6.87954	-0.7388	0.46167
C	-6.42293	-3.41226	0.02677
N	-7.1865	-4.28544	0.03733

C	0.57433	0.71927	0.04085
C	-0.5986	-0.09876	-0.03614
C	-0.80932	-1.4783	-0.15787
C	-2.84255	-0.47557	-0.05022
N	-1.85832	0.4774	0.01344
H	7.62325	0.56204	0.09394
H	7.18189	-1.86313	-0.11732
H	4.86086	-2.7326	-0.21633
H	5.70275	2.17851	0.21156
H	0.41312	1.79526	0.13058
H	-0.03513	-2.22592	-0.24659
H	-2.02105	1.45741	0.19365
C	-2.19588	-1.7091	-0.16314
H	-2.68943	-2.6643	-0.27788
C	2.48486	-1.03458	-0.09695
H	2.19123	-1.54025	-1.0286
H	2.17173	-1.69628	0.72407
C	-4.26648	-0.14618	-0.01849
C	-4.73741	1.12331	-0.4021
C	-5.20674	-1.10602	0.39982
C	-6.09716	1.42399	-0.35881
H	-4.04066	1.87258	-0.77002
C	-6.56559	-0.80614	0.43163
H	-4.8615	-2.08451	0.7206
C	-7.01872	0.46142	0.057
H	-6.43794	2.40998	-0.66315
H	-7.27359	-1.56201	0.76088
C	-8.52196	0.79313	0.09985
N	-9.64119	1.04011	0.13175

C	0.62602	1.85103	0.07095
C	2.84874	2.31883	0.12253
H	-6.84811	-1.68131	-0.09169
H	-5.26061	-3.57924	-0.09708
H	-2.81118	-3.1911	-0.06363
H	-5.98116	0.6767	-0.05139
H	-1.19006	2.96505	0.06027
C	-1.58983	-0.53553	-0.01658
H	-1.05153	-0.91543	0.86541
H	-1.04142	-0.89104	-0.90357
C	1.5638	2.88701	0.13473
H	1.31604	3.93821	0.19756
C	2.70317	0.93114	0.05488
C	3.70714	-0.1307	0.02315
C	5.00946	0.13834	-0.43742
C	3.40878	-1.43889	0.44778
C	5.97512	-0.86352	-0.47007
H	5.25346	1.13613	-0.78998
C	4.37456	-2.44221	0.40401
H	2.4247	-1.66768	0.84972
C	5.66272	-2.16037	-0.05405
H	6.97363	-0.63377	-0.83215
H	4.12269	-3.44419	0.74105
N	1.35421	0.67195	0.03868
H	0.95572	-0.24021	-0.11951
H	3.79012	2.84536	0.19608
C	6.73092	-3.26879	-0.09791
N	7.52625	-4.09407	-0.13057

9-(Z-i)

C	2.14415	0.40311	0.00002
C	1.68665	-0.918	0.00004
C	-0.24058	0.45791	0.00006
C	0.20874	-0.94352	0.00005
O	-0.46462	-1.98741	0.0001
C	-2.79167	0.29188	-0.00007
N	-2.99941	-1.07484	-0.00009
H	-2.20224	-1.72399	-0.00003
C	3.51488	0.65786	-0.00004
H	3.89829	1.67521	-0.00008
C	4.40125	-0.42332	-0.00004
H	5.47212	-0.23646	-0.00002
C	3.93283	-1.74624	-0.00006
H	4.64375	-2.56796	-0.00006
C	2.56426	-2.00446	0.00001
H	2.17122	-3.017	0.00007
C	-4.0614	0.89548	0.00001
H	-4.24213	1.96261	-0.00004
C	-5.02275	-0.12781	0.00003
H	-6.09837	-0.01632	0.00006
C	-4.3273	-1.3371	-0.00004
C	0.9843	1.39379	0.
C	-1.52154	0.93856	0.00003
H	-1.61985	2.02445	0.
C	1.0273	2.2822	1.26439
H	1.94028	2.88903	1.28379
H	0.17246	2.96785	1.28839

9-(E-i)

C	4.55745	0.43606	-0.00094
C	4.32617	-0.94826	-0.00116
C	3.02761	-1.46523	-0.0007
C	1.94969	-0.57948	-0.00014
C	2.19051	0.79789	0.00004
C	3.48422	1.32325	-0.00035
C	-0.17249	0.4933	0.0008
C	0.90387	1.52961	0.00067
O	0.75337	2.74703	0.00095
C	-1.45375	0.93541	0.00084
C	-2.74218	0.29709	0.00058
C	-3.22832	-1.0158	0.00112
C	-5.00852	0.36673	-0.00175
N	-3.86901	1.11251	-0.00107
H	5.57739	0.81085	-0.00134
H	5.17253	-1.63059	-0.0017
H	2.87289	-2.54141	-0.00085
H	3.62867	2.39995	-0.00024
H	-1.50528	2.02793	0.00071
H	-2.62864	-1.91212	0.00262
H	-3.83047	2.12141	-0.00183
C	-4.63911	-0.96845	-0.00041
H	-5.31487	-1.8126	-0.00057
C	0.45195	-0.90461	0.00045
C	0.09395	-1.7019	-1.27554
H	-0.98266	-1.8757	-1.35346
H	0.41105	-1.15855	-2.17147

9-(E-ii)

C	-6.14689	-2.15953	-0.09012
C	-5.08209	-3.07276	-0.1094
C	-3.7543	-2.63443	-0.09041
C	-3.50263	-1.26496	-0.05152
C	-4.56878	-0.35871	-0.03259
C	-5.89577	-0.78935	-0.05139
C	-2.56909	0.92869	0.01709
C	-4.05195	1.03537	0.00954
O	-4.71991	2.06299	0.03491
C	-1.80007	2.04473	0.06812
C	-0.37887	2.18751	0.08979
C	1.73437	3.02177	0.14391
H	-7.16948	-2.52677	-0.10548
H	-5.29269	-4.13875	-0.13966
H	-2.93967	-3.35502	-0.10606
H	-6.6996	-0.05876	-0.03565
H	-2.35374	2.98265	0.09815
C	-2.17015	-0.5343	-0.02582
C	0.37207	3.36504	0.16647
H	-0.04903	4.35846	0.24577
C	1.82455	1.63025	0.0558
N	0.53876	1.14756	0.03802
H	0.2999	0.18461	-0.13957
C	2.99264	0.75459	0.00687
C	2.93785	-0.58082	0.43392
C	4.22809	1.23506	-0.4747
C	4.05479	-1.41557	0.38003

H	1.00117	1.67368	2.17403
C	1.02723	2.28219	-1.26439
H	0.17236	2.9678	-1.28834
H	1.94018	2.88905	-1.28382
H	1.00109	1.67366	-2.17402
C	-4.85182	-2.78502	-0.00002
C	-5.08965	-3.44076	-1.2083
C	-5.08903	-3.44094	1.20789
C	-5.56522	-4.75189	-1.20856
H	-4.90327	-2.9231	-2.16042
C	-5.56381	-4.75281	1.20779
H	-4.90147	-2.92433	2.16028
C	-5.80205	-5.40829	-0.00015
H	-5.75329	-5.26859	-2.16089
H	-5.75036	-5.26986	2.16033
O	-6.28958	-6.75261	-0.00074
C	-7.71954	-6.7418	0.00144
H	-8.0737	-6.23315	-0.87076
H	-8.08383	-7.74787	0.0002
H	-8.07105	-6.23627	0.87653

H	0.59599	-2.67661	-1.27381
C	0.09491	-1.70178	1.27676
H	-0.98195	-1.873	1.35718
H	0.59473	-2.67762	1.27356
H	0.41519	-1.15961	2.1723
C	-6.39924	1.02815	-0.00341
C	-7.03025	1.32845	1.20411
C	-7.02799	1.3271	-1.21207
C	-8.29002	1.92695	1.20288
H	-6.53463	1.09194	2.15682
C	-8.28772	1.92668	-1.21347
H	-6.53073	1.09064	-2.16388
C	-8.91885	2.22651	-0.00628
H	-8.78773	2.16311	2.15463
H	-8.78304	2.16265	-2.1666
O	-10.21045	2.84022	-0.00721
C	-11.22011	1.82756	-0.01033
H	-11.1152	1.21662	0.86181
H	-12.18633	2.28726	-0.00967
H	-11.11428	1.22108	-0.88548

H	2.01646	-0.97866	0.8524
C	5.34682	0.42021	-0.52433
H	4.29897	2.25831	-0.83152
C	5.27097	-0.91653	-0.10046
H	3.96851	-2.43883	0.72704
H	6.29533	0.79105	-0.89992
H	2.57428	3.69779	0.22451
O	6.42522	-1.63492	-0.19498
C	6.4092	-2.99272	0.21807
H	7.42122	-3.36685	0.05432
H	6.15529	-3.08738	1.2821
H	5.70224	-3.58666	-0.37617
C	-1.33707	-0.88111	-1.27373
H	-1.06139	0.02015	-1.78032
H	-0.45422	-1.40837	-0.97798
H	-1.91678	-1.49609	-1.92997
C	-1.33396	-0.95008	1.19873
H	-0.46822	-1.48818	0.87339
H	-1.0297	-0.07631	1.73619
H	-1.92295	-1.57462	1.83742
-0.37992			

10-(Z-i)

C	-2.76323	1.77451	0.20413
C	-1.5141	2.39802	0.23962
C	-1.07666	0.08642	0.00686
C	-0.43585	1.39484	0.12941
O	0.77571	1.68647	0.15012
C	0.86907	-1.55948	-0.13251
N	1.95746	-0.70939	-0.06948
H	1.80697	0.30289	0.0412
C	-3.92139	2.54519	0.2959
H	-4.9047	2.08582	0.26318
C	-3.79921	3.9312	0.42616
H	-4.69635	4.54055	0.4993
C	-2.53996	4.55003	0.46082
H	-2.47362	5.63005	0.5603
C	-1.38212	3.78269	0.36661
H	-0.39225	4.22896	0.39021
C	1.3791	-2.87914	-0.24124
C	2.77245	-2.77294	-0.23573
H	3.46968	-3.59588	-0.3189
C	3.11792	-1.40944	-0.12594
C	-2.62092	0.25233	0.03845
C	-0.48515	-1.15198	-0.09255
H	-1.17424	-1.99241	-0.15157
C	4.42996	-0.77158	-0.0821
C	4.57664	0.62425	-0.14145
C	5.60422	-1.54617	0.02226
C	5.83152	1.22992	-0.09716
H	3.7028	1.26301	-0.23276
C	6.8564	-0.95619	0.06429
H	5.53108	-2.62786	0.08096
C	6.98335	0.4407	0.00577
H	5.89665	2.31075	-0.14688
H	7.75745	-1.55578	0.14747
O	8.25722	0.92396	0.05611
C	8.44531	2.32983	0.00788

10-(E-i)

C	5.58548	-3.17001	-0.23965
C	5.78047	-2.15623	0.71237
C	4.83615	-1.14427	0.89666
C	3.67982	-1.15423	0.11608
C	3.4908	-2.16791	-0.82337
C	4.43173	-3.18254	-1.0172
C	1.54953	-0.77591	-0.90201
C	2.18801	-1.993	-1.49636
O	1.70727	-2.72748	-2.35264
C	0.2403	-0.59091	-1.21725
C	-0.82494	0.31757	-0.86734
C	-1.00104	1.6951	-0.65228
C	-3.04656	0.68921	-0.4967
N	-2.10122	-0.2479	-0.81809
H	6.33826	-3.94364	-0.36404
H	6.68457	-2.15725	1.31589
H	5.00744	-0.36578	1.63398
H	4.2449	-3.95413	-1.75862
H	-0.11044	-1.40141	-1.86468
H	-2.25266	-1.24442	-0.75173
C	-2.37978	1.90711	-0.41535
H	-2.85135	2.87065	-0.27106
C	2.51984	-0.12858	0.11304
C	-4.45856	0.35761	-0.32192
C	-5.03534	-0.76683	-0.93153
C	-5.29454	1.17234	0.46878
C	-6.38358	-1.08344	-0.76077
H	-4.4339	-1.39734	-1.58168
C	-6.63719	0.87579	0.63743
H	-4.8728	2.03881	0.96976
C	-7.19521	-0.25903	0.02698
H	-6.78823	-1.95905	-1.25532
H	-7.27826	1.50191	1.25013
O	-8.52267	-0.4662	0.26089
C	-9.13949	-1.60127	-0.32629

10-(E-ii)

C	-6.14689	-2.15953	-0.09012
C	-5.08209	-3.07276	-0.1094
C	-3.7543	-2.63443	-0.09041
C	-3.50263	-1.26496	-0.05152
C	-4.56878	-0.35871	-0.03259
C	-5.89577	-0.78935	-0.05139
C	-2.56909	0.92869	0.01709
C	-4.05195	1.03537	0.00954
O	-4.71991	2.06299	0.03491
C	-1.80007	2.04473	0.06812
C	-0.37887	2.18751	0.08979
C	1.73437	3.02177	0.14391
H	-7.16948	-2.52677	-0.10548
H	-5.29269	-4.13875	-0.13966
H	-2.93967	-3.35502	-0.10606
H	-6.6996	-0.05876	-0.03565
H	-2.35374	2.98265	0.09815
C	-2.17015	-0.5343	-0.02582
C	0.37207	3.36504	0.16647
H	-0.04903	4.35846	0.24577
C	1.82455	1.63025	0.0558
N	0.53876	1.14756	0.03802
H	0.2999	0.18461	-0.13957
C	2.99264	0.75459	0.00687
C	2.93785	-0.58082	0.43392
C	4.22809	1.23506	-0.4747
C	4.05479	-1.41557	0.38003
H	2.01646	-0.97866	0.8524
C	5.34682	0.42021	-0.52433
H	4.29897	2.25831	-0.83152
C	5.27097	-0.91653	-0.10046
H	3.96851	-2.43883	0.72704
H	6.29533	0.79105	-0.89992
H	2.57428	3.69779	0.22451
O	6.42522	-1.63492	-0.19498

H	7.95863 2.83108 0.85483	H	-8.67892 -2.53559 0.02106	C	6.4092 -2.99272 0.21807
H	9.52354 2.48926 0.0654	H	-10.18288 -1.57321 -0.0073	H	7.42122 -3.36685 0.05432
H	8.06521 2.75578 -0.93001	H	-9.09461 -1.56309 -1.42275	H	6.15529 -3.08738 1.2821
C	-4.70245 -0.33169 -1.33977	C	1.22804 1.11062 1.97831	H	5.70224 -3.58666 -0.37617
C	-2.60038 -0.1953 -2.49698	C	1.7369 -1.2175 2.28	C	-1.33396 -0.95008 1.19873
C	-5.35384 -0.59019 -2.54559	C	0.55893 1.12207 3.20456	C	-0.56276 -2.11188 1.15484
H	-5.28167 -0.29912 -0.42209	H	1.28382 2.02537 1.40185	C	-1.34747 -0.16515 2.35166
C	-3.25041 -0.45355 -3.70557	C	1.06674 -1.21 3.50206	C	0.19419 -2.48896 2.26392
H	-1.5269 -0.04065 -2.49444	H	2.18674 -2.14137 1.93021	H	-0.55292 -2.7309 0.24601
C	-4.6299 -0.6537 -3.73713	C	0.47581 -0.03659 3.97409	C	-0.58958 -0.54168 3.46082
H	-6.42993 -0.74434 -2.55078	H	0.09944 2.04369 3.55241	H	-1.9551 0.75066 2.38636
H	-2.67103 -0.49799 -4.62427	H	1.00971 -2.12453 4.08692	C	0.18109 -1.70345 3.41718
H	-5.13522 -0.85802 -4.67728	H	-0.04352 -0.02759 4.92871	H	0.8016 -3.40503 2.22962
C	-3.21266 0.05353 2.52588	C	3.24616 1.47314 -1.74634	H	-0.60011 0.07762 4.36957
C	-3.57754 -1.86841 1.12981	C	3.80084 2.06584 0.51673	H	0.77805 -2.00093 4.2915
C	-3.6272 -0.67642 3.64013	C	3.92497 2.60054 -2.20998	C	-1.33707 -0.88111 -1.27373
H	-2.90688 1.08684 2.65494	H	2.76925 0.80834 -2.4599	C	-1.1724 0.06356 -2.28712
C	-3.99 -2.60155 2.24386	C	4.47792 3.1956 0.05505	C	-0.74706 -2.13957 -1.39087
H	-3.57965 -2.34449 0.15414	H	3.76411 1.87103 1.58364	C	-0.41844 -0.25048 -3.41767
C	-4.01854 -2.00898 3.50607	C	4.54208 3.47084 -1.31106	H	-1.63832 1.05534 -2.19483
H	-3.64309 -0.1984 4.61629	H	3.96916 2.79618 -3.27822	C	0.00798 -2.45352 -2.52125
H	-4.29396 -3.63788 2.11939	H	4.95681 3.86067 0.76923	H	-0.8766 -2.88417 -0.59218
H	-4.34364 -2.57711 4.37349	H	5.06808 4.35115 -1.67045	C	0.17226 -1.5093 -3.53466
C	-3.1818 -0.52686 1.24927	C	3.16122 1.19057 -0.37494	H	-0.28912 0.49384 -4.21677
C	-3.31231 -0.1338 -1.2916	C	1.83728 -0.05565 1.49711	H	0.4734 -3.4457 -2.61315
H	0.8069 -3.78005 -0.31766	H	-0.29432 2.48974 -0.77061	H	0.76674 -1.75666 -4.42612

TS-1 (4)

Charge = 0 Multiplicity = 1

Symbolic Z-Matrix:

C	-6.78656 1.94516 -0.12416
C	-7.37318 0.67271 -0.04443
C	-6.58882 -0.48212 0.02729
C	-5.20087 -0.35496 0.01879
C	-4.62254 0.91993 -0.0609
C	-5.40023 2.07758 -0.13302
C	-2.83039 -0.64213 0.03828
C	-3.14745 0.80927 -0.05346
O	-2.33071 1.72562 -0.11121
C	-1.5366 -1.07402 0.06531
C	-0.18881 -1.5505 0.09498
C	0.38017 -2.83265 0.18024
C	2.07575 -1.33678 0.05982
N	0.88569 -0.66841 0.03184
H	-7.41975 2.82656 -0.1789
H	-8.45668 0.58411 -0.03846
H	-7.05944 -1.46061 0.08867
H	-4.91807 3.04915 -0.19393
H	-1.18166 -0.00816 0.00488
H	-0.18755 -3.74967 0.26422
H	0.77784 0.32557 -0.12031
C	1.77422 -2.70321 0.15723
H	2.50443 -3.49666 0.24002
C	-4.13536 -1.43045 0.08602
H	-4.22377 -2.02828 1.00429
H	-4.22217 -2.13688 -0.75178
C	3.36693 -0.65439 0.01027

TS-2 (4)

Charge = 0 Multiplicity = 1

Symbolic Z-Matrix:

C	3.75568 3.16297 -0.86743
C	3.73887 3.33053 0.5253
C	3.53169 2.24138 1.37855
C	3.33339 0.98157 0.81624
C	3.37263 0.82072 -0.56742
C	3.56996 1.89998 -1.42946
C	3.12266 -1.3872 0.30993
C	3.1792 -0.61075 -0.95167
O	3.00558 -0.99297 -2.10791
C	2.26231 -2.50777 0.45899
C	0.86255 -2.57696 0.36684
C	-1.28868 -3.26372 0.09252
H	3.91809 4.02506 -1.50935
H	3.88462 4.32206 0.94683
H	3.51624 2.38385 2.45702
H	3.58318 1.73889 -2.50348
H	2.65999 -3.43856 0.02634
C	3.0584 -0.35368 1.45755
H	3.80953 -0.60021 2.22417
H	2.096 -0.36042 1.9967
C	0.02636 -3.70701 0.21029
H	0.37643 -4.73034 0.1942
C	-1.28559 -1.85807 0.18878
N	0.01178 -1.47488 0.39793
H	0.34658 -0.52762 0.29224
C	-2.38341 -0.91068 0.07904
C	-2.27986 0.40231 0.56889

C	3.51409	0.6855	0.39981
C	4.51836	-1.33604	-0.43436
C	4.74796	1.33373	0.34337
H	2.6605	1.23694	0.78607
C	5.75115	-0.70695	-0.48651
H	4.4344	-2.36827	-0.76114
C	5.87806	0.63763	-0.10158
H	4.81643	2.36839	0.65881
H	6.63531	-1.23167	-0.83454
O	7.13045	1.16588	-0.19615
C	7.31752	2.52452	0.17106
H	7.07004	2.69433	1.22711
H	8.37648	2.73406	0.01074
H	6.71553	3.19704	-0.45395

C	-3.602	-1.29042	-0.5247
C	-3.3322	1.3085	0.46051
H	-1.37323	0.72492	1.07389
C	-4.65663	-0.40168	-0.63245
H	-3.70885	-2.2908	-0.93266
C	-4.53225	0.9102	-0.14357
H	-3.21114	2.30997	0.85648
H	-5.59032	-0.69196	-1.10336
H	-2.16959	-3.87696	-0.03532
O	-5.62293	1.70524	-0.30155
C	-5.55447	3.04961	0.15487
H	-6.5223	3.49373	-0.08265
H	-5.38882	3.0976	1.23882
H	-4.7617	3.60779	-0.35927

TS-1 (9)

Charge = 0 Multiplicity = 1

Symbolic Z-Matrix:

C	7.19556	-0.27482	0.0284
C	6.80146	-1.61882	0.10851
C	5.44974	-1.97572	0.12587
C	4.48461	-0.97108	0.06233
C	4.88845	0.36796	-0.01851
C	6.2358	0.73276	-0.03633
C	2.51443	0.36115	-0.02414
C	3.70304	1.25249	-0.07707
O	3.69534	2.4768	-0.15378
C	1.25368	0.87859	-0.05883
C	-0.10275	1.32002	-0.08891
C	-1.99543	2.57907	-0.15928
H	8.25278	-0.02391	0.01676
H	7.56029	-2.39578	0.15794
H	5.16573	-3.0235	0.18835
H	6.50979	1.78207	-0.09951
H	1.62659	1.93271	-0.12318
C	2.96265	-1.10473	0.06739
C	-0.59504	2.6294	-0.17815
H	0.02863	3.50938	-0.26176
C	-2.38124	1.23592	-0.06094
N	-1.23196	0.49698	-0.03212
H	-1.19375	-0.4969	0.13757
C	-3.71378	0.63796	-0.00987
C	-3.95105	-0.68136	-0.42364
C	-4.81456	1.38294	0.46094
C	-5.22365	-1.25048	-0.36643
H	-3.13774	-1.27622	-0.8321
C	-6.08469	0.83327	0.51455
H	-4.66041	2.40078	0.80674
C	-6.30195	-0.49231	0.10481
H	-5.36287	-2.27147	-0.70262
H	-6.92978	1.40651	0.8825
H	-2.67253	3.41709	-0.2485
O	-7.58493	-0.94038	0.2034
C	-7.86482	-2.27516	-0.19048
H	-8.93346	-2.41846	-0.02186
H	-7.64046	-2.43744	-1.2529
H	-7.30184	-2.99961	0.41265
C	2.46751	-1.76452	1.37329

TS-2(9)

Charge = 0 Multiplicity = 1

Symbolic Z-Matrix:

C	5.8351	2.36671	0.33114
C	5.74435	2.10555	-1.04343
C	4.8001	1.20195	-1.54327
C	3.94715	0.56097	-0.64498
C	4.02905	0.84564	0.71252
C	4.97415	1.73164	1.22683
C	2.08435	-0.52812	0.47139
C	2.99035	0.08427	1.46189
O	2.94686	0.01855	2.69245
C	1.43382	-1.79928	0.64127
C	0.07662	-2.04272	0.45961
C	-2.00647	-2.90521	0.18377
H	6.57937	3.07014	0.69606
H	6.42227	2.60662	-1.73023
H	4.75231	1.00042	-2.61115
H	5.01674	1.91513	2.29663
H	2.01765	-2.70132	0.40964
C	2.89331	-0.50471	-0.89848
C	-0.67036	-3.24471	0.33214
H	-0.24664	-4.23952	0.36534
C	-2.11086	-1.48972	0.22416
N	-0.85481	-1.00966	0.40491
H	-0.54923	-0.04414	0.39877
C	-3.28531	-0.64353	0.08512
C	-3.27705	0.70474	0.48169
C	-4.48383	-1.16261	-0.45108
C	-4.40356	1.51193	0.34829
H	-2.38698	1.13612	0.93163
C	-5.6101	-0.37136	-0.58703
H	-4.52158	-2.19569	-0.78238
C	-5.58211	0.97698	-0.1905
H	-4.35615	2.5444	0.67376
H	-6.52905	-0.76854	-1.00589
H	-2.8385	-3.58525	0.06609
O	-6.73711	1.66757	-0.36757
C	-6.77619	3.03818	0.01094
H	-7.78462	3.38183	-0.22374
H	-6.04792	3.63132	-0.55642
H	-6.59003	3.16249	1.08515
C	3.61242	-1.82045	-1.2874

H 1.37106 -1.79184 1.3955
H 2.83054 -2.79592 1.46042
H 2.80819 -1.20624 2.2512
C 2.47128 -1.91995 -1.14904
H 2.8319 -2.95501 -1.10586
H 1.37495 -1.94675 -1.17266
H 2.81732 -1.47522 -2.08761

H 2.90301 -2.61778 -1.53847
H 4.24077 -1.65676 -2.16987
H 4.26002 -2.17139 -0.47627
C 1.93322 -0.09897 -2.03732
H 2.47395 0.00814 -2.98688
H 1.1547 -0.85697 -2.1839
H 1.44663 0.85657 -1.81489

TS-1 (10)

Charge = 0 Multiplicity = 1

Symbolic Z-Matrix:

C 6.53299 0.75063 -1.10323
C 6.2181 -0.61175 -0.98886
C 4.92692 -1.03151 -0.65788
C 3.9371 -0.07144 -0.44682
C 4.25986 1.28542 -0.56971
C 5.54903 1.71297 -0.89305
C 1.94094 1.18548 -0.08645
C 3.06209 2.1228 -0.34203
O 2.99427 3.34636 -0.39086
C 0.65222 1.62331 -0.03838
C -0.72328 1.98327 0.01733
C -2.69542 3.1054 0.12573
H 7.54585 1.05007 -1.35834
H 6.99269 -1.35539 -1.15832
H 4.70759 -2.0911 -0.56746
H 5.75671 2.77573 -0.97847
H 0.9602 2.69801 -0.12163
C 2.46776 -0.26904 -0.02741
C -1.30525 3.25924 0.06314
H -0.74701 4.1858 0.04643
C -2.98348 1.73189 0.10174
N -1.78735 1.08026 0.04595
H -1.66191 0.07829 0.02601
C -4.27045 1.03897 0.10228
C -4.42071 -0.24453 -0.44403
C -5.41289 1.65268 0.65529
C -5.65 -0.90422 -0.43672
H -3.57385 -0.73551 -0.91746
C -6.64072 1.01179 0.66217
H -5.32505 2.63833 1.10265
C -6.77125 -0.27654 0.11892
H -5.72316 -1.89229 -0.87634
H -7.51817 1.48294 1.09377
H -3.4324 3.89615 0.14249
O -8.01862 -0.82175 0.17976
C -8.2101 -2.12628 -0.34583
H -9.2636 -2.36146 -0.18509
H -7.99016 -2.1643 -1.42088
H -7.58923 -2.86605 0.17642
C 1.64116 -1.12686 -1.00021
C 0.46558 -1.76396 -0.56846
C 1.96796 -1.20537 -2.3608
C -0.34981 -2.45858 -1.46482
H 0.20267 -1.74097 0.48532

TS-2 (10)

Charge = 0 Multiplicity = 1

Symbolic Z-Matrix:

C 4.42743 3.74907 0.50803
C 4.44193 3.23816 -0.79784
C 3.72131 2.08567 -1.12744
C 2.97777 1.45735 -0.12902
C 2.95466 1.979 1.15775
C 3.68346 3.11529 1.50375
C 1.36195 0.20373 1.18463
C 2.08045 1.15303 2.04019
O 2.00844 1.2999 3.26382
C 0.91718 -1.10862 1.57521
C -0.37734 -1.57289 1.40673
C -2.30867 -2.75953 1.26718
H 5.00121 4.64244 0.74152
H 5.03073 3.73761 -1.56327
H 3.75666 1.68543 -2.13737
H 3.65395 3.48315 2.5254
H 1.64647 -1.93095 1.55922
C 2.18775 0.14572 -0.18443
C -0.95472 -2.86907 1.52779
H -0.40872 -3.76225 1.80058
C -2.59583 -1.39441 0.98208
N -1.4305 -0.71623 1.09294
H -1.24331 0.24606 0.83604
C -3.86227 -0.77399 0.63025
C -4.05439 0.61514 0.72382
C -4.95078 -1.56095 0.19555
C -5.2709 1.20635 0.39566
H -3.25328 1.2531 1.08638
C -6.16477 -0.98525 -0.13266
H -4.83225 -2.63551 0.09704
C -6.33818 0.40656 -0.03717
H -5.37984 2.28029 0.48976
H -6.99997 -1.58871 -0.47308
H -3.03997 -3.55545 1.28595
O -7.56552 0.87237 -0.38105
C -7.80969 2.27171 -0.30279
H -8.84349 2.4104 -0.62232
H -7.14349 2.83245 -0.97043
H -7.69294 2.64149 0.72364
C 1.12203 0.11193 -1.29103
C 0.58302 -1.09919 -1.75894
C 0.56249 1.30386 -1.78223
C -0.45152 -1.11896 -2.69521
H 0.98089 -2.03928 -1.39129

C	1.15413	-1.89855	-3.2584	C	-0.47239	1.28709	-2.71827
H	2.86628	-0.71694	-2.72531	H	0.94394	2.25582	-1.42786
C	-0.00954	-2.52881	-2.81678	C	-0.98408	0.0748	-3.1838
H	-1.24355	-2.96026	-1.10012	H	-0.84104	-2.07292	-3.04205
H	1.43414	-1.94488	-4.30768	H	-0.87679	2.22726	-3.08539
H	-0.63941	-3.07293	-3.51534	H	-1.7879	0.06036	-3.91514
C	2.47269	-0.85441	1.40648	C	3.26032	-0.96461	-0.30532
C	2.32516	-0.04078	2.53646	C	3.97408	-1.38271	0.82927
C	2.70691	-2.22507	1.60849	C	3.64524	-1.49067	-1.54814
C	2.40714	-0.57602	3.82439	C	5.01171	-2.30945	0.72997
H	2.14194	1.02107	2.41283	H	3.72977	-0.96147	1.80023
C	2.7888	-2.76178	2.89242	C	4.68628	-2.41578	-1.6506
H	2.81122	-2.88477	0.75179	H	3.13134	-1.17441	-2.44998
C	2.63951	-1.93779	4.00977	C	5.37202	-2.8356	-0.51135
H	2.28655	0.07931	4.68321	H	5.54306	-2.61459	1.62792
H	2.9677	-3.82666	3.0186	H	4.96129	-2.80429	-2.62815
H	2.70124	-2.35441	5.0116	H	6.18074	-3.55734	-0.58981

Reference:

1. M. J. Fraile, L. K. Jeune, A. J. Mayoral, N. Ravasiob and F. Zaccheriab, *Org. Biomol. Chem.* 2013, **11**, 4327-4332.
2. T. Fukuda, Y. Matsuo, F. Matsuoka, N. Yoshioka, G. Onodera, M. Kimura, F. Ishibashi and M. Iwao, *Heterocycles* 2019, **99**, 1032-1052.
3. F. Beaumard, P. Dauban and R. H. Dodd, *Synth.* 2010, **23**, 4033-4042.
4. J. E. Zweig and T. R. Newhouse, *J. Am. Chem. Soc.* 2017, **139**, 10956-10959.
5. D. Xu, C. Lu and W. ChenXu, *Tetrahedron* 2012, **68,** 1466-1474.
6. R. Rendy, Y. Zhang, A. McElrea, A. Gomez, D. A. Klumpp, *J. Org. Chem.* 2004, **69**, 2340.
7. E. Stadler, A. Eibel, D. Fast, H. Freißmuth, C. Holly, M. Wiech, N. Moszner and G. Gescheidt, *Photochem. Photobiol. Sci*. 2018, **17**, 660–669.
8. R. E. Stratmann, J. C. Burant, G. E. Scuseria and M. J. Frisch, *J. Chem. Phys*. 1997, **106**, 10175-10183.
9. A. D. Becke, *J. Chem. Phys*. 1993, **98**, 5648-5652.
10. M. M. Francl, W. J. Pietro, W. J. Hehre, J. S. Binkley, M. S. Gordon, D. J. Defrees and J. A. Pople, *J. Chem. Phys*. 1982, **77**, 3654-3665.
11. M. J. Frisch, G. W. Trucks, H. B. Schlegel, G. E. Scuseria, M. A. Robb, J. R. Cheeseman, G. Scalmani, V. Barone, B. Mennucci, G. A. Petersson, H. Nakatsuji, M. Caricato, X. Li, H. P. Hratchian, A. F. Izmaylov, J. Bloino, G. Zheng, J. L. Sonnenberg, M. Hada, M. Ehara, K. Toyota, R. Fukuda, J. Hasegawa, M. Ishida, T. Nakajima, Y. Honda, O. Kitao, H. Nakai, T. Vreven, J. A. Montgomery, J. E. Peralta, F. Ogliaro, M. Bearpark, J. J. Heyd, E. Brothers, K. N. Kudin, V. N. Staroverov, R. Kobayashi, J. Normand, K. Raghavachari, A. Rendell, J. C. Burant, S.S. Iyengar, J. Tomasi, M. Cossi, N. Rega, , J. M. Millam, M. Klene, J. E. Knox, J. B. Cross, V. Bakken, C. Adamo, J. Jaramillo, R. Gomperts, R. E. Stratmann, O. Yazyev, A. J. Austin, R. Cammi, C. Pomelli, J. W. Ochterski, R. L. Martin, K. Morokuma, V. G. Zakrzewski, G. A. Voth, P. Salvador, J. J. Dannenberg, S. Dapprich, A. D. Daniels, Ö. Farkas, J. B. Foresman, J. V. Ortiz, J. Cioslowski and D. J. Fox, *Gaussian 09, Revision D.01*, Wallingford CT, 2009.
12. a) C. Jamorski, M. E. Casida and D. R. Salahub, *J. Chem. Phys.* 1996, **104**, 5134-5147. b) M. E. Casida, C. Jamorski, K. C. Casida and D. R. Salahub, *J. Chem. Phys.* 1998, **108**, 4439-4449. c) M. Petersilka, U. Gossmann and E. Gross, *Phys. Rev. Lett.* 1996, **76**, 1212-1215.
